# Reductive cyclotrimerization of CO and isonitriles with a highly reactive Ca^I^ synthon†

**DOI:** 10.1039/d5sc02829a

**Published:** 2025-05-27

**Authors:** Stefan Thum, Jonathan Mai, Marcel A. Schmidt, Jens Langer, Sjoerd Harder

**Affiliations:** a Inorganic and Organometallic Chemistry, Friedrich-Alexander-Universität Erlangen-Nürnberg Egerlandstrasse 1 91058 Erlangen Germany sjoerd.harder@fau.de

## Abstract

Whereas the small molecule activation with β-diketiminate (BDI)Mg^I^ complexes of type (BDI)Mg–Mg(BDI) is extensively investigated, lack of similar Ca^I^ reagents prevents studies on Ca^I^ reactivity. Herein, we report on small molecule activation with dinitrogen complexes of type (BDI)Ca(N_2_)Ca(BDI) which acts as Ca^I^ synthon by release of N_2_ and two electrons. Reaction of [(BDI*)Ca(THP)]_2_(N_2_) with CO led to formation of a deltate product with the cyclic C_3_O_3_^2−^ dianion (1); BDI* = HC[C(Me)N(DIPeP)]_2_ (DIPeP = 2,6-(Et_2_CH)-phenyl) and THP = tetrahydropyran. Reaction with the isonitrile CyN≡C gave as the major product a complex with the triimino deltate C_3_(NCy)_3_^2−^ dianion (2) which is unstable in solution. Isolation of the side-product (BDI*)_2_Ca·(CN–Cy) (3) indicates dynamic ligand exchange and Schlenk equilibria. Variation of the isonitrile reagent led to isolation of (BDI*)_2_Ca·(CN–R) (4: R = xylyl, 5: R = *t*Bu). Crystal structures and NMR studies in solution are discussed for complexes 1–5. We also report an extensive DFT study on the reductive trimerization of MeN≡C with this Ca^I^ synthon. The key intermediate (BDI)Ca(MeNC)Ca(BDI) contains dianionic MeNC^2−^. Contrary to expectation, C–C coupling does not proceed by nucleophilic attack at a second MeNC reagent. Electron transfer results in two bridging MeNC˙^−^ radical anions. This rare singlet diradicaloid reacts further by radical coupling to [MeNC–CNMe]^2−^. Differences with Mg^I^ reactivity are discussed.

## Abstract 2

A strongly reducing Ca^I^ synthon releases N_2_ and two electrons for the reductive C–C coupling of CO and RN≡C.

## Graphical abstract

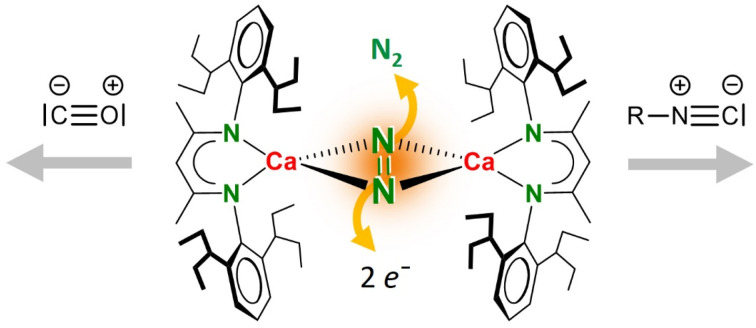

## Introduction

**Scheme 1:**
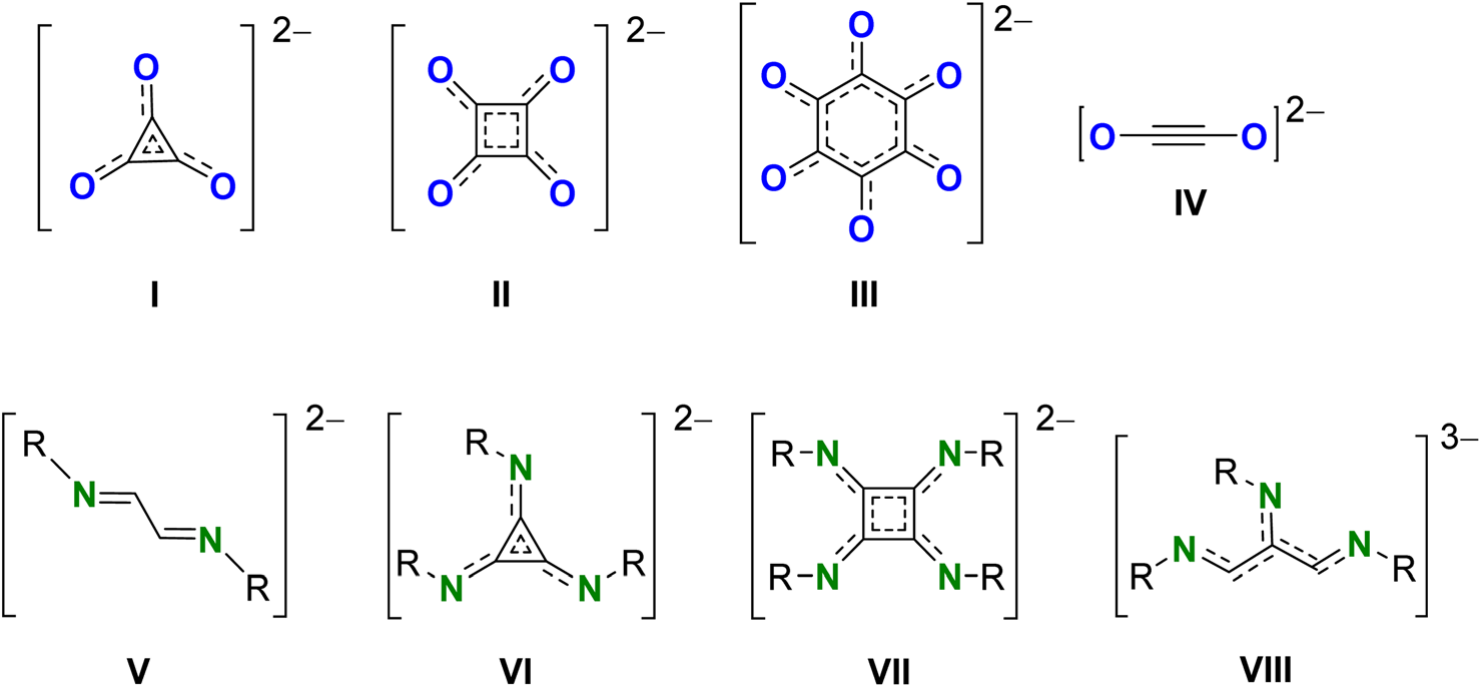
Reductive coupled CO and isonitrile R–NC products (I–VIII).

Carbon monoxide (CO) and isoelectronic isonitriles (RN≡C) are important C1 feedstocks for the preparation of fine chemicals.^1–5^ Especially the bulk reagent CO is a key building block in numerous industrial processes mediated by transition metal (TM) complexes such as the Cativa process for manufacturing acetic acid^6^ and the Fischer–Tropsch process for liquid hydrocarbons.^7^ Recent years have seen rapid growth of the field of main-group metal complexes mimicking TM complexes in their reactivity.^8^ Main group metal-mediated C–C bond formation is of particular interest due to the metal's high natural abundance, low toxicity and eco-friendly reputation compared to late TMs. Within this field, we are particularly interested in low oxidation state *s*-block chemistry.^9–12^ Reductive C–C homologation of CO by low oxidation state magnesium complexes [(^Ar^BDI)Mg]_2_ (^Ar^BDI = HC[C(Me)N(Ar)]_2_; Ar = aryl) is known to give deltates [C_3_O_3_]^2−^ (I, Scheme 1),^13,14^ squarates [C_4_O_4_]^2−^ (II),^15^ benzenehexolates [C_6_O_6_]^2−^ (III),^16^ or more recently, reductive dimerization of CO to ethynediolates [O–C≡C–O]^2−^ (IV).^17–20^ Product selectivity strongly depends on ligand bulk, the presence of catalytic amounts of Mo(CO)_6_ and activation of the Mg–Mg bond by asymmetric solvation and polarization (*vide infra*). There is also rapid development of low-valent *p*-block chemistry: heterobimetallic alkali metal aluminyl complexes have been investigated for CO homologation.^21,22^

As isonitriles are isoelectronic to CO, also the activation of this important feedstock with low oxidation state main group complexes is emerging.^23^ Reductive dimerization of isonitriles to corresponding 1,4-diazabutadiene-2,3-diyl [RN=C–C=NR]^2−^ (V) has been reported for low-valent dialanes,^24–26^ digallanes,^27^ Mg^I^ dimers,^28^ and Ge^I^ dimers.^29^ However, examples for the reductive cyclomerization of isonitriles are limited. Low-valent transition metal complexes have shown reductive isonitrile coupling to triimino deltate C_3_(NR)_3_^2−^ (VI), squaramidinate [C_4_N_4_R_4_]^2–^ (VII) or linear higher homologues (*e.g.*VIII).^26,30–32^ However, to the best of our knowledge, in main group metal chemistry formation of cyclic products could only be achieved with low-valent Al^II^ reagents. Reaction of a dialumane system with isonitriles gave reductive trimerization to triimino deltate C_3_(NR)_3_^2−^ (VI).^26^

The reduction of CO or isonitriles with low-valent alkaline-earth (Ae) metal reagents is so far limited to reactivity studies on Mg^I^ complexes like [(^Ar^BDI)Mg]_2_. This limitation is mainly due to the fact that the low oxidation chemistry of the early main group metals is hardly developed. Apart from complexes of Be^I^,^33,34^ Be^0^,^35,36^ and Mg^0^,^37–39^ there are currently no examples of heavier Ae^I^ or Ae^0^ reagents which are notoriously unstable and highly reactive. Attempts to prepare a [(^Ar^BDI)Ca]_2_ reagent led either to reduction of the aromatic solvent or of the inert gas N_2_ to give [(^Ar^BDI)Ca]_2_(C_6_H_6_) or [(^Ar^BDI)Ca]_2_(N_2_), respectively.^40^ However, it was found that such complexes react like a strongly reducing synthon for [(^Ar^BDI)Ca]_2_ by releasing C_6_H_6_ or N_2_ and two electrons.^40–43^ These complexes therefore enable reduction chemistry with well-defined heavier Ae metal reagents. We herein report the reduction of CO and isonitriles (R–N≡C) with different substituents R with the Ca^I^ synthon [(^Ar^BDI)Ca]_2_(N_2_). Comparing the outcome with well-established Mg^I^ reduction chemistry will demonstrate the influence of the Ae metal centre in such reactions. Our findings will be supported by computational studies.

## Results and discussion

### Reaction with CO

**Scheme 2:**
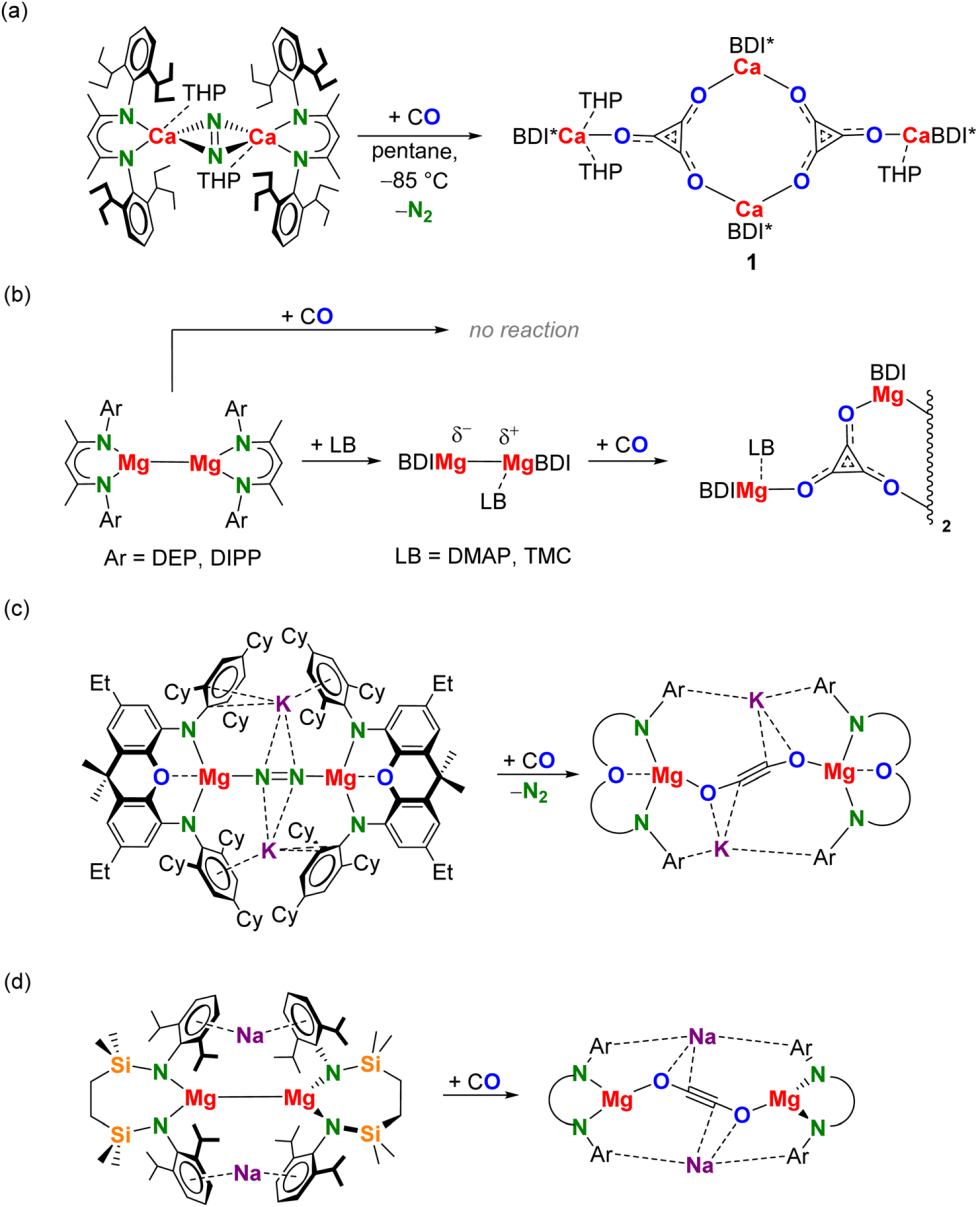
(a) Reaction of [(BDI*)Ca(THP)]_2_(N_2_) with CO. (b) Reaction of Mg^I^ complexes with CO after prior polarization of Mg–Mg bond.^13,14^ DEP = 2,6-Et-phenyl, DIPP = 2,6-iPr-phenyl, DMAP = 4-Me_2_N-pyridine, TMC = :C[N(Me)CMe]_2_. (c) Reaction of a heterobimetallic Mg/K dinitrogen complex with CO.^18^ (d) Reaction of a heterobimetallic Mg/Na complex with CO.^17^

In our reactivity studies we used the Ca^I^ synthon [(BDI*)Ca(THP)]_2_(N_2_); BDI* = HC[C(Me)N(DIPeP)]_2_ (DIPeP = 2,6-(Et_2_CH)-phenyl) and THP = tetrahydropyran (Scheme 2a). In contrast to the THF adduct, which decomposes slowly at room temperature, this THP adduct shows increased stability and can be easily obtained in crystalline purity.^40^ The THP ligand also affects the selectivity of the conversion. Reaction of the non-solvated complex [(BDI*)Ca]_2_(N_2_) with CO in methylcyclohexane-d_14_ led at −85 °C already to rapid conversion (Fig. S30†). However, this reaction is not very selective and many products formed. In contrast, the reaction of the THP adduct is much more selective.

Reacting a red-brown suspension of the Ca^I^ synthon [(BDI*)Ca(THP)]_2_(N_2_) in pentane at −85 °C with 1 bar of CO led upon warming to room temperature to an orange solution and precipitation of [(BDI*)Ca]_4_(THP)_3_(C_3_O_3_)_2_ (1) in form of microcrystalline white solid. After concentration and cooling to −25 °C, colourless crystals suitable for X-ray diffraction analysis were obtained. To monitor the formation of 1 and trap possible intermediates, the reaction was repeated in methylcyclohexane-d_14_. However, ^1^H NMR monitoring only showed quantitative formation of deltate complex 1, indicating that intermediates are likely to be short-lived, transient species.^44^ The ^1^H NMR spectrum of the reaction mixture shows two sets of signals for chemically inequivalent BDI* ligands assigned to isolated 1 (Fig. S29†). Despite the high selectivity of the reaction, the very good solubility induced by the flexible Et_2_CH-groups allowed isolation of the Ca deltate complex 1 in only 20% crystalline yield. Poor yields to high product solubility of complexes with the BDI* ligand is a known problem we already observed in various investigations.^41,42,45^

**Fig. 1:**
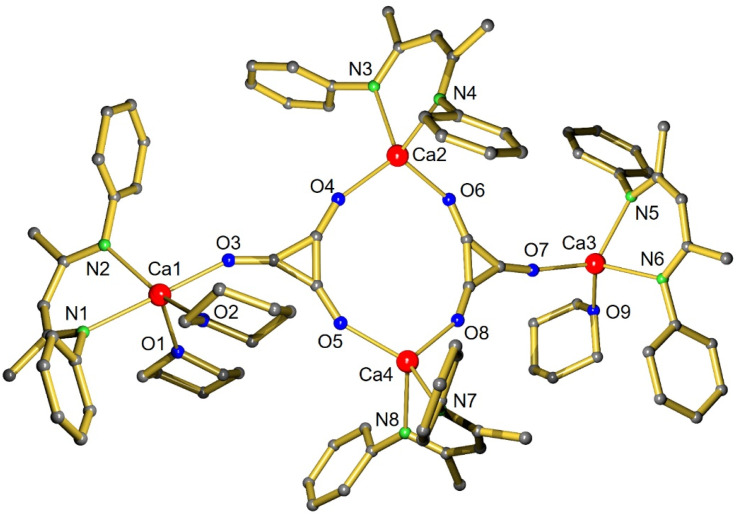
Molecular structure of [(BDI*)Ca]_4_(THP)_3_(C_3_O_3_)_2_ (1). The H atoms and Et_2_CH-substituents have been omitted for clarity.

Product 1 crystallized in the centrosymmetric triclinic space group *P*1̄ with two independent, but similar [(BDI*)Ca]_4_(THP)_3_(C_3_O_3_)_2_ aggregates in the asymmetric unit (Fig. 1). The molecular structure consists of two deltate dianions C_3_O_3_^2−^ bound to two bridging [(BDI*)Ca]^+^ fragments and two terminal [(BDI*)Ca]^+^ units. The Ca atoms in the terminal [(BDI*)Ca]^+^ units are additionally saturated by either one or two THP ligands. Both deltate dianions are planar with C–C–C angles close to 60° (59.8(2)–60.5(2)°). The C–C bond lengths between 1.397(3) Å and 1.410(3) Å and C–O bond lengths ranging from 1.276(3) Å to 1.289(7) Å are in agreement with the expected delocalization of π-electrons over the C_3_O_3_ core. Reports about the reductive trimerization of CO to C_3_O_3_^2−^ are limited to U^III46^ and Mg^I13,14^ complexes. The aromatic character of the C_3_ cycle in 1 is similar to the deltate dianions in (BDI)Mg complexes with C–C bond lengths (1.391(2)–1.402(2) Å;^13^ 1.396(3)–1.399(3) Å;^13^ 1.385(5)–1.399(6) Å)^14^ and C–O bond lengths (1.269(2)–1.288(2) Å;^13^ 1.276(3)–1.280(3) Å;^13^ 1.273(5)–1.278(5) Å)^14^ in comparable ranges. However, the highly symmetric deltate structure in 1 differs from the irregular deltate structure in an uranium complex which is caused by different coordination modes.^46^

One remarkable difference between reduction of CO with Mg^I^ reagents or the Ca^I^ synthon is the considerable higher reactivity of the latter. Symmetric Mg^I^ complexes of type (BDI)MgMg(BDI) do not react with CO and need to be activated with a Lewis base (LB) (Scheme 2b).^13,14^ Whereas addition of two LB ligands does result in elongation of the Mg–Mg bond, it also gives steric congestion, preventing Mg–CO coordination and further reactivity. However, addition of one equivalent of LB was shown to give a polarized, activated Mg–Mg bond (BDI)Mg^*δ*−^–^*δ*+^Mg(LB)(BDI) but leaves room for CO coordination at the second Mg centre. In contrast, the non-solvated Ca^I^ synthon [(BDI*)Ca]_2_(N_2_) does not need any activation and reacts instantaneously with CO. The role of the THP ligands is to control the selectivity of the reaction to deltate formation (1). The observed deltate formation contrasts with the CO reactivity of heterobimetallic Mg/K dinitrogen complex which acts as a synthon for a Mg^I^ radical and readily reduced CO to ethynediolate [O–C≡C–O]^2−^ (Scheme 2c).^18^ It also contrasts with the Mg^I^ reactivity of Hill's heterobimetallic Mg/Na complex which similarly led to [O–C≡C–O]^2−^ formation (Scheme 2d).^17^ Noteworthy, Jones and coworkers recently introduced a highly reactive heterobimetallic Ca/K dinitrogen complex that instantly reacts at room temperature with CO but only gave intractable product mixtures.^47^

### Reaction with isonitriles (R–N≡C)

**Scheme 3:**
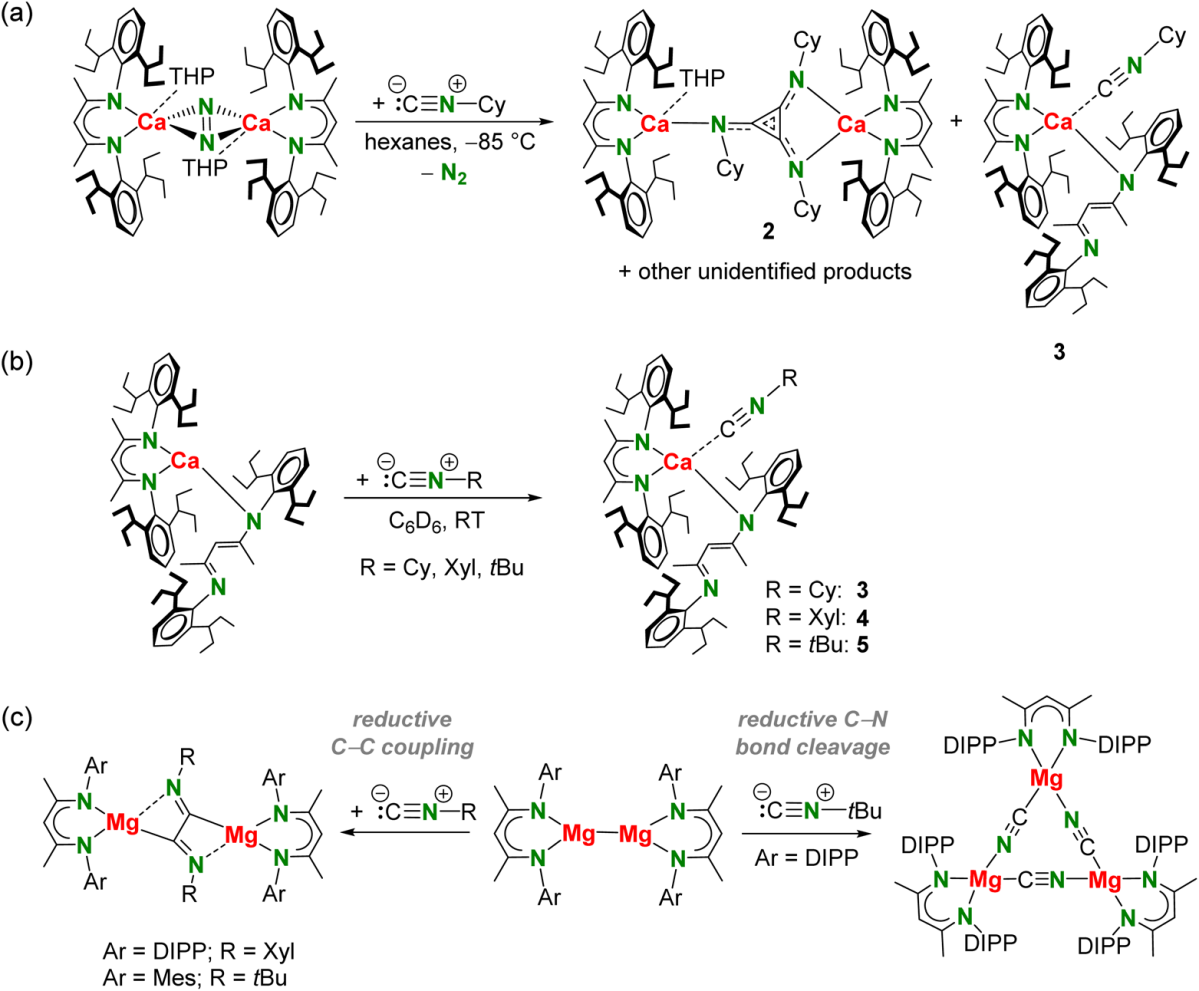
(a) Reaction of Ca^I^ synthon [(BDI*)Ca(THP)]_2_(N_2_) with Cy–NC. (b) Reaction of (BDI*)_2_Ca with R–NC. (c) Reactivity of [(^Ar^BDI)Mg]_2_ with isonitrile R–NC depending on substituents Ar and R.^28^ Xyl = 2,6-dimethyl-phenyl, Mes = 2,4,6-trimethyl-phenyl.

Dropwise addition of a hexanes solution of cyclohexyl isonitrile (Cy–NC, 3.2 equivalents) to a red-brown suspension of [(BDI*)Ca(THP)]_2_(N_2_) in hexanes at −85 °C resulted upon warming to 0 °C in a dark yellow-brown solution. The ^1^H NMR spectrum of the crude reaction mixture in benzene-d_6_ showed relatively selective formation of one major product with one major sharp singlet for the methine in the ligand backbone among smaller signals of minor side products (Fig. S31†). Changing the stoichiometry did not improve selectivity. The product could be isolated in the form of orange crystals suitable for X-ray diffraction analysis by storing a concentrated hexanes solution at −25 °C. This revealed reductive trimerization of Cy–NC to the triimino deltate dianion C_3_(NCy)_3_^2−^ and gave [(BDI*)Ca]_2_(C_3_(NCy)_3_)(THP) (2) in 27% isolated yield (Scheme 3a). A second crop of crystals gave a mixture of 2 and (BDI*)_2_Ca·(CN–Cy) (3). The latter side-product could be obtained in 10% isolated yield by isolation of a third crop of crystals. The formation of complex 3 shows that ligand exchange by Schlenk equilibria are operative. The reactant stoichiometry changes the product selectivity. Reaction of [(BDI*)Ca(THP)]_2_(N_2_) with only two equivalents gave less of the triimino deltate product 2 but more of 3 (Fig. S35†). Alternatively, complex 3 could be obtained in a high yield of 81% by reacting the homoleptic complex (BDI*)_2_Ca with one equivalent of Cy–NC in benzene (Scheme 3b).

The activation of Cy–NC with the Ca^I^ synthon stands in contrast to reported isonitrile reactivity with Mg^I^ complexes. The course of the reaction with low oxidation state Mg complexes of type [(^Ar^BDI)Mg]_2_ depends on the steric bulk of the Ar substituent and on the organic moiety R of the R–NC reagent (Scheme 3c).^28^ A bulky DIPP substituent and bulky *t*Bu groups resulted in a trimeric Mg cyanide complex which was formed by reductive R–NC bond cleavage. Small Ar (Ar = Mes) and bulky R (R = *t*Bu) as well as the combination of bulky Ar (Ar = DIPP) and smaller R (R = Xyl) groups led to reductive dimerization, *i.e.* [RN=C–C=NR]^2−^ flanked by two [(^Ar^BDI)Mg]^+^ units.

Since product formation by reduction of R–NC with Mg^I^ dimers strongly depends on the steric bulk of the Ar and R substituents,^28^ the influence of the substituent R in reduction of R–NC with the Ca^I^ synthon was investigated. Addition of a Xyl–NC solution in hexanes to a red-brown suspension of [(BDI*)Ca(THP)]_2_(N_2_) in hexanes at −85 °C resulted upon warming to 0 °C in a dark purple solution. ^1^H NMR spectroscopy indicated formation of several products (Fig. S37†) and only (BDI*)_2_Ca·(CN–Xyl) (4) could be isolated in 12% crystalline yield. Using bulky *t*Bu–NC resulted in a more selective reaction outcome with one major backbone signal for the methine in the ligand backbone but prolonged crystallization times led to formation of several side-products (Fig. S38 and S39†) and only (BDI*)_2_Ca·(CN–*t*Bu) (5) could be isolated. Complexes 4 and 5 could be isolated in high yields (85–91%) from a solution of homoleptic complex (BDI*)_2_Ca and the corresponding R–NC in benzene (Scheme 3b). All complexes 2–5 have been fully characterized by NMR methods, elemental analyses and single crystal X-ray diffraction.

**Fig. 2:**
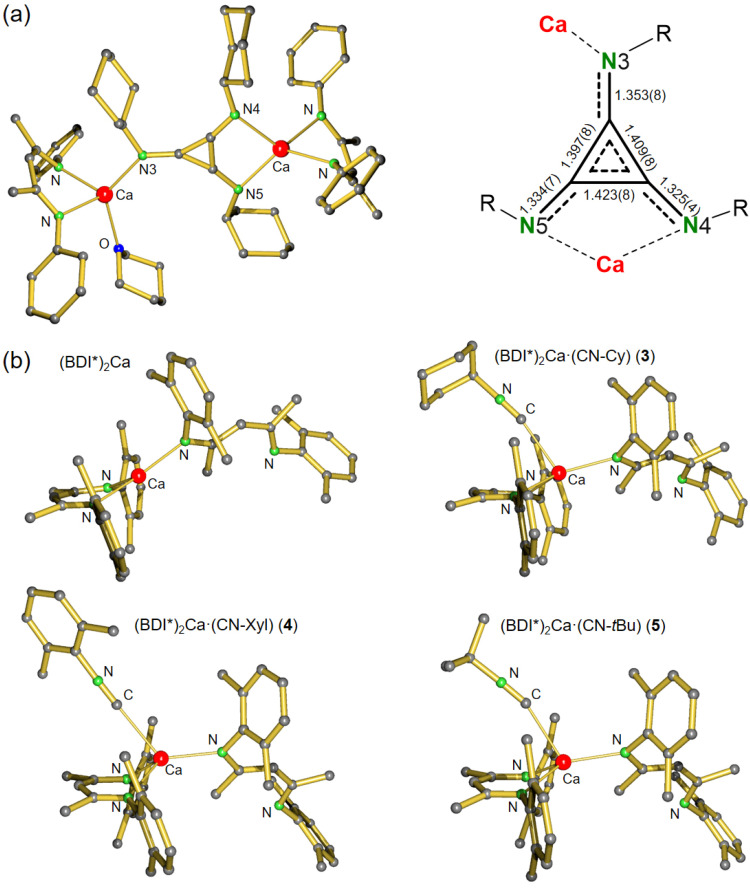
(a) Crystal structure of [(BDI*)Ca]_2_(C_3_(NCy)_3_)(THP) (2) and a detailed view of the triimino deltate dianion C_3_(NCy)_3_^2−^ with bond distances in Å. H atoms and Et_2_CH-groups have been omitted for clarity. (b) Comparison of the crystal structures of (BDI*)_2_Ca^40^ with the isonitrile adducts 3–5. H atoms and the Et groups of the Et_2_CH substituents have been omitted for clarity.

The crystal structure of 2 shows a C_3_(NCy)_3_^2−^ dianion that is bridging two [(BDI*)Ca]^+^ fragments in an asymmetric η^1^:η^2^ fashion (Fig. 2a). A similar coordination mode was recently found in an aluminium triimino deltate complex.^26^ The C_3_(NCy)_3_^2−^ dianion in 2 is disordered over an inversion centre in the middle of the molecule (Fig. S45†). One of the Ca centres is additionally coordinated by one THP molecule. The C_3_(NR)_3_ unit, a [3]radialene derivative, in 2 is of great interest as building block for polymers, organic conductors and ferromagnets.^48–51^ The expected aromatic character of the dianion is confirmed by inspection of the bond distances in C_3_(NCy)_3_^2−^. The C–C bond distances ranging from 1.397(8)–1.423(8) Å and C–N bond distances from 1.334(7)–1.353(8) Å are in between corresponding double and single bonds,^52^ thus supporting electronic delocalization over the entire C_3_N_3_ core. The bond distances for the triimino deltate dianion are in similar ranges to those observed in the dialumane system for C_3_(N*t*Bu)_3_^2−^ (C–C: 1.381(4)–1.402(4) Å, C–N: 1.341(3)–1.366(3) Å)^26^ and in the vanadium complex of C_3_(NXyl)_3_^2−^ (C–C: 1.388(5)–1.427(5) Å, C–N: 1.324(5)–1.369(4) Å).^31^ The C_3_ ring in 2 exhibits an almost perfect triangular geometry with internal C–C–C angles ranging from 59.1(4)° to 61.0(4)°, similar to those reported for a dialumane system (59.3(2)–60.8(2)°).^26^ The Ca complex 2 represents the first reductive *cyclo*-trimerization of an isonitrile promoted by an *s*-block metal complex. Despite its flexible Et_2_CH-groups, complex 2 shows only moderate solubility in aromatic and aliphatic solvents. Moreover, 2 is not stable in solution and decomposes at room temperature to unidentified products and meaningful ^13^C NMR data could not be obtained (Fig. S32 and S33†). The ^1^H NMR spectrum of 2 shows one characteristic signal for the methine in the ligand backbone indicating rapid fluctuation of the THP molecule between the two Ca centres in solution. Cooling the sample to −20 °C led to appearance of several new methine backbone signals (Fig. S34†) indicating formation of various species. Warming to room temperature resulted in coalescence to one signal, showing that this process is reversible. However, further heating to +80 °C gave irreversible decomposition of 2 in various unidentified species. These combined observations show that the products are not very stable and in dynamic exchange.

The crystal structures of (BDI*)_2_Ca·(CN–R) (R = Cy, Xyl, *t*Bu) (3–5, Fig. 2b) have in common that one BDI* ligand coordinates in η^2^-fashion while the other one is only η^1^-coordinated due to the steric demand of the bulky DIPeP-substituents. As the reported crystal structure of (BDI*)_2_Ca^40^ shows a similar combination of η^1^- and η^2^-coordination, this comes as no surprise. Comparing the four structures shows remarkable similarities (Fig. 2b, Table 1). Due to the needle-like form of the isonitrile ligands, coordination to Ca only needs minimal space. In all complexes 3–5, the isonitrile ligand hovers above the bidentate *N*,*N*-chelating BDI* ligand. Coordination of an additional isonitrile ligand hardly affects the Ca–N bond distances in (BDI*)_2_Ca. Elongations between 0.022 and 0.075 Å are observed (Table 1). The Ca–C bond distances range from 2.578(2) Å for the Cy–NC complex (3) to 2.664(2) Å in the Xyl–NC complex (4). Since the Ca–C bond in 5 with the bulky *t*Bu–NC ligand falls in between (2.599(2) Å) the poorer donor ability of Xyl–NC may be due to differences in electronic effects, *i.e.* partial delocalization of π-electron density of the C≡N bond in the xylyl ring.

**Table 1:** Comparison of selected bond lengths (Å) in (BDI*)_2_Ca·(CN–R) complexes (3–5) with those in (BDI*)_2_Ca

Complex	(BDI*)_2_Ca	(BDI*)_2_Ca·(CN–Cy) (3)	(BDI*)_2_Ca·(CN–Xyl) (4)	(BDI*)_2_Ca·(CN–*t*Bu) (5)
Ca–N (η^2^–BDI*)^a^	2.330	2.355	2.354	2.352
Ca–N (η^1^–BDI*)	2.290(1)	2.365(2)	2.362(1)	2.340(1)
Ca–C (isonitrile)	—	2.578(2)	2.664(2)	2.599(2)
C≡N	—	1.147(2)	1.156(2)	1.180(8)^b^

a Average value.

b Large standard deviation due to disorder.

Solutions of complexes 3–5 in C_6_D_6_ show ^1^H NMR spectra (Fig. S11, S17 and S23†) which show strong similarity to that of (BDI*)_2_Ca. However, all signals are shifted and partially broadened which indicates that there is in solution dynamic coordination of the isonitrile ligands.

### Computational and mechanistic studies

The complete structures of the Ca deltate 1 and Ca triimino deltate 2 were optimized at the B3PW91/def2tzvp//def2svp level of theory. The calculated geometries of the full aggregates fit reasonably well with the crystal structures (Fig. S49 and S50†), indicating a sufficient level of theory. Natural Population Analysis (NPA) (Fig. S51 and S52†) confirms that both complexes are ionically bound (NPA charges in 1: Ca +1.82, deltate anion −1.86; NPA charges in 2: Ca +1.79, triimino deltate −1.78). The C–C bonds in the C_3_-ring in the deltate anion C_3_O_3_^2−^ show Wiberg Bond Indices (WBI's) between 1.18–1.22, supporting delocalized single/double bonds (Fig. S53†). The C–O bonds are part of the delocalized system and show WBI's in the range of 1.23–1.29. A similar bonding situation is found in the triimino deltate C_3_(NCy)_3_^2−^ (Fig. S54†) with C–C bonds WBI's in the range of 1.15–1.20, and WBI's for deltate C–N bonds in the range of 1.25–1.38. Atoms-In-Molecule (AIM) analysis for Ca deltate 1 shows that the C_3_O_3_^2−^ dianion is not only bound to the Ca^2+^ cations but is also integrated in a network of O⋯H–C bonding interaction with the BDI* and THP ligands (Fig. S56†). Although such non-classical hydrogen bonding was once ridiculed,^53^ these weak interactions have been shown important in determining structure and reactivity.^54^ Such non-classical hydrogen bonds are enforced by high electron density on the acceptor side.^55^ As the O atoms in C_3_O_3_^2−^ carry most of the negative charge (NPA charges range from −0.88 to −0.93), the O⋯H–C bonding network in 1 should be considered important.

Although there are several computational studies on reductive CO homologation with low-valent Mg^I^ complexes,^13,14,16^ reductive trimerization of isonitrile with *s*-block metal reagents is so far unexplored. To gain further insights in the mechanism of isonitrile reduction with a Ca^I^ synthon, a DFT study at the B3PW91-GD3BJ/def2tzvp//B3PW91-GD3BJ/def2svp level of theory was conducted. Due to size limitations the DIPeP-substituents in the BDI* ligand have been replaced with smaller DIPP-substituents and Cy–NC was modelled with Me–NC.

**Scheme 4:**
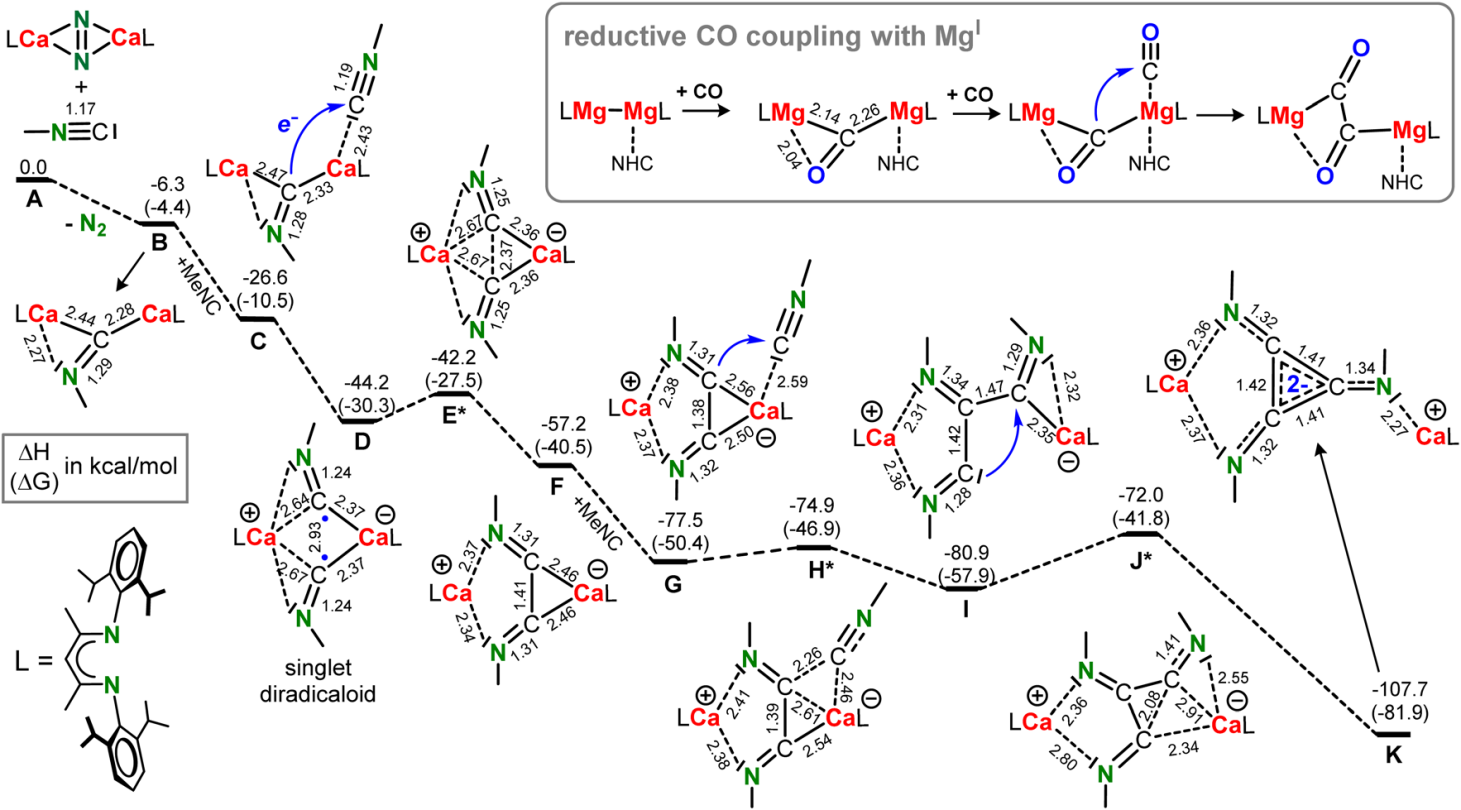
Reductive coupling of isonitrile. Energy profile for triimino deltate formation calculated at the B3PW91-GD3BJ/def2tzvp//B3PW91-GD3BJ/def2svp level of theory for a model system with L = ^DIPP^BDI and MeN≡C. Δ*H* in kcal mol^−1^. Between brackets: Δ*G* (298 K) in kcal mol^−1^. Selected bond lengths are given in Å. Inset: For comparison, reductive CO dimerization with (^DIPP^BDI)Mg–Mg(^DIPP^BDI) activated with a N-heterocyclic carbene (NHC) follows a different pathway.^13^

**Fig. 3:**
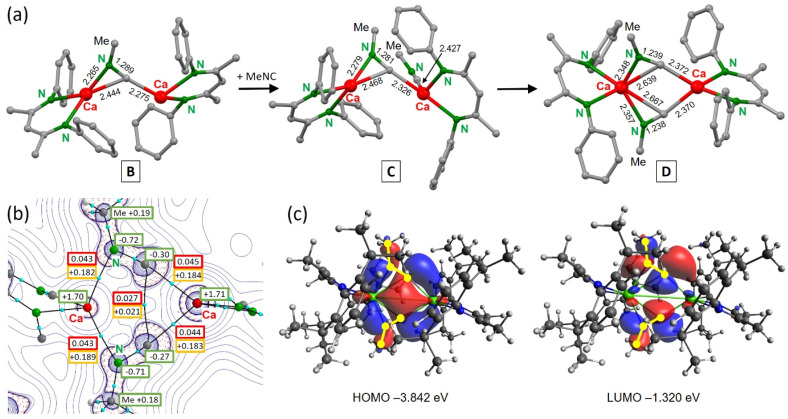
(a) Calculated intermediates on the pathway for reductive trimerization of MeN≡C with (^DIPP^BDI)Ca(N_2_)Ca(^DIPP^BDI). Selected bond distances in Å. (b) Atoms-In-Molecules (AIM) analysis for the singlet diradicaloid D showing bond paths and bond-critical-points (bcp in blue). NPA charges are shown in green boxes. The electron density *ρ*(**r**) and Laplacian ∇^2^*ρ*(**r**) are shown in a.u. in red and orange boxes, respectively. (c) HOMO and LUMO for singlet diradicaloid D. Ca is shown in green and [MeNC˙]^–^ in yellow.

The energy profile for reaction of (^DIPP^BDI)Ca(N_2_)Ca(^DIPP^BDI) with MeN≡C is shown in Scheme 4. The N_2_ complex reacts as a synthon for (^DIPP^BDI)Ca–Ca(^DIPP^BDI) by releasing N_2_ and transferring two electrons to the isonitrile (A–B). This formal N_2_^2−^/MeN≡C to N_2_/[MeN≡C]^2−^ exchange is exothermic by Δ*H* = −6.3 kcal mol^−1^ (Δ*G* = −4.4 kcal mol^−1^). Note that reaction of the Ca^I^ complex (^DIPP^BDI)Ca–Ca(^DIPP^BDI) with MeN≡C would be considerably more exothermic: Δ*H* = −40.6 kcal mol^−1^, Δ*G* = −25.9 kcal mol^−1^. The NPA charge on the bridging isonitrile in B is −1.60, reflecting its dianionic state (Fig. S64†). The [MeN≡C]^2−^ anion is bent (C–N–C 121.0°) and bridges asymmetrically between the Ca^2+^ cations with two unequal Ca–C contacts and one Ca–N bond (Fig. 3a). This differs from the reaction of (^Mes^BDI)Mg–Mg(^Mes^BDI) with CO which is endothermic by +9.4 kcal mol^−1^ and forms a product with a symmetrically bridging CO^2−^ anion between the Mg^2+^ centres (side-on bonding with two equal Mg–C and two equal Mg–O contacts).^16^ However, when the bulkier ^DIPP^BDI is used and one of the Mg centres is solvated with a N-heterocyclic carbene (NHC),^13^ this reaction becomes slightly exothermic (Δ*H* = −7.0 kcal mol^−1^) and CO bridges asymmetrically like the [MeNC]^2−^ anion in B (see inset in Scheme 4). For comparison, the reduction of ArN≡C with (^DIPP^BDI)Al^I^ was calculated to be endothermic by more than 10 kcal mol^−1^.^56^ The exothermic reduction of MeN≡C by (^DIPP^BDI)Ca(N_2_)Ca(^DIPP^BDI) is therefore a demonstration of the considerable reducing power of this N_2_ complex.

The next step in the reaction (B–C) is coordination of a second MeN≡C reagent to one of the Ca centres which is exothermic by Δ*H* = −20.3 kcal mol^−1^. Starting from this coordination complex C, we searched for the transition state for C–C bond formation, assuming C-nucleophilic attack of [MeNC]^2−^ at the C atom in the neutral MeN≡C ligand. To our surprise, we located an energy minimum which is highly symmetric, showing two nearly identical MeN≡C moieties bridging between [(BDI*)Ca]^+^ fragments (D); Fig. 3a. The negative charges on the fragments are similar (−0.80 and −0.83) and there is a rather short C⋯C distance of 2.934 Å, which is substantially shorter than the van der Waals equilibrium distance for two non-bound C atoms, *i.e.* the layer distance in graphite (3.35 Å). AIM analysis of D (Fig. 3b) shows indeed a clear bond path and bond-critical-point (bcp) between these C atoms. The electron density (*ρ*(**r**) = 0.021 e × B^−3^) and Laplacian (∇^2^*ρ*(**r**) = +0.027 e × B^−5^) are small but significant and confirm weak bonding. Optimization of this minimum in the triplet state resulted in a much longer C⋯C bond separation of 3.518 Å (Fig. S60†), *i.e.* an interatomic distance just above the van der Waals equilibrium distance of 3.35 Å. The unrestricted triplet state is only +3.1 kcal mol^−1^ higher in energy than the restricted singlet state. Optimization as an unrestricted singlet gave a similar minimum and energy as found in the restricted singlet optimization. The similar negative charges (−0.80 and −0.83) on both nearly identical MeN≡C moieties indicate bridging radical anions [MeNC˙]^−^ with anti-ferromagnetically coupled radical centres. Such singlet diradicals are known to be highly reactive intermediates in bond-breaking and bond-formation processes.^57–60^ Starting with the Niecke diradical,^61,62^ many examples of such fascinating species based on group 13, 14 or 15 elements could be isolated as stable diradicaloids.^63^ The highly reactive intermediate D with its open-shell singlet biradical character represents the first example of such a *s*-block substituted transient diradicaloid. The HOMO of diradicaloid D shows a bonding interaction between the C atoms which involves overlap of C p-orbitals but also has contributions of Ca d-orbitals (Table S2†). The LUMO is antibonding in respect of the two C atoms.

Although D is a minimum on the energy surface, isolation of such a diradicaloid is not possible. Coupling of the [MeNC˙]^−^ radical anions is nearly barrier-free (D–E*: +2.0 kcal mol^−1^) and generates a complex with a bridging [MeN=C–C=NMe]^2−^ anion with a C–C bond length of 1.407 Å (F). This reactivity is fundamentally different from the reductive coupling of C≡O with (^DIPP^BDI)Mg–Mg(^DIPP^BDI) which starts with formation of the dianion [CO]^2−^ that as a nucleophile attacks a neutral C≡O ligand (see inset in Scheme 4).^13^

After formation of F, coordination of the third MeN≡C reagent (G) is followed by immediate insertion, again a process with hardly any barrier (G–H*: +2.6 kcal mol^−1^). This gives a species with a linear triimino dianion (I). The ring closure in the final step requires only an activation enthalpy of Δ*H* = +8.9 kcal mol^−1^ (I–J*) to form the triimino deltate dianion stabilized by two [(BDI)Ca]^+^ fragments (K). Considering the highly strained nature of the cyclopropane framework, this is a very modest energy barrier. As often observed in cyclopropane synthesis, this ring closing step proceeds through a carbene intermediate (I). A recent computational study by Munz and Chu also provided evidence for carbene intermediates in isonitrile homologation by (^DIPP^BDI)Al^I^.^56^ However, similar as reported for reductive C–C coupling of isonitriles promoted by aluminyl anions,^64^ also in this case cyclopropane formation was not observed. Although there are examples of reductive isonitrile trimerizations to triimino deltates with transition metals^30,31^ or dialumane species,^26^ this is a first example for an *s*-block mediated reaction. The energy profile in Scheme 4 demonstrates that alternative C–C coupling products like the product from reductive isonitrile dimerization (intermediate F) or the open trimer (I) are higher in energy than the closed deltate trimer (K). Overall, the Ca-mediated reductive trimerization of MeN≡C to the deltate complex K is a highly exothermic process with Δ*H* = −107.7 kcal mol^−1^ (Δ*G* = −81.9 kcal mol^−1^).

## Conclusion

In contrast to the comprehensively investigated reactivity of (BDI)Mg–Mg(BDI) complexes,^9^ the chemistry of similar but hypothetical (BDI)Ca–Ca(BDI) complexes is fully unknown. Reaction of a closely related Ca^I^ synthon, [(BDI*)Ca(THP)]_2_(N_2_), with CO led to reductive trimerization and gave a product with the deltate dianion C_3_O_3_^2−^ (1). A similar product was observed for reductive coupling of CO with (BDI)Mg–Mg(BDI) complexes. However, the important difference is that the Ca^I^ synthon is even at low temperature highly reactive.

Reaction of Ca^I^ synthon [(BDI*)Ca(THP)]_2_(N_2_) with CyN≡C also led to a cyclic product (2) and a complex with the triimino deltate dianion C_3_(NCy)_3_^2−^ was isolated. This reaction is not fully selective and the isolation of the side-product (BDI*)_2_Ca·(CN–Cy) (3) shows that the reaction products are in dynamic exchange by Schlenk equilibria. Although reductive coupling of isonitriles to triimino deltates has been demonstrated for transition metal reagents or a dialumane, this reactivity represents a first example for such products formed by *s*-block metal mediated isonitrile homologation. For comparison, reaction of isonitriles with (BDI)Mg–Mg(BDI) complexes led to reductive dimerization to [RN=C–C=NR]^2–^ or reductive R–NC bond cleavage.

A computational study on Ca mediated triimino deltate formation showed a mechanism in which the first C–C coupling proceeds through a singlet diradical minimum. This is fundamentally different from CO coupling by low oxidation state (BDI)Mg–Mg(BDI) complexes which has been calculated to go through CO^2−^ → CO nucleophilic attack.

As low oxidation state (BDI)Ca–Ca(BDI) reagents are currently not accessible, direct comparison with (BDI)Mg–Mg(BDI) reactivity is not possible. However, the highly reducing Ca^I^ synthon [(BDI*)Ca(THP)]_2_(N_2_) provides a good alternative to study metal influences in *s*-block metal mediated reduction reactions.

## Author contributions

S. Thum: conceptualization, investigation, validation, formal analysis, writing – original draft, visualization. J. Mai: investigation, validation, formal analysis. M. A. Schmidt: investigation, validation, formal analysis. J. Langer: formal analysis, validation. Sjoerd Harder: conceptualization, writing – original draft – review and editing, visualization, validation, supervision, project administration.

## Data availability

All primary data are available in the ESI.†

## Conflicts of interest

There are no conflicts to declare.

## Supplementary Material

SC-OLF-D5SC02829A-s001

SC-OLF-D5SC02829A-s002
