# Supplementary material for: Reductive cyclotrimerization of CO and isonitriles with a highly reactive CaI synthon
Source: Chem Sci. 2025 May 27;16(26):12058–67. doi: 10.1039/d5sc02829a (PMC12135021; doi:10.1039/d5sc02829a)
Supplement: SC-016-D5SC02829A-s001 [file SC-016-D5SC02829A-s001.pdf]

Supplementary Information (SI) for Chemical Science.
This journal is © The Royal Society of Chemistry 2025

Supporting Information

Table of Contents

1. Materials and Methods S2
2. Synthetic Procedures S3
3. Spectroscopic Characterization S7
4. NMR Studies S22
5. Crystal Structure Determinations S35
6. Computational Details S45
7. References S95

1. Materials and Methods

All experiments were conducted in dry glassware under an inert nitrogen or argon atmosphere by applying standard Schlenk techniques or gloveboxes (MBraun) using freshly dried and degassed solvents. Methylcyclohexane, hexanes, and pentanes, were degassed with nitrogen, dried over a column with activated aluminum oxide (Innovative Technology, Pure Solv 400-4-MD, Solvent Purification System) and then stored under inert atmosphere over molecular sieves (3 Å). Tetrahydropyran (THP) was dried over freshly grounded CaH_2, distilled and stored over molecular sieves (3 Å) under inert atmosphere. Deuterated benzene (C_6D_6), methylcyclohexane (C_7D_{14}), and toluene (C_7D_8) were purchased either from Deutero GmbH or Sigma Aldrich, degassed and dried over molecular sieves (3 Å) and stored under an inert atmosphere. Following reagents were obtained commercially and used without further purification: Xyl–NC (Thermo Fisher Scientific), Cy–NC (Thermo Scientific, 99%), and *t*Bu–NC (TCI Chemicals, >95.0%). Research grade CO (99.999%) was purchased from Air Liquide and used as received. The following compounds were prepared according to literature procedures: $[(BDI^*)Ca(THP)]_2(N_2)$;[S1] $(BDI^*)_2Ca$;[S1] BDI* = $HC[C(Me)–N(DIPeP)]_2$, DIPeP = 2,6-(Et_2CH)-phenyl.

Infrared spectra were acquired on a Bruker Alpha II FT-IR spectrometer equipped with a Platinum ATR diamond from the neat compounds under inert conditions inside a glovebox. All spectra were recorded at room temperature in the range of 400–4000 cm^{-1} with a resolution of 4 cm^{-1} and baseline corrected. Wavenumbers $\tilde{\nu}$ are given in cm^{-1} and intensities of IR bands are described using the following terms: s = strong, m = medium and w = weak. NMR spectra were measured on Bruker Avance III H 400 MHz and Bruker Avance III HD 600 MHz NMR spectrometers. Chemical shifts (δ) are denoted in ppm (parts per million) and coupling constants in Hz (Hertz). ^1H and ^{13}C NMR spectra were referenced to the solvent residual signal ($SiMe_4$ = 0 ppm). Signal multiplicities are described using common abbreviations: s (singlet), d (doublet), t (triplet), q (quartet), quint (quintet), m (multiplet) and br (broad). Elemental analysis was performed with a Hekatech Eurovector EA3000 analyzer. All crystal structures have been measured on a SuperNova (Agilent) diffractometer with dual Cu and Mo microfocus sources and an Atlas S2 detector.

2. Synthetic Procedures

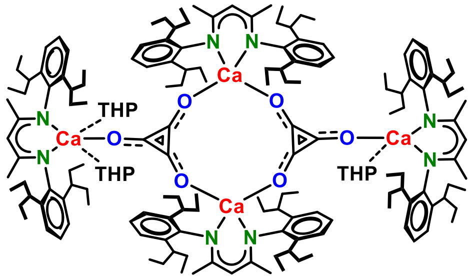

Synthesis of [(BDI*)Ca]$_4$(THP)$_3$(C_3O_3)$_2$ (1) [(BDI*)Ca(THP)]$_2$(N_2) (64.0 mg, 47.8 µmol) was suspended in precooled pentane (–25 °C, 2.0 mL). The suspension was degassed *via* two freeze-pump-thaw cycles and cooled to –85 °C. At this temperature, the flask was then backfilled with CO gas (1 atm) and sealed. Upon warming to room temperature, a white solid precipitated. After decantation of the pentane solution, the solid was dried *in* vacuo and [(BDI*)Ca]$_4$(THP)$_3$(C_3O_3)$_2$ (**1**) was isolated as microcrystalline solid. Crystals suitable for X-Ray diffraction analysis were obtained from the pentane solution after concentration to approximately half of its original volume and filtration at – 25 °C. The supernatant was decanted and the crystals were washed with cold pentane (–25 °C, 2 x 1 mL) and dried *in vacuo* (overall yield: 28 mg, 9.58 µmol, 20%).

^{1}H NMR (600.13 MHz, C_6D_6, 298K): δ = 0.81–0.86 (m, 24H, C**H$_3$**), 0.89–0.92 (m, 12H, C**H$_3$**), 0.95–0.98 (m, 12H, C**H$_3$**), 1.44 (s, 3H, C**H$_3$**-backbone), 1.57 (s, 6H, C**H$_3$**-backbone) 1.59–1.83 (m, 32H, C**H$_2$**), 2.28 (s, 6H, C**H$_3$**-Xyl), 2.60 (s, 3H, C**H$_3$**-backbone), 2.75 (m, 2H, C**H**), 2.90 (m, 2H, C**H**), 3.00 (m, 2H, C**H**), 3.87 (s, 1H, C**H**-backbone), 4.76 (s, 1H, C**H**-backbone), 6.61 (d, *J* = 7.7 Hz, 2H, C**H**-Xyl), 6.76 (t, *J* = 7.7 Hz, 1H, C**H**-Xyl), 6.99–7.01 (m, 4H, C**H**-arom.), 7.06–7.10 (m, 8H, C**H**-arom.) ppm.

^{13}C{^{1}H} NMR (150.92 MHz, C_6D_6, 298K): δ = 11.8 (**C**H$_3$), 11.9 (**C**H$_3$), 12.4 (**C**H$_3$), 22.8 (γ-**C**H$_2$-THP), 25.5 (**C**H$_3$-backbone), 25.6 (**C**H$_3$-backbone), 26.1 (β-**C**H$_2$-THP), 27.7 (**C**H$_2$), 27.9 (**C**H$_2$), 28.8 (**C**H$_2$), 28.9 (**C**H$_2$), 41.1 (**C**H), 41.5 (**C**H), 70.2 (α-**C**H$_2$-THP), 90.5 (**C**H-backbone), 92.4 (**C**H-backbone), 123.0 (**C**-arom.), 123.9 (**C**-arom.), 124.8 (**C**-arom.), 125.1 (**C**-arom.), 138.0 (**C**-arom.), 139.3 (**C**-arom.), 149.7 (**C**-arom.), 150.4 (**C**-arom.), 165.3 (**C**N-backbone), 166.6 (**C**N-backbone) ppm.

FT-IR (ATR, pure): $\tilde{\nu}$ = 2969 (m), 2927 (m), 2870 (w), 1504 (s), 1435 (s), 1400 (s), 1169 (m), 990 (m) cm^{-1}.

Elemental analysis Calculated for $C_{169}H_{258}Ca_4N_8O_9 \cdot 3\ C_5H_{12}$ (M = 2922.74 g/mol): C 75.61, H 10.14, N 3.83; Found: C 76.01 H 9.88, N 4.27.

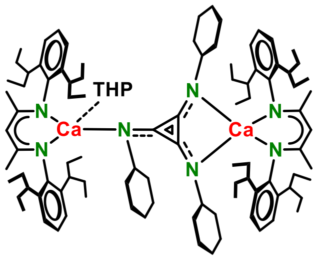

Synthesis of [(BDI*)Ca]$_2$(C$_3$(NCy)$_3$)(THP) (2) [(BDI*)Ca(THP)]$_2$(N$_2$) (75.0 mg, 56.0 µmol, 1.00 eq.) was suspended in precooled hexanes (–25 °C, 3 mL) The suspension was cooled to –85 °C. At this temperature Cy–NC (22.0 µL, 19.6 mg, 179.2 µmol, 3.20 eq.) diluted in precooled hexanes (–25 °C, 500 µL) was dropwise added under vigorous stirring. Upon warming to 0 °C over a course of 2 h, a brown solution was obtained. The hexanes solution was concentrated to approximately one-fourth of its original volume, filtered and cooled to –25 °C. [(BDI*)Ca]$_2$(C$_3$(NCy)$_3$)(THP) (**2**) was isolated as brown crystals suitable for X-ray diffraction analysis. The supernatant was decanted and the crystals were washed with cold pentane (–25 °C, 2 x 1 mL) and dried *in vacuo* (crystalline yield: 24.7 mg, 15.1 µmol, 27%). Attempts to increase the yield by cooling the remaining mother liquor again to –25 °C led to co-crystallization of [(BDI*)Ca]$_2$(C$_3$(NCy)$_3$)(THP) (**2**) and (BDI*)$_2$Ca·(CN–Cy) (**3**). A third crop of crystals was found to be pure (BDI*)$_2$Ca·(CN–Cy) (**3**) which could be isolated in a yield of 10% (8.0 mg, 6.2 µmol). An alternative method to prepare **3** is described below.

^{1}H NMR (600.13 MHz, C_6D_6, 298K): δ = 0.96 (m, 24H, C**H$_3$**), 1.00 (m, 24H, C**H$_3$**), 1.17 (m, 6H, THP β,γ-C**H$_2$**), 1.23–1.62 (m, 27H, C**H**/C**H$_2$**-Cy) 1.75 (s, 12H, C**H$_3$**-backbone), 1.76–1.81 (m, 32H, C**H$_2$**), 1.94 (m, 6H, C**H$_2$**-Cy), 3.14 (m, 8H, C**H**), 3.53 (m, 4H, α-C**H$_2$**), 4.87 (s, 2H, C**H**-backbone), 7.08–7.10 (m, 8H, C**H**-arom.), 7.13–7.14 (m, 4H, C**H**-arom.) ppm.

FT-IR (ATR, pure): $\tilde{\nu}$ = 2969 (s), 29621 (s), 2871 (m), 1506 (m), 1429 (s), 1399 (s), 1336 (m), 1166 (m), 1016 (m), 786 (s), 763 (s) cm^{-1}.

Elemental analysis Calculated for $C_{100}H_{157}Ca_2N_7O \cdot C_6H_{14}$ (M = 1639.74 g/mol): C 77.64, H 10.51, N 5.98; Found: C 77.93, H 10.17, N 6.57.

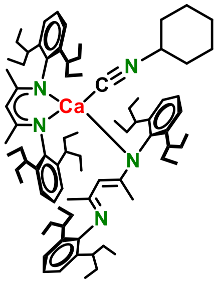

Synthesis of (BDI*)$_2$Ca·(CN–Cy) (3) (BDI*)$_2$Ca (49.5 mg, 45.0 µmol, 1.00 eq.) and Cy–NC (4.4 µL, 3.9 mg, 35.5 µmol, 1.00 eq.) were dissolved in C_6D_6 (500 µL). The solvent was removed under high vacuum and (BDI*)$_2$Ca·(CN–Cy) (**3**) was isolated as an off-white powder (47 mg, 36.3 µmol, 81%). Colorless crystals suitable for X-ray diffraction analysis were grown from a saturated hexanes solution at –25 °C. The supernatant was decanted and the crystals were washed with cold pentane (–25 °C, 2 x 1 mL) and dried *in vacuo*.

^{1}H NMR (600.13 MHz, C_6D_6, 298K): δ = 0.83 (m, 6H, C**H$_3$**), 0.89 (m, 24H, C**H$_3$**), 0.98 0.96 (m, 18H, C**H$_3$**), 1.23–1.45 (m, 10H, C**H$_2$**-Cy), 1.45 (s, 3H, C**H$_3$**-backbone), 1.57–1.90 (m, 32H, C**H$_2$**), 2.56 (s, 3H, C**H$_3$**-backbone), 2.75–2.81 (m, 1H, C**H**-Cy), 2.75–3.15 (m, 8H, C**H**), 3.86 (s, 1H, C**H**-backbone), 4.77 (s, 1H, C**H**-backbone), 7.03–7.08 (m, 4H, C**H**-arom.), 7.09–7.14 (m, 8H, C**H**-arom.) ppm.

^{13}C{^{1}H} NMR (150.92 MHz, C_6D_6, 298K): δ = 11.6 (**C**H$_3$), 12.4 (**C**H$_3$), 13.3 (**C**H$_3$), 14.4 (**C**H$_3$), 22.4 (**C**H$_2$-Cy), 23.1 (**C**H$_3$-backbone), 23.2 (**C**H$_2$-Cy), 24.1 (**C**H$_3$-backbone), 24.8 (**C**H$_2$), 25.8 (**C**H$_3$-backbone), 26.2 (**C**H$_2$-Cy), 27.2 (**C**H$_2$), 29.4 (**C**H$_2$), 30.4 (**C**H$_2$), 31.8 (**C**H$_2$), 35.0 (**C**H$_2$), 39.5 (**C**H), 40.6 (**C**H), 42.2 (**C**H-Cy), 52.8 (**C**H), 93.1 (**C**H-backbone), 95.4 (**C**H-backbone), 120.9 (**C**-arom.), 121.9 (**C**-arom.), 123.5 (**C**-arom.), 123.9 (**C**-arom.), 124.2 (**C**-arom.), 126.0 (**C**-arom.), 134.9 (**C**-arom.), 150.2 (**C**-arom.), 153.9 (**C**-arom.), 161.0 (**C**N-backbone), 162.4 (**C**N-backbone), 166.7 (**C**N-backbone) ppm.

Elemental analysis Calculated for $C_{81}H_{125}CaN_5 \cdot C_6H_{14}$ (M = 1295.18 g/mol): C 80.68, H 10.82, N 5.41; Found: C 80.42, H 10.39, N 5.72.

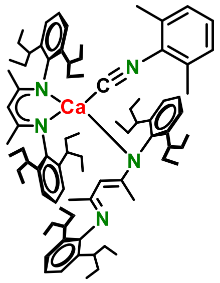

Synthesis of (BDI*)$_2$Ca·(CN–Xyl) (4) (BDI*)$_2$Ca (42 mg, 38.4 µmol, 1.00 eq.) and Xyl–NC (5 mg, 38.4 µmol, 1.00 eq.) were dissolved in C_6D_6 (500 µL). The solvent was removed under high vacuum and (BDI*)$_2$Ca·(CN–Xyl) (**4**) was isolated as an off-white powder (40 mg, 32.5 µmol, 85%). Colorless crystals suitable for X-ray diffraction analysis were grown from a saturated hexanes solution at –25 °C. The supernatant was decanted and the crystals were washed with cold pentane (–25 °C, 2 x 1 mL) and dried *in vacuo*.

^{1}H NMR (600.13 MHz, C_6D_6, 298K): δ = 0.81–0.86 (m, 24H, C**H$_3$**), 0.89–0.92 (m, 12H, C**H$_3$**), 0.95–0.98 (m, 12H, C**H$_3$**), 1.44 (s, 3H, C**H$_3$**-backbone), 1.57 (s, 6H, C**H$_3$**-backbone) 1.59–1.83 (m, 32H, C**H$_2$**), 2.28 (s, 6H, C**H$_3$**-Xyl), 2.60 (s, 3H, C**H$_3$**-backbone), 2.75 (m, 2H, C**H**), 2.90 (m, 2H, C**H**), 3.00 (m, 2H, C**H**), 3.87 (s, 1H, C**H**-backbone), 4.76 (s, 1H, C**H**-backbone), 6.61 (d, *J* = 7.7 Hz, 2H, C**H**-Xyl), 6.76 (t, *J* = 7.7 Hz, 1H, C**H**-Xyl), 6.99–7.01 (m, 4H, C**H**-arom.), 7.06–7.10 (m, 8H, C**H**-arom.) ppm.

^{13}C{^{1}H} NMR (150.92 MHz, C_6D_6, 298K): δ = 11.7 (**C**H$_3$), 11.9 (**C**H$_3$), 12.2 (**C**H$_3$), 12.8 (**C**H$_3$), 12.9 (**C**H$_3$), 13.2 (**C**H$_3$), 19.0 (**C**H$_3$-Xyl), 23.0 (**C**H$_3$-backbone), 23.9 (**C**H$_3$-backbone), 25.7 (**C**H$_3$-backbone), 27.9 (**C**H$_2$), 28.1 (**C**H$_2$), 28.4 (**C**H$_2$), 29.4 (**C**H$_2$), 29.9 (**C**H$_2$), 30.6 (**C**H$_2$), 40.3 (**C**H), 41.0 (**C**H), 42.2 (**C**H), 93.2 (**C**H-backbone), 96.5 (**C**H-backbone), 121.2 (**C**-arom.), 123.2 (**C**-arom.), 123.5 (**C**-arom.), 124.3 (**C**-Xyl), 125.2 (**C**-arom.), 126.1 (**C**-arom.), 130.4 (**C**-Xyl), 133.5 (**C**-Xyl), 134.8 (**C**-arom.), 135.9 (**C**-Xyl), 138.2 (**C**-arom.), 139.7 (**C**-

arom.), 140.4 (**C**-arom.), 148.6 (**C**-arom.), 149.9 (**C**-arom.), 153.4 (**C**-arom.), 159.1 (**C**N-backbone), 162.6 (**C**N-backbone), 167.9 (**C**N-backbone) ppm.

Elemental analysis Calculated for $C_{83}H_{123}CaN_5$ (M = 1231.01 g/mol): C 80.98, H 10.07, N 5.69; Found: C 81.20, H 10.25, N 5.85.

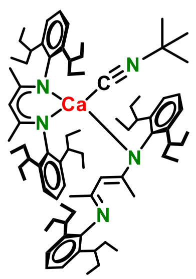

Synthesis of $(BDI^*)_2Ca\cdot(CN\text{–}t\text{Bu})$ (5) $(BDI^*)_2Ca$ (42 mg, 38.4 µmol, 1.00 eq.) and *t*Bu–NC (3.4 µL, 3.2 mg, 38.4 µmol, 1.00 eq.) were dissolved in C_6D_6 (500 µL). The solvent was removed under high vacuum and $(BDI^*)_2Ca\cdot(CN\text{–}t\text{Bu})$ (**5**) was isolated as an off-white powder (43 mg, 35.1 µmol, 91%). Colorless crystals suitable for X-ray diffraction analysis were grown from a saturated hexanes solution at –25 °C. The supernatant was decanted and the crystals were washed with cold pentane (–25 °C, 2 x 1 mL) and dried *in vacuo*.

^{1}H NMR (600.13 MHz, C_6D_6, 298K): δ = 0.85–0.87 (m, 24H, C$\mathbf{H_3}$), 0.83–0.97 (m, 24H, C$\mathbf{H_3}$), 1.00 (s, 9H, C$\mathbf{H_3}$-^{t}Bu), 1.42 (s, 3H, C$\mathbf{H_3}$-backbone), 1.58 (s, 6H, C$\mathbf{H_3}$-backbone) 1.61–1.80 (m, 32H, C$\mathbf{H_2}$), 2.51 (s, 3H, C$\mathbf{H_3}$), 2.70–2.76 (m, 2H, C**H**), 2.76–2.96 (m, 6H, C**H**), 3.84 (s, 1H, C**H**-backbone), 4.71 (s, 1H, C**H**-backbone), 6.98–7.00 (m, 4H, C**H**-arom.), 7.04–7.10 (m, 8H, C**H**-arom.) ppm.

^{13}C{^{1}H} NMR (150.92 MHz, C_6D_6, 298K): δ = 11.9 (**C**H_3), 12.0 (**C**H_3), 12.2 (**C**H_3), 12.3 (**C**H_3), 13.3 (**C**H_3), 23.0 (**C**H_3-backbone), 24.0 (**C**H_3-backbone), 25.8 (**C**H_3-backbone), 27.0 (**C**H_2), 27.4 (**C**H_2), 27.9 (**C**H_2), 28.4 (**C**H_2), 29.6 (**C**H_3-^{t}Bu), 30.5 (**C**H_2), 39.8 (**C**H), 40.8 (**C**H), 42.2 (**C**H), 56.0 (**C**Me_3-^{t}Bu), 93.1 (**C**H-backbone), 95.8 (**C**H-backbone), 121.0 (**C**-arom.), 122.4 (**C**-arom.), 123.4 (**C**-arom.), 123.9 (**C**-arom.), 126.0 (**C**-arom.), 134.9 (**C**-arom.),138.0 (**C**-arom.), 139.6 (**C**-arom.), 143.5 (**C**-arom.), 149.4 (**C**-arom.), 150.0 (**C**-arom.), 153.7 (**C**-arom.), 160.4 (**C**N-backbone), 162.4 (**C**N-backbone), 167.1 (**C**N-backbone) ppm.

Elemental analysis Calculated for $C_{79}H_{123}CaN_5 \cdot 0.5\ C_6H_{14}$ (M = 1226.06 g/mol): C 80.33, H 10.69, N 5.71; Found: C 81.00, H 10.61, N 5.50.

3. Spectroscopic Characterization

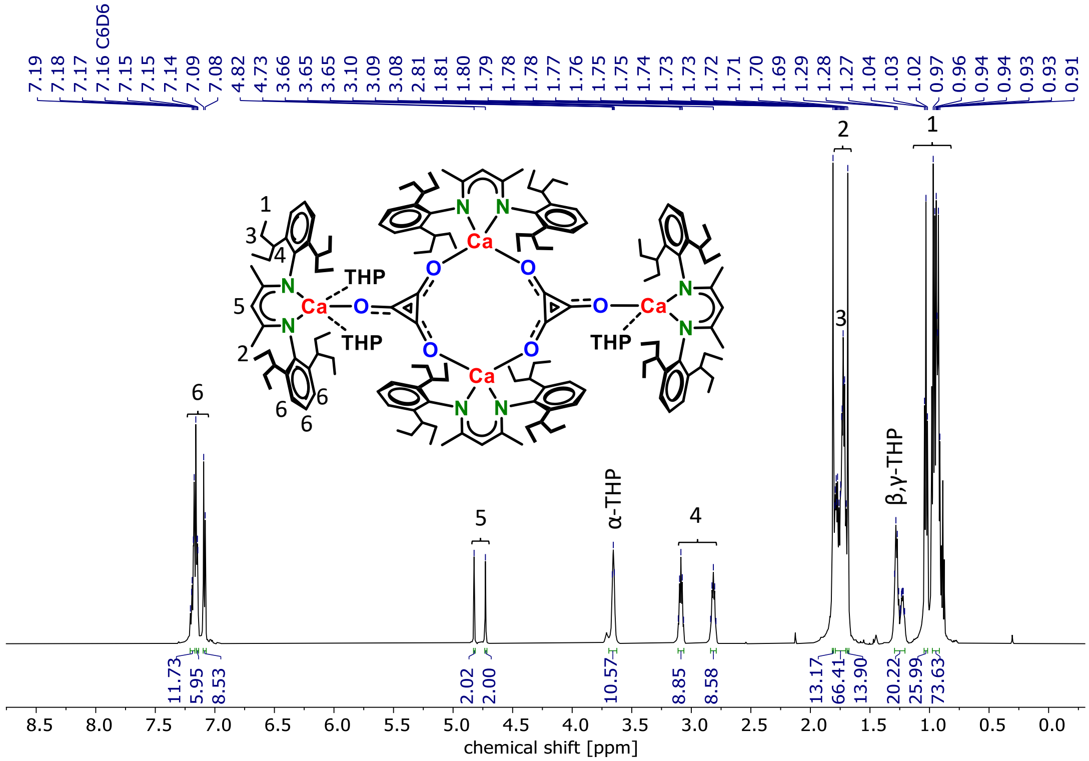

Figure S1. ^{1}H NMR spectrum (600.13 MHz, C_6D_6, 298K) of $[(BDI^*)Ca]_4(THP)_3(C_3O_3)_2$ (**1**).

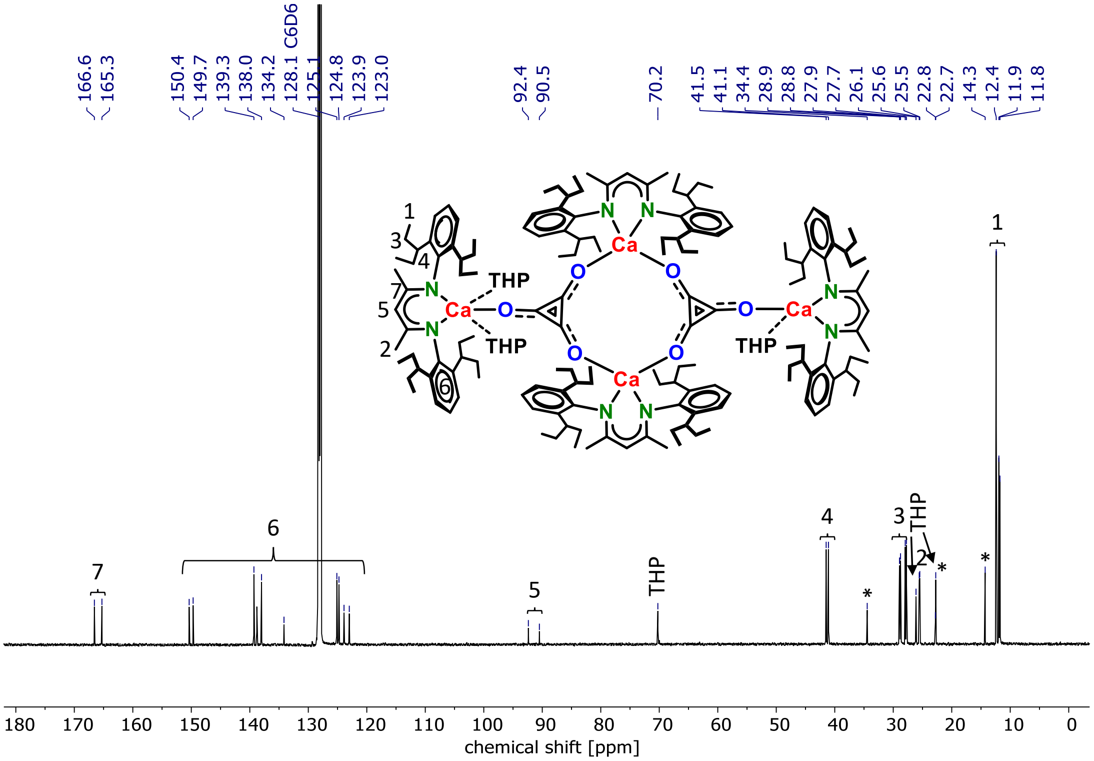

Figure S2. $^{13}C\{^{1}H\}$ NMR spectrum (150.92 MHz, C_6D_6, 298K) of $[(BDI^*)Ca]_4(THP)_3(C_3O_3)_2$ (**1**). Co-crystallized pentane is marked with asterisks.

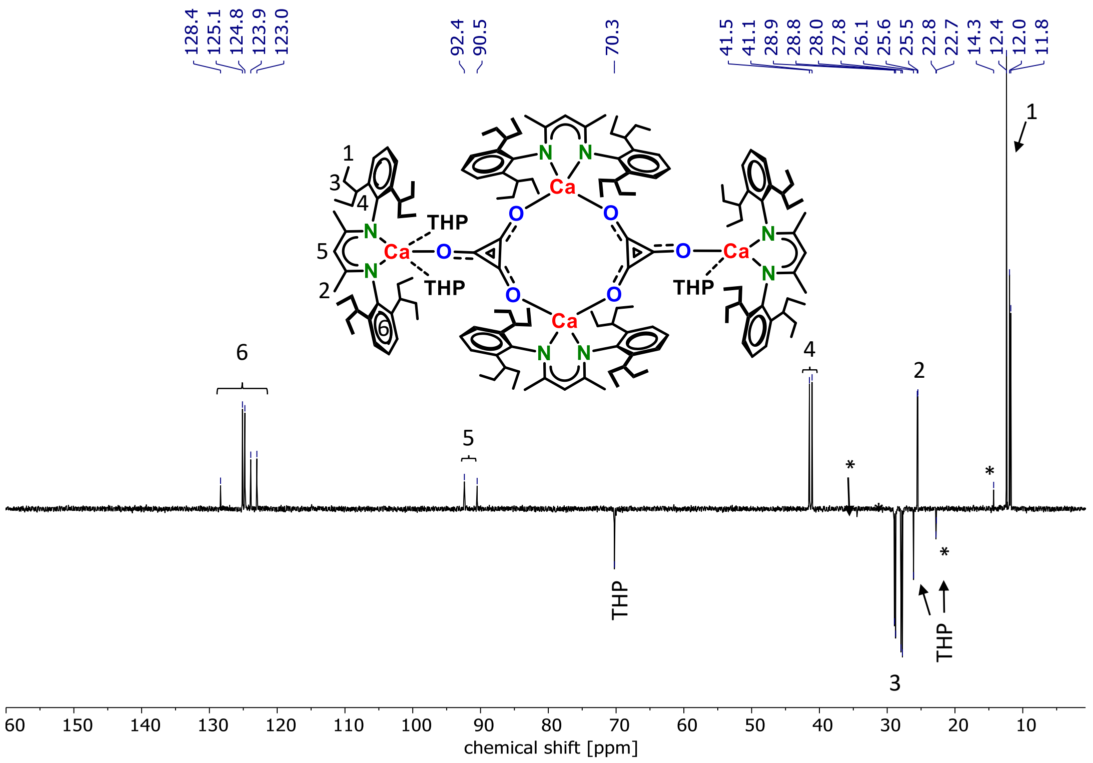

Figure S3. ^{13}C(DEPT 135) NMR spectrum (150.92 MHz, C_6D_6, 298K) of $[(BDI^*)Ca]_4(THP)_3(C_3O_3)_2$ (**1**).

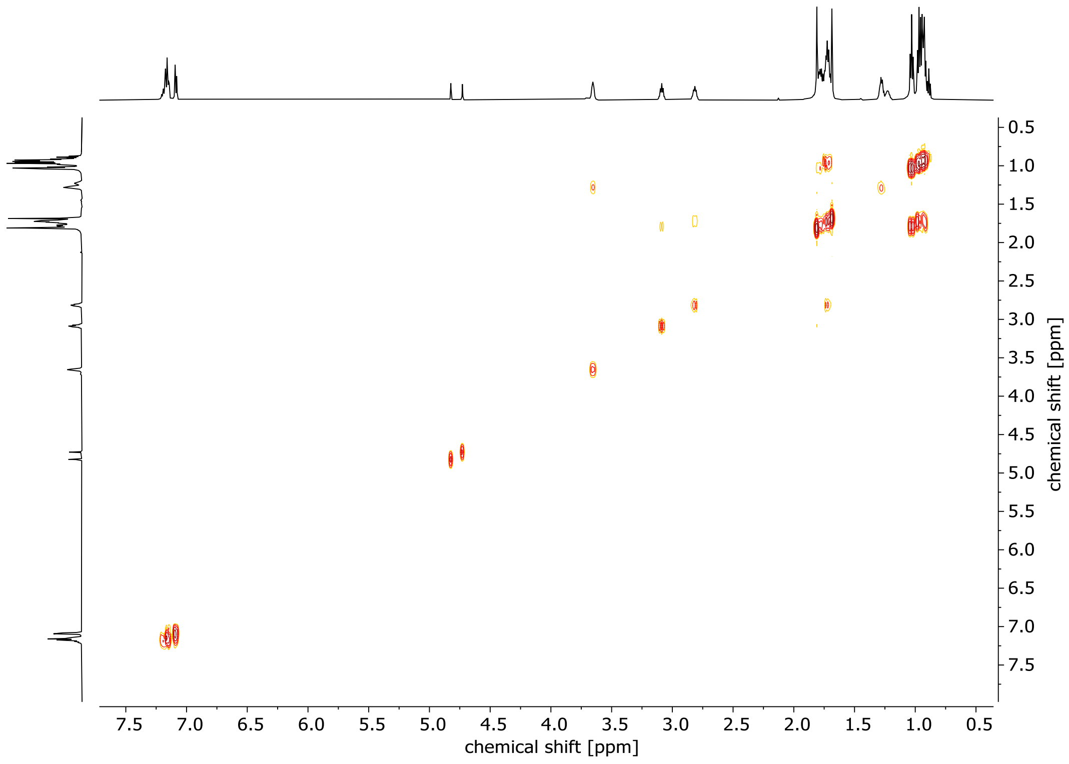

Figure S4. ^{1}H-^{1}H COSY NMR spectrum (600.13 MHz, C_6D_6, 298K) of $[(BDI^*)Ca]_4(THP)_3(C_3O_3)_2$ (**1**).

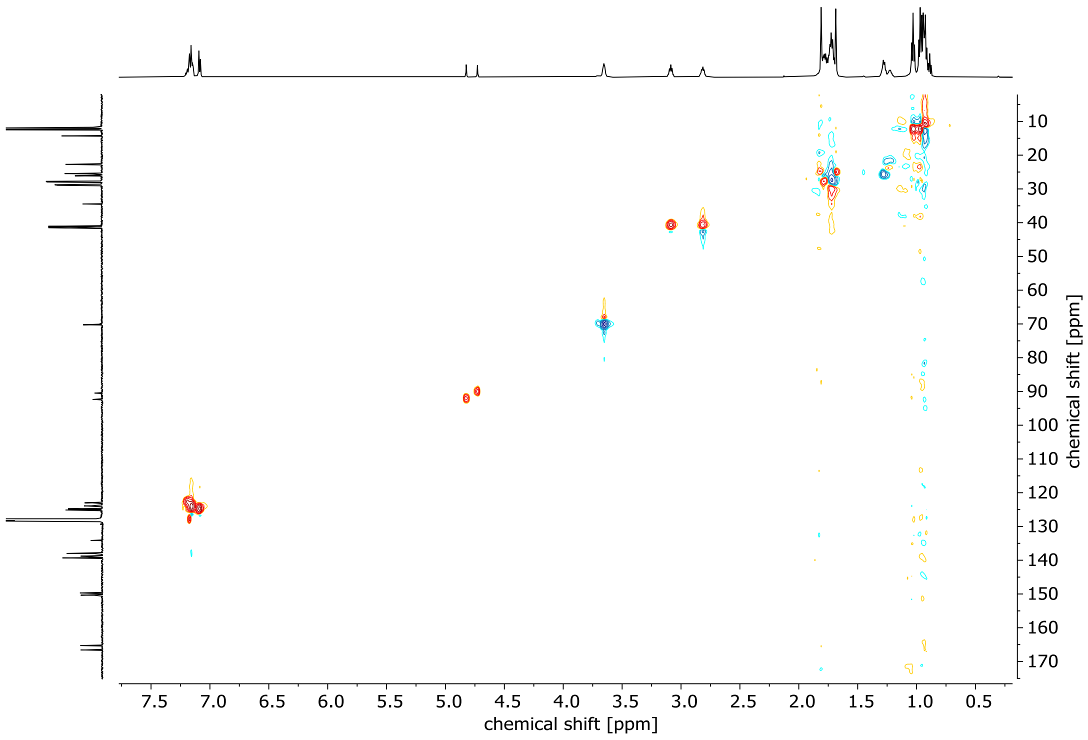

Figure S5. ^{1}H-^{13}C HSQC NMR spectrum (600.13/150.91 MHz, C_6D_6, 298K) of $[(BDI^*)Ca]_4(THP)_3(C_3O_3)_2$ (**1**).

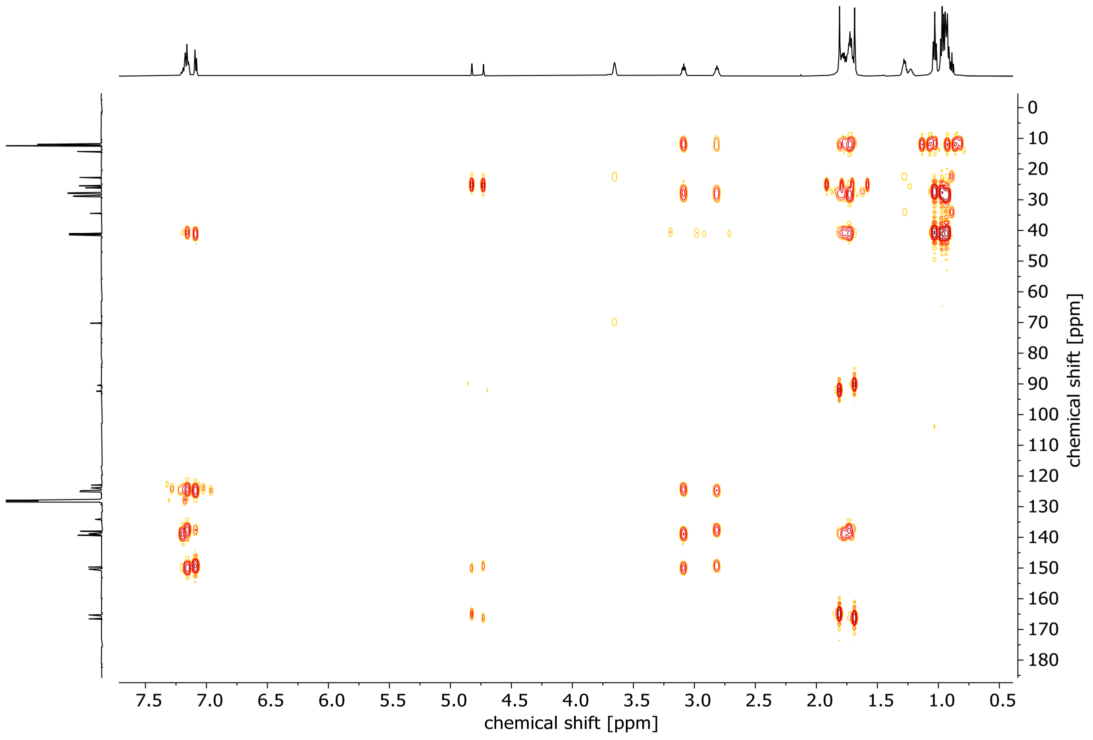

Figure S6. ^{1}H-^{13}C HMBC NMR spectrum (600.13/150.91 MHz, C_6D_6, 298K) of [(BDI*)Ca]$_4$(THP)$_3$(C_3O_3)$_2$ (**1**).

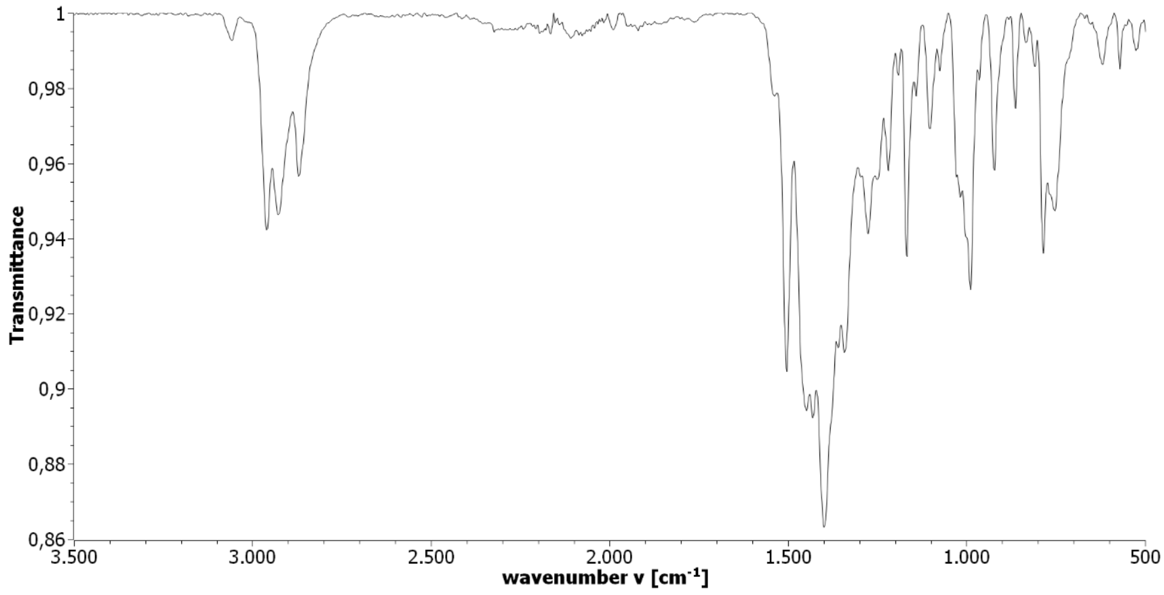

Figure S7. FT-IR ATR spectrum of [(BDI*)Ca]$_4$(THP)$_3$(C_3O_3)$_2$ (**1**).

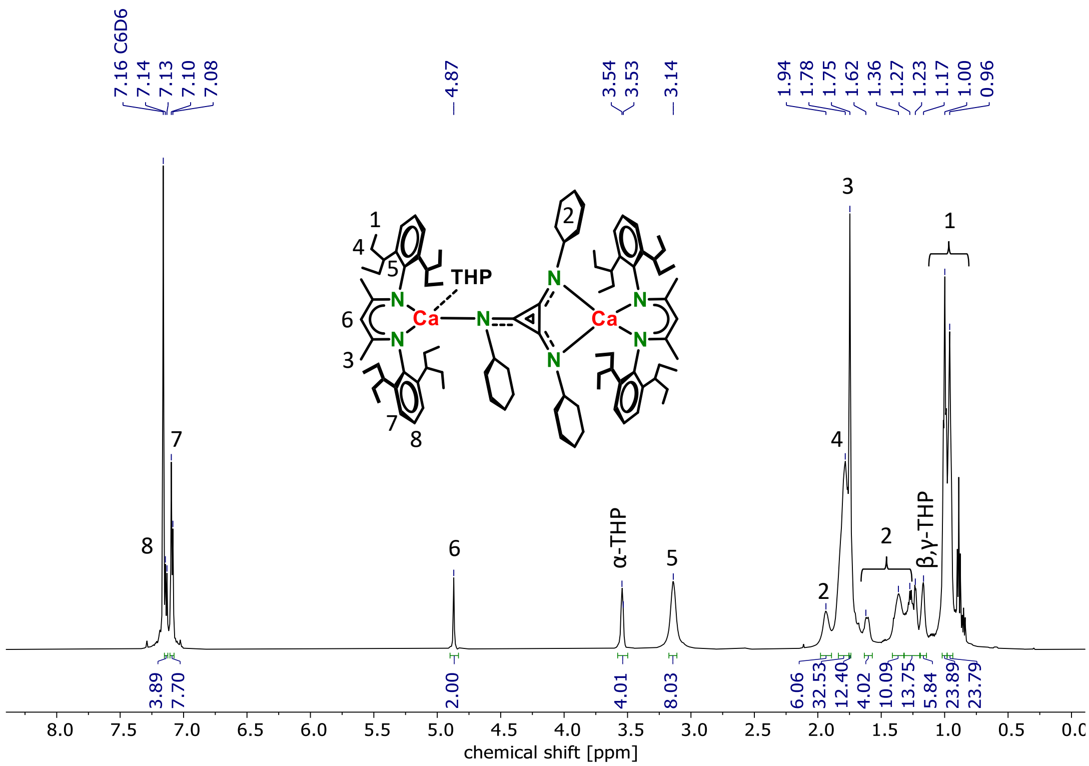

Figure S8. ^{1}H NMR spectrum (600.13 MHz, C_6D_6, 298K) of [(BDI*)Ca]$_2$(C_3(NCy)$_3$)(THP) (**2**).

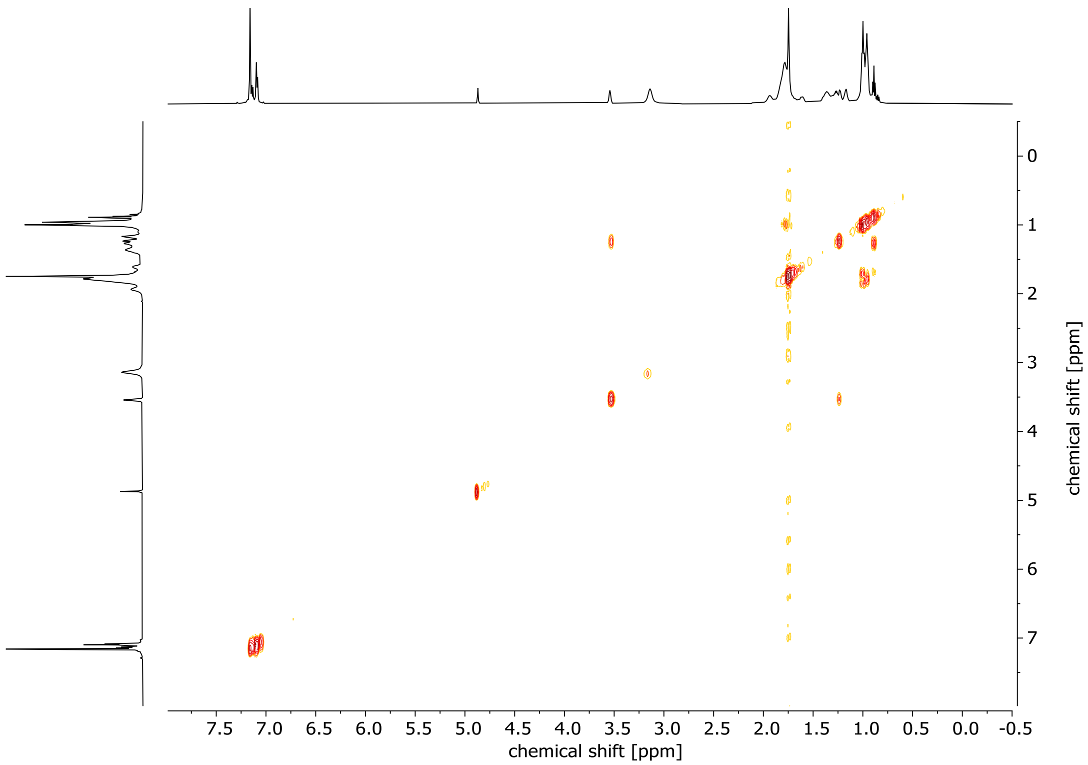

Figure S9. ^{1}H-^{1}H COSY NMR spectrum (600.13 MHz, C_6D_6, 298K) of [(BDI*)Ca]$_2$(C_3(NCy)$_3$)(THP) (**2**).

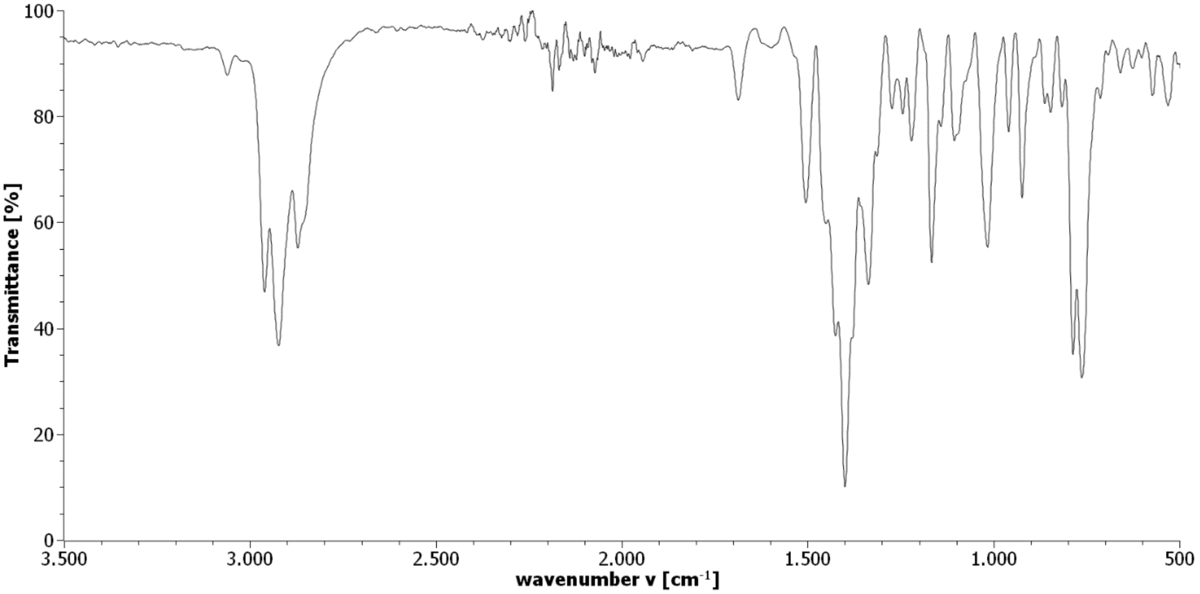

Figure S10. FT-IR ATR spectrum of [(BDI*)Ca]$_2$(C$_3$(NCy)$_3$)(THP) (**2**).

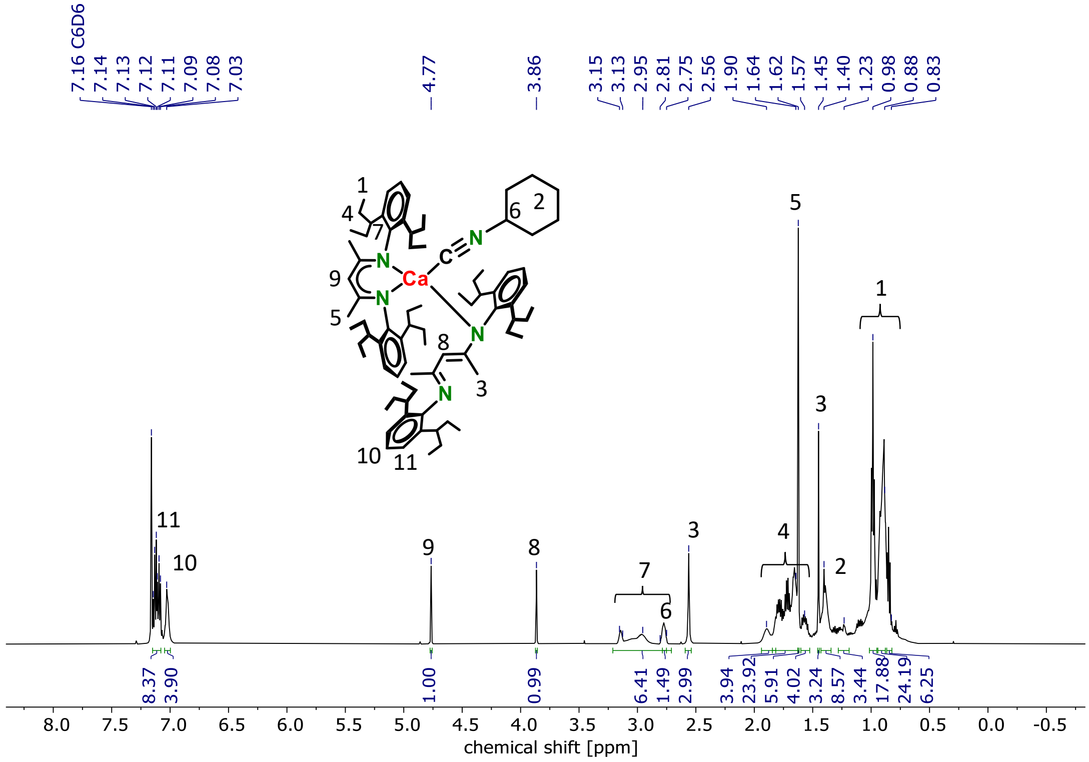

Figure S11. ^{1}H NMR spectrum (600.13 MHz, C_6D_6, 298K) of (BDI*)$_2$Ca·(CN–Cy) (**3**).

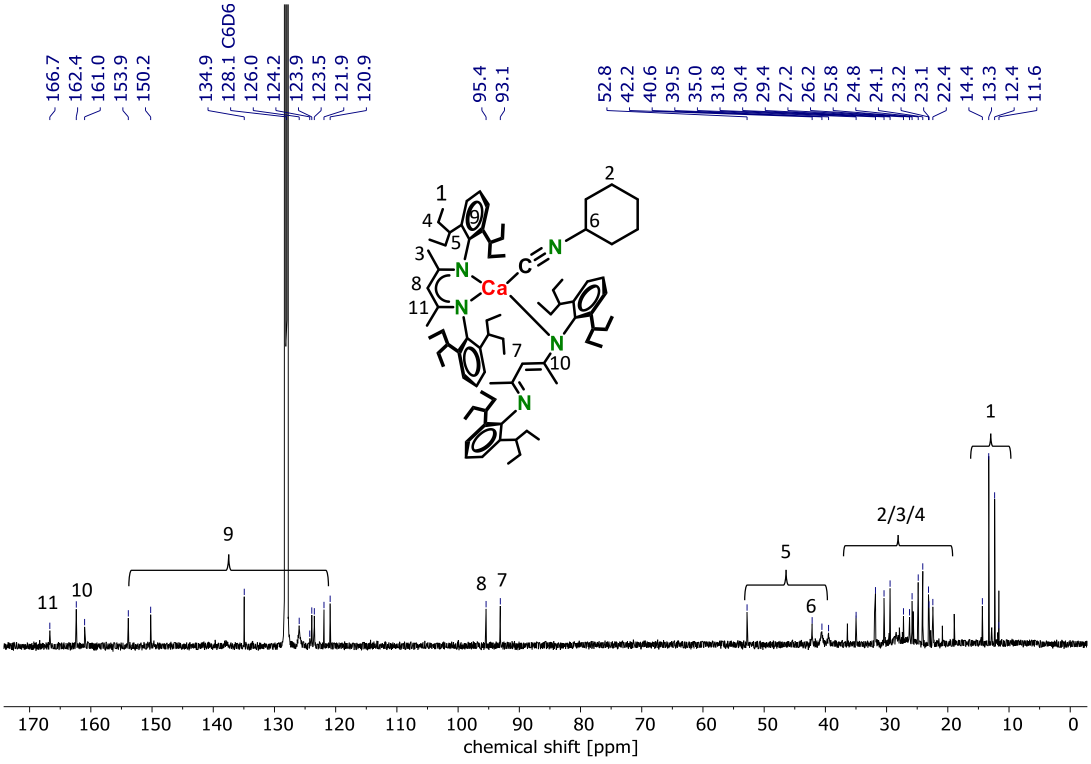

Figure S12. $^{13}C\{^{1}H\}$ NMR spectrum (150.92 MHz, C_6D_6, 298K) of $(BDI^*)_2Ca\cdot(CN–Cy)$ (**3**).

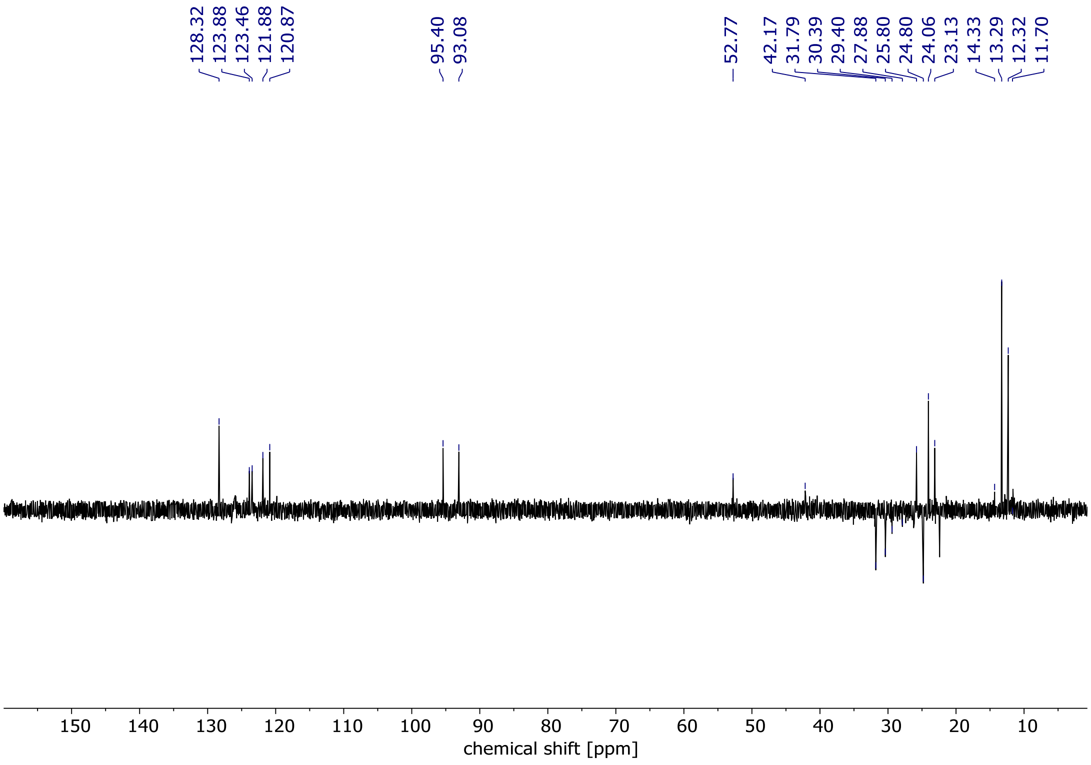

Figure S13. ^{13}C(DEPT 135) NMR spectrum (150.92 MHz, C_6D_6, 298K) of $(BDI^*)_2Ca\cdot(CN–Cy)$ (**3**).

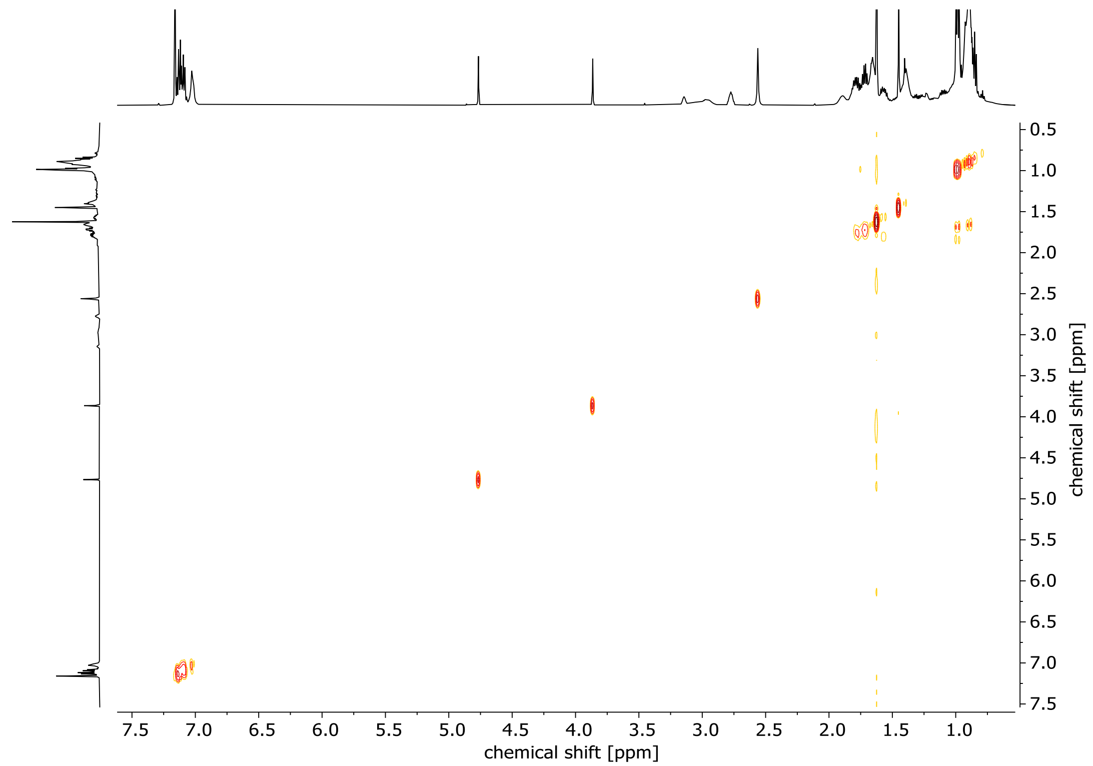

Figure S14. ^{1}H-^{1}H COSY NMR spectrum (600.13 MHz, C_6D_6, 298K) of (BDI*)$_2$Ca·(CN–Cy) (**3**).

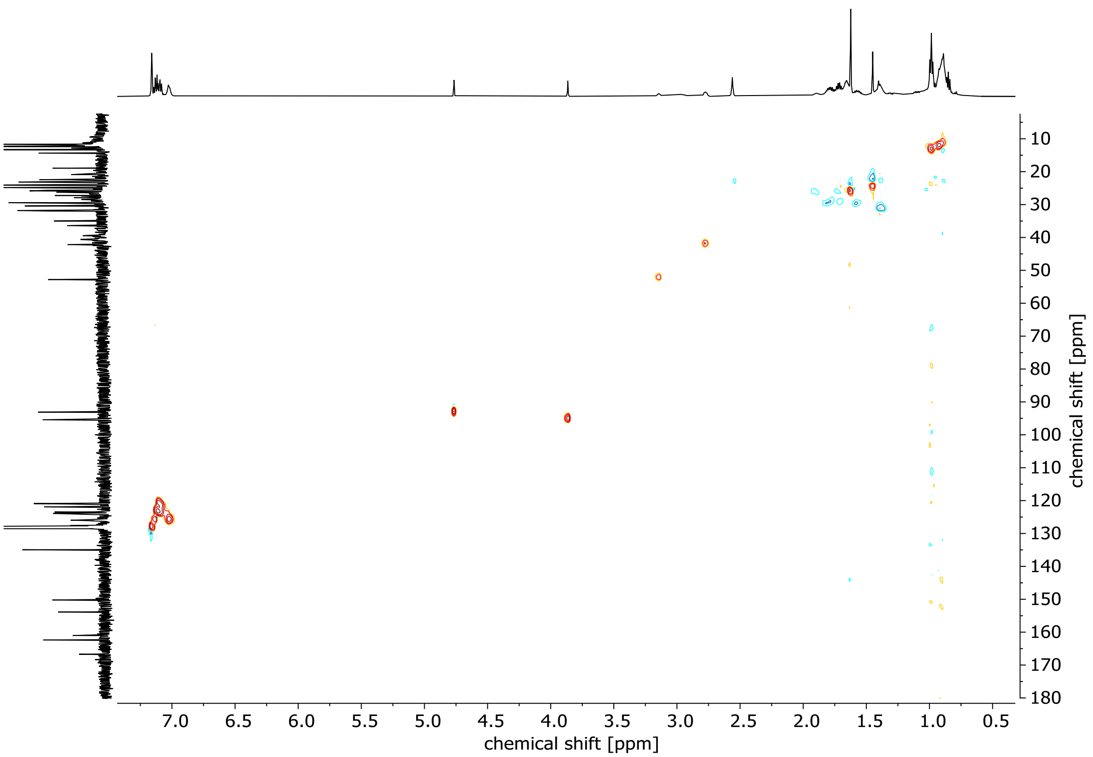

Figure S15. ^{1}H-^{13}C HSQC NMR spectrum (600.13/150.91 MHz, C_6D_6, 298K) of (BDI*)$_2$Ca·(CN–Cy) (**3**).

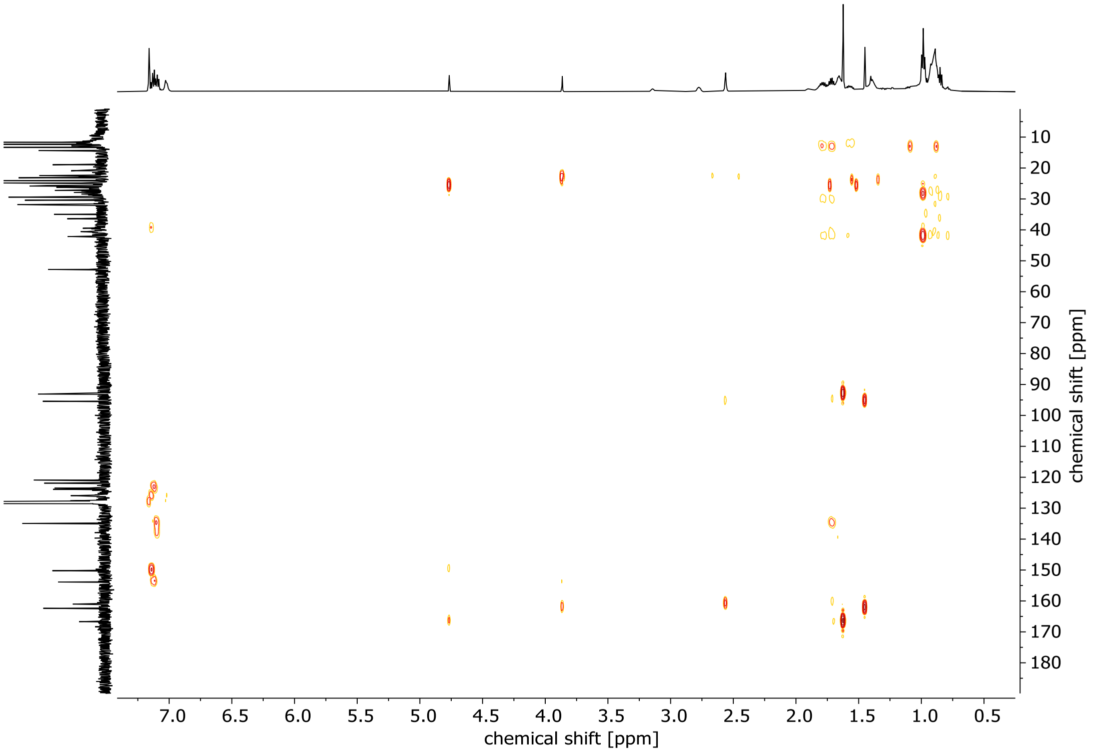

Figure S16. ^{1}H-^{13}C HMBC NMR spectrum (600.13/150.91 MHz, C_6D_6, 298K) of $(BDI^*)_2Ca\cdot(CN–Cy)$ (**3**).

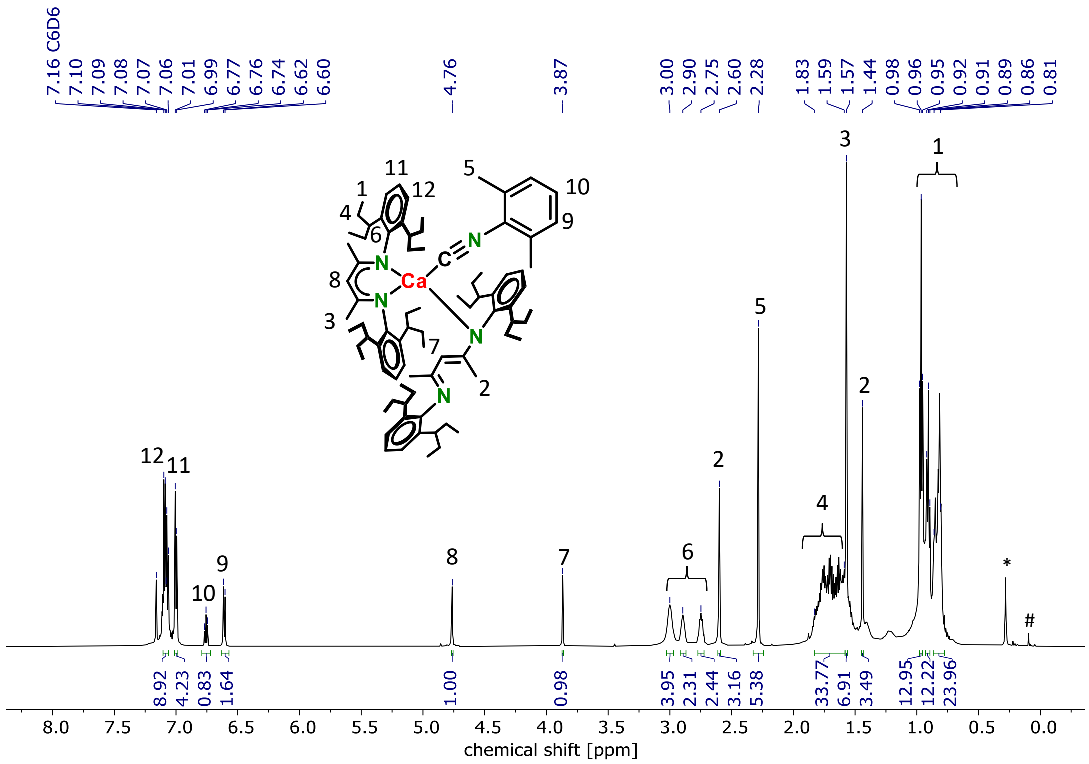

Figure S17. ^{1}H NMR spectrum (600.13 MHz, C_6D_6, 298K) of $(BDI^*)_2Ca\cdot(CN–Xyl)$ (**4**). Residual signals of silicon grease (*) and $HN(SiMe_3)_2$ (#) are marked.

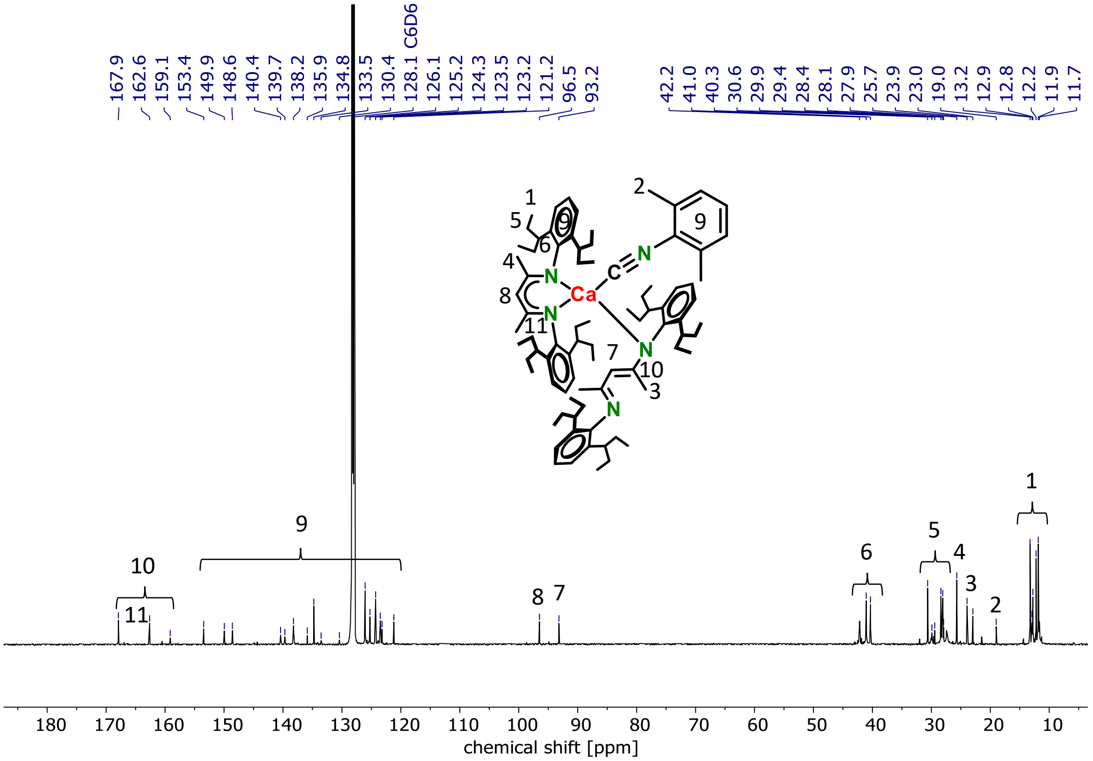

Figure S18. $^{13}C\{^1H\}$ NMR spectrum (150.92 MHz, C_6D_6, 298K) of $(BDI^*)_2Ca{\cdot}(CN–Xyl)$ (**4**).

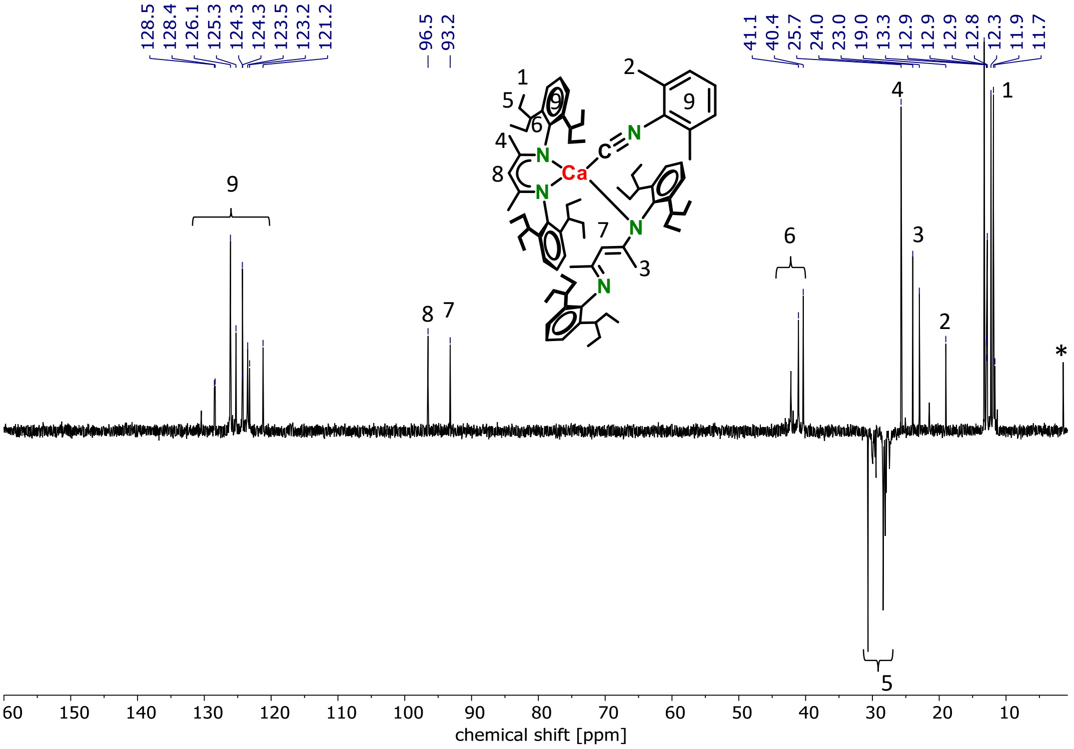

Figure S19. ^{13}C(DEPT 135) NMR spectrum (150.92 MHz, C_6D_6, 298K) of $(BDI^*)_2Ca{\cdot}(CN–Xyl)$ (**4**). Residual signal of silicon grease (*) is marked.

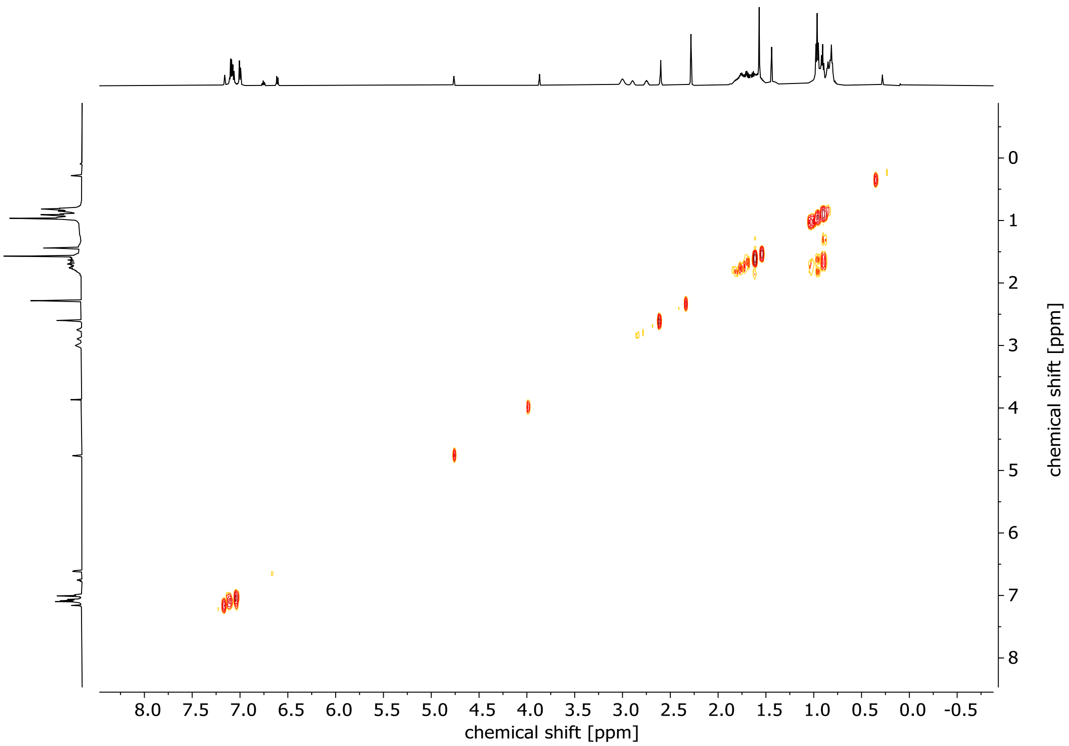

Figure S20. ^{1}H-^{1}H COSY NMR spectrum (600.13 MHz, C_6D_6, 298K) of $(BDI^*)_2Ca\cdot(CN–Xyl)$ (**4**).

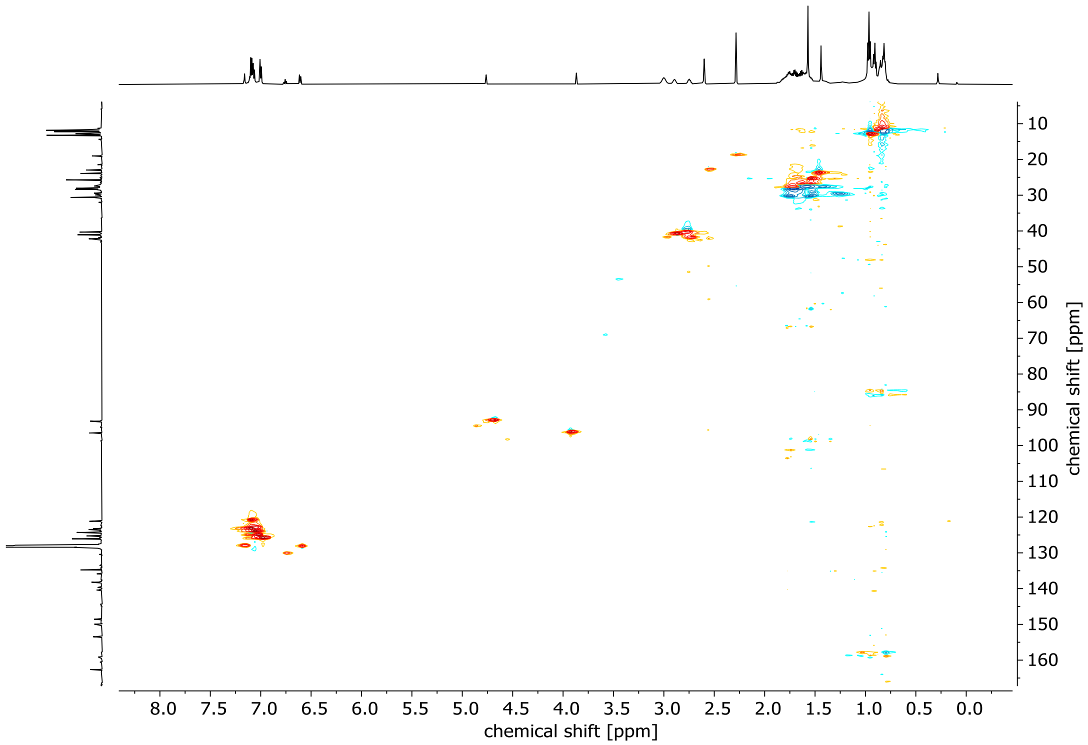

Figure S21. ^{1}H-^{13}C HSQC NMR spectrum (600.13/150.91 MHz, C_6D_6, 298K) of $(BDI^*)_2Ca\cdot(CN–Xyl)$ (**4**).

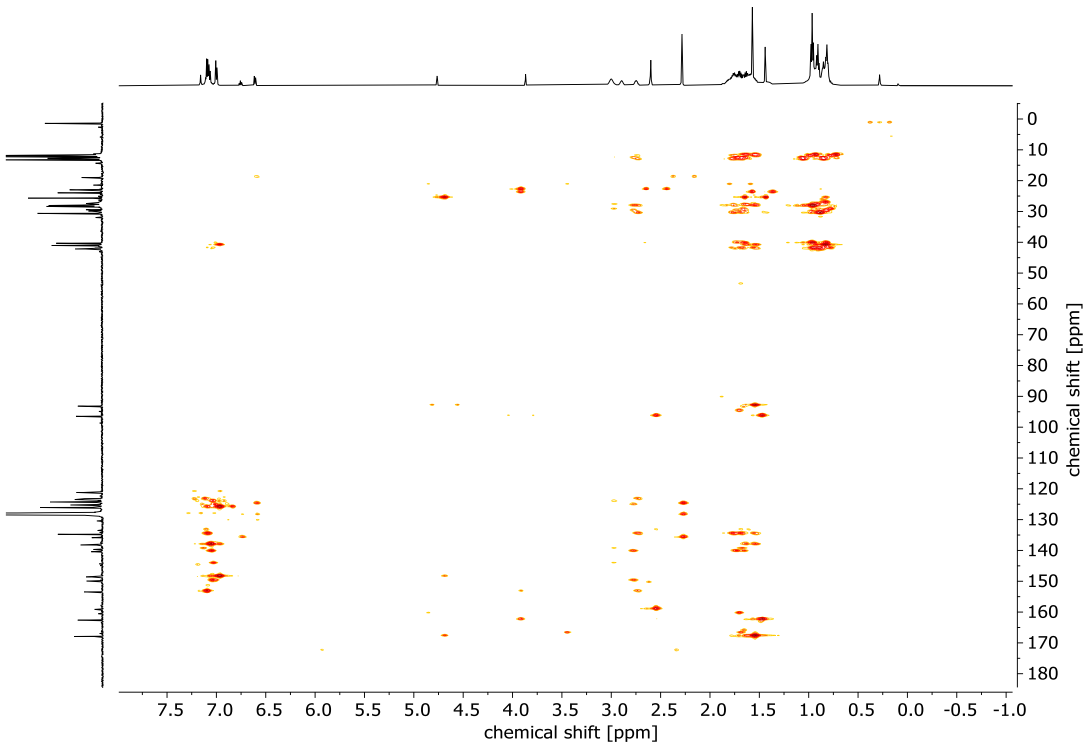

Figure S22. ^{1}H-^{13}C HMBC NMR spectrum (600.13/150.91 MHz, C_6D_6, 298K) of $(BDI^*)_2Ca\cdot(CN\text{–}Xyl)$ (**4**).

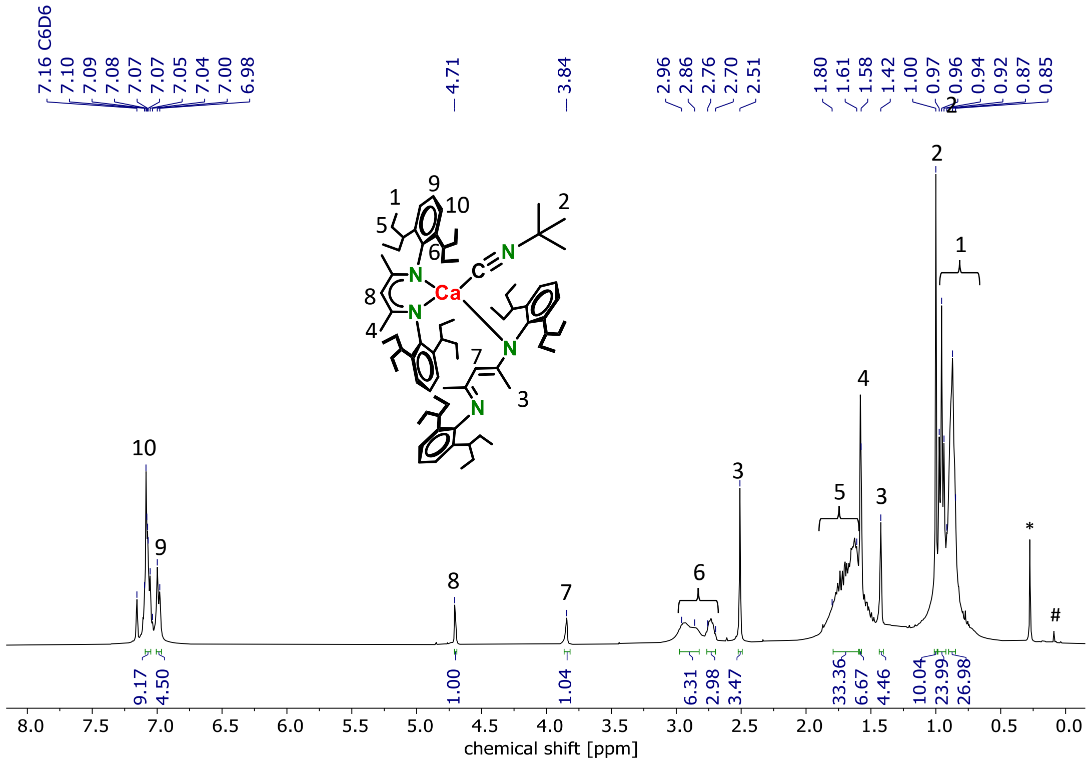

Figure S23. ^{1}H NMR spectrum (400.13 MHz, C_6D_6, 298K) of $(BDI^*)_2Ca\cdot(CN\text{–}t\text{Bu})$ (**5**). Residual signals of silicon grease (*) and $HN(SiMe_3)_2$ (#) are marked.

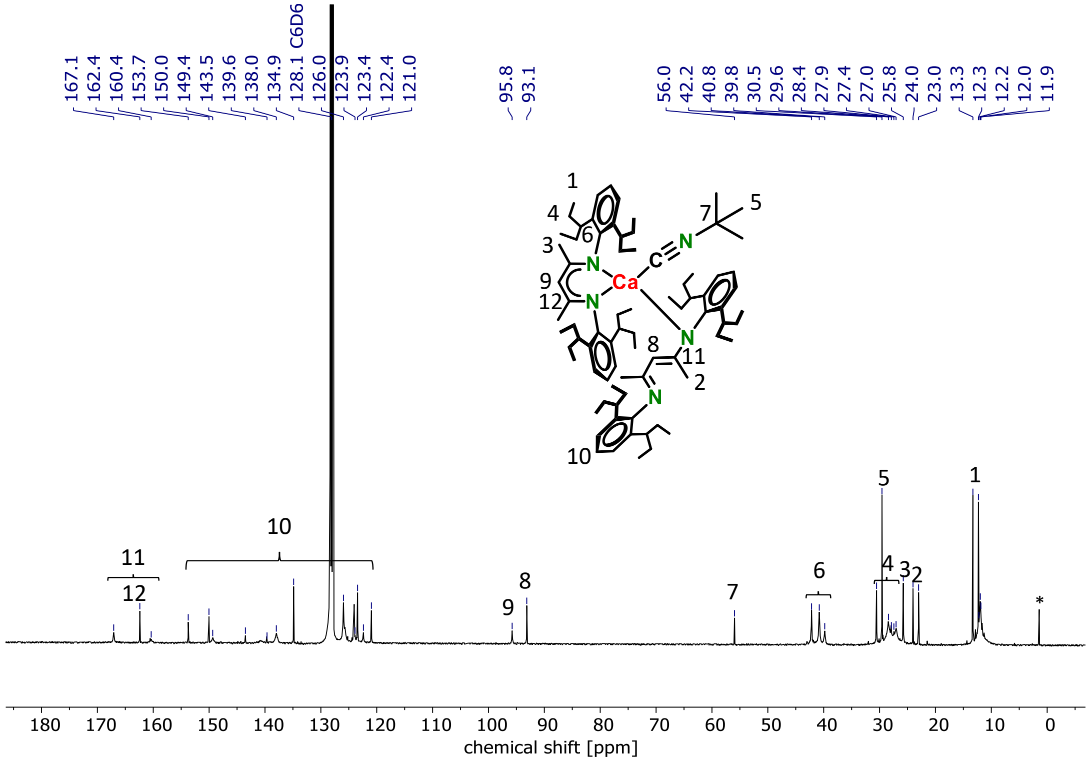

Figure S24. $^{13}C\{^1H\}$ NMR spectrum (100.62 MHz, C_6D_6, 298K) of $(BDI^*)_2Ca{\cdot}(CN{-}t\text{Bu})$ (**5**). Residual signal of silicon grease (*) is marked.

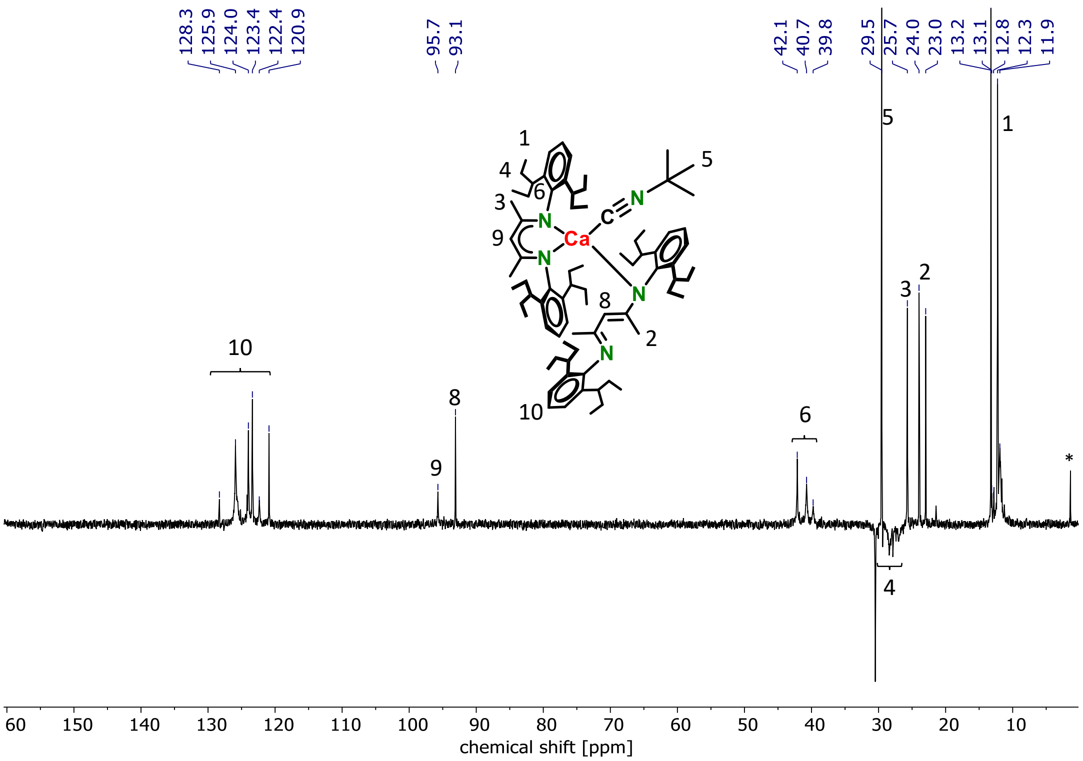

Figure S25. ^{13}C(DEPT 135) NMR spectrum (100.62 MHz, C_6D_6, 298K) of $(BDI^*)_2Ca{\cdot}(CN{-}t\text{Bu})$ (**5**).). Residual signal of silicon grease (*) is marked.

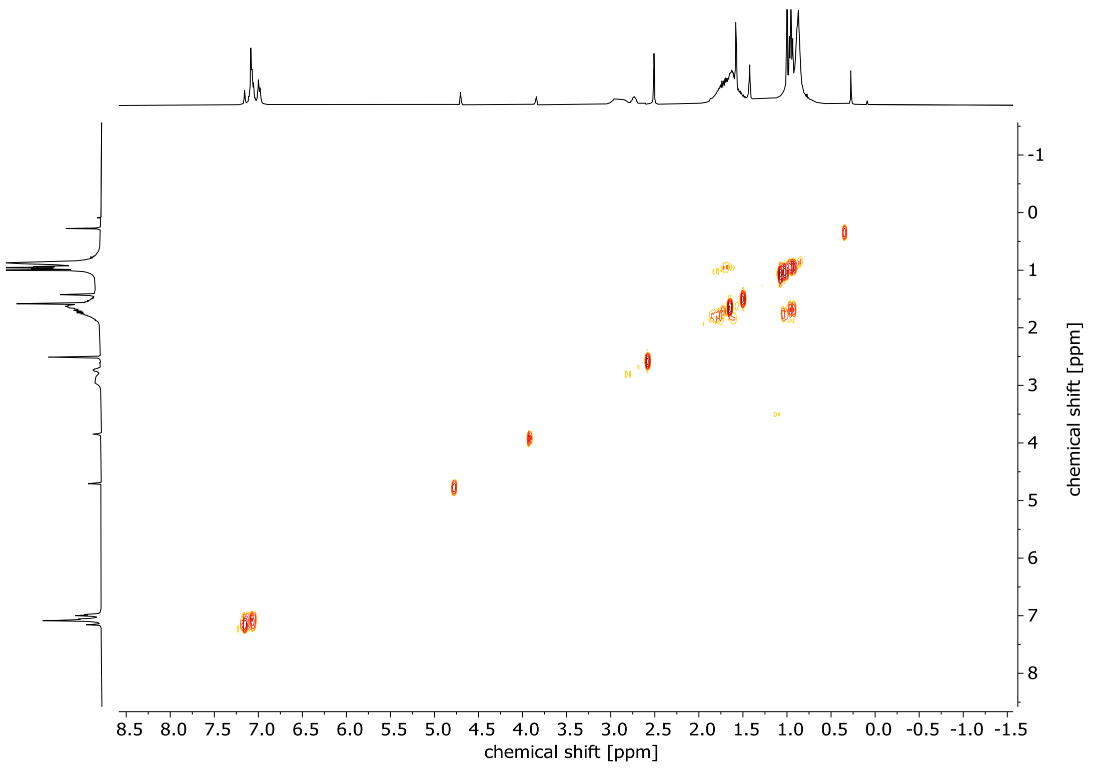

Figure S26. ^{1}H-^{1}H COSY NMR spectrum (400.13 MHz, C_6D_6, 298K) of $(BDI^*)_2Ca\cdot(CN{-}t\mathrm{Bu})$ (**5**).

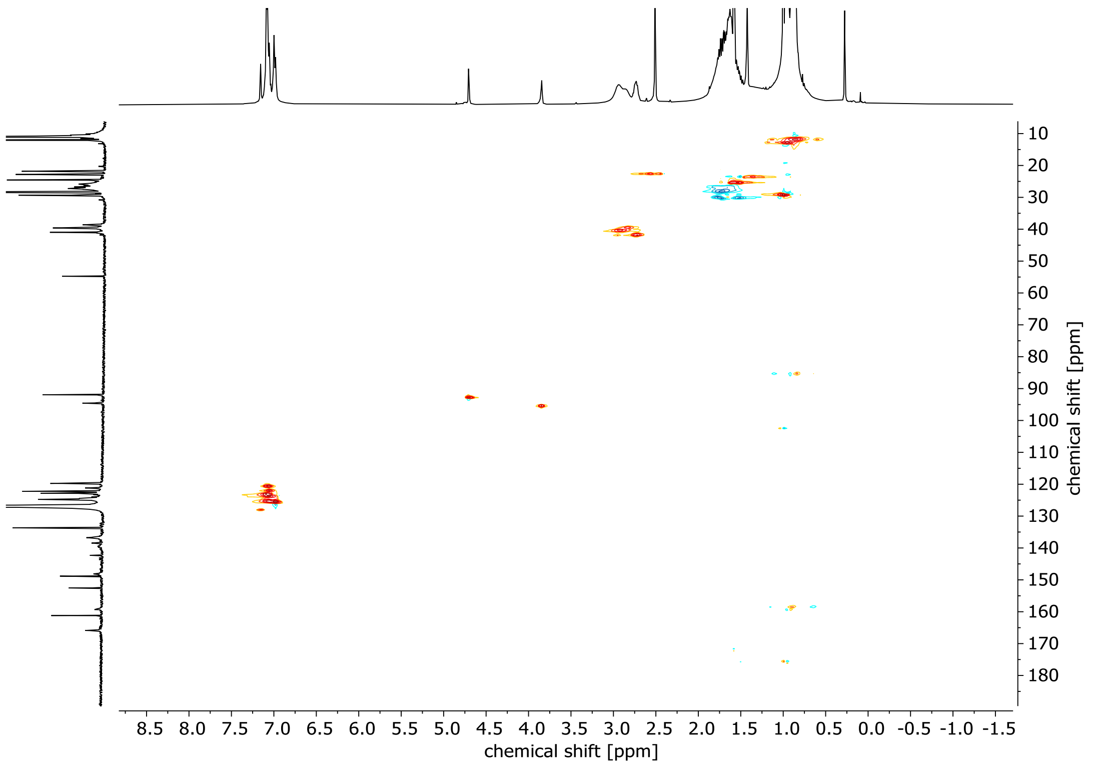

Figure S27. ^{1}H-^{13}C HSQC NMR spectrum (400.13/100.62 MHz, C_6D_6, 298K) of $(BDI^*)_2Ca\cdot(CN{-}t\mathrm{Bu})$ (**5**).

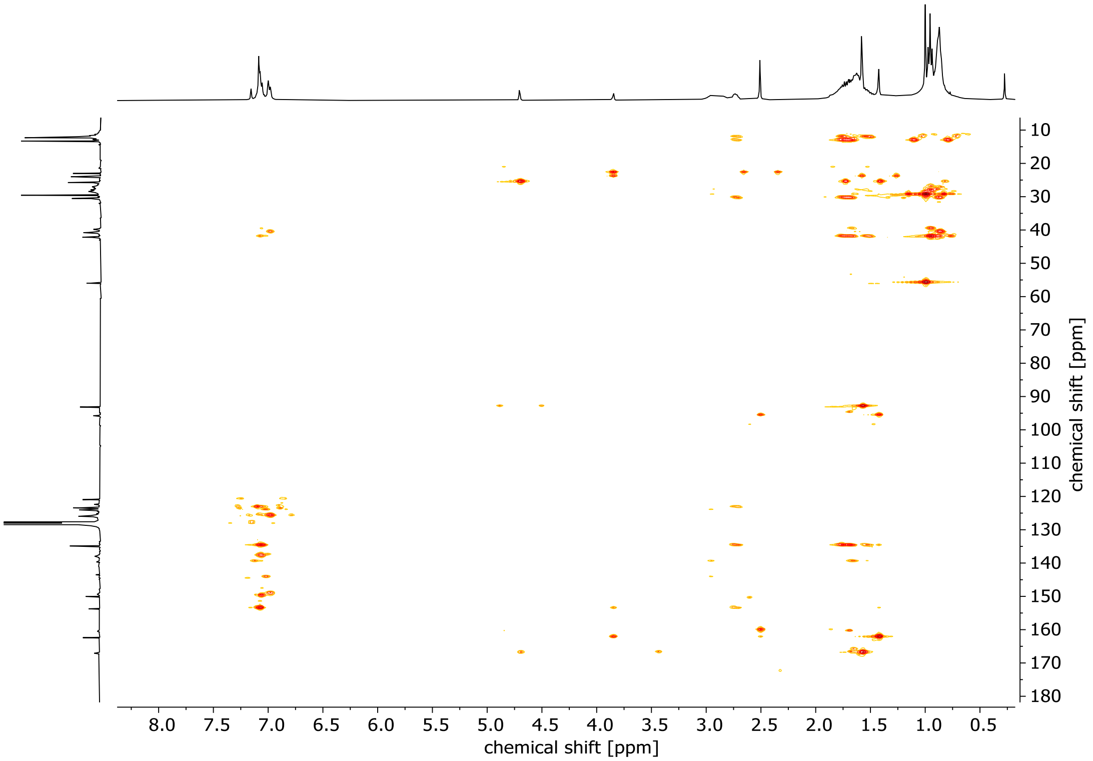

Figure S28. ^{1}H-^{13}C HMBC NMR spectrum (400.13/100.62 MHz, C_6D_6, 298K) of $(BDI^*)_2Ca$·(CN–*t*Bu) (**5**).

4. NMR Studies

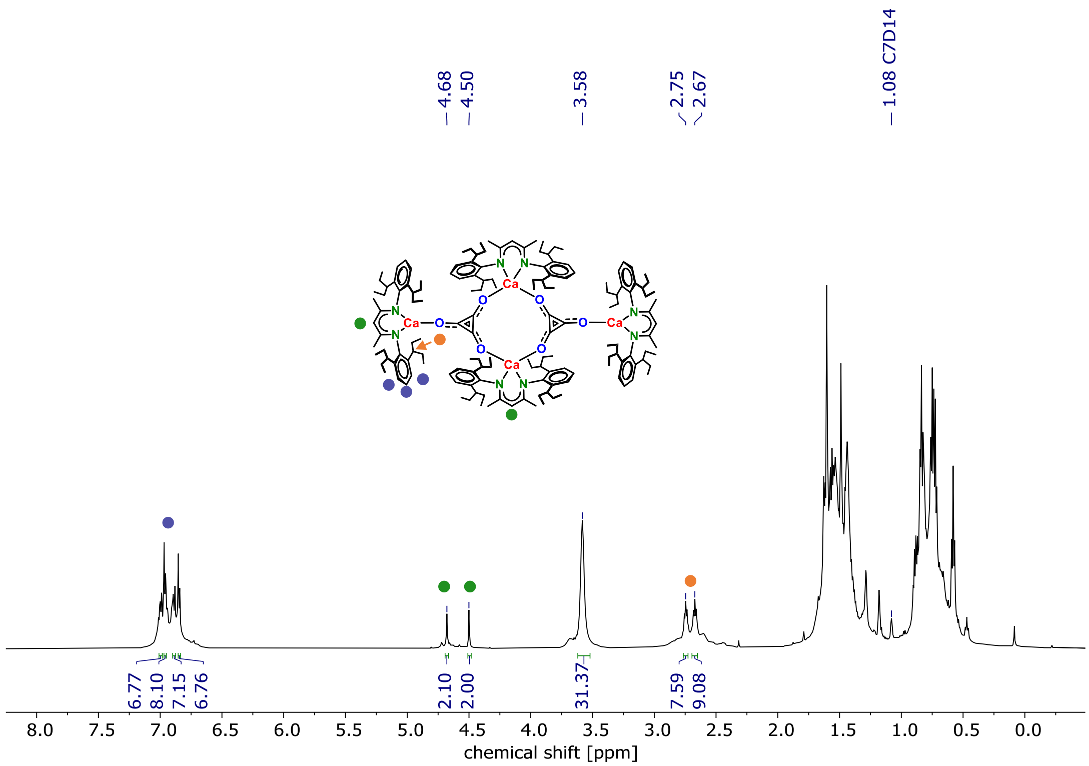

Figure S29. ^{1}H NMR spectrum (600.13 MHz, C_7D_{14}, 298K) of the crude product from conversion of $[(BDI^*)Ca(THP)]_2(N_2)$ with CO shows selective and near quantitative formation of the deltate complex **1**. Characteristic signals are marked.

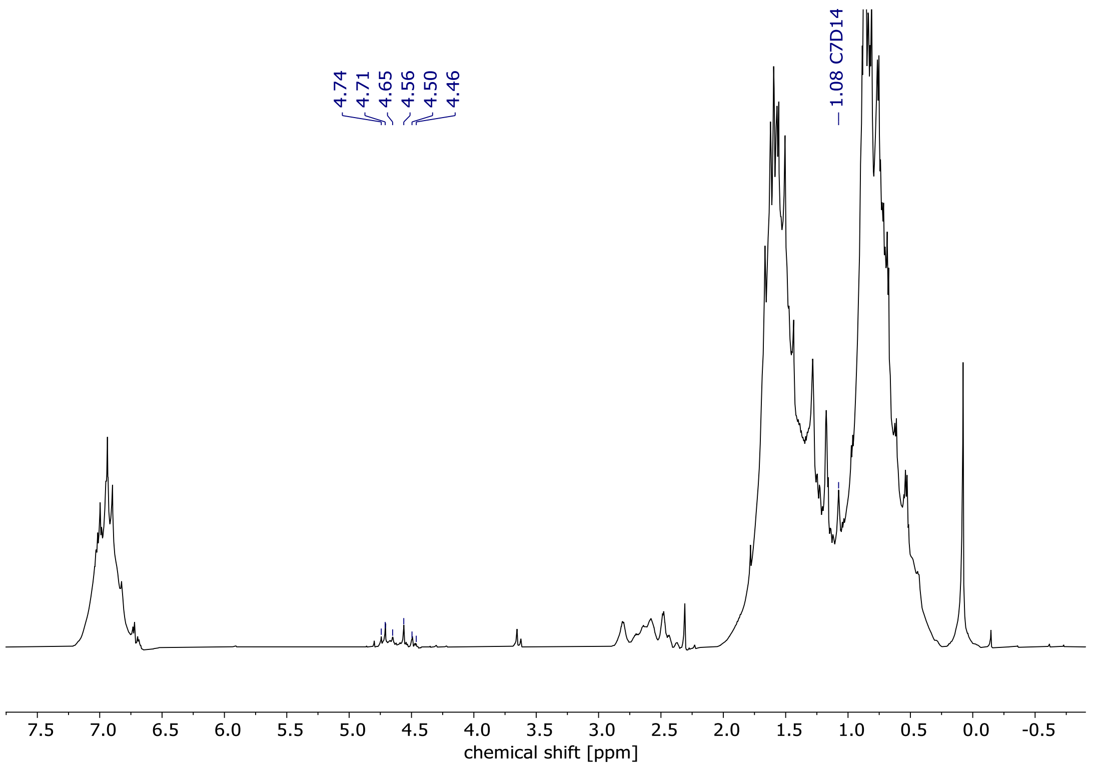

Figure S30. Crude ^{1}H NMR spectrum (600.13 MHz, C_7D_{14}, 298K) from conversion of THP-free Ca^{I} synthon $[(BDI^{*})Ca]_2(N_2)$ with CO shows rapid conversion and unselective formation of various products. However, the reduction is less selective than compared to the conversion of CO with the THP adduct $[(BDI^{*})Ca(THP)]_2(N_2)$ (see Figure S29).

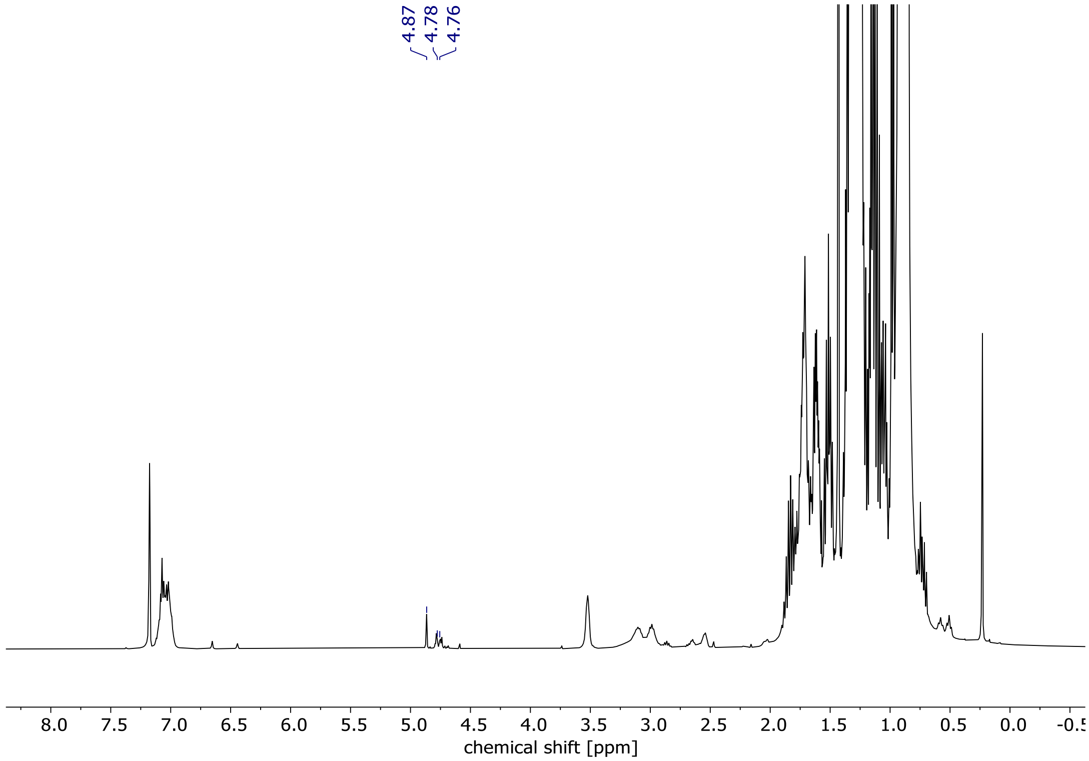

Figure S31. ^1H NMR spectrum (600.13 MHz, C_6D_6, 298K) of dried reaction mixture from conversion of $[(BDI^*)Ca(THP)]_2(N_2)$ with Cy–NC. This shows formation of one major product (methine signal at 4.87 ppm) and some side products (signals in range 4.6-4.8 ppm).

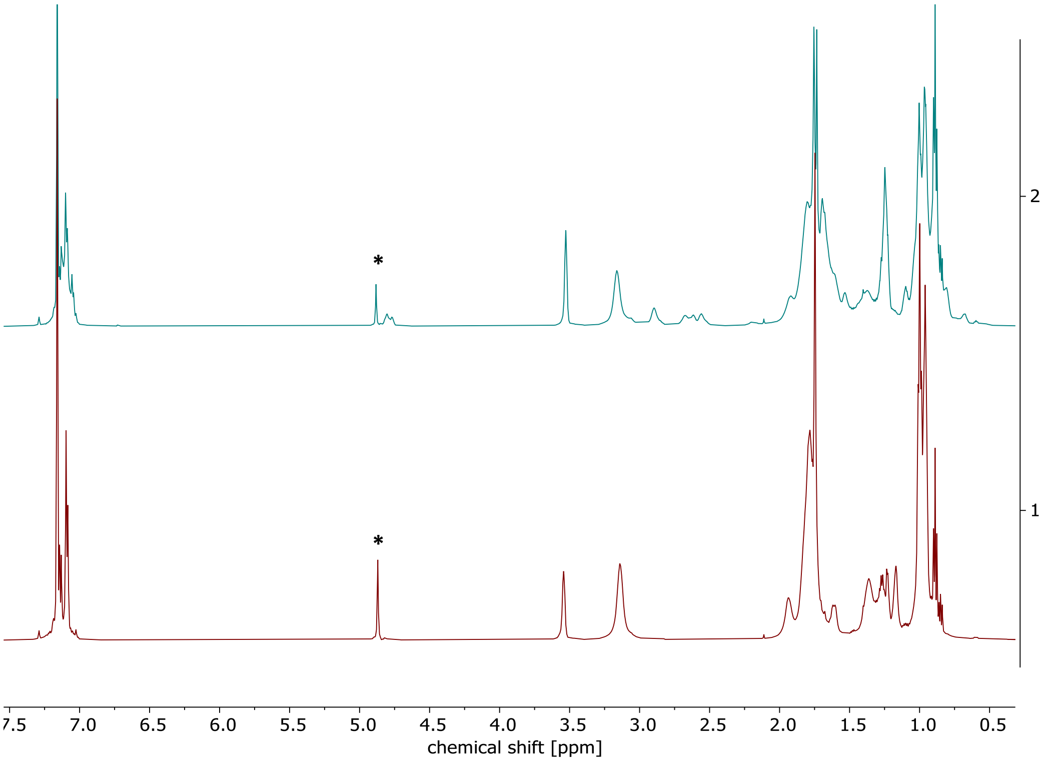

Figure S32. Comparison of ^{1}H NMR spectra of crystalline [(BDI*)Ca]$_2$(C$_3$(NCy)$_3$)(THP) (**2**) in C_6D_6 (bottom) with a C_6D_6 solution of **2** that has been kept at room temperature for 2 hours (top) showing the instability of **2** in solution and the formation of several unidentified decomposition products.

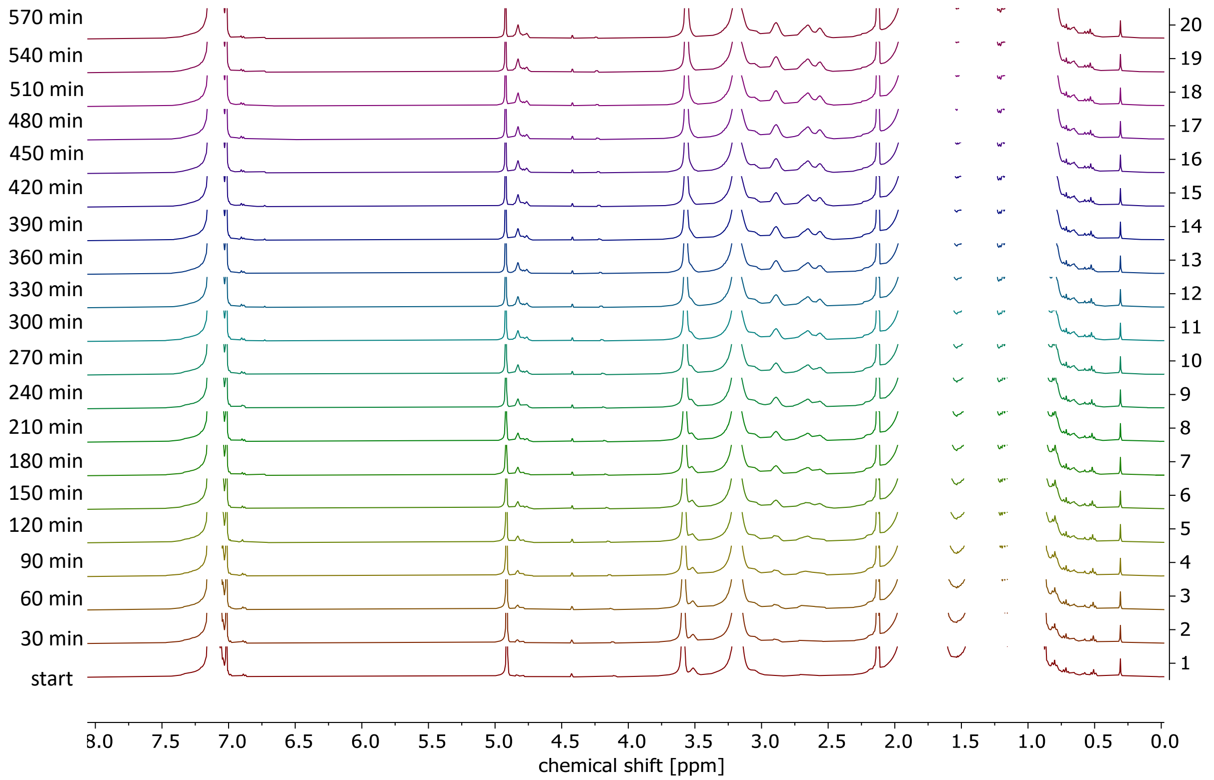

Figure S33. Stacked ^1H NMR spectra (400.13 MHz, C_7D_{14}, 298K) of $[(BDI^*)Ca]_2(C_3(NCy)_3)(THP)$ (**2**) recorded every 30 minutes showing its decomposition over time.

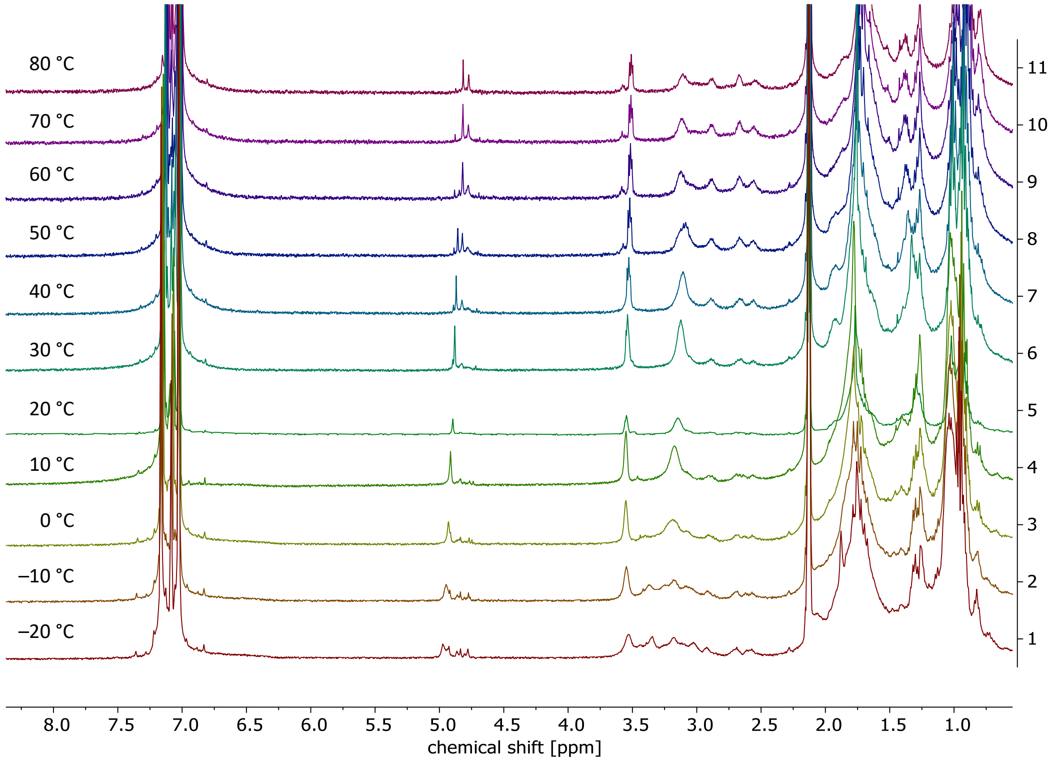

Figure S34. Variable temperature ^{1}H NMR spectra of $[(BDI^{*})Ca]_2(C_3(NCy)_3)(THP)$ (**2**) in C_7D_8 from –20 °C to +80 °C. Cooling shows formation of several species (indicative are the BDI* backbone CH signals in the range 4.75–5.00 ppm). This process is reversible when the solution is warmed to room temperature again. Heating results in irreversible decomposition of **2**.

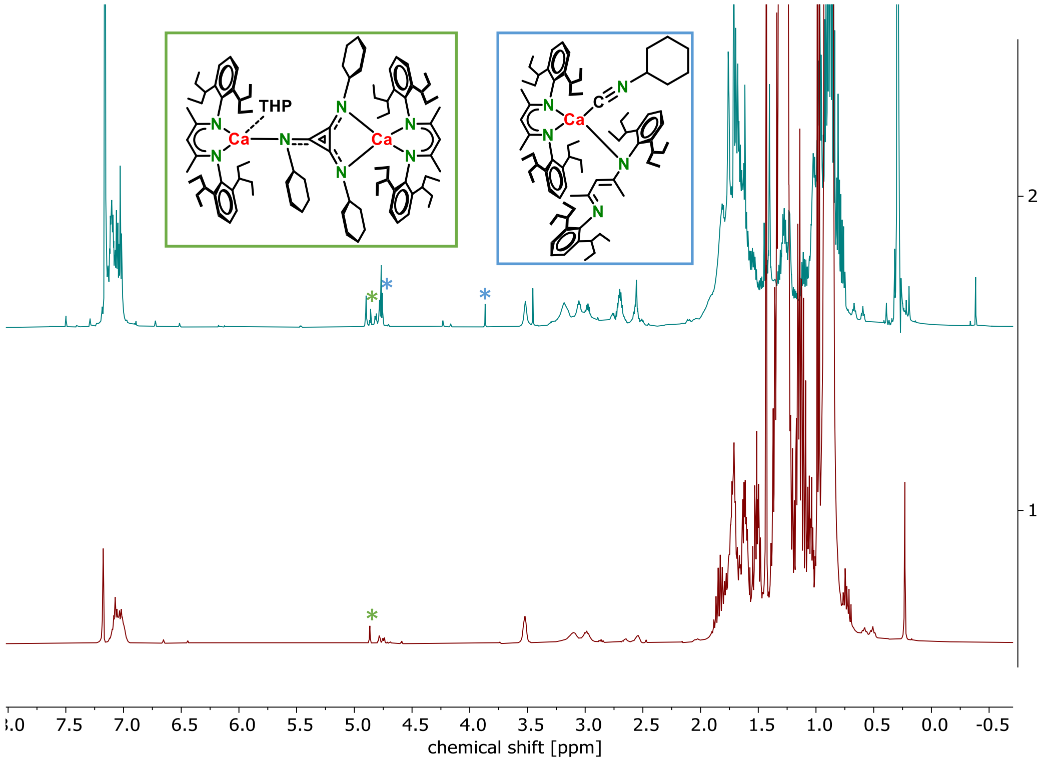

Figure S35. Bottom: ^{1}H NMR spectrum (600.13 MHz, C_6D_6, 298K) of dried reaction mixture from conversion of $[(BDI^*)Ca(THP)]_2(N_2)$ with 3.20 eq. Cy–NC, which gives mainly triimino deltate **2** (green asterisks). Top: ^{1}H NMR spectrum (600.13 MHz, C_6D_6, 298K) of dried reaction mixture from conversion of $[(BDI^*)Ca(THP)]_2(N_2)$ with 2.05 eq Cy–NC, which gives only a small quantity of triimino deltate **2** (green asterisks) but a larger amount of the side product (BDI*)Ca·(CN-Cy) **3** (blue asterisks) and other unidentified products.

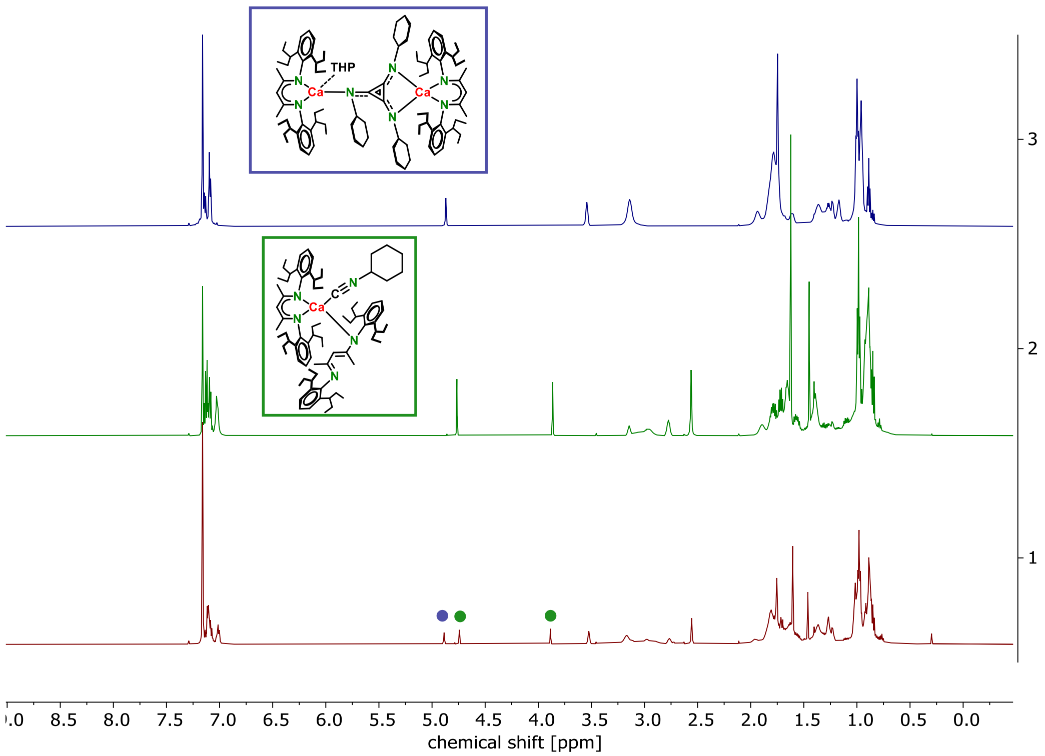

Figure S36. Bottom: ^{1}H NMR spectrum (600.13 MHz, C_6D_6, 298K) of second crop of crystals isolated in the synthesis of **2**. Middle: Reference ^{1}H NMR spectrum (600.13 MHz, C_6D_6, 298K) of **3**. Top: Reference ^{1}H NMR spectrum (600.13 MHz, C_6D_6, 298K) of **2**.

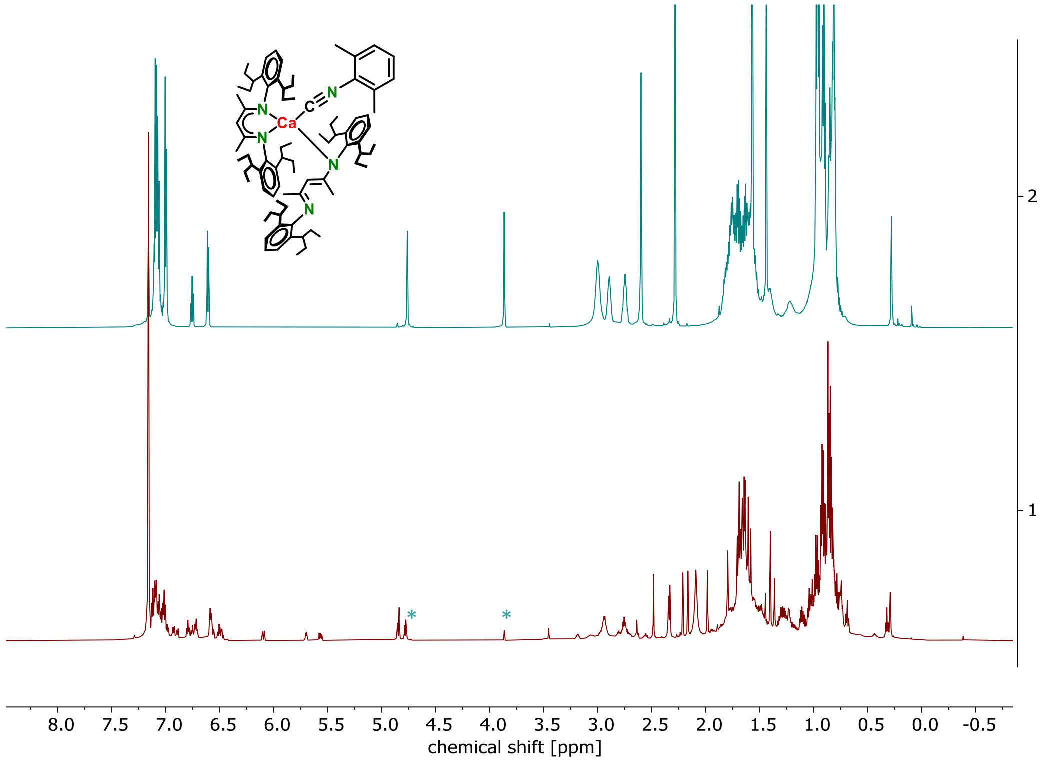

Figure S37. Bottom: ^{1}H NMR spectrum (600.13 MHz, C_6D_6, 298K) of dried reaction mixture from conversion of $[(BDI^*)Ca(THP)]_2(N_2)$ with Xyl–NC, showing formation of several products of which **4** is marked with green asterisk. Top: ^{1}H NMR spectrum (600.13 MHz, C_6D_6, 298K) of isolated complex **4**.

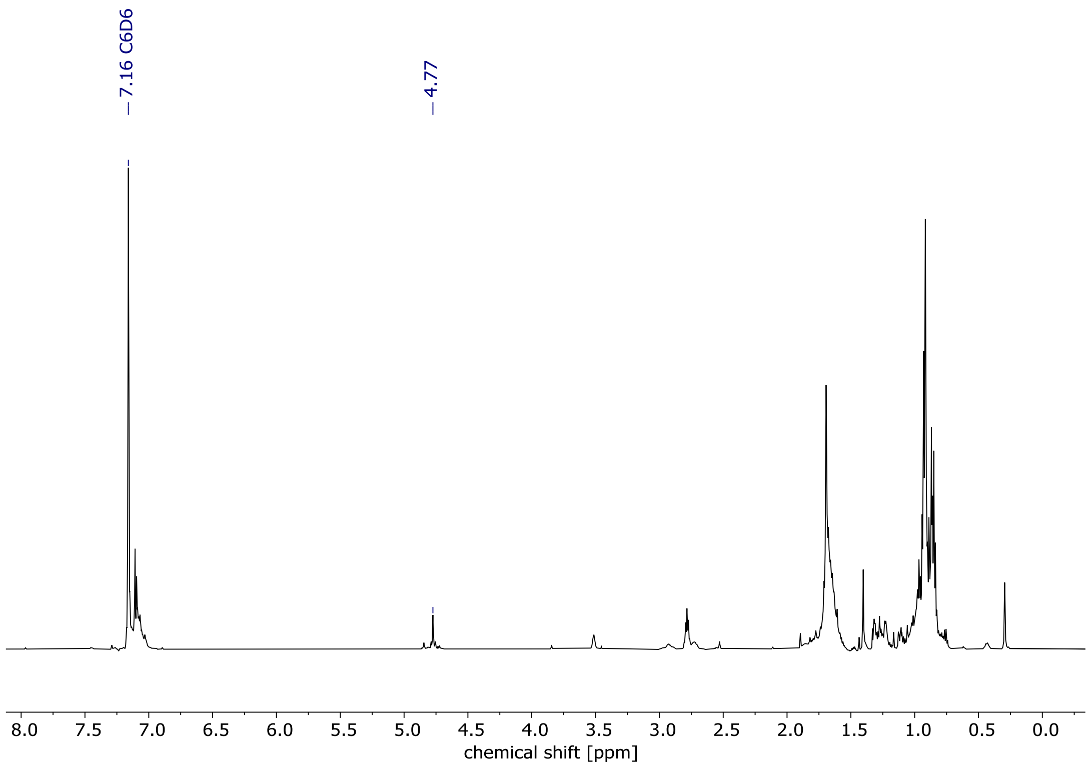

Figure S38. ^{1}H NMR spectrum (600.13 MHz, C_6D_6, 298K) of dried reaction mixture from conversion of $[(BDI^*)Ca(THP)]_2(N_2)$ with *t*Bu–NC. A major singlet at 4.77 ppm for the backbone CH of the BDI* ligand shows formation of one major product which we propose to be a cyclic deltate complex similar to **2**.

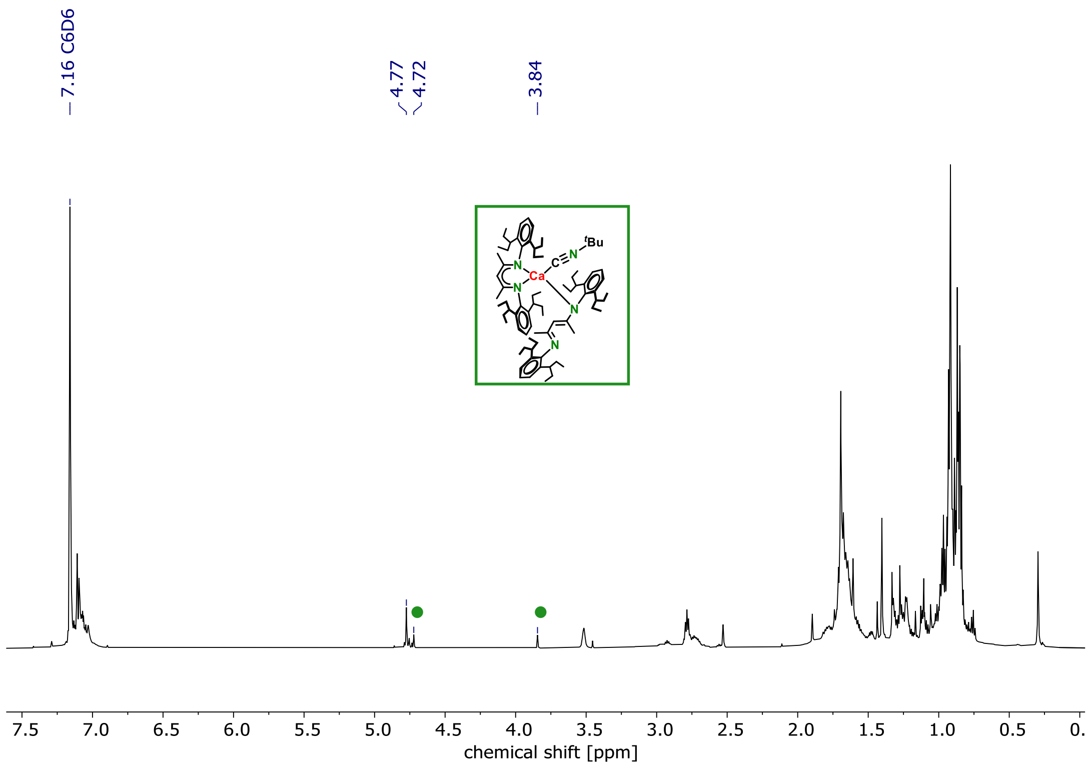

Figure S39. ^{1}H NMR spectrum (600.13 MHz, C_6D_6, 298K) of a solution of the product from conversion of $[(BDI^*)Ca(THP)]_2(N_2)$ with *t*Bu–NC after 4 days at room temperature. Formation of (BDI*)Ca·(CN-Cy) **5** is visible. The ligand backbone methine signals of **5** are marked with green dots.

Variable temperature ^{1}H NMR of (BDI*)$_2$Ca·(CN–R) (3-5)

Variable temperature ^{1}H NMR spectra of the Ca complexes **3**-**5** (Figures S40–S42) show upon temperature lowering decoalescence. The broad room temperature signals split into two signals, one for the homoleptic complex (BDI*)$_2$Ca (green asterisks) and the other presumably for the coordinated isonitrile analog (BDI*)$_2$Ca·(CN–R) (**3**-**5**). In case of (BDI*)$_2$Ca·(CN–Xyl) (**5**) only signals of (BDI*)$_2$Ca can be observed at lower temperatures (Figure S42) which may be attributed to weak coordination of the isonitrile ligand CN–Xyl.

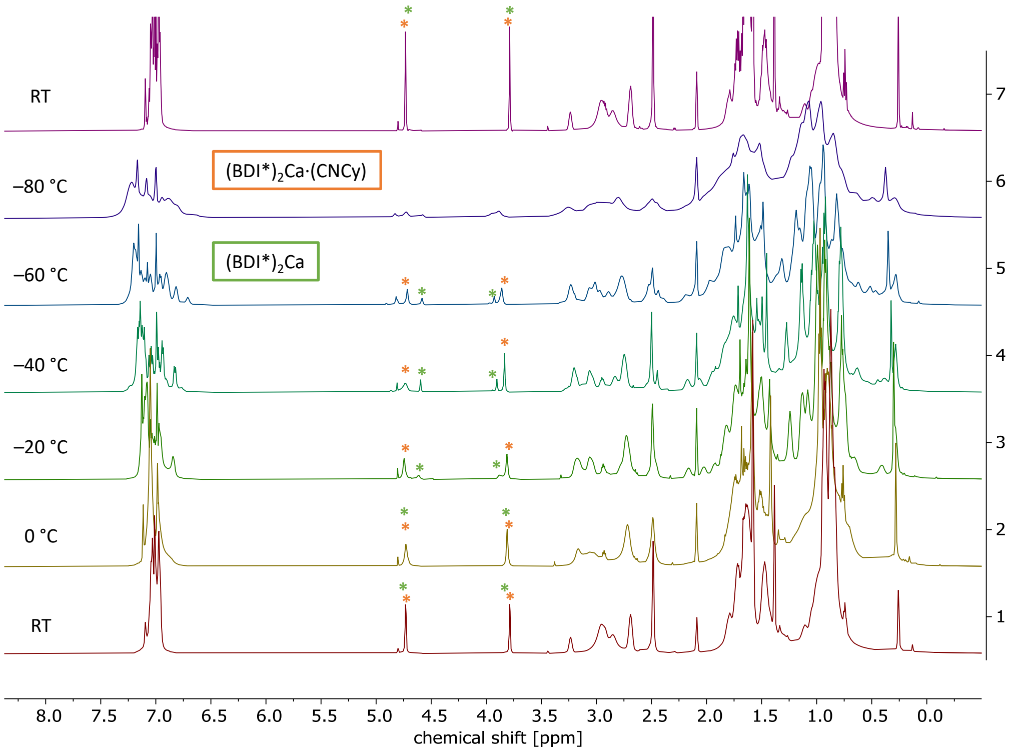

Figure S40. Variable temperature ^{1}H NMR spectra (600.13 MHz, C_7D_8) of (BDI*)$_2$Ca·(CN–Cy) (**3**).

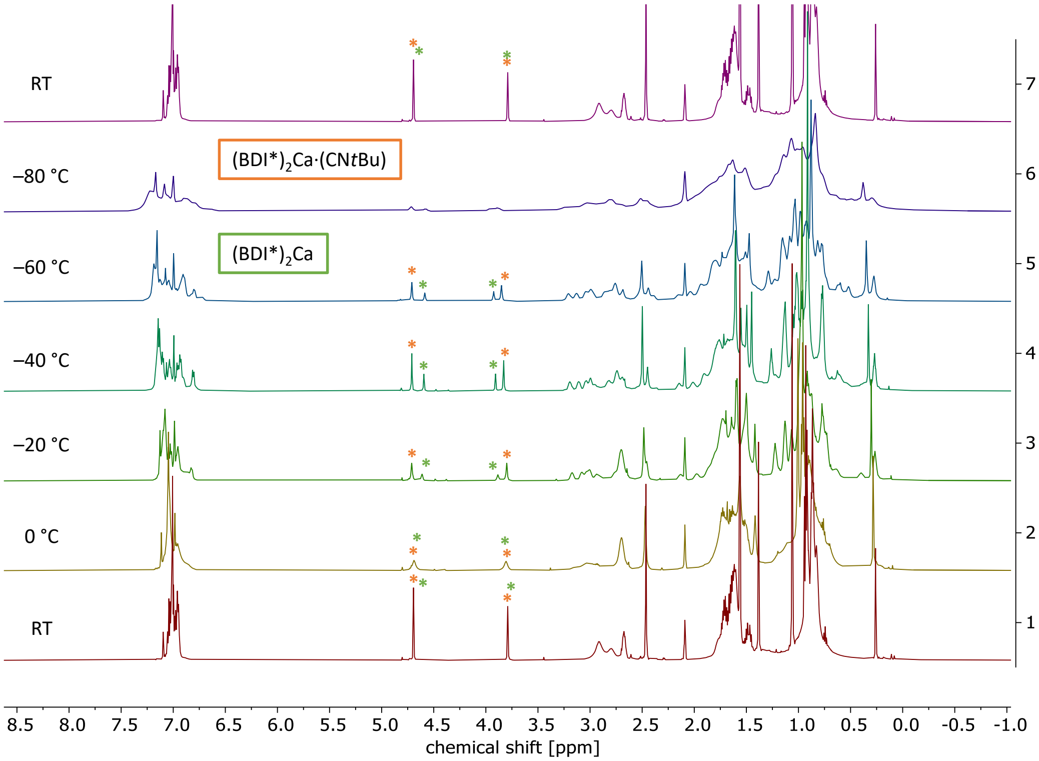

Figure S41. Variable temperature ^{1}H NMR spectra (600.13 MHz, C_7D_8) of (BDI*)$_2$Ca·(CN–*t*Bu) (**4**).

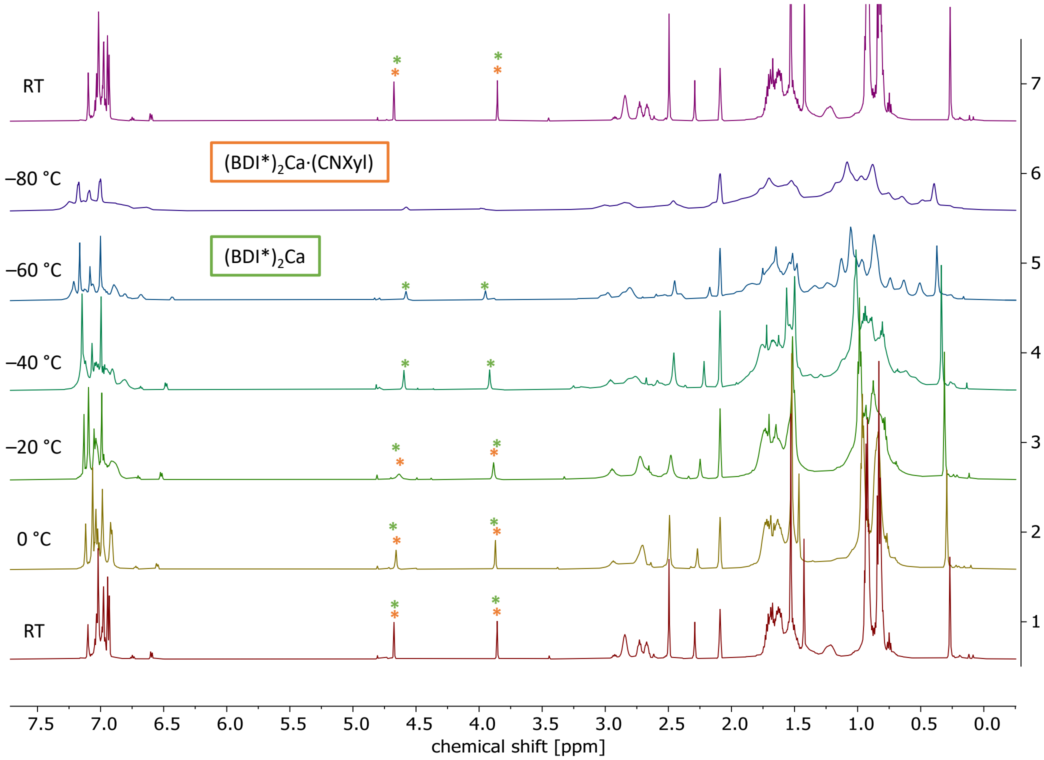

Figure S42. Variable temperature ^{1}H NMR spectra (600.13 MHz, C_7D_8) of (BDI*)$_2$Ca·(CN–Xyl) (**5**).

5. Crystal Structure Determinations

Suitable single crystals of compounds **1**-**5** were embedded in protective perfluoropolyalkylether oil (viscosity 1800 cSt; ABCR GmbH) on a microscope slide and a single specimen was selected and subsequently transferred to the cold nitrogen gas stream of the diffractometer.

The intensity data was collected at 100 K using CuK_α radiation (λ = 1.54184 Å) on an Agilent SuperNova dual radiation diffractometer with microfocus X-ray sources and mirror optics. The measured data were processed with the CrysAlisPro software package.[S2,S3] Data were corrected for Lorentz and polarization effects, and an empirical absorption correction using spherical harmonics as well as a numerical absorption correction based on gaussian integration over a multifaceted crystal model were applied. Using Olex2,[S4] the structures were solved by dual-space methods (SHELXT)[S5] and refined by full-matrix least-squares procedures on F^2 using SHELXL.[S6] All non-hydrogen atoms were refined with anisotropic displacement parameters. Most H-atoms were placed in geometrically calculated positions and refined by using a riding model where each H-atom was assigned a fixed isotropic displacement parameter with a value equal to 1.2U_{eq} (CH or CH_2) or 1.5U_{eq} (CH_3) of its parent C-atom.

The structure of [{(BDI*)Ca}$_4$(THP)$_3$(C_3O_3)$_2$] (**1**) also suffered from disorder. Here, a O_2Ca(BDI*) moiety, a NC(Me)CHC(Me)NDIPeP moiety, a DIPeP group, two out of three THP ligands, two additional 3-pentyl groups as well as two out of three *n*-pentane solvent molecules were involved. The disorder was modeled with the help of similarity restraints (SADI, SIMU) and rigid bond restraints (RIGU).[S7] For the *n*-pentane moieties, additional DFIX restraints were used to ensure a stable refinement and reasonable geometries. The relative occupancies of the two alternative orientations of these groups were refined to 0.5729(18)/0.4271(18) (O_2Ca(BDI*)), 0.724(2)/0.276(2) (NC(Me)CHC(Me)NDIPeP), 0.625(5)/0.375(5) (DIPeP), 0.724(2)/0.276(2) (THP 1), 0.553(7)/0.447(7) (THP 2), 0.776(7)/0.224(7) (3-pentyl 1), 0.750(6)/0.250(6) (3-pentyl 2), 0.522(7)/0.478(7) (*n*-pentane 1) and 0.602(7)/0.398(7) (*n*-pentane 2), respectively. In parts of the disordered O_2Ca(BDI*) moiety, it was necessary to include a third orientation in order to better describe the disorder. Here, site occupancy factors of 0.4271(18), 0.382(2) and 0.191(2) were obtained. Generation of the cif file with Olex2/ShelXL[S4,S6] led to implausible values for the standard deviation of some torsion angles within the deltate anions (e.g. O4-C49-C50-C48 179(100)°), which resulted in level A alerts with checkcif. Since this was clearly a software issue and not a crystallographic problem, these values were manually replaced in the cif file with the values obtained *via* the esd command in Olex2[S4] (e.g. O4-C49-C50-C48 179.1(5)°).

For the investigated crystal of compound [{(BDI*)Ca}$_2$(C$_3$(NCy)$_3$)(THP)] (**2**), the asymmetric unit consisted of half of the molecule and 0.5 *n*-hexane and showed heavy disorder. The central part of **2** (the Ca{(CyN)$_3$C$_3$}Ca(THP) moiety) was disordered about an inversion center in the middle of the molecule. Consequently, the site occupancy factor for this moiety was constrained to 0.5. However, it was necessary to additionally introduce two alternative orientations for the THP ligand with site occupancy factors of 0.279(4) and 0.221(4). Three out of four 3-pentyl groups of the BDI* ligand and the co-crystallized *n*-hexane were disordered as well. The relative occupancies of the two alternative orientations of these groups were refined to 0.790(4)/0.210(4) (3-pentyl 1), 0.662(6)/0.338(6) (3-pentyl 2), 0.847(5)/0.153(5) (3-pentyl 3) and 0.556(6)/0.444(6) (0.5 *n*-hexane), respectively. During refinement of the above-mentioned disordered parts of **2**, a substantial amount of similarity restraints (SADI, SIMU) and rigid bond restraints (RIGU)[S7] were applied. During the final stages of the refinement, it was noticed that the hydrogen atom H3 in the ligand backbone deviated significantly from the position calculated *via* the riding model. Therefore, this H atom was placed in the position indicated by a difference electron density map and its positions was refined together with an isotropic displacement parameter.

Substantial disorder was also present in case of compound [(BDI*)$_2$Ca·(CN–Cy)] (**3**). Here, a NDIPeP moiety, three additional 3-pentyl groups and the cyclohexyl group were affected. The disorder was modeled with the help of similarity restraints (SADI, SIMU) and rigid bond restraints (RIGU),[S7] and site occupancy factors of 0.753(2)/0.247(2) (NDIPeP), 0.875(5)/0.125(5) (3-pentyl 1), 0.541(5)/0.459(5) (3-pentyl 2), 0.549(7)/0.491(7) (3-pentyl 3) and 0.840(3)/0.160(3) (cyclohexyl) were determined, respectively. The co-crystallized solvent was found to be disordered as well. The solvent cavity was filled with a mixture of hexane isomers, but a suitable solvent model could not be built. Therefore, their contribution to the structure factors was secured by back-Fourier transformation using the solvent mask routine[S8] of the program Olex2.[S4] The solvent accessible voids treated this way had a size of 456.1 Å^3 (11.3% of the unit cell) and contained 100.9 electrons/unit cell. This is consistent with the presence of 1 C_6H_{14} per formula unit which account for 100 electrons per unit cell.

The structure of [(BDI*)$_2$Ca·(CN–Xyl)] (**4**) suffered from disorder as well. Here, a DIPeP moiety and two additional 3-pentyl groups adopted two alternative orientations. Refinement of the disorder succeeded with the help of similarity restraints (SADI, SIMU) and rigid bond restraints (RIGU).[S7] For one atom (C32A) within the less occupied orientation of the DIPeP group, an additional ISOR was necessary in order to achieve a stable refinement. This treatment led to site occupancy factors of 0.881(3)/0.119(3) (DIPeP), 0.785(5)/0.215(5) (3-pentyl 1) and 0.668(5)/0.332(59, respectively.

In case of [$(BDI^*)_2Ca\cdot(CN{-}tBu)$] (**5**), only the N*t*Bu group and the co-crystallized solvent was affected by disorder. The two alternative orientations of the N*t*Bu moiety showed site occupancy factors of 0.502(3) and 0.498(3). The solvent cavity was occupied mainly by *n*-hexane, but there was significant contribution of other hexane isomers, that could not be modeled satisfactorily. (An isomeric mixture of hexanes was used for crystallization.) Therefore, a solvent mask[S8] was calculated for this solvent moiety using Olex2[S4] and 52.8 electrons were found in a volume of 338.0 A^3 (8.8% of the unit cell) in one void. This is consistent with the presence of 0.5 hexane per formula unit which accounts for 50 electrons per unit cell.

The crystal structure data has been deposited with the Cambridge Crystallographic Data Centre. CCDC 2428665-2428669 contain the supplementary crystallographic data for the complexes. This data can be obtained free of charge from The Cambridge Crystallographic Data Centre via www.ccdc.cam.ac.uk/data_request/cif.

Crystallographic and refinement data are summarized in Table **S1**.

Table S1. Crystal data and structure refinement for compounds **1-5**.

Compound	[{(BDI*)Ca}$_4$(THP)$_3$(C$_3$O$_3$)$_2$]·3(*n*-Pentane) (**1**)	[{(BDI*)Ca}$_2$(C$_3$(NCy)$_3$)(THP)]·*n*-Hexane (**2**)	[(BDI*)$_2$Ca·(CN–Cy)]·Hexane (**3**)
Identification code	hasj221019a	hasj210609a	hasj210813a
Empirical formula	$C_{184}H_{294}Ca_4N_8O_9$	$C_{106}H_{171}Ca_2N_7O$	$C_{87}H_{139}CaN_5$
Formula weight	2922.57	1639.65	1295.10
Temperature/K	100.0(2)	100.0(2)	100.0(4)
Crystal system	triclinic	triclinic	triclinic
Space group	P-1	P-1	P-1
a/Å	18.2798(2)	12.6865(4)	13.4820(3)
b/Å	18.3492(2)	13.1676(5)	16.3223(5)
c/Å	27.9435(3)	16.5859(6)	20.2700(5)
α/°	77.9900(10)	82.572(3)	68.531(2)
β/°	77.6940(10)	88.483(3)	87.284(2)
γ/°	75.3370(10)	63.757(3)	76.379(2)
Volume/Å^3	8740.62(17)	2462.78(16)	4030.60(19)
Z	2	1	2
ρ_{calc}g/cm^3	1.110	1.106	1.067
μ/mm^{-1}	1.511	1.371	0.996
F(000)	3212.0	904.0	1432.0
Crystal size/mm^3	0.194 × 0.176 × 0.141	0.4 × 0.209 × 0.088	0.49 × 0.337 × 0.203
Radiation	Cu Kα (λ = 1.54184)	Cu Kα (λ = 1.54184)	Cu Kα (λ = 1.54184)
2Θ range for data collection/°	5.552 to 144.874	5.376 to 144.994	5.986 to 145.14
Index ranges	-22 ≤ h ≤ 21, -21 ≤ k ≤ 22, -34 ≤ l ≤ 34	-14 ≤ h ≤ 15, -16 ≤ k ≤ 16, -20 ≤ l ≤ 20	-16 ≤ h ≤ 16, -20 ≤ k ≤ 14, -25 ≤ l ≤ 24
Reflections collected	82794	27358	44579
Independent reflections	33569 [R_{int} = 0.0229, R_{sigma} = 0.0285]	9517 [R_{int} = 0.0252, R_{sigma} = 0.0234]	15569 [R_{int} = 0.0238, R_{sigma} = 0.0224]
Data/restraints/parameters	33569/14950/2998	9517/1132/891	15569/3373/1132
Goodness-of-fit on F^2	1.017	1.022	1.067
Final R indexes [I>=2σ (I)]	R_1 = 0.0648, wR_2 = 0.1776	R_1 = 0.0494, wR_2 = 0.1363	R_1 = 0.0585, wR_2 = 0.1652
Final R indexes [all data]	R_1 = 0.0741, wR_2 = 0.1878	R_1 = 0.0553, wR_2 = 0.1425	R_1 = 0.0632, wR_2 = 0.1708
Largest diff. peak/hole / e Å^{-3}	0.79/-0.49	0.34/-0.28	0.53/-0.35
CCDC number	2428669	2428665	2428666

Table S1. Crystal data and structure refinement for compounds **1-5** (continued).

Compound	[(BDI*)$_2$Ca·(CN–Xyl)] (**4**)	[(BDI*)$_2$Ca·(CN–*t*Bu)]·0.5Hexane (**5**)
Identification code	hasj211027a	hasj220207a
Empirical formula	$C_{83}H_{123}CaN_5$	$C_{82}H_{130}CaN_5$
Formula weight	1230.94	1225.98
Temperature/K	100.0(6)	100.0(5)
Crystal system	monoclinic	triclinic
Space group	P2$_1$/c	P-1
a/Å	17.1763(3)	13.5980(4)
b/Å	17.4722(2)	14.8279(5)
c/Å	26.1660(3)	21.9613(7)
α/°	90	75.108(3)
β/°	108.952(2)	77.232(3)
γ/°	90	64.237(3)
Volume/Å^3	7426.9(2)	3823.5(2)
Z	4	2
ρ_{calc}g/cm^3	1.101	1.065
μ/mm^{-1}	1.061	1.026
F(000)	2704.0	1354.0
Crystal size/mm^3	0.318 × 0.228 × 0.196	0.15 × 0.111 × 0.082
Radiation	Cu Kα (λ = 1.54184)	Cu Kα (λ = 1.54184)
2Θ range for data collection/°	6.192 to 144.848	6.734 to 144.918
Index ranges	-21 ≤ h ≤ 13, -21 ≤ k ≤ 20, -22 ≤ l ≤ 32	-16 ≤ h ≤ 14, -18 ≤ k ≤ 12, -26 ≤ l ≤ 22
Reflections collected	28338	25197
Independent reflections	14219 [R_{int} = 0.0305, R_{sigma} = 0.0416]	14615 [R_{int} = 0.0349, R_{sigma} = 0.0508]
Data/restraints/parameters	14219/2001/1019	14615/0/838
Goodness-of-fit on F^2	1.025	1.022
Final R indexes [I>=2σ (I)]	R_1 = 0.0427, wR_2 = 0.1048	R_1 = 0.0406, wR_2 = 0.0955
Final R indexes [all data]	R_1 = 0.0515, wR_2 = 0.1119	R_1 = 0.0509, wR_2 = 0.1024
Largest diff. peak/hole / e Å^{-3}	0.49/-0.42	0.24/-0.39
CCDC number	2428667	2428668

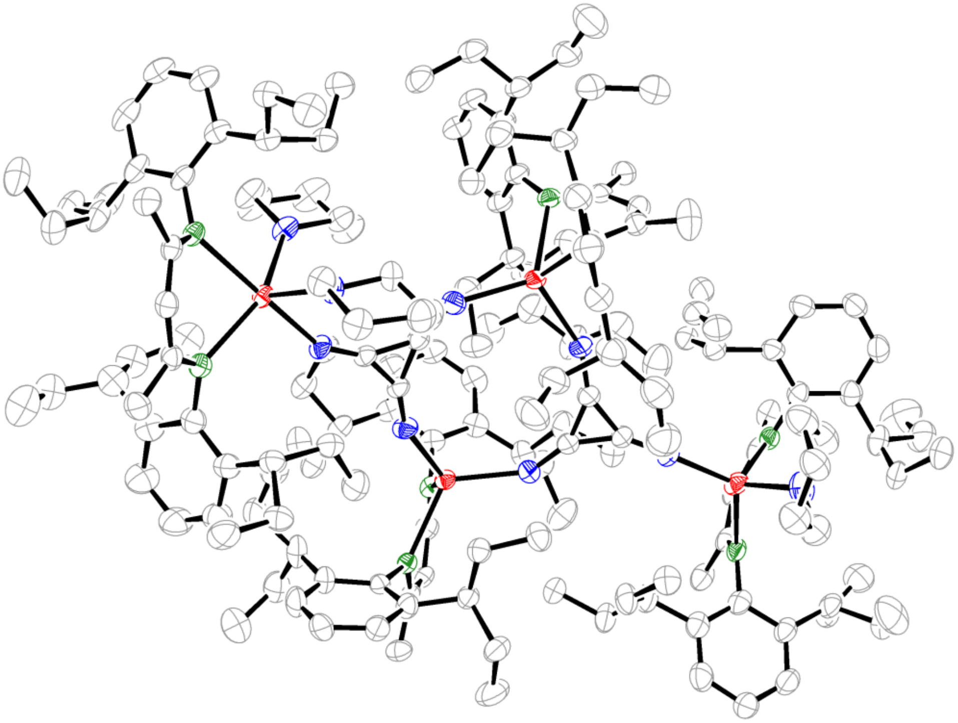

Figure S43. Solid state structure of [(BDI*)Ca]$_4$(THP)$_3$(C$_3$O$_3$)$_2$ (**1**). Ellipsoids represent 50% probability. Hydrogen atoms have been omitted for clarity.

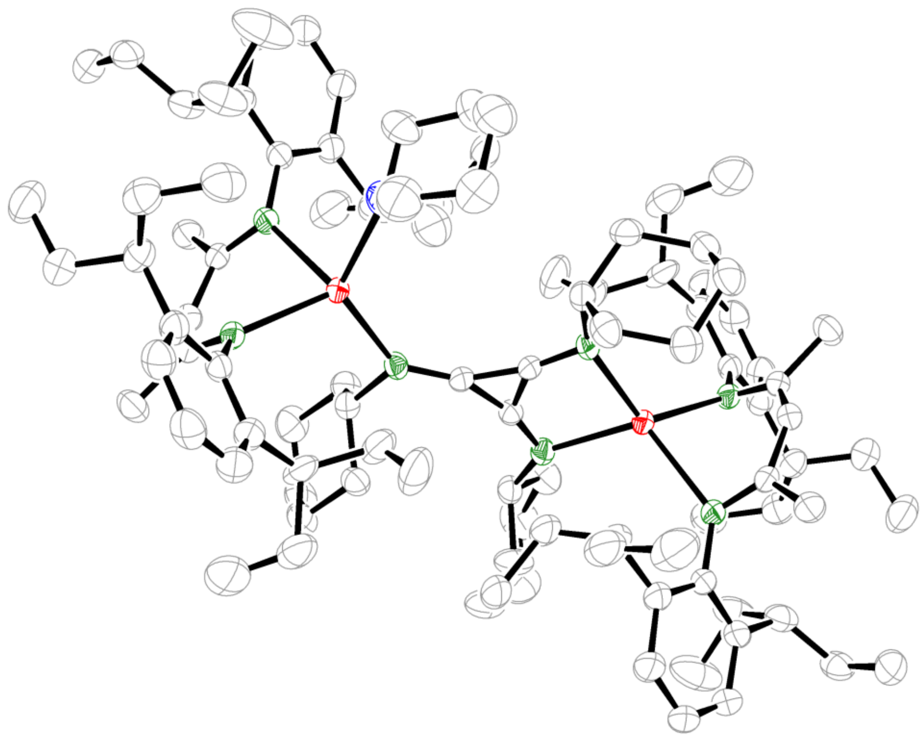

Figure S44. Solid state structure of [(BDI*)Ca]$_2$(C$_3$(NCy)$_3$)(THP) (**2**). Ellipsoids represent 50% probability. Hydrogen atoms have been omitted for clarity.

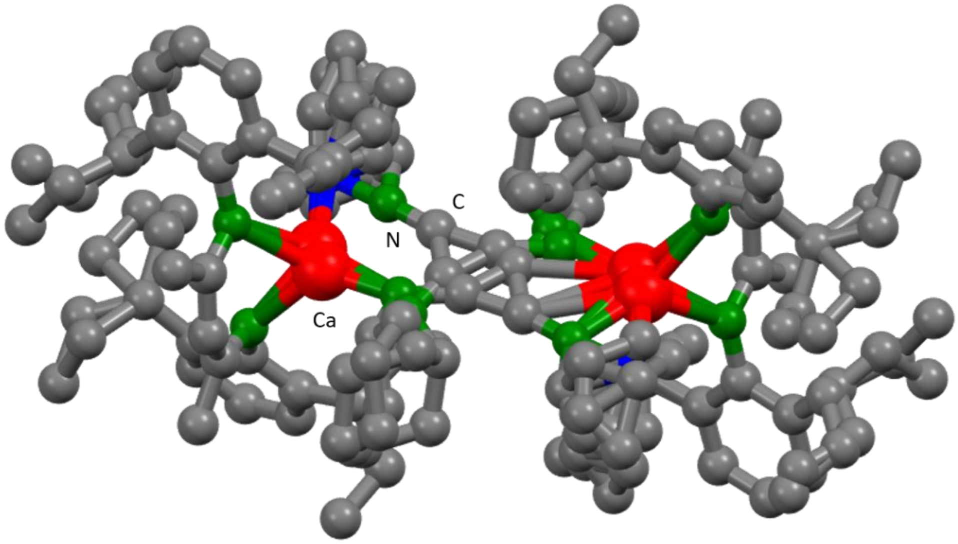

Figure S45. Solid state structure of [(BDI*)Ca]$_2$(C$_3$(NCy)$_3$)(THP) (**2**) showing disorder in the C$_3$(NCy)$_3^{2-}$ dianion over an inversion center in the middle of the molecule.

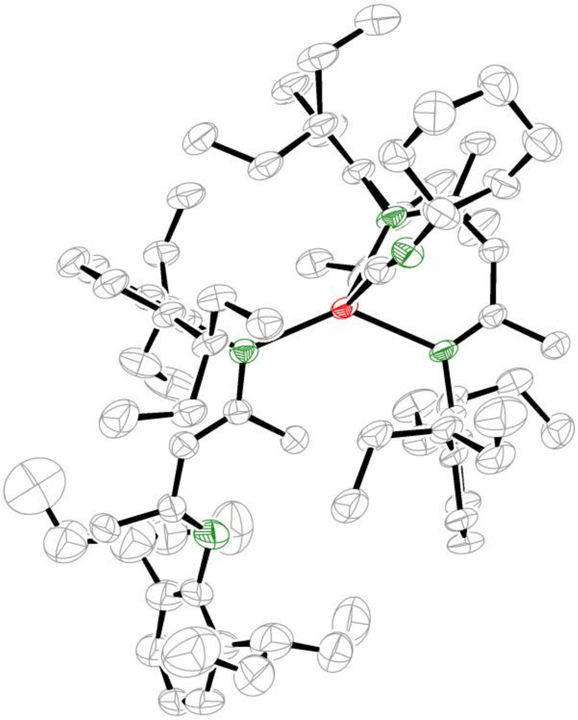

Figure S46. Solid state structure of (BDI*)$_2$Ca·(CN–Cy) (**3**). Ellipsoids represent 50% probability. Hydrogen atoms have been omitted for clarity.

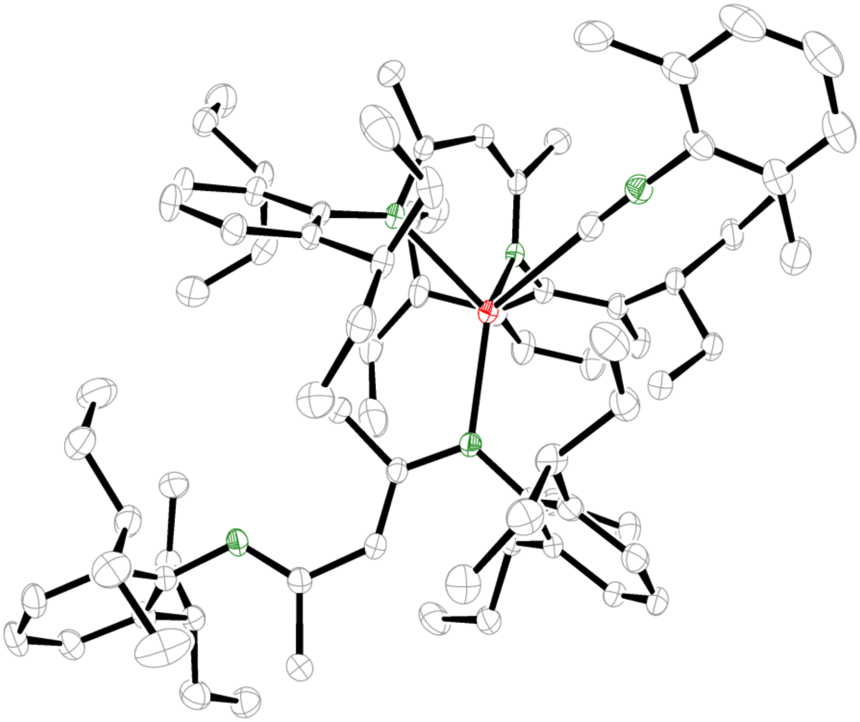

Figure S47. Solid state structure of $(BDI^{*})_2Ca{\cdot}(CN{-}Xyl)$ (**4**). Ellipsoids represent 50% probability. Hydrogen atoms have been omitted for clarity.

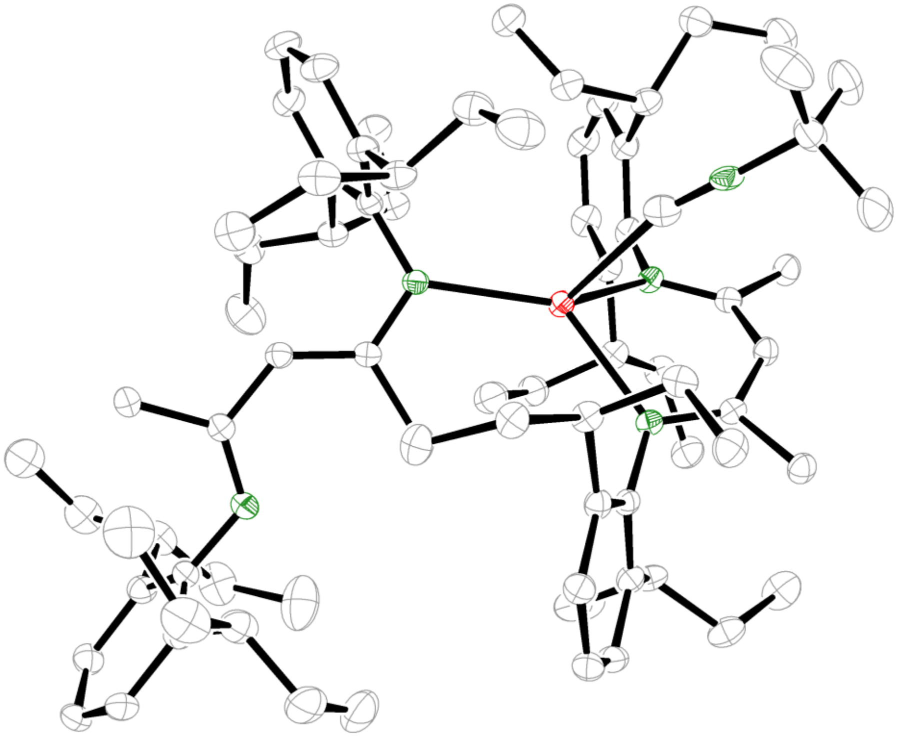

Figure S48. Solid state structure of (BDI*)$_2$Ca·(CN–*t*Bu) (**5**). Ellipsoids represent 50% probability. Hydrogen atoms have been omitted for clarity.

6. Computational Details

All calculations were carried out using Gaussian 16A.[S9] All methods were used as implemented. All structures were fully optimized at a B3PW91-GD3BJ/def2svp level of theory which includes Grimme D3 dispersion correction using Becke–Johnson dampening (GD3BJ).[S10–S14] All structures were characterized as true minima (Nimag = 0) or as transition states (Nimag = 1) by frequency calculations on the same level of theory. Energies were determined at a B3PW91-GD3BJ/def2tzvp level of theory. The same level of theory was used for calculation of the NPA charge calculations with NBO7.[S15] All structures were evaluated using Molecule 2.3.[S16] QTAIM analysis was carried out using AIMAll (v17) with the wave functions obtained at the B3PW91-GD3BJ/def2tzvp level of theory.[S17,S18]

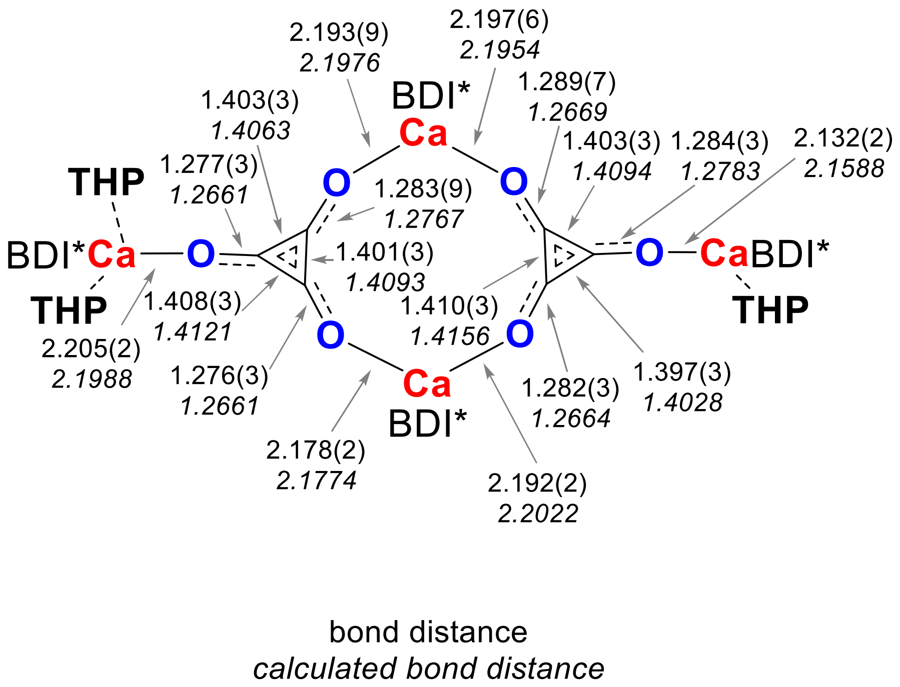

Figure S49. Comparison of calculated bond distances with those in the crystal structure (Å) of $[(BDI^*)Ca]_4(THP)_3(C_3O_3)_2$ (**1**).

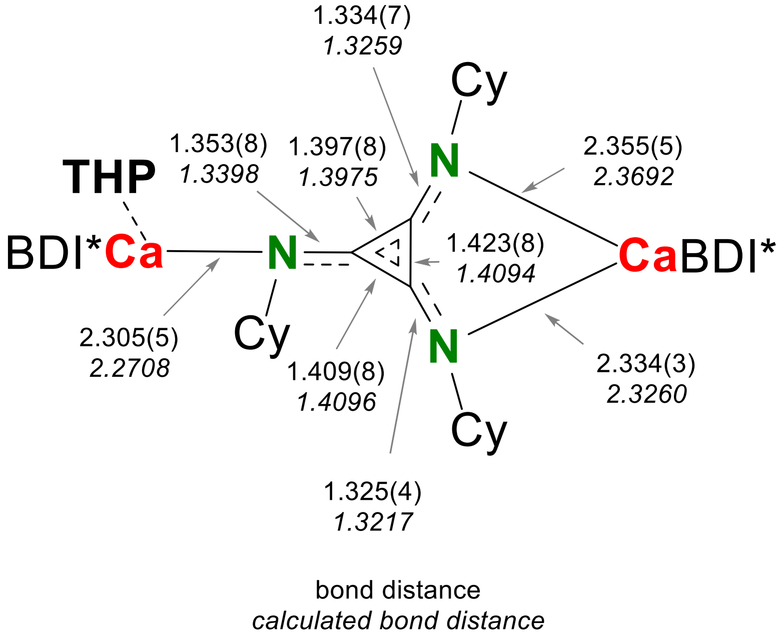

Figure S50. Comparison of calculated bond distances with those in the crystal structure (Å) of $[(BDI^*)Ca]_2(C_3(NCy)_3)(THP)$ (**2**).

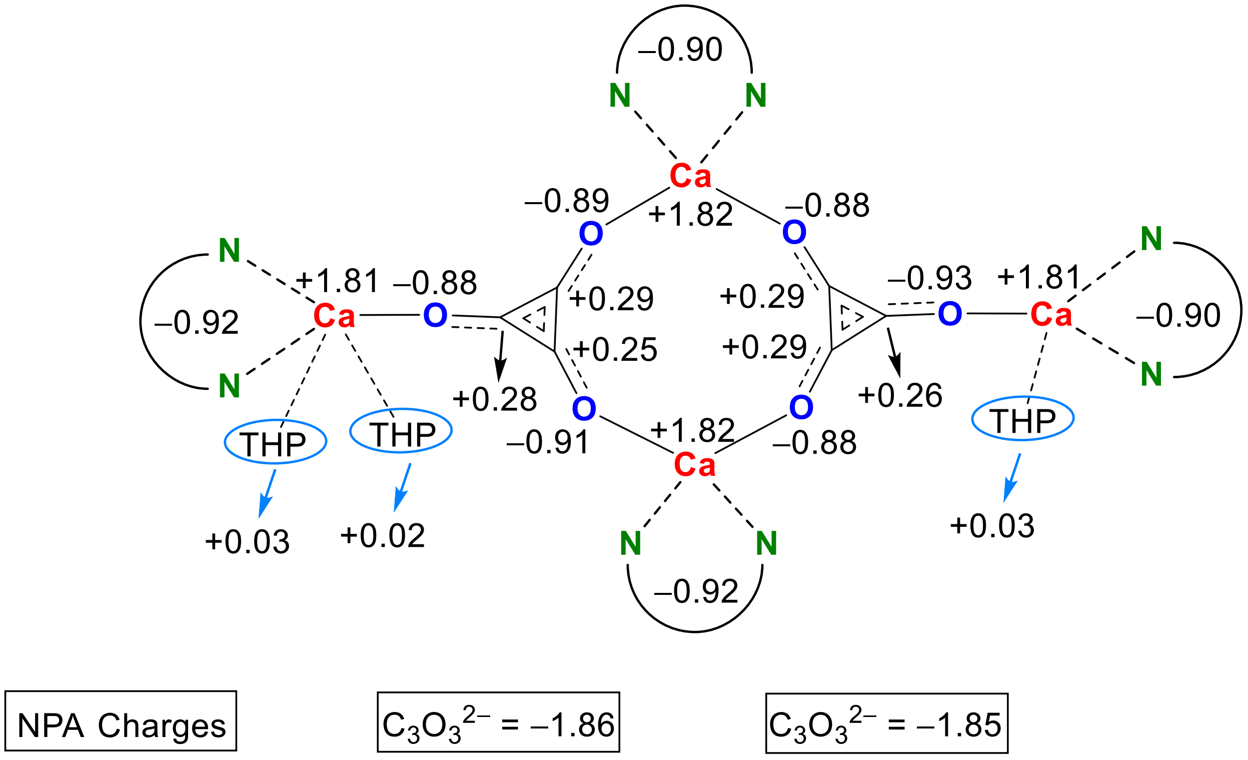

Figure S51. NPA charges of [(BDI*)Ca]$_4$(THP)$_3$(C_3O_3)$_2$ (**1**).

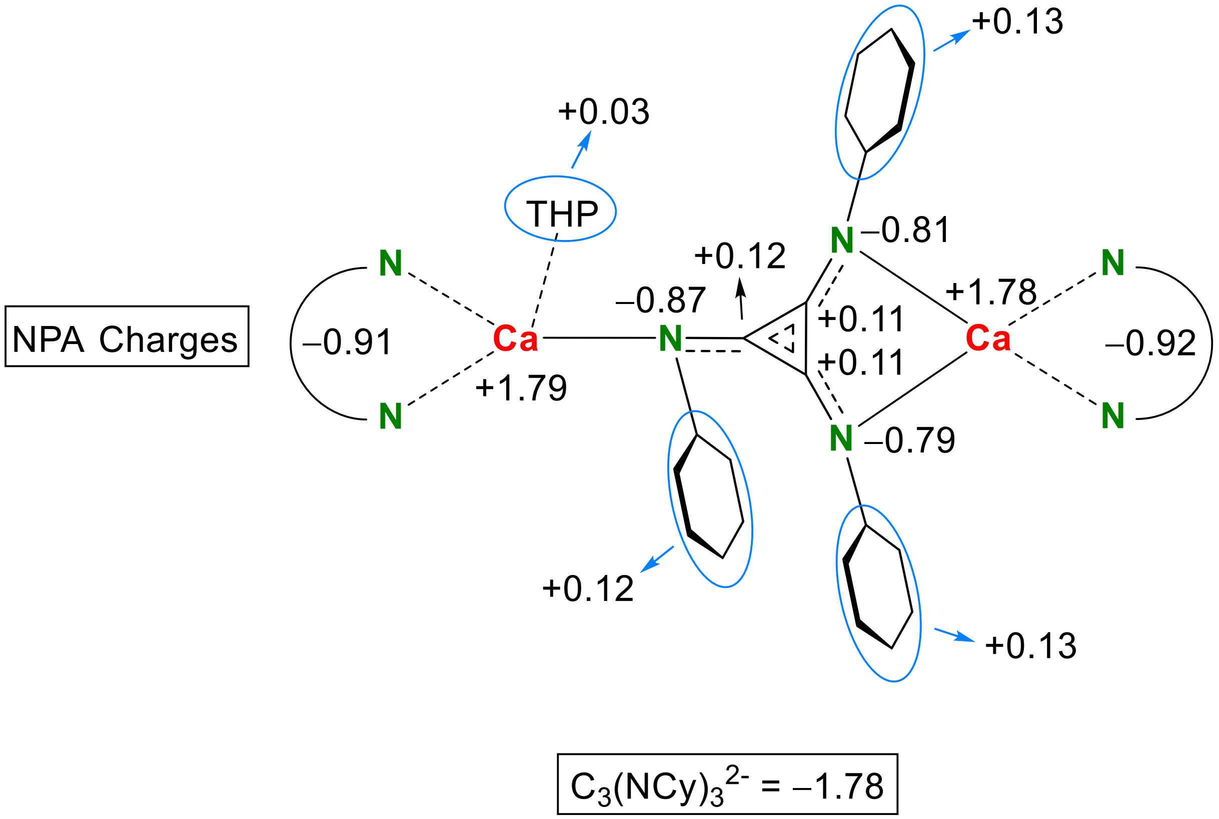

Figure S52. NPA charges of [(BDI*)Ca]$_2$(C_3(NCy)$_3$)(THP) (**2**).

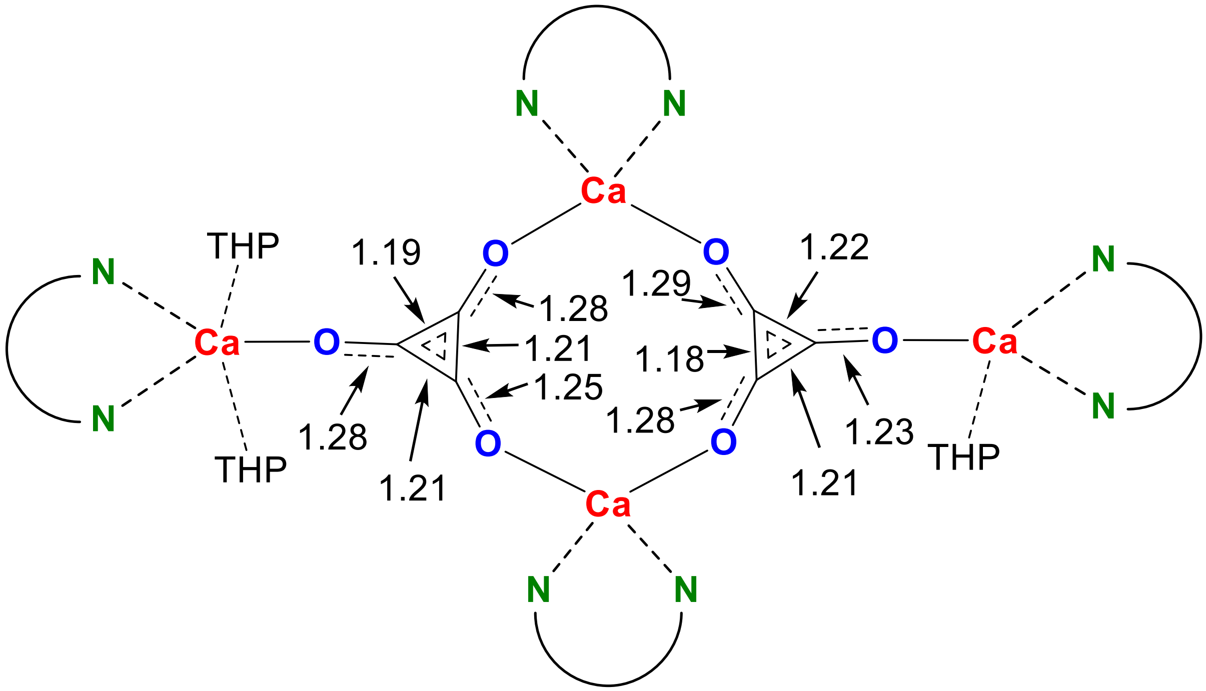

Wiberg Bond Indices

Figure S53. Wiberg Bond Indices (WBI) of the deltate dianions $C_3O_3^{2-}$ in $[(BDI^*)Ca]_4(THP)_3(C_3O_3)_2$ (**1**).

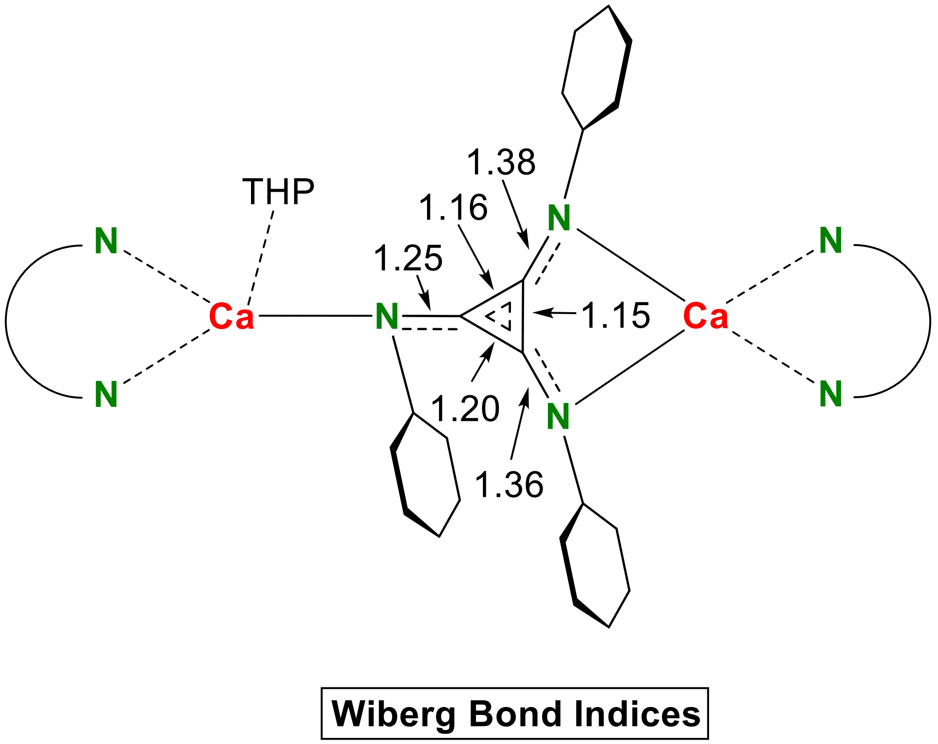

Figure S54. Wiberg Bond Indices (WBI) of the triimino deltate dianion $C_3(NCy)_3^{2-}$ in $[(BDI^*)Ca]_2(C_3(NCy)_3)(THP)$ (**2**).

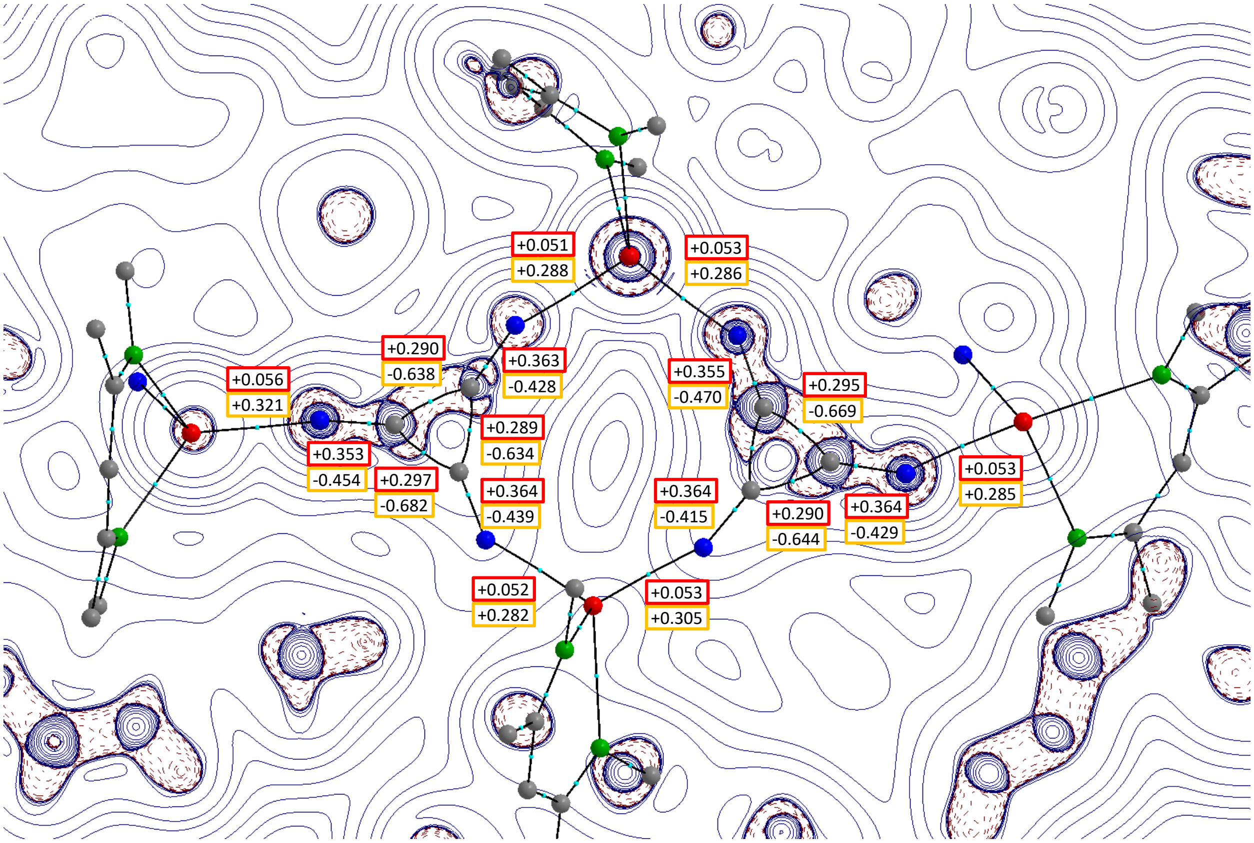

Figure S55. Contour plots of the Laplacian of $[(BDI^*)Ca]_4(THP)_3(C_3O_3)_2$ (**1**) showing areas of electron density concentration (dashed lines) and depletion (solid lines). The BCP's are shown in blue. The electron density $\rho(\mathbf{r})$ in e·B^{-3} (red box) and the Laplacian $\nabla^2\rho(\mathbf{r})$ in e·B^{-5} (yellow box) in the BCPs (blue) are given.

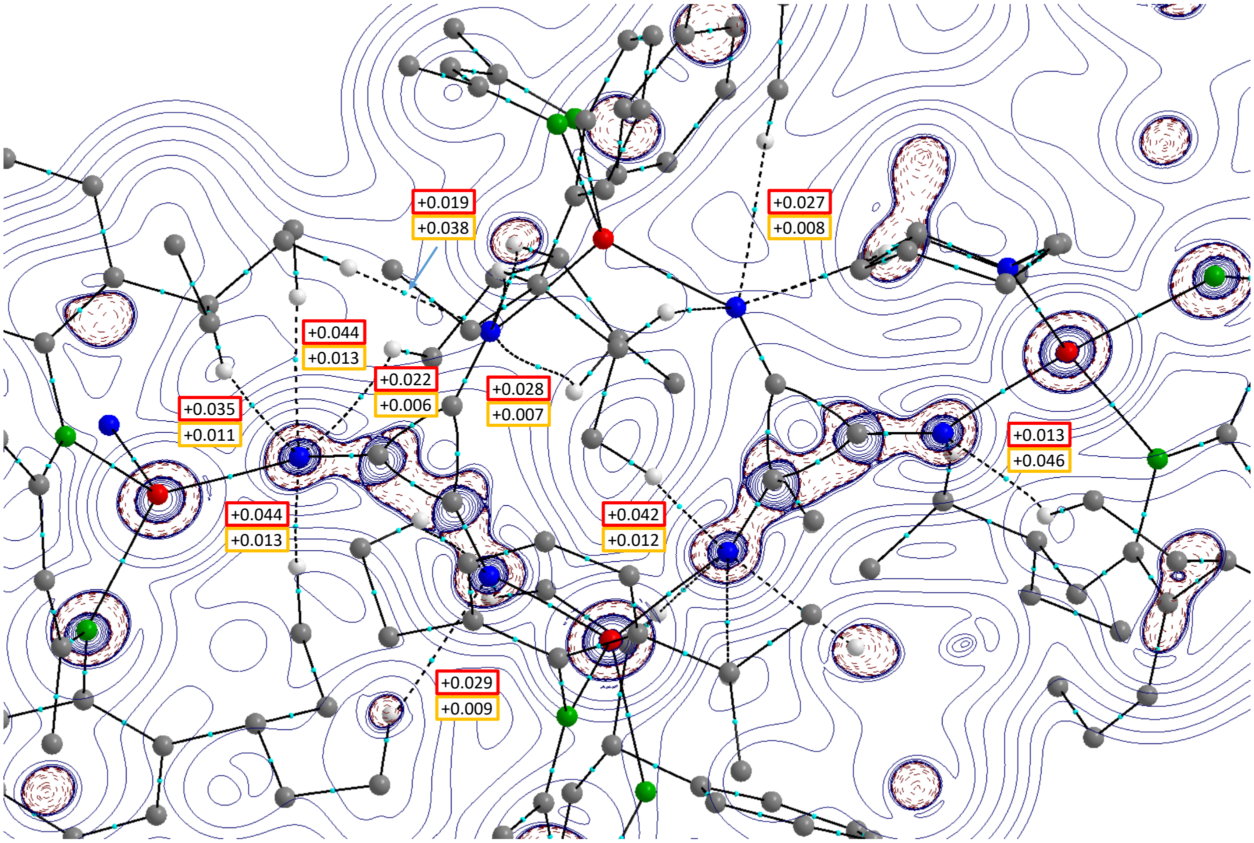

Figure S56. Weak O···H–C bonding interactions of the $C_3O_3^{2-}$ dianions with organic fragments of the BDI* ligand are displayed in the contour plots of the Laplacian of [(BDI*)Ca]$_4$(THP)$_3$(C_3O_3)$_2$ (**1**) showing areas of electron density concentration (dashed lines) and depletion (solid lines). The BCP's are shown in blue. The electron density $\rho(\mathbf{r})$ in e·B^{-3} (red box) and the Laplacian $\nabla^2\rho(\mathbf{r})$ in e·B^{-5} (yellow box) in the BCPs (blue) are given.

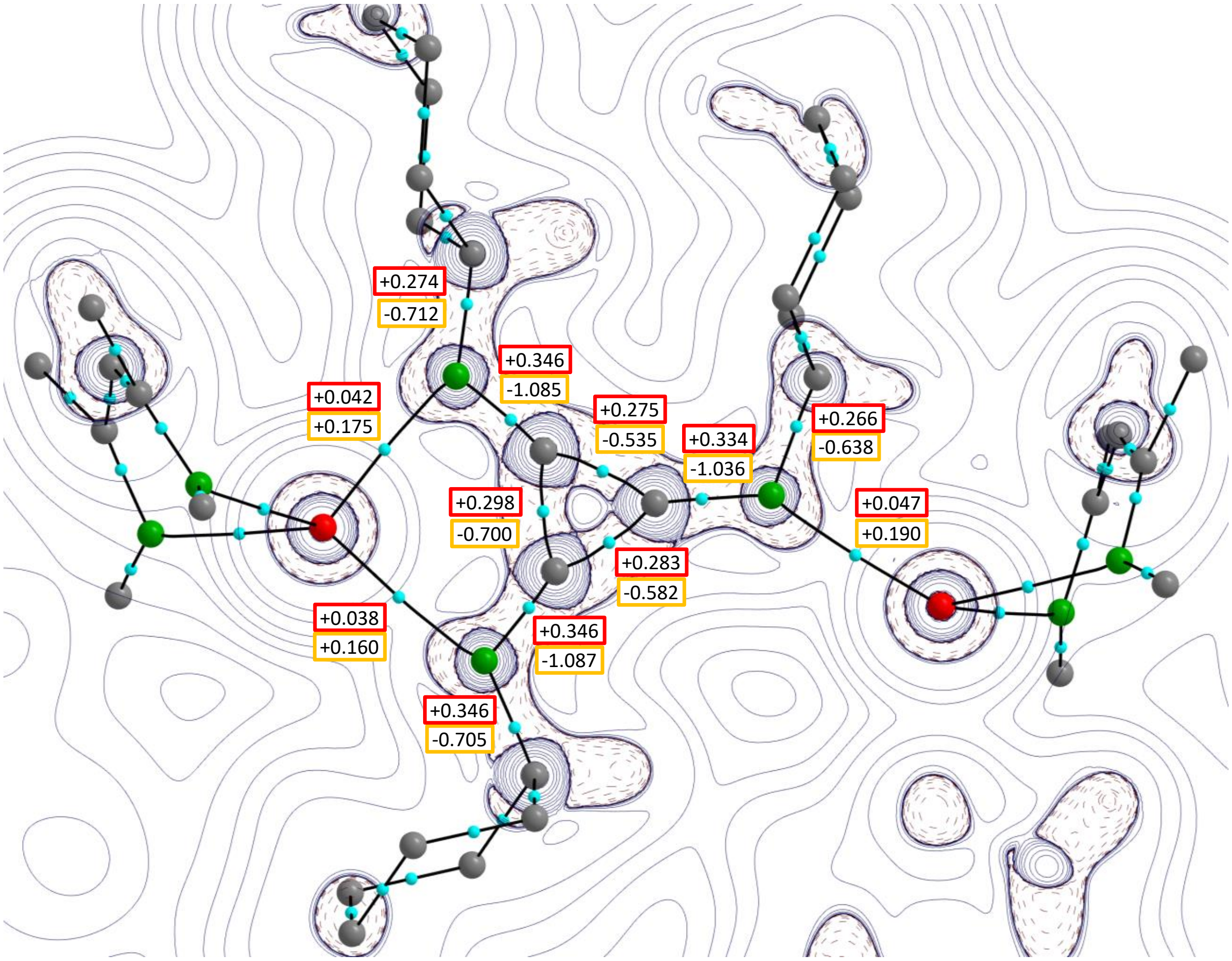

Figure S57. Contour plots of the Laplacian of **2** showing areas of electron density concentration (dashed lines) and depletion (solid lines). The BCP's are shown in blue. The electron density $\rho(\mathbf{r})$ in e·B^{-3} (red box) and the Laplacian $\nabla^2\rho(\mathbf{r})$ in e·B^{-5} (yellow box) in the BCPs (blue) are given.

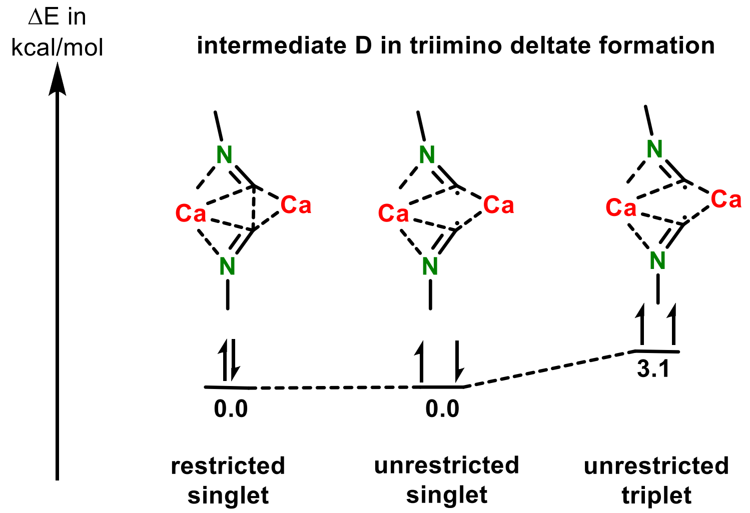

Figure S58. Energy difference ΔE of intermediate **D** in triimino deltate formation for restricted singlet (left), unrestricted singlet (middle) and unrestricted triplet configuration (right). Energy ΔE given in kcal mol^{-1}.

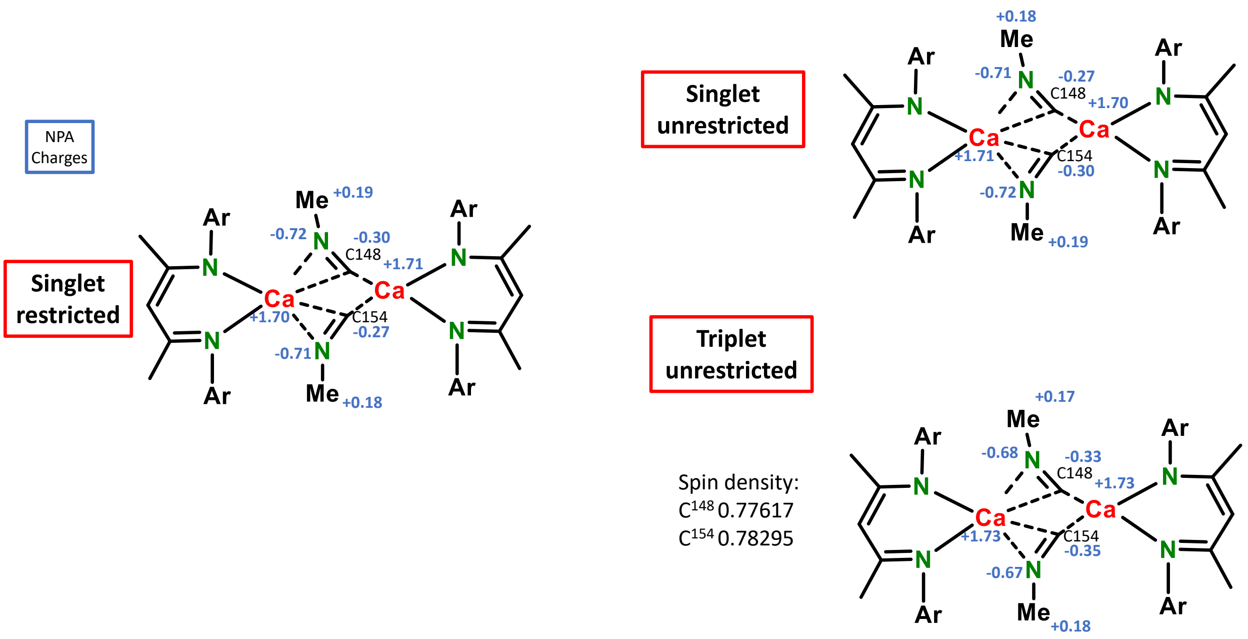

Figure S59. Comparison of NPA charges in intermediate **D** calculated as restricted singlet, unrestricted singlet or as triplet.

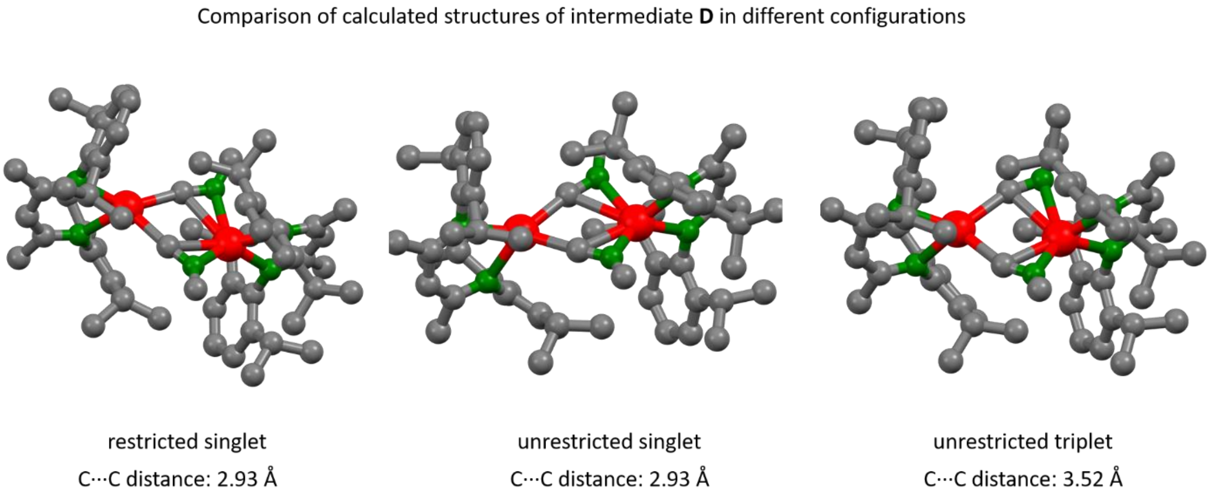

Figure S60. Comparison of calculated structures of intermediate **D** calculated as restricted singlet (left), unrestricted singlet (middle) and unrestricted triplet (right).

Table S2. Selected MO's for intermediate **D** calculated a restricted singlet with most prominent orbital contributions, computed at the B3PW91-GD3BJ/def2tzvp//B3PW91-GD3BJ/def2svp level of theory.

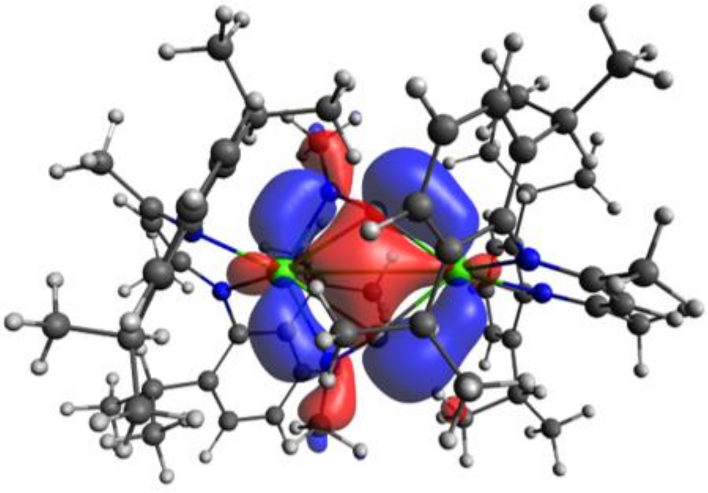<br>HOMO –3.842 eV	C154-p=0.3018<br>C148-p=0.3018<br>Ca2-d=0.0804<br>Ca2-s=0.0256<br>Ca1-d=0.0511<br>Ca1-s=-0.0173<br>Ca1-p=0.0119<br>N147-p=0.0408<br>N153-p=0.0380<br>N153-s=0.0368<br>N147-s=0.0293
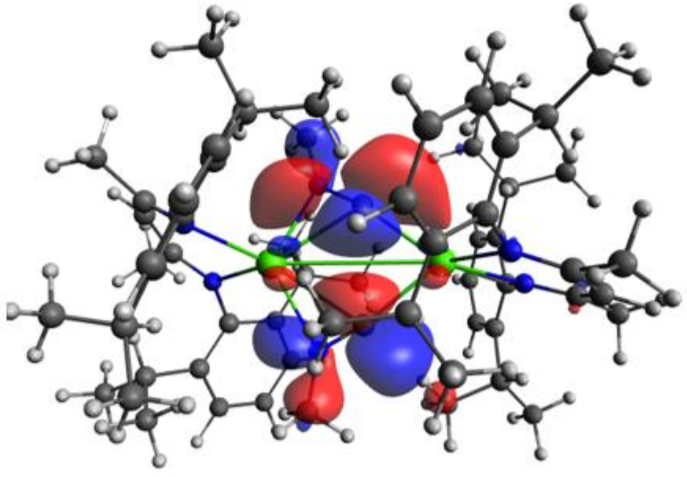<br>LUMO –1.320 eV	C154-p=0.3087<br>C154-s=-0.0284<br>C148-p=0.3044<br>C148-s=-0.0377<br>Ca2-p=0.1274<br>Ca1-p=0.0648<br>Ca1-d=0.0165<br>N153-s=0.0495<br>N153-p=0.0244<br>N153-d=0.0108<br>N147-s=0.0399<br>N147-p=0.0316<br>N147-d=0.0105

Table S3. Most prominent orbital contribution at lone pair for C atoms C148 and C154 for intermediate **D** calculated in singlet restricted state, computed at the B3PW91-GD3BJ/def2tzvp//B3PW91-GD3BJ/def2svp level of theory.

(2.00000) 94.4835% LP(1) C148	(2.00000) 94.4835% LP (1) C148
2.990% Ca1 s(74.33%) p 0.02(1.31%) d 0.33(24.36%)<br>1.247% Ca2 s(96.78%) p 0.01(0.71%) d 0.03(2.51%)<br>0.455% N147 s(49.37%) p 0.99(48.88%) d 0.03(1.27%)<br>94.487% C148 s(67.84%) p 0.47(32.15%) d 0.00(0.01%)	2.724% Ca1 s(67.62%) p 0.03(2.22%) d 0.45(0.16%)<br>1.182% Ca2 s(96.64%) p 0.01(1.04%) d 0.02(2.32%)<br>0.473% N153 s(52.04%) p 0.89(46.25%) d 0.03(1.34%)<br>94.291% C154 s(68.93%) p 0.45(31.06%) d 0.00(0.01%)

Table S4. Selected MO's for intermediate **D** calculated in singlet unrestricted state with most prominent orbital contributions, computed at the B3PW91-GD3BJ/def2tzvp//B3PW91-GD3BJ/def2svp level of theory.

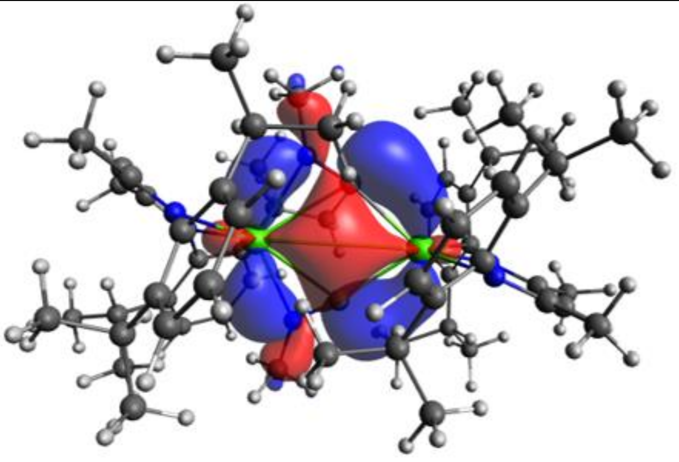<br>HOMO –3.842 eV	Alpha<br><br>C154-p=0.3019<br>C148-p=0.3018<br>Ca2-d=0.0804<br>Ca2-s=0.0256<br>Ca1-d=0.0511<br>Ca1-p=0.0119<br>Ca1-s=-0.0172<br>N147-p=0.0408<br>N147-s=0.0293<br>N153-p=0.0380<br>N153-s=0.0368	Beta<br><br>C154-p=0.3019<br>C148-p=0.3018<br>Ca2-d=0.0804<br>Ca2-s=0.0256<br>Ca1-d=0.0511<br>Ca1-p=0.0119<br>Ca1-s=-0.0172<br>N147-p=0.0408<br>N147-s=0.0293<br>N153-p=0.0380<br>N153-s=0.0368
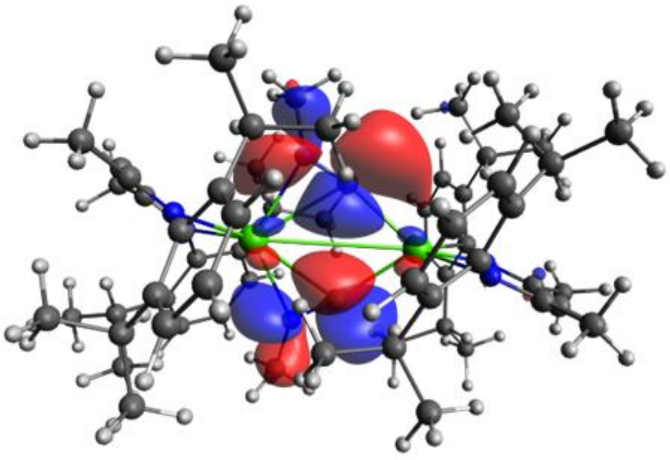<br>LUMO –1.320 eV	Alpha<br><br>C154-p=0.3086<br>C154-s=-0.0284<br>C148-p=0.3043<br>C148-s=-0.0376<br>Ca2-p=0.1274<br>Ca1-p=0.0648<br>Ca1-d=0.0165<br>N153-s=0.0494<br>N153-p=0.0243<br>N153-d=0.0108<br>N147-s=0.0399<br>N147-p=0.0316<br>N147-d=0.0105	Beta<br><br>C154-p=0.3086<br>C154-s=-0.0284<br>C148-p=0.3043<br>C148-s=-0.0376<br>Ca2-p=0.1274<br>Ca1-p=0.0648<br>Ca1-d=0.0165<br>N153-s=0.0494<br>N153-p=0.0243<br>N153-d=0.0108<br>N147-s=0.0399<br>N147-p=0.0316<br>N147-d=0.0105

Table S5. Most prominent orbital contribution at lone pair for C atoms C148 and C154 for intermediate **D** calculated in singlet unrestricted state, computed at the B3PW91-GD3BJ/def2tzvp//B3PW91-GD3BJ/def2svp level of theory.

**Alpha (1.00000) 94.4835% LP(1) C148**	**Beta (1.00000) 94.4835% LP(1) C148**
2.989% Ca1 s(74.33%) p 0.02(1.31%) d 0.33(24.36%)<br>1.247% Ca2 s(96.77%) p 0.01(0.71%) d 0.03(2.51%)<br>0.455% N147 s(49.38%) p 0.99(48.88%) d 0.03(1.27%) f 0.01(0.47%)<br>94.488% C148 s(67.84%) p 0.47(32.15%) d 0.00(0.01%)	2.989% Ca1 s(74.33%) p 0.02(1.31%) d 0.33(24.36%)<br>1.247% Ca2 s(96.77%) p 0.01(0.71%)d 0.03(2.51%)<br>0.455% N147 s(49.38%) p 0.99(48.88%)d 0.03(1.27%)<br>94.488% C148 s(67.84%) p 0.47(32.15%) d 0.00(0.01%)
**Alpha (1.00000) 94.2872% LP(1) C154**	**Beta (1.00000) 94.2872% LP(1) C154**
2.724% Ca1 s(67.62%) p 0.03(2.22%) d 0.45(30.16%)<br>1.182% Ca2 s(96.64%) p 0.01(1.04%) d 0.02(2.32%)<br>0.473% N153 s(52.04%) p 0.89(46.25%) d 0.03(1.34%)<br>94.290% C154 s(68.93%) p 0.45(31.06%) d 0.00(0.01%)	2.724% Ca1 s(67.62%) p 0.03(2.22%) d 0.45(30.16%)<br>1.182% Ca2 s(96.64%) p 0.01(1.04%) d 0.02(2.32%)<br>0.473% N153 s(52.04%) p 0.89(46.25%) d 0.03(1.34%)<br>94.290% C154 s(68.93%) p 0.45(31.06%) d 0.00(0.01%)

Table S6. Selected MO's for intermediate **D** calculated in triplet unrestricted state with most prominent orbital contributions, computed at the B3PW91-GD3BJ/def2tzvp//B3PW91-GD3BJ/def2svp level of theory.

	Alpha	Beta
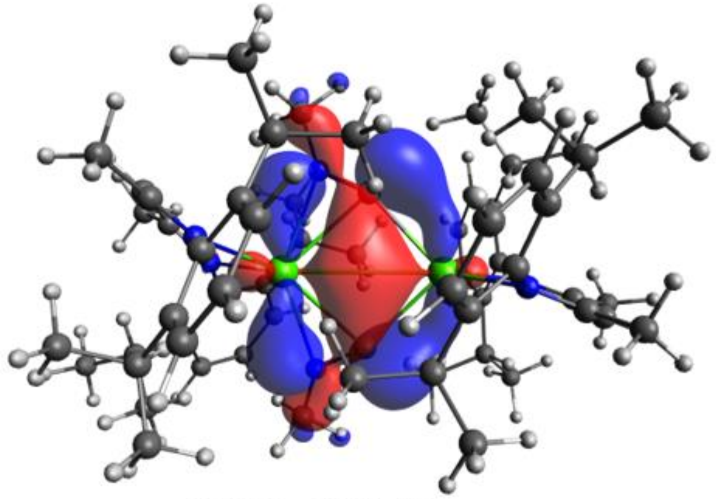<br>SOMO –4.588 eV	C148-p=0.3419<br>C148-s=-0.0355<br>C154-p=0.2920<br>C154-s=-0.0265<br>Ca2-p=0.0433<br>Ca2-d=0.0412<br>Ca1-p =0.0196<br>Ca1-d=0.0110<br>C149-p=0.0241<br>C155-p=0.0227<br>N147-p=0.0585<br>N147-s=0.0519<br>N153-p=0.0461<br>N153-s=0.0460	C18-p=0.3739<br>N4-p=0.2198<br>N6-p=0.1783<br>C16-d=0.0208<br>C22-p=0.0184<br>C22-d=0.0175<br>C48-p=0.0144<br>Ca2-d=0.0131<br>C34-p=0.0126<br>C16-p=0.0107
	Alpha	Beta
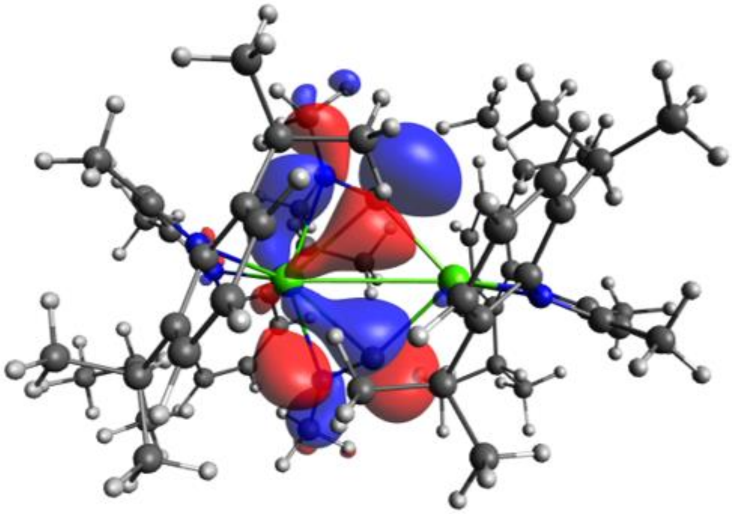<br>LUMO –3.741 eV	C15-p=0.1794<br>C21-p=0.1766<br>N3-p=0.0884<br>Ca1-d=0.0865<br>N5-p=0.0725<br>C148-p=0.0353<br>C89-p=0.0294<br>C47-p=0.0293<br>C81-p=0.0229<br>N147-p=0.0198<br>C35-p=0.0164<br>C17-d=0.0154	C154-p=0.2363<br>C148-p=0.2143<br>Ca1-d=0.1142<br>Ca2-d=0.0973<br>Ca1-p=0.0483<br>Ca2-p=0.0474<br>N153-p=0.0320<br>N147-p=0.0292<br>Ca2-s=0.0227<br>N153-s=0.0220<br>N147-s=0.0203

Table S7. Most prominent orbital contribution at lone pair for C atoms C148 and C154 for intermediate **D** calculated in triplet unrestricted state, computed at the B3PW91-D3BJ/def2tzvp//B3PW91-GD3BJ/def2svp level of theory.

**Alpha (1.00000) 95.2393% LP(1) C148**	**Beta (1.00000) 94.2081% LP(1) C148**
2.475% Ca1 s(79.86%) p 0.02(1.25%) d 0.24(18.88%)<br>1.260% Ca2 s(93.36%) p 0.02(2.28%) d 0.05(4.36%)<br>0.314% N147 s(60.38%) p 0.58(34.81%) d 0.07(3.93%)<br>95.242% C148 s(63.58%) p 0.57(36.40%) d 0.00(0.02%)	2.856% Ca1 s(73.69%) p 0.01(1.05%) d 0.34(25.26%)<br>1.222% Ca2 s(93.35%) p 0.01(0.78%) d 0.06(5.87%)<br>0.563% N147 s(36.47%) p 1.72(62.66%) d 0.01(0.54%)<br>94.213% C148 s(65.43%) p 0.53(34.54%) d 0.00(0.02%)
**Alpha (1.00000) 89.9196% LP(2) C148**	**Beta (1.00000) 94.0540% LP(1) C154**
1.266% Ca1 s(10.66%) p 0.46(4.92%) d 7.92(84.42%)<br>1.980% Ca2 s(1.78%) p 4.06(7.23%) d51.07(90.99%)<br>2.688% N147 s(22.95%) p 3.24(74.27%) d 0.12(2.72%)<br>89.922% C148 s(4.56%) p 20.92(95.32%) d 0.03(0.12%)<br>2.421% C149 s(40.93%) p 1.43(58.66%) d 0.01(0.22%)	2.674% Ca1 s(70.13%) p 0.02(1.52%) d 0.40(28.35%)<br>1.255% Ca2 s(92.62%) p 0.01(0.81%) d 0.07(6.57%)<br>0.518% N153 s(37.85%) p 1.62(1.16%) d 0.02(0.70%)<br>94.059% C154 s(65.92%) p 0.52(34.05%) d 0.00(0.02%)<br>0.593% C155 s(18.18%) p 4.49(81.71%) d 0.01(0.10%)
**Alpha (1.00000) 94.7401% LP(1) C154**	**Alpha (1.00000) 90.4345% LP(2) C154**
2.551% Ca1 s(77.76%) p 0.02(1.50%) d 0.27(20.73%)<br>1.395% Ca2 s(96.79%) p 0.02(1.77%) d 0.01(1.44%)<br>0.328% N153 s(51.65%) p 0.87(45.02%) d 0.05(2.60%)<br>94.742% C154 s(67.01%) p 0.49(32.97%) d 0.00(0.02%)<br>0.210% C155 s(9.70%) p 9.24(89.71%) d 0.05(0.50%)	1.037% Ca1 s(0.55%) p 13.30(7.33%) d99.99(2.12%)<br>2.148% Ca2 s(2.45%) p 4.40(10.77%) d 35.44(86.78%)<br>2.645% N153 s(21.24%) p 3.57(75.73%) d 0.14(2.96%)<br>90.436% C154 s(1.55%) p 63.38(98.33%) d 0.07(0.11%)<br>2.181% C155 s(40.63%) p 1.45(58.99%) d 0.00(0.19%)

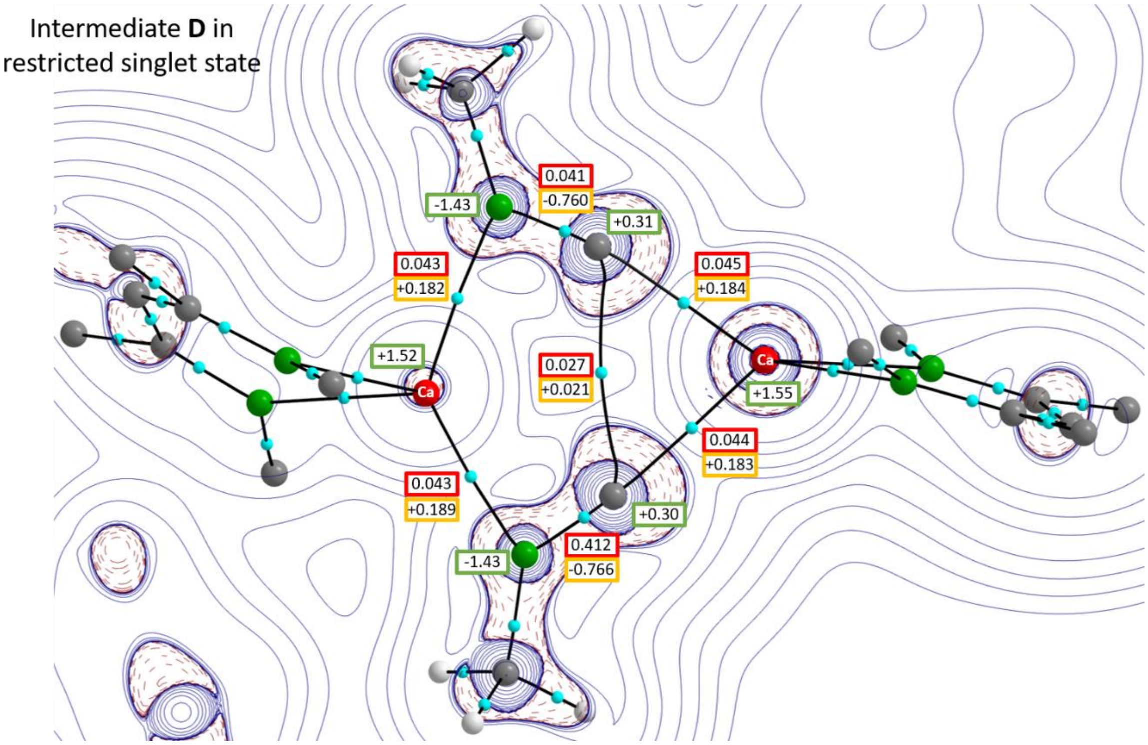

Figure S61. Contour plot of the Laplacian for intermediate **D** in its restricted singlet state showing areas of electron density concentration (dashed lines) and depletion (solid lines). The BCP's are shown in blue. The electron density $\rho(\mathbf{r})$ in e·B^{-3} (red box), the Laplacian of the electron density $\nabla^2\rho(\mathbf{r})$ in e·B^{-5} (yellow box) in the BCPs (blue) and the AIM charges for selected atoms (green boxes) are given.

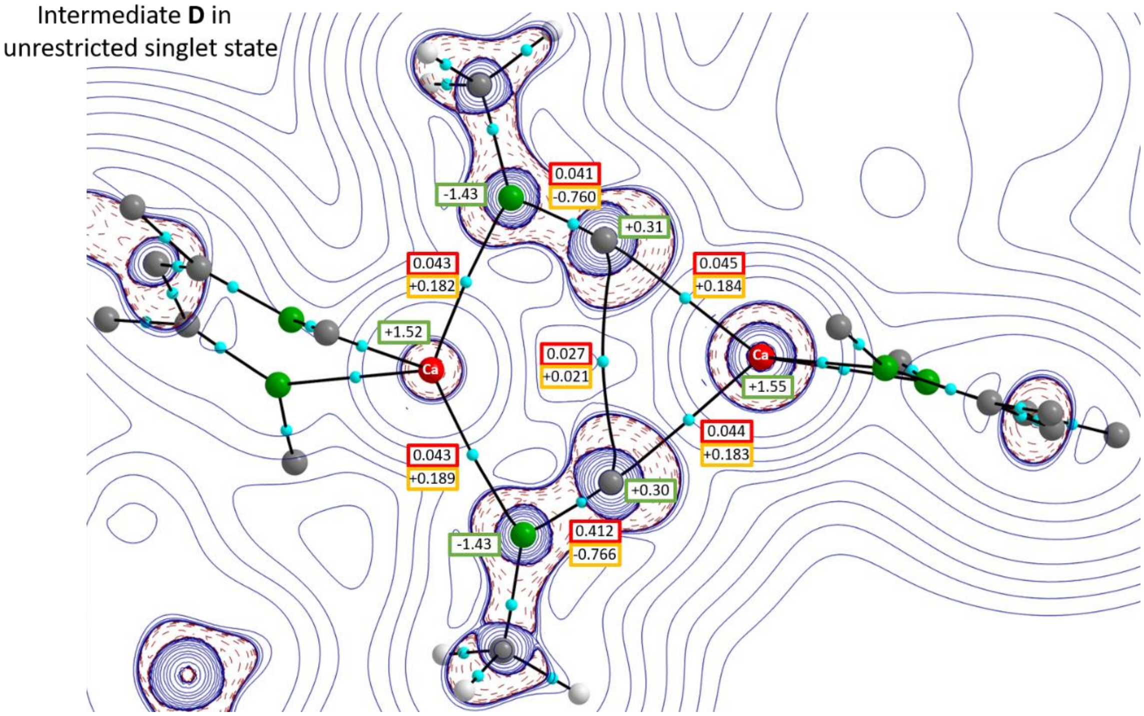

Figure S62. Contour plot of the Laplacian for intermediate **D** in its unrestricted singlet state showing areas of electron density concentration (dashed lines) and depletion (solid lines). The BCP's are shown in blue. The electron density $\rho(\mathbf{r})$ in $e{\cdot}B^{-3}$ (red box), the Laplacian of the electron density $\nabla^2\rho(\mathbf{r})$ in $e{\cdot}B^{-5}$ (yellow box) in the BCPs (blue) and the AIM charges for selected atoms (green boxes) are given. Notably, there is no difference to the restricted singlet state calculation (see **Figure S55**).

Intermediate **D** in unrestricted triplet state

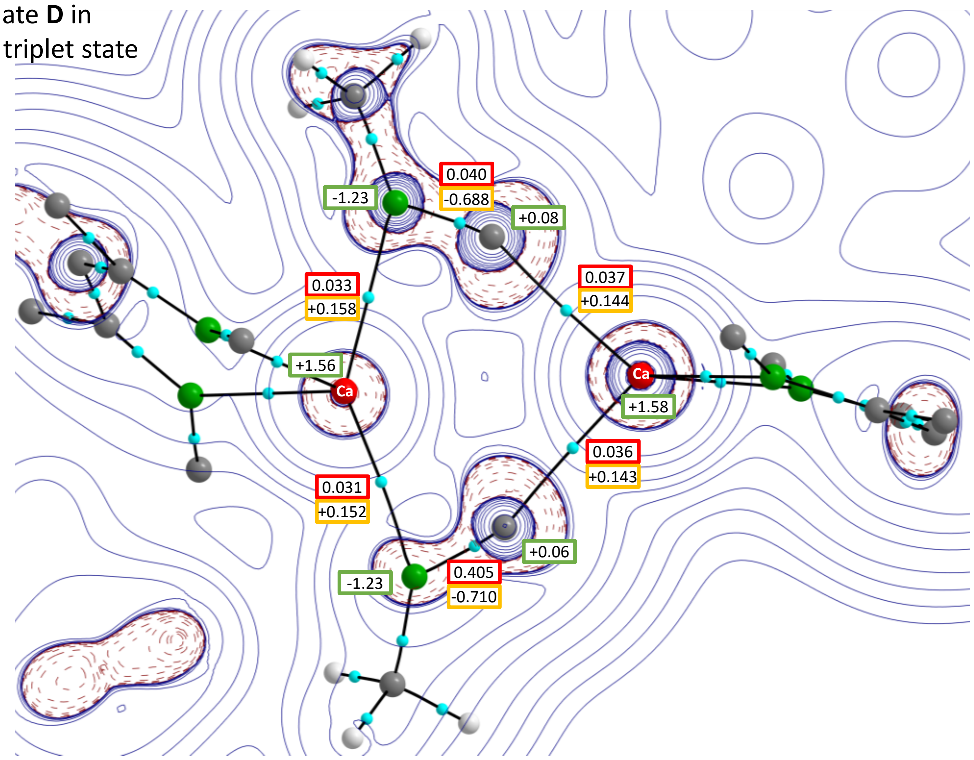

Figure S63. Contour plot of the Laplacian for intermediate **D** in its unrestricted triplet state showing areas of electron density concentration (dashed lines) and depletion (solid lines). The BCP's are shown in blue. The electron density $\rho(\mathbf{r})$ in e·B^{-3} (red box), the Laplacian of the electron density $\nabla^2\rho(\mathbf{r})$ in e·B^{-5} (yellow box) in the BCPs (blue) and the AIM charges for selected atoms (green boxes) are given.

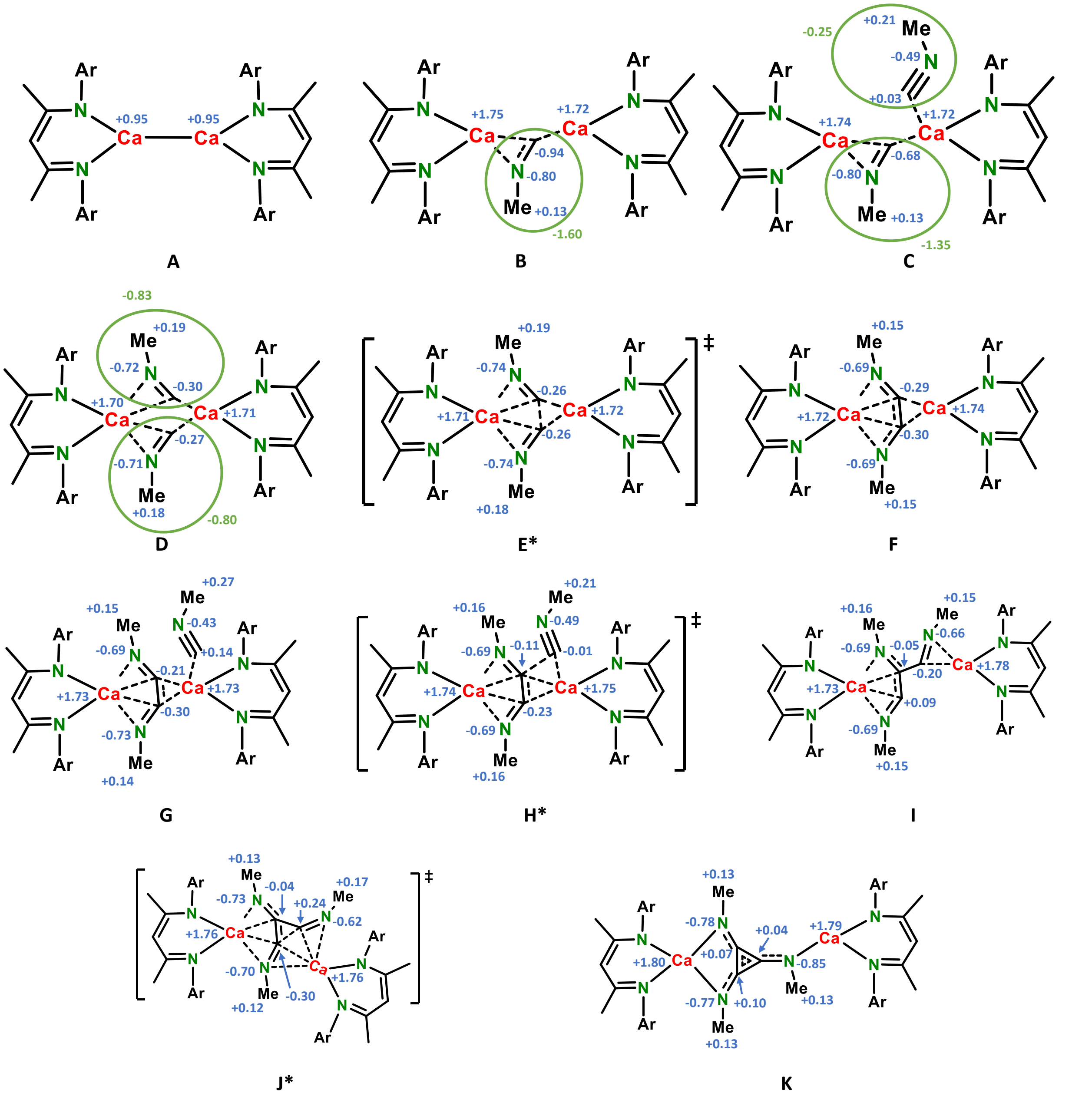

Figure S64. Calculated NPA charges for intermediates **A**-**K** on the pathway for reductive trimerization of Me-NC with (^{DIPP}BDI)Ca-Ca(^{DIPP}BDI) in restricted singlet state configuration. NPA charges for selected fragments are given in green.

XYZ Coordinates

448

[(BDI*)Ca]4(THP)3(C3O3)2 (**1**)

C	-9.381523	-4.651613	-2.955848
C	-10.349837	-3.749997	-2.518810
C	-11.920313	-2.238376	0.021730
C	-12.461134	-1.118163	0.899801
C	-8.073939	-4.524888	-2.501483
C	-11.998760	-1.281365	-2.315845
C	-10.037182	-2.736655	-1.609708
C	-11.084126	-1.732554	-1.170449
C	-9.030729	-3.206409	1.654754
C	-11.268941	-0.663786	-3.497623
C	-1.523360	-7.674671	0.022095
C	-7.713820	-3.531964	-1.581474
C	-2.611223	-6.652098	-3.453859
C	-5.936435	-4.579846	-0.147401
C	-8.714180	-2.659118	-1.097493
C	-4.484853	-4.629464	0.305329
C	-6.271651	-3.439581	-1.124022
C	2.293620	-8.983756	0.856537
C	-8.693930	-1.826017	1.133635
C	-5.849558	-3.808932	3.916952
C	-1.981686	-6.283814	2.021735
C	-1.350954	-6.368922	0.765205
C	-2.442912	-5.657614	4.373713
C	-2.074720	-5.255865	-3.724322
C	-5.296586	-3.383758	-2.305381
C	1.650823	-7.812717	1.585905
C	-8.682880	-0.808219	2.109606
C	-1.892031	-5.285729	3.015426
C	-5.576769	-2.252943	-3.282750
C	0.862961	-5.880253	-3.170802
C	-4.829245	-2.901999	4.592575
C	-0.042820	-5.472793	-2.185149
C	2.056274	-6.511414	-2.835325
C	-11.968401	3.338165	2.416224
C	0.261267	-5.738280	-0.831879
C	-11.989921	3.074534	0.918101
C	-1.262968	-4.659050	-2.570175
C	-10.877615	1.979738	-1.089288
C	2.386079	-6.687094	-1.493323
C	1.516930	-6.292532	-0.473642
C	1.914718	-6.417032	0.987397
C	-8.534865	0.592381	1.991597
C	-10.716571	2.417522	0.377729
C	-8.001568	0.632323	-4.109382
C	-8.900087	1.374120	3.234443
C	-11.187269	3.084977	-2.087951
C	-3.665443	-2.484385	3.665813
C	1.170440	-5.468416	6.042112
C	-5.151755	-1.063268	2.094372
C	1.203053	-5.310304	4.528833
C	-0.796471	-3.224218	-2.880754
C	-6.830466	0.901725	-5.033086
C	3.352057	-5.948514	1.251241
C	-1.227025	-3.215456	3.941947
C	-2.330829	-2.446786	4.384586
C	-1.898738	-2.234998	-3.212962
C	-3.919987	-1.129010	2.982410
C	-9.460336	3.251542	0.540226
C	-8.208269	2.632584	0.794078
C	1.232996	-3.853300	4.024633
C	0.048083	-3.055294	4.541858
C	-2.161355	-1.600667	5.485624
C	-7.656882	2.681532	-2.922979
C	5.878189	-5.742982	-1.411113
C	-2.763795	-0.704576	-0.251194
C	-6.608623	2.405390	-5.180509
C	-3.765521	0.160807	-0.715296
C	3.615207	-4.511880	0.835402
C	-9.500594	4.637884	0.364505
C	5.392207	-4.916581	-2.419028
C	0.169857	-2.186601	5.630359
C	-0.926598	-1.476636	6.115311
C	-6.479151	3.049345	-3.802756
C	-7.024222	3.409387	0.850708
C	2.601296	-3.212415	4.280364
C	6.845445	-5.261963	-0.539780
C	-5.571209	2.524430	2.711603
C	2.921542	-2.739108	-2.753135
C	-5.691213	2.772847	1.195571
C	6.146183	-4.290532	-5.641564
C	4.988779	-3.537548	-5.003503
C	-2.527706	0.690397	-0.298519
C	-3.043233	1.419173	-4.054669
C	-8.342859	5.409505	0.422983
C	-5.802995	3.749026	3.584023
C	5.884288	-3.621473	-2.606362
C	-7.119886	4.793608	0.663547
C	-0.312697	-0.116237	2.517390
C	2.809917	-1.856004	3.624712
C	5.328675	-2.759158	-3.724626
C	1.997356	-1.182424	0.114928
C	7.362041	-3.969959	-0.678749
C	4.111568	-1.938960	-3.254523
C	-4.470905	3.516974	0.646892
C	-4.447780	3.643185	-0.867309
C	6.897035	-3.151260	-1.733465
C	-1.947328	2.416652	-4.398688
C	0.415740	0.438245	-4.126005
C	9.664747	-4.376559	0.303504
C	-1.622291	1.773515	4.535317
C	10.362458	-4.434743	-1.045368
C	-0.184950	1.211779	-2.961689
C	7.821155	-3.370405	1.716412
C	8.412987	-3.491816	0.305814

C	-2.559999	2.848373	5.056706
C	3.324929	-0.724974	-0.034236
C	0.343206	1.137345	3.065153
C	2.262639	0.196139	-0.008345
C	6.771877	-2.279225	1.834091
C	-0.876293	2.549326	-3.310575
C	8.804127	-2.729035	-3.764083
C	-0.625346	2.258556	3.479169
C	8.459771	-1.679374	-2.728959
C	0.150018	3.619641	-3.617465
C	0.187570	3.458484	3.914161
C	3.599294	1.737641	-3.368391
C	0.797002	3.661278	-4.858613
C	0.743451	3.487615	5.197722
C	4.049869	1.666811	2.747956
C	5.645486	1.157324	-4.693071
C	4.326115	1.927509	-4.695082
C	-1.653121	5.610795	0.182365
C	-1.104546	5.387921	-1.103059
C	-2.028344	5.756752	2.623021
C	-1.175955	5.274697	1.469474
C	9.300696	-0.538456	-2.775548
C	-1.723621	6.157392	-2.245409
C	4.517506	2.032577	-2.205813
C	0.513242	4.567199	-2.628718
C	4.893517	1.825248	4.007921
C	0.447645	4.526106	3.025398
C	6.228245	1.104529	3.836015
C	4.848035	2.039353	1.517727
C	6.468376	1.504438	-3.466499
C	9.479504	-0.688068	3.934068
C	1.815948	4.574940	-5.114051
C	10.587535	-0.967927	2.928871
C	1.519681	4.560541	5.623271
C	6.914341	1.533447	2.552430
C	10.341176	-0.471196	1.498253
C	11.461305	-0.966767	0.559855
C	9.575586	0.446245	-1.806875
C	1.559174	5.489614	-2.876070
C	3.003465	4.469067	-0.443679
C	1.305664	5.583230	3.425313
C	2.203665	5.465782	-4.116871
C	1.795758	5.596474	4.734845
C	10.117406	1.025380	1.375449
C	9.403233	1.554580	0.268053
C	12.810139	-0.287310	0.748038
C	10.791005	1.301822	-2.101836
C	2.041539	6.425811	-1.784171
C	1.709092	6.661547	2.438800
C	3.266973	5.817074	-1.085903
C	7.041191	3.968328	-0.690066
C	0.771304	7.880893	2.484414
C	4.187919	5.960075	2.645037
C	10.639294	1.907966	2.326542

C	9.211605	2.953763	0.152276
C	8.480688	3.547261	-1.034347
C	1.182800	8.568584	-2.910280
C	2.355212	7.844894	-2.266944
C	3.174321	7.094558	2.603235
C	6.881226	5.131207	0.274508
C	0.852750	8.771958	1.252793
C	10.453845	3.283031	2.218675
C	9.744437	3.794344	1.137198
C	8.736385	5.091321	-3.062636
C	9.286320	4.669590	-1.707033
H	-9.644406	-5.441800	-3.663200
H	-12.745681	-2.863150	-0.363570
H	-11.369916	-3.830118	-2.901379
H	-12.612157	-2.133174	-2.656982
H	-13.088679	-1.511447	1.714378
H	-11.310041	-2.909829	0.638805
H	-13.074812	-0.403082	0.329554
H	-7.308769	-5.214777	-2.865434
H	-9.312481	-3.899787	0.853818
H	-12.722178	-0.554244	-1.910947
H	-9.832535	-3.167831	2.404165
H	-1.994793	-8.441864	0.648436
H	-6.213907	-5.539355	-0.618101
H	-3.182570	-7.036881	-4.312769
H	-11.972211	-0.300994	-4.262920
H	-6.597835	-4.479964	0.727159
H	-10.596164	-1.391587	-3.975631
H	-2.156464	-7.504357	-0.861309
H	-1.795703	-7.361375	-3.245749
H	-11.629768	-0.558162	1.357149
H	-8.141294	-3.620170	2.154131
H	-3.285633	-6.650937	-2.582263
H	-0.565902	-8.057825	-0.355582
H	-2.527327	-7.178640	2.320941
H	2.090824	-9.930908	1.380382
H	-4.348538	-5.326083	1.143148
H	-10.524898	-0.851463	-0.815407
H	-2.708748	-6.720459	4.421491
H	-6.687501	-4.051599	4.589148
H	1.907969	-9.081688	-0.169902
H	-5.387135	-4.757059	3.600571
H	-5.299667	-4.352932	-2.833340
H	-10.658035	0.189696	-3.171669
H	0.564273	-7.972110	1.650318
H	-3.813790	-4.969941	-0.496591
H	-6.265096	-3.339499	3.015709
H	-8.930522	-1.149367	3.116524
H	-1.460909	-5.259858	-4.642383
H	3.387843	-8.875759	0.787653
H	-12.837589	2.411782	0.672435
H	-3.335844	-5.063799	4.611751
H	-1.709464	-5.437443	5.162558
H	-2.919026	-4.582091	-3.938434

H	-11.675633	1.222725	-1.129247
H	-6.152127	-2.499291	-0.556905
H	-4.117641	-3.642000	0.621011
H	-6.603351	-2.309296	-3.678533
H	2.003312	-7.793598	2.631369
H	-11.893904	2.397411	2.983285
H	-12.884566	3.850921	2.746816
H	-4.281793	-3.266066	-1.901465
H	0.628854	-5.695174	-4.222256
H	-4.424319	-3.414867	5.480477
H	-8.139727	-0.440124	-3.910314
H	2.741641	-6.844004	-3.618890
H	-1.927399	-4.618351	-1.689919
H	-10.572884	1.485950	0.942952
H	-4.888850	-2.282096	-4.140192
H	1.142525	-6.531974	6.325039
H	3.346691	-7.129063	-1.229336
H	-3.581436	-3.236943	2.867995
H	0.339464	-5.822978	4.080912
H	-9.954528	1.460891	-1.391236
H	-9.984443	1.322014	3.411425
H	-5.330944	-1.998569	4.982097
H	-8.408255	0.932435	4.113296
H	-8.943081	1.015308	-4.542180
H	-5.110335	-1.826876	1.304274
H	-11.222590	2.683264	-3.112634
H	-6.090101	-1.195359	2.650078
H	-5.422702	-1.276442	-2.794109
H	-12.194005	4.015766	0.381827
H	-12.163670	3.553824	-1.892360
H	1.254001	-5.730969	1.540228
H	-11.109799	3.965948	2.700443
H	2.090514	-5.822572	4.122234
H	-7.024397	0.424469	-6.006831
H	0.284718	-4.981107	6.478831
H	-2.391912	-2.464690	-4.168761
H	4.078436	-6.616250	0.758273
H	-2.660426	-2.213494	-2.423982
H	3.540714	-6.064049	2.333061
H	-5.929454	0.426355	-4.612926
H	-0.067774	-3.268390	-3.707983
H	-8.618331	2.430928	3.160822
H	-5.207833	-0.082192	1.603526
H	2.056335	-5.024221	6.522789
H	-10.427521	3.880639	-2.063368
H	5.497774	-6.760124	-1.302486
H	-0.210442	-2.849356	-2.023660
H	1.115379	-3.921801	2.929297
H	-8.599278	3.098029	-3.318254
H	-3.992954	-0.350321	3.761403
H	-3.036059	-0.876227	2.381301
H	-3.012416	-1.016938	5.846012
H	4.611987	-5.289753	-3.081805
H	-1.488324	-1.222062	-3.291003

H	-7.461929	2.851383	-5.722915
H	-6.277957	1.732576	2.999089
H	-10.459265	5.126316	0.180227
H	3.537873	-4.390468	-0.252188
H	-7.538007	3.045620	-1.891182
H	4.160975	-4.237526	-4.804253
H	2.545607	-3.457668	-3.495850
H	2.887480	-3.820985	1.283069
H	-5.666612	1.775158	0.728308
H	7.215604	-5.903928	0.263718
H	5.816920	-4.836781	-6.539156
H	3.361378	-3.913183	3.896952
H	-5.711976	2.608045	-5.786053
H	3.164570	-3.302180	-1.843255
H	4.624728	-4.188176	1.121848
H	6.582175	-5.024190	-4.946314
H	-3.832578	1.423985	-4.820234
H	-2.662561	0.390718	-3.990464
H	-5.553836	2.706409	-3.317703
H	2.793677	-3.140124	5.364450
H	1.140316	-2.067280	6.115431
H	-0.812757	-0.814298	6.976758
H	-4.567673	2.109492	2.902115
H	2.089737	-2.070371	-2.493983
H	-3.507756	1.645563	-3.084181
H	-0.920624	-0.612964	3.281902
H	4.580696	-2.812087	-5.728606
H	-6.819284	4.150538	3.451325
H	-6.416541	4.146499	-3.873375
H	-5.673827	3.504460	4.648644
H	-4.597626	2.655895	-1.326159
H	0.453898	-0.826112	2.182716
H	-0.956675	0.118816	1.658427
H	2.670808	-1.917975	2.536108
H	9.399458	-5.395447	0.635921
H	-2.210780	0.955690	4.095518
H	-0.348437	0.137416	-4.858912
H	6.951300	-3.605664	-5.948089
H	7.380172	-4.338081	2.010038
H	-0.911095	0.574259	-2.434188
H	-8.396195	6.492132	0.286102
H	2.106661	-1.102704	4.006559
H	9.701624	-4.865975	-1.812088
H	0.901593	-0.479977	-3.765054
H	-3.571819	2.962633	0.956854
H	-5.233068	4.317595	-1.241826
H	3.794029	-1.306770	-4.101923
H	-3.289083	2.430088	5.767748
H	11.275817	-5.047730	-1.002705
H	-5.100119	4.562665	3.347062
H	-1.487220	2.138767	-5.361734
H	-6.215259	5.400507	0.722274
H	3.829622	-1.483792	3.805278
H	-1.073896	1.308134	5.370856

H	8.251032	-3.662046	-3.618091
H	4.437060	-1.256268	-2.458260
H	6.108488	-2.025768	-3.985929
H	5.965887	-2.387251	1.092902
H	-2.391335	3.413626	-4.561165
H	-4.377423	4.511467	1.112707
H	-3.477054	4.028101	-1.205452
H	-3.129005	3.306200	4.235536
H	6.292632	-2.267965	2.823852
H	10.360130	-3.987059	1.066962
H	0.961465	0.882491	3.942357
H	1.176141	1.024284	-4.659904
H	-1.404926	2.858081	-2.395738
H	8.638211	-3.181433	2.432243
H	0.616367	1.403738	-2.227958
H	9.880546	-2.947418	-3.749557
H	-1.220860	2.552872	2.596334
H	10.647354	-3.425214	-1.381387
H	1.068703	1.528371	2.330078
H	8.729916	-2.486895	-0.016102
H	-2.013174	3.655791	5.566095
H	8.565082	-2.339301	-4.765759
H	3.228726	0.707207	-3.274805
H	7.238816	-1.285554	1.715196
H	3.700769	0.628281	2.641681
H	5.453380	0.073246	-4.692630
H	0.497999	2.962310	-5.641575
H	0.544414	2.659778	5.882558
H	-2.706289	4.937736	2.911329
H	3.687469	1.611745	-5.534880
H	6.063875	0.014948	3.799025
H	-2.033176	5.468076	-3.045924
H	2.726551	2.404363	-3.300853
H	-2.558618	6.217619	0.185607
H	4.026816	1.780155	-1.259989
H	4.350919	1.446854	4.887994
H	3.155289	2.306117	2.789002
H	-2.590051	6.745234	-1.918889
H	8.517491	-1.097796	3.595403
H	4.270477	1.828383	0.612649
H	10.746279	-2.057700	2.865445
H	6.241206	1.379196	-5.593306
H	9.996872	-0.540745	-3.615595
H	-2.642252	6.623575	2.347597
H	9.418256	-0.951536	1.134415
H	11.571050	-2.053162	0.712883
H	9.706907	-1.146148	4.908811
H	-1.432775	5.996227	3.512497
H	7.381069	0.893789	-3.395438
H	4.526208	3.003397	-4.844707
H	6.903860	1.299180	4.683969
H	2.622394	3.745561	-1.175950
H	-0.984577	6.836877	-2.692546
H	7.838328	0.973732	2.372990
H	11.128994	-0.852420	-0.481574
H	4.783845	3.102691	-2.185324
H	2.312762	4.586108	-6.086980
H	1.920913	4.583295	6.639318
H	5.076811	2.899131	4.190167
H	2.264745	4.560072	0.366223
H	5.094192	3.116321	1.533051
H	9.338309	0.389286	4.101377
H	6.767032	2.569273	-3.491647
H	11.540530	-0.564757	3.310941
H	3.918061	4.043026	-0.017562
H	7.189498	2.604110	2.583745
H	1.243113	6.495370	-1.029522
H	11.447666	0.822593	-2.837895
H	13.549055	-0.679341	0.032473
H	6.521673	3.088686	-0.285553
H	1.592169	6.211947	1.439094
H	13.217457	-0.446607	1.758789
H	-0.264052	7.533785	2.591397
H	8.377386	2.740048	-1.772635
H	3.018557	6.167919	-4.309675
H	2.428384	6.426125	5.058965
H	4.050199	5.321166	3.528622
H	4.089263	5.311823	1.765296
H	11.367529	1.512357	-1.191789
H	10.478499	2.275647	-2.509244
H	0.814622	8.026226	-3.795068
H	6.522641	4.214107	-1.630634
H	12.738615	0.800508	0.593895
H	4.083556	5.724615	-1.824972
H	0.343986	8.667587	-2.203475
H	11.211626	1.512322	3.167116
H	3.625119	6.529268	-0.323522
H	0.988061	8.460846	3.399555
H	8.686653	4.234676	-3.754852
H	5.822658	5.402604	0.375177
H	0.576284	8.202407	0.351829
H	5.217425	6.347362	2.664107
H	7.254309	4.885635	1.279478
H	3.209667	7.820181	-2.965973
H	3.272944	7.712336	3.513766
H	7.721887	5.511823	-2.984850
H	1.464591	9.582738	-3.232615
H	2.709561	8.421890	-1.396244
H	3.424668	7.771359	1.770173
H	0.160596	9.624463	1.330909
H	10.327948	4.327977	-1.821969
H	7.412472	6.032140	-0.069681
H	1.861709	9.182844	1.095237
H	10.872607	3.955787	2.970783
H	9.621679	4.874541	1.042623
H	9.372340	5.858143	-3.530766
H	9.342764	5.549108	-1.045016
Ca	-7.096898	0.139053	-0.918386

Ca -0.710344 -3.107464 0.768509
Ca 0.140060 2.742424 0.151253
Ca 6.556130 -0.082036 -0.519398
N -8.401307 -1.650029 -0.153612
N -0.635587 -5.396303 0.206418
N -1.347673 -4.088604 2.838853
N -8.125333 1.223151 0.897544
N 7.472213 -1.857749 -1.867296
N -0.106768 4.551467 -1.359410
N -0.097172 4.531311 1.719717
N 8.872246 0.660121 -0.693952
O -7.808478 1.256366 -2.837902
O -2.384677 -1.877915 0.037269
O -4.958114 0.324476 -1.145475
O 1.172117 -2.128314 0.279767
O -1.766821 1.677973 -0.073309
O 4.569408 -0.938765 -0.127452
O 1.877628 1.412579 -0.053228
O 5.731096 1.276619 -2.265310
O 6.075290 1.311782 1.420316

267
[(BDI*)Ca]2(C3N3Cy3)(THP) (**2**)
N -5.096192 1.676351 0.398875
N -4.903211 -1.407008 0.599168
C -5.666791 2.669684 2.579211
H -4.697917 3.031251 2.963934
H -6.290504 2.399743 3.440666
H -6.124834 3.507500 2.041118
C -5.395842 1.481855 1.682771
C -5.379099 0.228211 2.340072
H -5.572549 0.287448 3.411077
C -5.241147 -1.090090 1.850163
C -5.473227 -2.198459 2.849556
H -6.384148 -2.763721 2.610335
H -5.565039 -1.806977 3.869749
H -4.644830 -2.919191 2.811931
C -5.411264 2.928901 -0.186303
C -4.403404 3.898087 -0.403623
C -4.761450 5.143085 -0.930093
H -3.991079 5.902804 -1.076546
C -6.082945 5.436238 -1.256747
H -6.347130 6.420184 -1.651274
C -7.059238 4.458427 -1.094048
H -8.091228 4.675649 -1.380179
C -6.745229 3.196508 -0.578646
C -5.072419 -2.742411 0.159999
C -3.946794 -3.574594 -0.059667
C -4.149764 -4.876768 -0.534813
H -3.289033 -5.533485 -0.671386
C -5.423891 -5.356580 -0.811215
H -5.562079 -6.374880 -1.181953
C -6.525217 -4.531445 -0.598572
H -7.524875 -4.913303 -0.813110
C -6.380555 -3.231564 -0.106061
C -2.536943 -3.090973 0.216030
H -2.609895 -2.174386 0.822702
C -1.792229 -2.686817 -1.072223
H -2.330915 -1.859791 -1.566997
H -0.815392 -2.272420 -0.777925
C -1.597718 -3.776734 -2.116616
H -2.552692 -4.114454 -2.543838
H -1.097884 -4.661009 -1.697684
H -0.967902 -3.413183 -2.941785
C -1.695157 -4.071356 1.043871
H -1.494957 -4.988014 0.465033
H -0.710666 -3.602002 1.201673
C -2.303008 -4.445030 2.386192
H -3.282032 -4.933254 2.262632
H -2.446821 -3.555107 3.017737
H -1.649817 -5.137873 2.938172
C -7.601172 -2.343709 0.074817
H -7.389128 -1.640649 0.894109
C -8.808601 -3.823374 1.793635
H -9.747547 -4.355162 2.011142
H -8.630074 -3.119742 2.622142
H -7.994788 -4.564783 1.803991
C -2.953906 3.555619 -0.138171
H -2.940921 2.759456 0.623819
C -2.322357 2.945953 -1.406552
H -1.360970 2.483013 -1.128422
H -2.982510 2.134958 -1.763430
C -2.149144 3.890077 -2.587575
H -3.118888 4.278111 -2.932947
H -1.670798 3.372831 -3.433194
H -1.512553 4.748987 -2.333549
C -2.115623 4.700358 0.434582
H -1.082939 4.333369 0.553772
H -2.048208 5.530783 -0.286972
C -2.634129 5.220894 1.766660
H -3.658512 5.611905 1.672478
H -1.996710 6.028998 2.156108
H -2.653232 4.421509 2.522877
C -7.815941 2.131680 -0.456027
H -7.285388 1.179934 -0.299275
C -8.716833 2.354816 0.771559
H -8.098998 2.709218 1.608474
H -9.421808 3.177228 0.554369
C -9.472834 1.110938 1.219275
H -10.095798 1.319061 2.102785
H -8.768329 0.308252 1.489736
H -10.140116 0.721675 0.434756
C -8.639995 2.002211 -1.744446
H -9.236661 2.918584 -1.895747
C -7.805037 1.720778 -2.984527
H -8.437232 1.581405 -3.874890
H -7.209241 0.806825 -2.849746
H -7.103844 2.543633 -3.190474

C	-7.817229	-1.459306	-1.163911
H	-8.679578	-0.804368	-0.966936
H	-6.950514	-0.785370	-1.236909
C	-8.027556	-2.194588	-2.478153
H	-8.942023	-2.807398	-2.462822
H	-7.187415	-2.869046	-2.702532
H	-8.127840	-1.484020	-3.313177
C	-8.881981	-3.091385	0.461265
H	-9.173382	-3.796433	-0.334895
H	-9.696124	-2.347684	0.499261
H	-9.378144	1.195327	-1.609942
H	-3.303099	1.004912	-4.424493
O	-3.610960	-0.133868	-2.732718
C	-2.757182	0.268096	-3.806359
H	-1.900677	0.778184	-3.346588
C	-2.316335	-0.924707	-4.632004
H	-1.671698	-1.563825	-4.009657
H	-1.695923	-0.568353	-5.469140
C	-3.519069	-1.722937	-5.128323
H	-3.187921	-2.637396	-5.643844
H	-4.075775	-1.125428	-5.872614
C	-4.442470	-2.066919	-3.962688
H	-5.376681	-2.529696	-4.314933
H	-3.963463	-2.794191	-3.289513
C	-4.787604	-0.823725	-3.169878
H	-5.395065	-0.122489	-3.769186
H	-5.350489	-1.072371	-2.260715
Ca	-3.435067	0.162791	-0.395876
Ca	3.447180	-0.181436	-0.015552
N	-1.535348	0.300461	0.840549
N	2.145378	-0.134811	1.911514
N	1.507967	-0.387940	-1.360880
C	-0.236177	0.038699	0.643550
C	0.811303	-0.214922	-0.246097
C	1.076611	-0.112317	1.134261
C	-1.894655	0.648986	2.212082
H	-2.954588	0.971389	2.194153
C	-1.103325	1.835218	2.771890
H	-0.033260	1.581568	2.786380
H	-1.203797	2.686430	2.082707
C	-1.556245	2.213358	4.177989
H	-0.948989	3.049009	4.563160
H	-2.599057	2.580043	4.140741
C	-1.487447	1.018717	5.126250
H	-0.432763	0.717792	5.250076
H	-1.849035	1.301338	6.128675
C	-2.287945	-0.162333	4.584102
H	-3.358324	0.109858	4.560027
H	-2.202548	-1.029818	5.259427
C	-1.850987	-0.543272	3.173029
H	-0.828102	-0.956921	3.184433
H	-2.503431	-1.335125	2.774898
C	2.071017	-0.019046	3.348443
H	1.019347	-0.059761	3.690181

C	2.665366	1.310677	3.819991
H	2.076275	2.136550	3.393127
H	3.678818	1.387927	3.389313
C	2.735220	1.430343	5.338775
H	1.710780	1.460200	5.752355
H	3.207781	2.385362	5.622767
C	3.487666	0.253992	5.954590
H	4.540407	0.283912	5.619613
H	3.504419	0.336773	7.053975
C	2.866484	-1.072229	5.524075
H	1.842875	-1.143080	5.936331
H	3.428873	-1.920096	5.949545
C	2.809649	-1.187352	4.004575
H	3.833383	-1.203466	3.591791
H	2.327307	-2.128702	3.699653
C	0.834923	-0.426685	-2.636022
H	-0.247865	-0.604307	-2.485793
C	1.003772	0.897067	-3.398754
H	0.417356	0.857572	-4.335871
H	0.590600	1.718675	-2.794405
C	2.468231	1.161505	-3.738661
H	3.035151	1.306302	-2.802267
H	2.571297	2.104377	-4.299017
C	3.068874	-0.000150	-4.528516
H	2.583376	-0.047072	-5.520960
H	4.138771	0.176977	-4.717567
C	2.870461	-1.337160	-3.818343
H	3.460667	-1.353916	-2.885750
H	3.264334	-2.161676	-4.433344
C	1.403352	-1.576593	-3.477391
H	0.821992	-1.671163	-4.412449
H	1.288624	-2.520510	-2.922972
N	5.292601	-1.590969	0.466222
N	5.029609	1.564207	0.302800
C	6.565153	-2.234732	2.465530
H	5.777249	-2.797261	2.988770
H	7.222253	-1.777032	3.214673
H	7.134448	-2.974571	1.887560
C	5.925166	-1.196718	1.570157
C	6.035122	0.140229	2.022018
H	6.553621	0.234525	2.975908
C	5.754141	1.384713	1.407328
C	6.363596	2.590901	2.085152
H	7.140654	3.033871	1.444900
H	6.815342	2.326168	3.048645
H	5.608615	3.373617	2.239245
C	5.476534	-2.928080	0.031910
C	4.606531	-3.960966	0.459201
C	4.872644	-5.273953	0.057852
H	4.221679	-6.080007	0.402249
C	5.953005	-5.575310	-0.767828
H	6.149745	-6.609435	-1.060934
C	6.767931	-4.547039	-1.229173
H	7.600795	-4.778171	-1.897775

C	6.542358	-3.219021	-0.850973
C	5.058600	2.855942	-0.284945
C	4.047583	3.805766	-0.010258
C	4.167054	5.093326	-0.547759
H	3.405930	5.840165	-0.314165
C	5.232764	5.439962	-1.370108
H	5.310888	6.451759	-1.775093
C	6.191230	4.480928	-1.685624
H	7.013667	4.745317	-2.352867
C	6.125506	3.189727	-1.155062
C	2.812224	3.422867	0.776005
H	3.064436	2.540059	1.381876
C	1.696468	2.978743	-0.190390
H	2.016023	2.068212	-0.723511
H	0.824140	2.670325	0.408947
C	1.280637	4.000670	-1.236901
H	2.109483	4.238840	-1.918559
H	0.934344	4.944052	-0.787489
H	0.455529	3.612070	-1.847104
C	2.308490	4.497006	1.745755
H	1.956050	5.382964	1.190966
H	1.410589	4.093261	2.245267
C	3.326809	4.924267	2.791652
H	4.218023	5.368644	2.322355
H	3.659821	4.064751	3.393165
H	2.904738	5.670589	3.482204
C	7.127192	2.129043	-1.562992
H	7.206552	1.414415	-0.729312
C	9.201942	3.279964	-0.614168
H	10.221751	3.626769	-0.841498
H	9.274285	2.552020	0.209728
H	8.629102	4.144374	-0.243984
C	3.363487	-3.629483	1.258083
H	3.555696	-2.689189	1.795523
C	2.195945	-3.331713	0.299399
H	1.364030	-2.909185	0.886362
H	2.486060	-2.534064	-0.403777
C	1.709342	-4.501481	-0.539355
H	2.522337	-4.922659	-1.149451
H	0.919121	-4.171267	-1.225609
H	1.291591	-5.312582	0.076575
C	2.970258	-4.672035	2.307903
H	2.014744	-4.348399	2.756523
H	2.749007	-5.638420	1.825468
C	4.003514	-4.877989	3.404913
H	4.972541	-5.190557	2.986248
H	3.681680	-5.650015	4.120739
H	4.167517	-3.949029	3.972973
C	7.448684	-2.114785	-1.355974
H	6.918957	-1.166270	-1.168600
C	8.755670	-2.075436	-0.545222
H	8.506042	-2.162689	0.522419
H	9.350595	-2.974409	-0.787740
C	9.588608	-0.817898	-0.744320
H	10.494508	-0.837691	-0.119116
H	9.010906	0.075064	-0.462122
H	9.914534	-0.690079	-1.787840
C	7.710574	-2.221784	-2.863840
H	8.315749	-3.121489	-3.073253
C	6.446019	-2.239300	-3.709730
H	6.679533	-2.272365	-4.785122
H	5.839973	-1.339929	-3.528139
H	5.816753	-3.109747	-3.472097
C	6.574369	1.318643	-2.750371
H	7.289296	0.510425	-2.968544
H	5.646686	0.822538	-2.418782
C	6.298404	2.111521	-4.019109
H	7.198022	2.634251	-4.379625
H	5.514431	2.866937	-3.860057
H	5.964534	1.446859	-4.830041
C	8.538110	2.657322	-1.833838
H	8.531000	3.382529	-2.664260
H	9.148587	1.812386	-2.193218
H	8.343272	-1.372751	-3.170240

146

LCa-CaL (A)

Ca	1.767358	-0.090234	0.035513
Ca	-1.774711	0.054201	-0.123382
N	3.478352	-1.279227	1.102776
N	-3.734536	-0.805513	-1.063235
N	3.724941	0.777848	-0.840464
N	-3.493642	1.148704	0.982927
C	5.595611	-2.161238	1.988790
C	-6.029398	-1.438855	-1.673611
H	4.946263	-2.813440	2.587374
H	-5.535783	-2.137483	-2.361897
H	6.242153	-1.584745	2.668246
H	-6.706433	-2.005406	-1.016198
H	6.258021	-2.790876	1.375054
H	-6.655851	-0.750806	-2.262256
C	4.795108	-1.232495	1.104468
C	-5.026638	-0.654900	-0.858563
C	5.529606	-0.346789	0.283598
C	-5.544245	0.248650	0.096405
H	6.615888	-0.409858	0.360355
H	-6.631379	0.304051	0.166238
C	5.021026	0.580259	-0.644300
C	-4.818680	1.107688	0.942693
C	6.005411	1.379922	-1.459654
C	-5.597603	2.034239	1.841949
H	5.822403	2.457380	-1.330694
H	-5.309696	1.879201	2.892656
H	5.868719	1.170424	-2.531589
H	-5.355712	3.082236	1.608249
H	7.044467	1.158304	-1.186010
H	-6.680750	1.890713	1.743228
C	2.681582	-2.085418	1.925583

C	-3.130561	-1.728076	-1.924615
C	2.400760	-1.690570	3.260885
C	-2.963737	-3.080080	-1.519939
C	1.462387	-2.428938	3.985627
C	-2.191541	-3.919722	-2.325850
H	1.237446	-2.152170	5.016077
H	-2.053459	-4.963315	-2.041022
C	0.786797	-3.506726	3.415627
C	-1.569536	-3.449965	-3.482082
H	0.050026	-4.060370	4.002084
H	-0.962324	-4.126539	-4.087658
C	1.039342	-3.862397	2.095677
C	-1.706385	-2.116478	-3.849496
H	0.497405	-4.698785	1.649751
H	-1.202906	-1.749919	-4.746122
C	1.976931	-3.163383	1.329279
C	-2.475267	-1.235220	-3.083260
C	3.085355	-0.468186	3.855805
C	-3.584345	-3.569361	-0.218514
H	4.157154	-0.542799	3.609725
H	-4.627925	-3.214727	-0.201946
C	2.979413	-0.391438	5.376569
C	-3.626184	-5.090440	-0.099499
H	3.594634	0.438264	5.756187
H	-4.193037	-5.383238	0.797134
H	1.944175	-0.204215	5.703736
H	-2.615816	-5.518306	0.000472
C	2.574680	0.835263	3.223262
C	-2.891597	-2.961796	1.010628
H	2.973050	1.712169	3.756561
H	-3.259969	-3.427743	1.937604
H	2.920665	0.929265	2.182627
H	-3.114963	-1.887830	1.099967
C	2.284562	-3.566422	-0.102094
C	-2.660259	0.215112	-3.494720
H	2.674973	-2.666436	-0.612046
H	-2.937556	0.774094	-2.582024
C	3.419890	-4.594232	-0.135454
C	-3.852754	0.349131	-4.447203
H	3.694763	-4.844121	-1.172532
H	-4.040003	1.405037	-4.699228
H	4.316214	-4.212702	0.373997
H	-4.767156	-0.063797	-3.997206
C	1.062259	-4.042272	-0.881001
C	-1.398819	0.858161	-4.065167
H	0.663643	-4.992066	-0.490327
H	-1.103907	0.409165	-5.026832
H	0.256390	-3.291554	-0.851649
H	-0.552031	0.761253	-3.366687
C	3.204475	1.658627	-1.806682
C	-2.772232	2.051768	1.787188
C	2.812568	2.966332	-1.423312
C	-2.289499	1.635138	3.053008
C	2.105804	3.754272	-2.336203
C	-1.410588	2.470201	3.749173
H	1.795288	4.761272	-2.045450
H	-1.030256	2.152192	4.723660
C	1.782097	3.277247	-3.603241
C	-1.008374	3.694857	3.223519
H	1.222289	3.904847	-4.300560
H	-0.317766	4.332643	3.780164
C	2.165777	1.990749	-3.972416
C	-1.482465	4.096574	1.977870
H	1.900153	1.614989	-4.964022
H	-1.156354	5.053440	1.562314
C	2.870822	1.163386	-3.094119
C	-2.354694	3.292488	1.239559
C	3.134103	3.499304	-0.038678
C	-2.699265	0.297731	3.641740
H	3.704029	2.713668	0.480796
H	-3.408788	-0.158913	2.934779
C	1.864032	3.764532	0.773202
C	-1.503570	-0.647973	3.773626
H	1.268720	4.577390	0.329174
H	-0.762162	-0.259854	4.489909
H	1.203665	2.880680	0.815535
H	-0.973993	-0.782770	2.814030
C	4.020080	4.745719	-0.101234
C	-3.424336	0.463856	4.979322
H	4.304413	5.073097	0.911337
H	-3.780929	-0.509277	5.352452
H	4.943063	4.555187	-0.669548
H	-4.293671	1.132320	4.886288
C	3.248725	-0.247859	-3.510155
C	-2.820588	3.735420	-0.136796
H	3.818965	-0.686751	-2.677091
H	-3.513540	2.963270	-0.504990
C	2.011894	-1.123311	-3.733296
C	-1.655061	3.819521	-1.127620
H	1.410870	-0.757930	-4.580648
H	-0.944701	4.612002	-0.844783
H	2.300021	-2.164567	-3.947917
H	-2.016931	4.039750	-2.144355
C	4.155563	-0.252493	-4.743039
C	-3.591609	5.056142	-0.080439
H	4.483335	-1.276477	-4.982450
H	-3.985200	5.320582	-1.074635
H	3.633205	0.144509	-5.628182
H	-2.946693	5.885101	0.252426
H	5.053263	0.363993	-4.584569
H	-4.440885	4.996822	0.616866
H	1.344464	-1.130324	-2.853419
H	-1.075074	2.880616	-1.167980
H	3.115436	-5.523731	0.371961
H	-3.663506	-0.198576	-5.384430
H	1.321678	-4.208753	-1.937372
H	-1.564242	1.931542	-4.243815
H	3.498918	5.585825	-0.587590

H -2.759532 0.890378 5.747492
H 2.107758 4.052763 1.807634
H -1.819742 -1.642290 4.125244
H 3.325791 -1.318235 5.858443
H -4.108155 -5.556764 -0.972075
H 1.474874 0.895656 3.245047
H -1.799620 -3.101204 0.973482

152
LCa-(CN-Me)-CaL (B)
Ca -2.018420 -0.030193 -0.462814
Ca 1.954136 0.520073 0.618064
N -3.134459 -2.056378 -0.763676
N 3.989236 -0.672487 0.853996
N -4.177133 0.380272 0.383170
N 3.379601 1.679268 -0.865975
C -4.756844 -3.859591 -1.062302
C 6.367124 -1.296856 0.858917
H -4.268262 -4.178504 -1.994562
H 6.028357 -2.238033 1.310778
H -5.841008 -3.999702 -1.157407
H 7.055431 -1.520207 0.031577
H -4.388143 -4.535021 -0.272624
H 6.942510 -0.745675 1.620747
C -4.399767 -2.437387 -0.703272
C 5.208651 -0.437402 0.400693
C -5.432833 -1.582710 -0.278183
C 5.526497 0.612346 -0.487082
H -6.439992 -1.999115 -0.306147
H 6.578481 0.678929 -0.767301
C -5.322966 -0.271638 0.240903
C 4.680480 1.557922 -1.103589
C -6.612779 0.399047 0.656579
C 5.318976 2.479375 -2.116163
H -6.652675 1.426401 0.264549
H 4.807053 2.386116 -3.086436
H -6.680739 0.484449 1.751703
H 5.208774 3.531481 -1.813408
H -7.487330 -0.159187 0.299522
H 6.384803 2.260744 -2.253829
C -2.127643 -2.797635 -1.390824
C 3.615924 -1.880949 1.456829
C -1.994243 -2.731250 -2.806893
C 3.524700 -3.066051 0.672216
C -0.883096 -3.343499 -3.395142
C 3.075224 -4.235947 1.288611
H -0.755337 -3.310399 -4.477721
H 3.012003 -5.159633 0.711936
C 0.074615 -4.003714 -2.626021
C 2.693911 -4.253448 2.630968
H 0.936229 -4.468070 -3.110661
H 2.344167 -5.181941 3.087606
C -0.065126 -4.059809 -1.243479
C 2.737046 -3.080327 3.373869
H 0.689608 -4.570182 -0.640916
H 2.417903 -3.091728 4.418389
C -1.155354 -3.462694 -0.604326
C 3.183541 -1.882792 2.804726
C -3.057158 -2.026707 -3.645956
C 3.875553 -3.027659 -0.808225
H -4.030006 -2.457762 -3.354641
H 4.882438 -2.587820 -0.899327
C -2.896958 -2.261472 -5.145675
C 3.940629 -4.405851 -1.459495
H -3.748793 -1.825507 -5.688746
H 4.281846 -4.312190 -2.501392
H -1.981691 -1.788329 -5.536574
H 2.955148 -4.896483 -1.482426
C -3.164650 -0.519496 -3.363093
C 2.923400 -2.106468 -1.582613
H -3.800786 -0.026924 -4.113509
H 3.080801 -2.203262 -2.667458
H -3.641228 -0.332712 -2.389591
H 3.109940 -1.056059 -1.324147
C -1.297778 -3.518246 0.904561
C 3.261157 -0.614791 3.633778
H -2.317213 -3.175019 1.138941
H 3.369497 0.228846 2.929000
C -1.141036 -4.939610 1.447119
C 4.531799 -0.609571 4.488699
H -1.356936 -4.962912 2.526538
H 4.628531 0.334428 5.048136
H -1.825148 -5.640215 0.944288
H 5.427500 -0.732440 3.862328
C -0.316685 -2.562418 1.588021
C 2.006350 -0.371168 4.470817
H 0.723374 -2.831787 1.349725
H 1.887512 -1.130295 5.259567
H -0.463507 -1.513752 1.279182
H 1.100347 -0.382346 3.846488
C -4.136979 1.588019 1.111387
C 2.622810 2.687773 -1.504406
C -3.867066 2.801094 0.438126
C 1.831814 2.367724 -2.633129
C -3.757731 3.982679 1.176310
C 0.987118 3.343793 -3.170322
H -3.542135 4.921868 0.659991
H 0.375063 3.101771 -4.043438
C -3.912593 3.978114 2.558835
C 0.913998 4.617143 -2.614572
H -3.831370 4.908724 3.125391
H 0.245207 5.367444 -3.042902
C -4.151887 2.773456 3.220120
C 1.700957 4.928928 -1.508391
H -4.236396 2.771412 4.309094
H 1.641522 5.929614 -1.073674
C -4.254547 1.568044 2.524463
C 2.564252 3.987234 -0.942138

C -3.603279 2.796982 -1.055870
C 1.878193 0.978954 -3.240641
H -3.914807 1.803525 -1.422074
H 2.705506 0.445874 -2.752759
C -2.103816 2.979626 -1.337698
C 0.588925 0.211817 -2.936792
H -1.791522 4.019015 -1.155444
H -0.270794 0.716286 -3.406493
H -1.464435 2.386826 -0.654327
H 0.403972 0.180115 -1.843879
C -4.438943 3.817983 -1.825851
C 2.167275 0.998657 -4.741553
H -4.253848 3.737399 -2.908937
H 2.277914 -0.027097 -5.127166
H -5.513785 3.664107 -1.648202
H 3.095302 1.547886 -4.960815
C -4.346081 0.245796 3.266192
C 3.388886 4.337893 0.282847
H -4.785314 -0.494530 2.582918
H 4.166842 3.567170 0.384103
C -2.933625 -0.250355 3.595775
C 2.526438 4.271707 1.545693
H -2.460681 0.396304 4.351838
H 1.720860 5.022626 1.511423
H -2.957341 -1.279587 3.988594
H 3.129401 4.458506 2.448680
C -5.228057 0.297362 4.511218
C 4.092889 5.689133 0.158983
H -5.338145 -0.709311 4.943768
H 4.764459 5.852112 1.016304
H -4.797186 0.939250 5.295989
H 3.377361 6.526364 0.143528
H -6.234373 0.681290 4.280757
H 4.694540 5.748306 -0.761115
H -2.290105 -0.223164 2.701827
H 2.041645 3.288963 1.654057
H -0.113578 -5.312740 1.313887
H 4.514736 -1.437397 5.215601
H -0.427117 -2.596494 2.682264
H 2.060766 0.610204 4.965832
H -4.192734 4.848546 -1.525548
H 1.354292 1.478393 -5.309597
H -1.847201 2.746075 -2.383119
H 0.620678 -0.825089 -3.303616
H -2.856221 -3.333142 -5.390515
H 4.639868 -5.075230 -0.935919
H -2.177008 -0.033441 -3.415087
H 1.872222 -2.353787 -1.377711
N 0.269577 1.459096 1.806717
C -0.448403 0.829988 0.940608
C -0.349102 2.262322 2.854465
H 0.076198 3.279463 2.849595
H -1.439918 2.324033 2.713732
H -0.130506 1.826925 3.844554

158
LCa-(CN-Me)-CaL + MeCN coord (C)
Ca 2.052124 -0.050192 0.479942
Ca -2.037247 0.087616 0.320433
N 2.755402 -2.325632 0.406693
N -4.087986 -0.127621 -0.867096
N 4.014970 0.034928 -0.931494
N -2.817384 2.320809 0.132887
C 4.009369 -4.392121 0.046566
C -6.323721 0.221706 -1.839438
H 3.964918 -4.699792 1.101004
H -6.264044 -0.840744 -2.108873
H 4.954159 -4.741234 -0.387488
H -7.157898 0.351179 -1.131576
H 3.176223 -4.907833 -0.455728
H -6.574332 0.805313 -2.737582
C 3.846268 -2.895938 -0.088625
C -5.044120 0.720222 -1.202473
C 4.879034 -2.213287 -0.764566
C -4.960589 2.108965 -0.981007
H 5.730202 -2.830706 -1.054968
H -5.814683 2.691801 -1.328204
C 4.946619 -0.870659 -1.184507
C -3.909322 2.851098 -0.406978
C 6.196107 -0.490446 -1.953960
C -4.046926 4.355504 -0.439790
H 6.128736 0.518406 -2.380899
H -3.994411 4.783167 0.572257
H 6.400622 -1.207604 -2.762573
H -3.207243 4.795888 -0.999736
H 7.062992 -0.519373 -1.274704
H -4.987446 4.668466 -0.909207
C 1.786943 -3.117500 1.068563
C -4.030928 -1.454009 -1.306683
C 1.901564 -3.371069 2.457223
C -4.115758 -2.494553 -0.346531
C 0.860557 -4.036167 3.108837
C -3.904629 -3.814471 -0.758476
H 0.937412 -4.224936 4.182855
H -3.975421 -4.623814 -0.029207
C -0.277310 -4.451796 2.421529
C -3.615452 -4.111113 -2.086419
H -1.085179 -4.961590 2.952053
H -3.456037 -5.146432 -2.395608
C -0.372419 -4.222039 1.052450
C -3.524385 -3.081978 -3.023765
H -1.257831 -4.556660 0.506268
H -3.283695 -3.332329 -4.057744
C 0.653774 -3.574755 0.358266
C -3.715227 -1.746747 -2.663161
C 3.124782 -2.925437 3.237765
C -4.500199 -2.165267 1.084685
H 3.848658 -2.534209 2.507581

H	-4.157533	-1.134954	1.292523
C	3.791704	-4.086308	3.979057
C	-6.025171	-2.127098	1.226968
H	4.724608	-3.753332	4.460970
H	-6.318073	-1.829624	2.246239
H	3.139065	-4.490334	4.769311
H	-6.455501	-3.120224	1.021604
C	2.785822	-1.781782	4.195072
C	-3.858459	-3.079889	2.122072
H	3.693335	-1.395822	4.686539
H	-4.076477	-2.713842	3.136451
H	2.299190	-0.956941	3.655250
H	-2.766491	-3.113133	2.002986
C	0.538833	-3.357747	-1.136514
C	-3.560380	-0.616700	-3.671748
H	1.519997	-3.008448	-1.487964
H	-4.428934	0.048919	-3.550612
C	0.204192	-4.643484	-1.893173
C	-3.563623	-1.085817	-5.123590
H	0.227139	-4.468998	-2.980569
H	-3.562495	-0.217677	-5.799860
H	0.923699	-5.442313	-1.658447
H	-4.451236	-1.693680	-5.355485
C	-0.480404	-2.265425	-1.456586
C	-2.319399	0.238980	-3.392118
H	-1.479656	-2.549305	-1.091788
H	-1.398757	-0.357563	-3.462548
H	-0.181095	-1.317031	-0.972085
H	-2.376762	0.697771	-2.395083
C	3.945560	1.262350	-1.604985
C	-1.839248	3.160432	0.714855
C	4.109755	2.465954	-0.871901
C	-1.973873	3.578377	2.061093
C	3.865021	3.686535	-1.510734
C	-0.919640	4.279024	2.652714
H	3.990121	4.617623	-0.954355
H	-1.016135	4.618819	3.686814
C	3.481914	3.733423	-2.847401
C	0.250332	4.560468	1.949501
H	3.297344	4.694102	-3.333443
H	1.072422	5.086972	2.439067
C	3.343048	2.548379	-3.568728
C	0.359239	4.177784	0.616834
H	3.045581	2.601112	-4.617012
H	1.266167	4.415368	0.055550
C	3.560232	1.303111	-2.975002
C	-0.682426	3.499031	-0.022322
C	4.634575	2.417841	0.551334
C	-3.232321	3.247817	2.843200
H	4.294315	1.467141	0.995329
H	-4.029816	3.058184	2.110052
C	4.123865	3.543968	1.443100
C	-3.054549	1.953432	3.637549
H	4.515068	4.526006	1.132439
H	-2.276960	2.068477	4.408938
H	3.026370	3.585404	1.444005
H	-2.739320	1.124933	2.986806
C	6.165973	2.364968	0.542062
C	-3.698091	4.391238	3.743017
H	6.561022	2.272728	1.566205
H	-4.675898	4.153199	4.189818
H	6.530921	1.510026	-0.042844
H	-3.799523	5.332177	3.180581
C	3.364561	0.010348	-3.754805
C	-0.575663	3.136864	-1.489731
H	4.217997	-0.643262	-3.520940
H	-1.569302	2.787716	-1.806880
C	2.111753	-0.746474	-3.305848
C	0.408750	1.991268	-1.709172
H	1.199960	-0.168206	-3.512811
H	1.427052	2.288420	-1.413805
H	2.029727	-1.709836	-3.831826
H	0.448510	1.691830	-2.764395
C	3.358842	0.199359	-5.268879
C	-0.207509	4.338375	-2.360420
H	3.336051	-0.780174	-5.770296
H	-0.226877	4.062631	-3.426475
H	2.472303	0.757914	-5.609878
H	0.806342	4.702416	-2.137344
H	4.253370	0.737662	-5.617230
H	-0.910664	5.170857	-2.206872
H	2.157100	-0.970627	-2.231563
H	0.124207	1.104342	-1.110567
H	-0.802942	-5.009856	-1.643082
H	-2.669801	-1.684245	-5.362317
H	-0.558975	-2.093245	-2.538267
H	-2.245089	1.063611	-4.117117
H	6.582722	3.281778	0.094721
H	-2.999140	4.573324	4.574823
H	4.444725	3.376639	2.481877
H	-3.991479	1.661701	4.138234
H	4.038556	-4.915865	3.299077
H	-6.471142	-1.414043	0.519977
H	2.091964	-2.117824	4.983099
H	-4.245020	-4.109206	2.057565
N	-1.031353	-0.575205	2.254908
C	0.033760	-0.261614	1.616978
C	-1.032202	-1.045580	3.631323
H	-1.700437	-0.423720	4.248677
H	-1.412096	-2.077585	3.668597
H	-0.014285	-1.034484	4.053069
N	1.593154	1.786694	3.555172
C	1.840031	1.225509	2.533179
C	0.856844	1.905357	4.754484
H	0.110816	2.705050	4.640920
H	0.332852	0.963701	4.991953
H	1.522343	2.171079	5.589319

158
LCa-(CN-Me)2-CaL (D)
Ca -2.183386 -0.118878 -0.168724
Ca 1.823085 -0.114599 0.329973
N -4.285693 -1.049425 -0.539579
N 3.406040 -1.736098 0.930398
N -3.305060 1.719747 -1.099497
N 3.637637 1.275601 0.827140
C -6.518375 -1.510390 -1.448119
C 5.115140 -2.739270 2.386278
H -6.598399 -2.262351 -0.651177
H 6.097682 -2.855127 1.902738
H -6.433621 -2.051665 -2.404279
H 5.298888 -2.592162 3.460148
H -7.444174 -0.920557 -1.485485
H 4.557166 -3.673304 2.239051
C -5.312236 -0.621274 -1.250019
C 4.379091 -1.548700 1.810947
C -5.356690 0.651565 -1.861779
C 4.826767 -0.282299 2.250899
H -6.256754 0.850533 -2.445221
H 5.600355 -0.315084 3.020728
C -4.468034 1.741699 -1.747111
C 4.564700 1.008238 1.733649
C -4.931395 3.035064 -2.382102
C 5.417086 2.125284 2.295399
H -4.092650 3.578149 -2.840054
H 6.456002 1.799160 2.441110
H -5.359797 3.705760 -1.620767
H 5.403208 3.012508 1.649016
H -5.702846 2.850207 -3.140527
H 5.027091 2.422861 3.281929
C -4.229445 -2.283613 0.126130
C 3.176714 -2.972466 0.298091
C -3.935833 -3.484953 -0.564388
C 4.078984 -3.455887 -0.682705
C -3.769172 -4.660348 0.174392
C 3.753429 -4.619296 -1.388368
H -3.545091 -5.592286 -0.351183
H 4.445480 -4.993139 -2.147560
C -3.868463 -4.666227 1.562158
C 2.559093 -5.296989 -1.160674
H -3.733975 -5.596265 2.119459
H 2.319858 -6.199314 -1.728325
C -4.120541 -3.475136 2.238787
C 1.668554 -4.809797 -0.206753
H -4.179730 -3.480392 3.328926
H 0.728408 -5.336361 -0.029424
C -4.294419 -2.274842 1.545940
C 1.956716 -3.659876 0.532371
C -3.746195 -3.505000 -2.069406
C 5.342602 -2.695454 -1.043125
H -4.026091 -2.511914 -2.449746
H 5.470324 -1.884412 -0.312872
C -4.643642 -4.539184 -2.751816
C 6.592566 -3.575227 -0.989574
H -4.560347 -4.462343 -3.847315
H 7.498912 -2.972072 -1.156330
H -4.362280 -5.567189 -2.473248
H 6.573031 -4.358479 -1.763972
C -2.272100 -3.720491 -2.425794
C 5.189812 -2.033233 -2.414262
H -2.130898 -3.725179 -3.518594
H 6.082864 -1.440527 -2.666671
H -1.642726 -2.917341 -2.011035
H 4.320972 -1.360620 -2.428818
C -4.588066 -0.976995 2.276666
C 0.998591 -3.182553 1.606121
H -4.202018 -0.156846 1.646667
H 1.198217 -2.113820 1.773628
C -6.097758 -0.741245 2.378762
C 1.303655 -3.866502 2.942588
H -6.312729 0.223552 2.865019
H 0.630735 -3.498669 3.733891
H -6.564350 -0.729422 1.383638
H 2.340194 -3.676644 3.257577
C -3.905338 -0.873033 3.636888
C -0.473034 -3.324166 1.229432
H -4.313396 -1.594799 4.361775
H -0.805988 -4.372351 1.220429
H -2.821468 -1.044903 3.549350
H -0.670974 -2.907525 0.230924
C -2.627101 2.933466 -0.853726
C 3.482266 2.539302 0.221682
C -1.412109 3.216701 -1.523153
C 4.041803 2.740398 -1.067458
C -0.784853 4.446674 -1.296041
C 3.831134 3.960661 -1.712874
H 0.143787 4.680371 -1.818202
H 4.268463 4.129666 -2.698765
C -1.316772 5.370700 -0.403087
C 3.058596 4.962663 -1.128177
H -0.820520 6.331806 -0.247788
H 2.904571 5.911362 -1.648116
C -2.458169 5.044764 0.325849
C 2.464705 4.736732 0.108291
H -2.837843 5.747790 1.070779
H 1.827922 5.506644 0.550200
C -3.115854 3.827942 0.132443
C 2.652894 3.533041 0.796196
C -0.809559 2.215674 -2.489722
C 4.846936 1.631371 -1.717340
H -1.106650 1.207034 -2.153082
H 4.440947 0.685506 -1.320333
C 0.716289 2.245968 -2.496771
C 4.713379 1.604240 -3.237883
H 1.116618 3.138497 -2.999833
H 5.196654 2.474018 -3.710285

H 1.127134 2.258443 -1.474584
H 3.657686 1.596750 -3.548899
C -1.381744 2.373048 -3.899801
C 6.317299 1.684110 -1.293281
H -0.945417 1.628901 -4.585109
H 6.885475 0.856721 -1.747064
H -2.471981 2.233913 -3.900369
H 6.423004 1.603634 -0.203232
C -4.265621 3.417248 1.034615
C 1.948222 3.307259 2.119034
H -4.839404 2.635011 0.517210
H 2.169246 2.271474 2.417943
C -3.697116 2.784436 2.310355
C 0.430794 3.452014 1.981305
H -3.117880 3.523046 2.887373
H 0.142547 4.496244 1.794861
H -4.502714 2.399087 2.955184
H -0.079755 3.126332 2.900499
C -5.229916 4.554414 1.364866
C 2.480302 4.233674 3.215737
H -6.093899 4.172404 1.930519
H 2.007512 4.004888 4.184427
H -4.756024 5.332036 1.984682
H 2.260926 5.287526 2.980782
H -5.608955 5.039971 0.452511
H 3.568797 4.140481 3.334509
H -3.014936 1.950672 2.079990
H 0.030674 2.859952 1.145975
H -6.576930 -1.538555 2.969265
H 1.169384 -4.957061 2.862087
H -4.053222 0.131545 4.060382
H -1.112145 -2.796213 1.953372
H -1.163570 3.376806 -4.298505
H 6.780987 2.632069 -1.610605
H 1.114905 1.365742 -3.021690
H 5.197835 0.706309 -3.648626
H -5.699901 -4.398987 -2.477333
H 6.689569 -4.077013 -0.015332
H -1.899229 -4.679480 -2.031997
H 5.045733 -2.783666 -3.207988
N 0.465648 0.135830 2.240295
C -0.547798 0.122319 1.529216
C 0.574475 0.236402 3.684529
H 1.272311 1.044748 3.945030
H -0.405796 0.422704 4.151729
H 0.997531 -0.702165 4.073126
N 0.959420 -1.100991 -1.617996
C -0.185278 -0.855367 -1.213048
C 1.372281 -1.849733 -2.793421
H 2.047422 -1.230180 -3.401686
H 1.931276 -2.738260 -2.464570
H 0.505340 -2.156466 -3.399203

158

TS LCa-(CN-Me)2-CaL (E*)
Ca -2.354881 -0.192861 -0.072198
Ca 1.923764 -0.281512 0.360421
N -4.518122 -0.870368 -0.587618
N 3.656434 -1.802910 0.789679
N -3.123185 1.715292 -1.230699
N 3.642573 1.236785 0.911500
C -6.670366 -1.138187 -1.735977
C 5.589721 -2.742082 1.972386
H -6.941455 -1.798142 -0.900689
H 6.394530 -2.823041 1.223754
H -6.546955 -1.771345 -2.629430
H 6.063288 -2.579101 2.949572
H -7.498653 -0.445189 -1.936091
H 5.059097 -3.703676 1.977023
C -5.388029 -0.389687 -1.455308
C 4.670496 -1.596101 1.615011
C -5.189725 0.814934 -2.167249
C 5.005813 -0.338039 2.169475
H -5.979673 1.062151 -2.877761
H 5.805935 -0.366757 2.909775
C -4.192348 1.801853 -2.023295
C 4.589886 0.960217 1.803952
C -4.417590 3.072710 -2.815173
C 5.298582 2.104433 2.493988
H -3.472709 3.470761 -3.211212
H 6.175899 1.760955 3.055547
H -4.841071 3.858900 -2.170547
H 5.610267 2.873040 1.772449
H -5.116687 2.903641 -3.643997
H 4.609745 2.600402 3.194780
C -4.709915 -2.033596 0.176574
C 3.500032 -3.033991 0.119031
C -4.499635 -3.321887 -0.372273
C 4.297446 -3.324099 -1.013985
C -4.579556 -4.432736 0.473378
C 4.074991 -4.516040 -1.710227
H -4.420630 -5.431239 0.057926
H 4.685776 -4.747307 -2.586359
C -4.842028 -4.292928 1.832550
C 3.078695 -5.406460 -1.317219
H -4.899397 -5.174736 2.475023
H 2.919707 -6.334275 -1.871922
C -5.014408 -3.020708 2.372990
C 2.275675 -5.097801 -0.221762
H -5.204870 -2.914114 3.442784
H 1.482411 -5.787605 0.074593
C -4.944017 -1.880553 1.569164
C 2.462614 -3.918124 0.505025
C -4.129400 -3.508968 -1.831228
C 5.307249 -2.314968 -1.529652
H -4.212720 -2.527848 -2.321214
H 5.499777 -1.590406 -0.725872
C -5.073313 -4.474486 -2.550493

C	6.645128	-2.941744	-1.918988
H	-4.838971	-4.517478	-3.625775
H	7.374495	-2.159488	-2.181286
H	-4.983812	-5.498474	-2.154360
H	6.551797	-3.605659	-2.792993
C	-2.669053	-3.953133	-1.960391
C	4.700299	-1.529784	-2.695481
H	-2.382519	-4.053672	-3.019607
H	5.402537	-0.769836	-3.069985
H	-1.989365	-3.221947	-1.495204
H	3.778640	-1.014763	-2.385340
C	-5.149138	-0.492659	2.148291
C	1.586342	-3.602801	1.701744
H	-4.602239	0.210914	1.496597
H	1.656103	-2.517551	1.870787
C	-6.620471	-0.077971	2.056417
C	2.127014	-4.270919	2.969336
H	-6.764718	0.947881	2.431235
H	1.500545	-4.023460	3.841494
H	-6.976711	-0.111350	1.017036
H	3.154055	-3.944095	3.186618
C	-4.604210	-0.336189	3.565049
C	0.112006	-3.933453	1.480726
H	-5.173831	-0.932437	4.294919
H	-0.067855	-5.019113	1.434325
H	-3.548729	-0.644822	3.621363
H	-0.263209	-3.485131	0.549819
C	-2.355959	2.880505	-1.004411
C	3.443383	2.567792	0.481849
C	-1.055117	2.995803	-1.550037
C	4.072189	2.990770	-0.718054
C	-0.340244	4.181786	-1.352221
C	3.873020	4.302520	-1.155917
H	0.660713	4.289137	-1.771712
H	4.361536	4.645331	-2.069970
C	-0.874824	5.226861	-0.606251
C	3.052725	5.182565	-0.450801
H	-0.304991	6.148985	-0.472313
H	2.915446	6.207375	-0.804379
C	-2.116064	5.071717	0.005720
C	2.383354	4.736817	0.682974
H	-2.506751	5.872607	0.637409
H	1.701495	5.410490	1.206977
C	-2.862410	3.902750	-0.162749
C	2.549426	3.431314	1.157310
C	-0.446302	1.853461	-2.340244
C	4.911295	2.007544	-1.515225
H	-0.844108	0.912135	-1.922274
H	4.445698	1.020319	-1.357850
C	1.071106	1.788671	-2.197613
C	4.910982	2.297507	-3.014329
H	1.579022	2.585898	-2.759385
H	5.462862	3.219820	-3.255528
H	1.382352	1.930300	-1.149585
H	3.889620	2.401297	-3.409594
C	-0.868661	1.876018	-3.809866
C	6.346533	1.895608	-0.991704
H	-0.422925	1.030816	-4.358106
H	6.926944	1.191039	-1.608786
H	-1.960685	1.801447	-3.906003
H	6.374977	1.525915	0.041240
C	-4.140574	3.676937	0.624433
C	1.723890	2.951915	2.336529
H	-4.743761	2.931115	0.086935
H	2.041686	1.918943	2.549116
C	-3.784671	3.066836	1.984918
C	0.234647	2.928967	1.978446
H	-3.181640	3.768182	2.583122
H	-0.152136	3.944679	1.811572
H	-4.690473	2.814272	2.557722
H	-0.358463	2.464317	2.780445
C	-4.998115	4.929163	0.790687
C	1.957361	3.780097	3.601530
H	-5.955740	4.676327	1.271534
H	1.395451	3.359500	4.450572
H	-4.508648	5.686043	1.423834
H	1.619391	4.819720	3.466351
H	-5.219054	5.397825	-0.180517
H	3.021070	3.811590	3.879501
H	-3.188081	2.148242	1.870544
H	0.042078	2.368074	1.052334
H	-7.252970	-0.754124	2.653698
H	2.137509	-5.367534	2.860861
H	-4.668858	0.714273	3.885581
H	-0.497241	-3.537414	2.307534
H	-0.543285	2.810575	-4.294250
H	6.853890	2.873282	-1.024765
H	1.457840	0.828477	-2.571220
H	5.403173	1.479094	-3.560701
H	-6.124188	-4.167726	-2.440445
H	7.064716	-3.536424	-1.093351
H	-2.502490	-4.926233	-1.471511
H	4.441834	-2.199249	-3.531448
N	0.477053	-0.411870	2.161492
C	-0.530472	-0.363652	1.419378
C	0.423759	-0.401788	3.612685
H	1.031934	0.431100	3.996782
H	-0.610970	-0.307839	3.981690
H	0.866607	-1.334565	3.995092
N	0.792004	-1.472901	-1.277289
C	-0.321160	-1.143067	-0.810163
C	0.974289	-2.311695	-2.451686
H	1.564244	-1.764236	-3.201895
H	1.549649	-3.204395	-2.164991
H	0.009892	-2.610774	-2.891000

158

LCa-(MeNC-CNMe)-CaL (F)

Ca	-2.606107	0.268482	-0.798988
Ca	2.152856	0.023613	-0.256489
N	-4.283471	1.260034	0.457674
N	3.958159	1.193073	-1.187615
N	-3.927173	-1.567377	-0.308806
N	4.005615	-1.169028	0.571173
C	-6.544587	1.799829	1.233117
C	6.183154	1.962819	-1.901233
H	-6.368811	2.014409	2.298104
H	5.637821	2.819431	-2.318514
H	-7.561935	1.401648	1.130065
H	7.002049	2.332115	-1.266352
H	-6.475022	2.761986	0.704108
H	6.647680	1.410474	-2.733891
C	-5.517538	0.828811	0.699060
C	5.266985	1.033599	-1.133947
C	-5.935927	-0.503438	0.511437
C	5.893047	0.004318	-0.399493
H	-6.973899	-0.707968	0.775366
H	6.983117	-0.005729	-0.444038
C	-5.167221	-1.635239	0.153789
C	5.312247	-0.975294	0.432267
C	-5.818696	-2.987342	0.323486
C	6.271032	-1.813841	1.247872
H	-5.164707	-3.654645	0.904436
H	6.307766	-1.428112	2.279893
H	-5.969009	-3.478302	-0.650356
H	5.942429	-2.859838	1.316688
H	-6.788792	-2.908621	0.828993
H	7.288392	-1.778627	0.837880
C	-3.830975	2.504514	0.933091
C	3.301951	2.359726	-1.606445
C	-3.617808	2.706307	2.322259
C	3.369550	3.537533	-0.812964
C	-3.097267	3.928570	2.750977
C	2.522206	4.600613	-1.130035
H	-2.930443	4.092529	3.818065
H	2.553003	5.510595	-0.528976
C	-2.750623	4.930992	1.846368
C	1.619297	4.521465	-2.188251
H	-2.338262	5.876850	2.204842
H	0.958649	5.363685	-2.407957
C	-2.903394	4.704272	0.483514
C	1.567022	3.370138	-2.965972
H	-2.599692	5.475734	-0.228824
H	0.862161	3.317644	-3.796829
C	-3.438197	3.502667	0.008210
C	2.402390	2.281705	-2.700043
C	-3.807982	1.570115	3.312077
C	4.299920	3.607246	0.389188
H	-4.459091	0.816524	2.848105
H	5.268184	3.183981	0.084073
C	-4.465588	2.005526	4.620577
C	4.578848	5.030382	0.865948
H	-4.674333	1.129748	5.254304
H	5.356262	5.020635	1.645163
H	-3.818015	2.679370	5.203449
H	3.684400	5.497841	1.307829
C	-2.460162	0.885759	3.565411
C	3.792261	2.746326	1.549368
H	-2.581893	0.013741	4.226471
H	4.468331	2.823371	2.415125
H	-2.005173	0.543668	2.623988
H	3.755386	1.686385	1.264258
C	-3.550200	3.268500	-1.488262
C	2.389062	1.040399	-3.575201
H	-4.068024	2.299034	-1.618443
H	2.506059	0.161338	-2.913916
C	-4.441195	4.303998	-2.176601
C	3.602374	1.028421	-4.510612
H	-4.554613	4.075373	-3.248040
H	3.625187	0.106413	-5.113190
H	-5.441926	4.328239	-1.720623
H	4.540952	1.088429	-3.944521
C	-2.165175	3.198956	-2.148003
C	1.092140	0.850427	-4.352830
H	-1.658482	4.174019	-2.094981
H	0.955147	1.636559	-5.112334
H	-1.472557	2.494730	-1.653142
H	0.223921	0.854491	-3.675357
C	-3.183009	-2.736792	-0.584441
C	3.517669	-2.199730	1.402213
C	-2.169108	-3.131742	0.319426
C	3.324361	-1.976262	2.786016
C	-1.423698	-4.279738	0.032880
C	2.795978	-3.005474	3.570812
H	-0.645810	-4.597027	0.730523
H	2.647246	-2.842781	4.641054
C	-1.649398	-5.011530	-1.129072
C	2.452486	-4.231748	3.010333
H	-1.060587	-5.908233	-1.335202
H	2.049301	-5.029728	3.638570
C	-2.599944	-4.572831	-2.049542
C	2.599678	-4.428090	1.638856
H	-2.738160	-5.125992	-2.980899
H	2.299866	-5.382343	1.201976
C	-3.364941	-3.429708	-1.806140
C	3.114229	-3.424974	0.812386
C	-1.900514	-2.332959	1.579863
C	3.623650	-0.619349	3.394206
H	-2.317688	-1.325675	1.416829
H	4.255218	-0.073229	2.678717
C	-0.409734	-2.174663	1.870516
C	2.328267	0.181854	3.559450
H	0.049493	-3.105083	2.233812
H	1.637633	-0.324234	4.252296
H	0.128554	-1.876914	0.958418
H	1.790608	0.302878	2.605784

C -2.654260 -2.920573 2.775448
C 4.389648 -0.704571 4.713608
H -2.451218 -2.339471 3.688086
H 4.677159 0.301259 5.057574
H -3.741111 -2.915587 2.606388
H 5.306208 -1.304204 4.606104
C -4.305712 -2.872508 -2.858983
C 3.245335 -3.629609 -0.686688
H -5.069705 -2.276011 -2.339316
H 3.059419 -2.645323 -1.148806
C -3.540643 -1.913327 -3.778112
C 2.210864 -4.600344 -1.247276
H -2.765216 -2.448448 -4.347566
H 2.398500 -5.635940 -0.920730
H -4.214336 -1.413934 -4.492165
H 2.245731 -4.599665 -2.347080
C -5.030550 -3.943067 -3.671212
C 4.660639 -4.051666 -1.092304
H -5.776165 -3.481992 -4.337184
H 4.714660 -4.221302 -2.179519
H -4.339147 -4.517079 -4.307794
H 4.948801 -4.988189 -0.587965
H -5.553070 -4.656758 -3.016249
H 5.403315 -3.283764 -0.841190
H -3.012536 -1.131937 -3.206216
H 1.194689 -4.318576 -0.941531
H -4.010656 5.314188 -2.095533
H 3.565062 1.887875 -5.198835
H -2.238741 2.920246 -3.211373
H 1.107225 -0.112941 -4.883639
H -2.342173 -3.961249 2.958922
H 3.784496 -1.161326 5.512667
H -0.251774 -1.409868 2.644239
H 2.531359 1.190413 3.950935
H -5.416553 2.531014 4.441907
H 4.928628 5.675212 0.045305
H -1.748596 1.579483 4.040405
H 2.787746 3.061357 1.862531
N 0.495471 -1.231644 -1.395220
C -0.187107 -0.128153 -1.216651
C 0.223384 -2.033708 -2.561471
H 1.164859 -2.329533 -3.053452
H -0.295759 -2.962313 -2.274412
H -0.410699 -1.498277 -3.290316
N 0.532292 1.585667 0.398853
C -0.296440 0.692613 -0.078747
C 0.073813 2.533262 1.381796
H 0.606260 2.400469 2.340052
H 0.287616 3.559207 1.038367
H -1.008763 2.454861 1.569949

164
LCa-(MeNC-CNMe)-CaL + MeNC (G)
Ca 2.526934 0.967139 0.498597
Ca -1.990046 0.006499 -0.687684
N 3.461050 -0.565565 2.013837
N -3.488110 -1.749077 -1.146467
N 4.480922 0.407560 -0.661161
N -3.989681 1.248147 -0.891553
C 5.146708 -1.889284 3.193355
C -5.411338 -2.916345 -2.104689
H 4.914072 -1.472153 4.183163
H -5.563755 -3.535793 -1.207780
H 6.217086 -2.122573 3.145833
H -6.386892 -2.701649 -2.557069
H 4.576403 -2.827827 3.116085
H -4.822619 -3.529909 -2.802161
C 4.728473 -0.950080 2.087247
C -4.671292 -1.651041 -1.738710
C 5.736749 -0.570148 1.170948
C -5.321543 -0.433430 -2.039531
H 6.745467 -0.879429 1.445836
H -6.248008 -0.540983 -2.604157
C 5.620061 -0.016500 -0.119752
C -5.058414 0.878789 -1.590820
C 6.886738 0.036277 -0.943444
C -6.131332 1.902038 -1.894224
H 6.944825 0.979344 -1.506270
H -6.792471 2.038459 -1.023884
H 6.902073 -0.773896 -1.688364
H -5.692711 2.885264 -2.112917
H 7.777863 -0.066417 -0.311306
H -6.752773 1.584052 -2.741139
C 2.507004 -1.076831 2.929765
C -2.964076 -3.018095 -0.803457
C 2.338579 -0.477113 4.202574
C -3.090539 -3.483293 0.527325
C 1.340507 -0.963679 5.052714
C -2.514501 -4.709661 0.874710
H 1.204619 -0.504771 6.035319
H -2.616869 -5.076474 1.899476
C 0.508540 -2.011151 4.665900
C -1.807080 -5.464322 -0.056151
H -0.271507 -2.373361 5.339556
H -1.358940 -6.417840 0.232973
C 0.669482 -2.582737 3.408306
C -1.663234 -4.987264 -1.356008
H 0.011940 -3.397107 3.096655
H -1.091248 -5.571263 -2.081411
C 1.657989 -2.134892 2.527017
C -2.230734 -3.772505 -1.751515
C 3.184883 0.706602 4.635624
C -3.819564 -2.666207 1.576708
H 3.996098 0.816151 3.900448
H -4.124771 -1.726927 1.091439
C 3.825445 0.507612 6.010420
C -5.092538 -3.365898 2.056672
H 4.504462 1.342225 6.245487

H -5.637265 -2.734738 2.776804
H 3.068964 0.465155 6.809736
H -4.862913 -4.320542 2.557068
C 2.358280 1.994693 4.598340
C -2.891577 -2.314160 2.740800
H 2.977915 2.872525 4.842068
H -3.411527 -1.693149 3.485593
H 1.918923 2.155191 3.604069
H -2.008886 -1.757636 2.391883
C 1.802001 -2.799162 1.171139
C -2.011052 -3.258391 -3.162577
H 2.625040 -2.290861 0.645837
H -2.682574 -2.398869 -3.307071
C 2.188028 -4.273862 1.309754
C -2.350454 -4.298662 -4.231158
H 2.362326 -4.722559 0.319270
H -2.277118 -3.856497 -5.237035
H 3.104519 -4.397055 1.906570
H -3.371036 -4.691136 -4.105532
C 0.533700 -2.641616 0.332566
C -0.576786 -2.750902 -3.326578
H -0.345448 -3.064360 0.837987
H 0.148545 -3.563562 -3.165814
H 0.340967 -1.577720 0.132445
H -0.340157 -1.954277 -2.606637
C 4.395812 0.603458 -2.056482
C -3.988256 2.498087 -0.233275
C 4.215546 1.902679 -2.583277
C -4.759767 2.665441 0.946945
C 4.060884 2.064906 -3.964663
C -4.708127 3.885577 1.624568
H 3.927947 3.069716 -4.374239
H -5.297470 4.018390 2.534643
C 4.073125 0.972354 -4.823356
C -3.923044 4.938733 1.159049
H 3.953163 1.115006 -5.899824
H -3.911798 5.893703 1.690749
C 4.221399 -0.309359 -4.296447
C -3.160760 4.763970 0.008663
H 4.198747 -1.170039 -4.969097
H -2.554748 5.592140 -0.367681
C 4.376018 -0.518985 -2.925002
C -3.164110 3.552095 -0.693432
C 4.169291 3.120291 -1.680172
C -5.570845 1.513029 1.512173
H 4.300115 2.761982 -0.644179
H -5.764033 0.802506 0.696457
C 2.815929 3.827084 -1.782028
C -4.740454 0.762790 2.554266
H 2.649476 4.221600 -2.796321
H -4.496518 1.414475 3.408658
H 1.980831 3.141695 -1.567374
H -3.794022 0.413293 2.119287
C 5.330128 4.079462 -1.950190
C -6.924226 1.938575 2.078652
H 5.310575 4.927693 -1.247589
H -7.515577 1.053210 2.359296
H 6.298499 3.568317 -1.843652
H -7.505585 2.517630 1.344660
C 4.391111 -1.932827 -2.372543
C -2.329619 3.413463 -1.951543
H 4.762845 -1.892404 -1.339251
H -2.281439 2.344250 -2.207284
C 2.955049 -2.457531 -2.307481
C -0.889125 3.877726 -1.747241
H 2.521507 -2.537214 -3.316544
H -0.827588 4.961796 -1.555725
H 2.915131 -3.452365 -1.837080
H -0.292194 3.667609 -2.645960
C 5.298235 -2.883326 -3.152205
C -2.990357 4.126951 -3.133791
H 5.346959 -3.862832 -2.651516
H -2.398222 3.989265 -4.052428
H 4.929283 -3.060169 -4.174909
H -3.078931 5.209519 -2.945560
H 6.324003 -2.491445 -3.232171
H -4.001337 3.736883 -3.322353
H 2.313525 -1.778346 -1.728063
H -0.421349 3.342539 -0.907242
H 1.387591 -4.852523 1.797317
H -1.659804 -5.156107 -4.199728
H 0.634961 -3.159400 -0.629794
H -0.412003 -2.345921 -4.337075
H 5.279255 4.488702 -2.971607
H -6.819682 2.557029 2.984215
H 2.753742 4.671239 -1.078884
H -5.283347 -0.115509 2.936128
H 4.406577 -0.425752 6.056220
H -5.770286 -3.583830 1.217623
H 1.526790 1.951563 5.319902
H -2.537143 -3.219036 3.259219
N -0.161265 0.773103 -1.991652
C 0.418879 0.671456 -0.811984
C 0.646555 0.639298 -3.177715
H 0.082318 0.131785 -3.975961
H 0.926898 1.632603 -3.574554
H 1.588652 0.091440 -2.997959
N -0.799646 0.447077 1.324889
C -0.028055 1.028696 0.443747
C -0.855273 0.961111 2.671804
H -0.473573 1.995812 2.739368
H -1.889214 0.940358 3.046750
H -0.254957 0.330511 3.348147
N 1.084585 4.185285 1.561001
C 1.828549 3.314311 1.329481
C -0.030560 5.020708 1.764445
H 0.040998 5.906561 1.117931
H -0.949714 4.467459 1.512118

H -0.071475 5.345087 2.813912

164
TS LCa-(MeNC-CNMe)-CaL + MeNC (H*)
Ca -2.500957 0.615403 -0.861718
Ca 2.090903 0.177249 0.425650
N -3.772438 -1.292435 -1.305110
N 3.412190 -1.341454 1.624329
N -4.398718 1.088257 0.412340
N 4.197679 1.201131 0.189075
C -5.679852 -2.802565 -1.531454
C 5.225985 -2.242130 3.006730
H -5.509910 -3.001169 -2.599253
H 5.021012 -3.252338 2.624110
H -6.757008 -2.835836 -1.328566
H 6.308602 -2.117153 3.130670
H -5.190901 -3.624574 -0.985378
H 4.757425 -2.185923 4.001558
C -5.069811 -1.486499 -1.114487
C 4.646160 -1.186449 2.092414
C -5.935083 -0.546050 -0.510060
C 5.484759 -0.094349 1.791295
H -6.992019 -0.812902 -0.530173
H 6.460831 -0.120016 2.276203
C -5.627280 0.607832 0.238544
C 5.316349 0.952908 0.859440
C -6.788628 1.299155 0.916356
C 6.548527 1.786455 0.584747
H -6.682070 2.391559 0.848075
H 7.078108 1.392986 -0.298088
H -6.821470 1.054145 1.989080
H 6.294067 2.830589 0.359727
H -7.745167 0.998690 0.470304
H 7.244998 1.754543 1.432484
C -2.960720 -2.301836 -1.875693
C 2.701764 -2.536343 1.876141
C -2.870616 -2.441418 -3.283320
C 2.618318 -3.502540 0.841741
C -1.980499 -3.382632 -3.810451
C 1.899586 -4.678277 1.076541
H -1.902722 -3.494730 -4.894686
H 1.841572 -5.438352 0.295187
C -1.180423 -4.166313 -2.982785
C 1.246985 -4.893802 2.288570
H -0.483689 -4.888609 -3.414443
H 0.689765 -5.818598 2.454790
C -1.265258 -4.012987 -1.602651
C 1.289059 -3.915527 3.277067
H -0.631500 -4.617894 -0.949922
H 0.748604 -4.075582 4.213120
C -2.149476 -3.094253 -1.029083
C 2.004853 -2.728603 3.091946
C -3.682188 -1.561643 -4.217160
C 3.310163 -3.252378 -0.486000
H -4.427775 -1.032979 -3.604812
H 3.288773 -2.162422 -0.648297
C -4.438579 -2.368585 -5.274247
C 4.788059 -3.650146 -0.440853
H -5.095860 -1.710633 -5.863832
H 5.269630 -3.463406 -1.413913
H -3.751567 -2.861591 -5.979939
H 4.897618 -4.721327 -0.207080
C -2.792404 -0.498263 -4.867583
C 2.596485 -3.902075 -1.666918
H -3.382963 0.162774 -5.521697
H 3.053768 -3.575210 -2.613010
H -2.296495 0.133554 -4.115443
H 1.533143 -3.625855 -1.690579
C -2.209601 -2.948801 0.478898
C 1.981021 -1.644785 4.154027
H -3.054455 -2.281122 0.707743
H 2.783154 -0.932234 3.911590
C -2.477781 -4.283430 1.176191
C 2.241212 -2.180659 5.561891
H -2.607539 -4.130561 2.259001
H 2.328024 -1.350936 6.280560
H -3.386956 -4.765507 0.785711
H 3.170186 -2.769214 5.605419
C -0.932379 -2.300555 1.015201
C 0.660847 -0.871579 4.096006
H -0.041336 -2.871842 0.722313
H -0.192679 -1.530137 4.320229
H -0.805105 -1.275357 0.632877
H 0.484438 -0.438445 3.100545
C -4.140621 2.024659 1.435732
C 4.212920 2.151799 -0.857643
C -3.730628 3.338438 1.110637
C 4.738021 1.788054 -2.123047
C -3.419729 4.230641 2.143410
C 4.716231 2.726261 -3.157870
H -3.114965 5.250340 1.894784
H 5.118754 2.456097 -4.136748
C -3.489767 3.843006 3.475882
C 4.194704 4.003067 -2.961253
H -3.246635 4.552406 4.270365
H 4.198889 4.729781 -3.777313
C -3.852662 2.534095 3.789816
C 3.656550 4.342627 -1.724167
H -3.872584 2.221536 4.836263
H 3.236158 5.339911 -1.578047
C -4.171935 1.609856 2.793852
C 3.637702 3.430306 -0.663022
C -3.618173 3.800144 -0.330051
C 5.245391 0.378786 -2.369311
H -3.808395 2.922738 -0.973543
H 5.502298 -0.060497 -1.394758
C -2.218089 4.332561 -0.643337
C 4.116635 -0.472963 -2.952577

H -2.008511 5.257962 -0.084865
H 3.791644 -0.084543 -3.930811
H -1.441570 3.601113 -0.370472
H 3.236282 -0.466372 -2.294564
C -4.704920 4.818143 -0.683225
C 6.495743 0.321902 -3.244399
H -4.640018 5.110035 -1.743228
H 6.882950 -0.707757 -3.290611
H -5.708461 4.405664 -0.501067
H 7.293781 0.968901 -2.849229
C -4.416351 0.154957 3.153551
C 3.047752 3.823182 0.678350
H -4.952731 -0.319501 2.320310
H 2.781947 2.890873 1.199485
C -3.068662 -0.560975 3.271164
C 1.761217 4.633747 0.540907
H -2.478181 -0.152854 4.106402
H 1.939085 5.627279 0.099619
H -3.201952 -1.641529 3.438014
H 1.308196 4.794950 1.530403
C -5.261882 -0.035550 4.410902
C 4.077908 4.550013 1.546932
H -5.493282 -1.101931 4.557713
H 3.643593 4.820415 2.522623
H -4.739900 0.309287 5.317446
H 4.421681 5.476636 1.059021
H -6.214063 0.513251 4.345383
H 4.959422 3.921174 1.735339
H -2.474309 -0.429950 2.356370
H 1.025971 4.102113 -0.079728
H -1.638541 -4.983555 1.045261
H 1.421719 -2.828678 5.910597
H -0.950770 -2.250779 2.111076
H 0.652130 -0.046632 4.825438
H -4.602320 5.730355 -0.074202
H 6.291730 0.637261 -4.279867
H -2.110756 4.550514 -1.715847
H 4.431383 -1.519561 -3.084720
H -5.062511 -3.152266 -4.817943
H 5.337579 -3.075066 0.316386
H -2.003594 -0.964229 -5.479655
H 2.668274 -5.001075 -1.640102
N 0.402062 1.662737 1.197998
C -0.258583 1.083375 0.232588
C -0.329992 2.194923 2.322987
H 0.255257 2.081620 3.248308
H -0.509732 3.274887 2.186547
H -1.317056 1.719237 2.455082
N 0.669059 -0.469447 -1.412148
C 0.102145 0.648174 -1.031815
C 0.594974 -0.888533 -2.794170
H 0.644690 -0.048567 -3.507564
H 1.411580 -1.587799 -3.014868
H -0.343941 -1.435266 -2.981271
N -0.417984 2.490673 -3.198841
C -1.136855 1.778946 -2.543166
C 0.960401 2.844086 -3.223304
H 1.076434 3.927791 -3.083363
H 1.505724 2.320454 -2.415644
H 1.407214 2.586550 -4.194660

164
LCa-(MeNC-CNMe-CNMe)-CaL (I)
Ca -3.131943 -0.076247 -0.144168
Ca 3.018593 0.532194 0.834209
N -4.266592 -1.982815 -0.838186
N 4.993231 -0.698030 0.589708
N -5.327457 0.379780 0.432070
N 4.169485 1.890291 -0.713098
C -5.927689 -3.655224 -1.473479
C 7.410723 -1.092736 0.488110
H -5.510030 -3.745513 -2.488120
H 7.377091 -1.482602 1.516077
H -7.015674 -3.786688 -1.520158
H 7.454623 -1.970216 -0.175171
H -5.498267 -4.483860 -0.889582
H 8.332274 -0.513259 0.349944
C -5.546252 -2.326053 -0.869679
C 6.184846 -0.253759 0.202512
C -6.583334 -1.515271 -0.364697
C 6.395330 0.947925 -0.502313
H -7.591508 -1.919933 -0.453182
H 7.432504 1.146614 -0.774213
C -6.475824 -0.257703 0.265431
C 5.454339 1.857352 -1.038611
C -7.742702 0.383096 0.774948
C 5.978860 2.804663 -2.093829
H -7.909040 1.354938 0.285123
H 5.759557 2.389834 -3.091303
H -7.652892 0.594023 1.851735
H 5.492481 3.788240 -2.048223
H -8.620790 -0.252540 0.607749
H 7.066555 2.929306 -2.015937
C -3.240264 -2.857719 -1.243441
C 4.829069 -2.045574 0.973539
C -2.596813 -2.644666 -2.486194
C 4.879685 -3.066106 -0.013372
C -1.478580 -3.419099 -2.812493
C 4.656033 -4.388652 0.371924
H -0.972684 -3.256201 -3.767868
H 4.696792 -5.180774 -0.378595
C -0.999940 -4.391357 -1.940536
C 4.365369 -4.718817 1.695279
H -0.123286 -4.985916 -2.207261
H 4.197019 -5.760747 1.977905
C -1.633096 -4.589909 -0.714778
C 4.272967 -3.710410 2.646546
H -1.236815 -5.337513 -0.023891

H 4.023943 -3.966152 3.679581
C -2.745242 -3.833310 -0.340890
C 4.492269 -2.370509 2.306317
C -3.095087 -1.570605 -3.435855
C 5.057531 -2.705878 -1.478155
H -4.022888 -1.167109 -2.999585
H 5.652654 -1.783548 -1.535530
C -3.450596 -2.133763 -4.812662
C 5.788207 -3.768211 -2.295178
H -3.876546 -1.349198 -5.457788
H 5.987603 -3.396079 -3.312068
H -2.563710 -2.539338 -5.324892
H 5.195717 -4.690850 -2.400664
C -2.086682 -0.422498 -3.551757
C 3.691950 -2.377256 -2.086231
H -2.488714 0.402066 -4.161054
H 3.794370 -2.031006 -3.126771
H -1.797230 -0.005160 -2.572088
H 3.190397 -1.586984 -1.511589
C -3.358202 -3.978732 1.040145
C 4.351092 -1.298423 3.370608
H -4.356640 -3.519486 1.007024
H 4.471392 -0.321676 2.867380
C -3.534284 -5.434176 1.470329
C 5.476973 -1.373542 4.404372
H -4.073035 -5.488911 2.429022
H 5.386323 -0.563940 5.146074
H -4.106017 -6.007704 0.724632
H 6.463493 -1.290517 3.924388
C -2.540846 -3.191955 2.070641
C 2.969226 -1.342370 4.028153
H -1.534686 -3.623142 2.191597
H 2.838965 -2.267415 4.610907
H -2.394897 -2.141435 1.772626
H 2.167359 -1.310426 3.274286
C -5.247157 1.651325 1.033691
C 3.306385 2.817604 -1.338910
C -5.395278 2.814890 0.236998
C 2.690403 2.499814 -2.571351
C -5.181026 4.064098 0.822997
C 1.843694 3.439569 -3.166479
H -5.291975 4.967454 0.218131
H 1.373365 3.205563 -4.125064
C -4.814340 4.178731 2.162646
C 1.575328 4.657868 -2.550676
H -4.649774 5.164545 2.604010
H 0.915197 5.384263 -3.030717
C -4.642527 3.031026 2.929988
C 2.126293 4.932237 -1.301379
H -4.334598 3.123179 3.974753
H 1.880785 5.872851 -0.804907
C -4.848144 1.758353 2.387533
C 2.983209 4.025022 -0.670425
C -5.691091 2.692224 -1.246651
C 2.853189 1.125550 -3.190392
H -6.066082 1.673230 -1.422147
H 3.663915 0.614578 -2.650453
C -4.397250 2.844837 -2.055808
C 1.566355 0.323605 -2.968180
H -3.988356 3.862640 -1.954644
H 0.709985 0.823286 -3.446048
H -3.602544 2.164623 -1.707402
H 1.326437 0.226890 -1.898129
C -6.761789 3.667861 -1.732707
C 3.233351 1.167837 -4.669614
H -7.006424 3.478112 -2.789475
H 3.422189 0.151100 -5.048374
H -7.687549 3.569381 -1.145807
H 4.141991 1.766382 -4.833914
C -4.605327 0.518278 3.228322
C 3.559711 4.322685 0.702240
H -4.875195 -0.349101 2.605137
H 3.732562 3.347939 1.187610
C -3.125110 0.386117 3.603606
C 2.595692 5.104750 1.590988
H -2.795408 1.225699 4.235202
H 2.451795 6.137861 1.237166
H -2.942976 -0.544979 4.162020
H 2.993923 5.170510 2.614961
C -5.500957 0.482332 4.468278
C 4.917893 5.024164 0.614760
H -5.358422 -0.458035 5.023590
H 5.307922 5.241988 1.621791
H -5.271351 1.311335 5.156579
H 4.830916 5.978178 0.070047
H -6.564565 0.559864 4.196319
H 5.661235 4.403513 0.097413
H -2.458918 0.378158 2.724047
H 1.610806 4.616716 1.635437
H -2.567128 -5.941607 1.612445
H 5.448510 -2.331837 4.946896
H -3.035200 -3.196756 3.054628
H 2.833176 -0.496898 4.718699
H -6.429223 4.715210 -1.658219
H 2.431636 1.603054 -5.287318
H -4.567898 2.647901 -3.125961
H 1.645675 -0.691463 -3.385182
H -4.188262 -2.945822 -4.730008
H 6.751882 -4.039296 -1.836816
H -1.151909 -0.764920 -4.021180
H 3.032545 -3.259308 -2.077697
N 1.808088 1.633735 2.531614
C 0.763753 0.961307 2.212001
C 1.901993 2.215301 3.856068
H 2.868320 1.945335 4.312883
H 1.879465 3.313955 3.781445
H 1.079027 1.882966 4.510650
N 1.196756 -0.889494 0.756769

C 0.437065 0.162538 1.086520
C 0.628784 -1.991590 0.019968
H 1.326284 -2.841580 0.046814
H -0.334648 -2.318600 0.443805
H 0.432292 -1.762915 -1.043848
N -1.284548 1.317732 -0.238684
C -0.907070 0.329353 0.504925
C -0.456879 2.454683 -0.580254
H 0.604324 2.325241 -0.308203
H -0.512779 2.654754 -1.659949
H -0.835636 3.351609 -0.064144

164
TS LCa-(MeNC-CNMe-CNMe)-CaL ring closure (J*)
Ca -2.626112 -0.048517 0.373953
Ca 2.188096 -0.143474 0.794176
N -4.088742 0.638450 -1.318054
N 4.061811 -0.918664 -0.390677
N -3.547184 -2.003178 -0.481814
N 3.503634 1.807535 0.859571
C -5.850475 0.883399 -2.990971
C 6.458143 -1.291358 -0.739035
H -5.234148 1.533805 -3.628791
H 6.511298 -2.257759 -0.215904
H -6.481380 1.553462 -2.385160
H 6.314159 -1.526906 -1.804042
H -6.499734 0.271137 -3.629009
H 7.416202 -0.772334 -0.615319
C -4.992278 0.038519 -2.080645
C 5.303702 -0.467403 -0.214697
C -5.223734 -1.349708 -2.059254
C 5.641927 0.754208 0.401228
H -6.013781 -1.721292 -2.712287
H 6.712746 0.949537 0.463061
C -4.519199 -2.320345 -1.310584
C 4.823174 1.851238 0.765528
C -4.886123 -3.768714 -1.517929
C 5.544275 3.161691 0.984356
H -4.638923 -4.375263 -0.635505
H 5.042608 3.783123 1.738170
H -4.308541 -4.173829 -2.365011
H 6.588458 2.995456 1.279750
H -5.951919 -3.885425 -1.756279
H 5.550655 3.740728 0.047057
C -3.694528 1.974165 -1.497923
C 3.840656 -2.036085 -1.229143
C -4.082187 2.948000 -0.547168
C 3.444387 -1.807502 -2.572023
C -3.517645 4.226743 -0.614856
C 3.230944 -2.908893 -3.405152
H -3.804015 4.979922 0.123849
H 2.940841 -2.748910 -4.444761
C -2.602959 4.552215 -1.608459
C 3.355124 -4.212200 -2.928999
H -2.164429 5.551797 -1.647826
H 3.179191 -5.059437 -3.595946
C -2.238422 3.593159 -2.554835
C 3.673681 -4.426453 -1.592953
H -1.515331 3.862352 -3.325158
H 3.733047 -5.449054 -1.211674
C -2.756465 2.296917 -2.519326
C 3.917076 -3.354756 -0.726560
C -5.100720 2.613937 0.527461
C 3.222808 -0.389164 -3.071097
H -5.417517 1.574339 0.349837
H 2.790268 0.170617 -2.224212
C -6.347444 3.493905 0.407362
C 4.530420 0.319230 -3.438145
H -7.114537 3.185493 1.135133
H 4.323376 1.329222 -3.825834
H -6.112628 4.552998 0.599382
H 5.071235 -0.238823 -4.219393
C -4.502769 2.689891 1.935396
C 2.230735 -0.314541 -4.228872
H -5.252200 2.410974 2.692527
H 1.978749 0.732874 -4.447553
H -3.638937 2.018020 2.070823
H 1.294557 -0.840145 -3.993711
C -2.299382 1.233413 -3.508081
C 4.181868 -3.623453 0.743172
H -3.204287 0.731932 -3.890282
H 4.502352 -2.672637 1.197534
C -1.557433 1.803992 -4.713748
C 5.293869 -4.647511 0.974559
H -1.358210 1.005056 -5.443565
H 5.520723 -4.738143 2.048393
H -2.136678 2.590075 -5.221793
H 6.220151 -4.362956 0.453427
C -1.449849 0.147622 -2.835913
C 2.885569 -4.060520 1.432272
H -0.605120 0.571177 -2.273190
H 2.489741 -4.977340 0.967463
H -2.061788 -0.462721 -2.157377
H 2.098654 -3.294088 1.356183
C -2.895835 -2.882569 0.399475
C 2.765643 3.002976 1.015753
C -3.434355 -3.046744 1.706686
C 2.492185 3.814399 -0.111679
C -2.636421 -3.667567 2.670381
C 1.730643 4.973929 0.058868
H -3.008520 -3.783749 3.688721
H 1.512329 5.601804 -0.808557
C -1.364946 -4.150265 2.358213
C 1.228643 5.332641 1.307058
H -0.756515 -4.620406 3.134035
H 0.638695 6.244944 1.422390
C -0.884948 -4.051027 1.058801
C 1.460008 4.503818 2.400919

H 0.095347 -4.460442 0.809818
H 1.039589 4.768341 3.373547
C -1.630177 -3.415217 0.062285
C 2.209647 3.328718 2.275998
C -4.866679 -2.604954 2.003287
C 2.920172 3.394302 -1.504979
H -5.490639 -3.055533 1.210862
H 3.615059 2.548660 -1.399435
C -5.103712 -1.088065 1.925657
C 1.700004 2.887987 -2.280551
H -4.371188 -0.541706 2.536906
H 0.941094 3.679960 -2.380138
H -5.072067 -0.724887 0.887831
H 1.225579 2.041255 -1.762524
C -5.388505 -3.136461 3.336162
C 3.646147 4.501897 -2.268132
H -6.458385 -2.902149 3.442805
H 4.019477 4.124014 -3.232884
H -5.272359 -4.227417 3.417426
H 4.505360 4.889343 -1.699840
C -1.109789 -3.338032 -1.359419
C 2.416510 2.424755 3.475236
H -1.828396 -2.728881 -1.928013
H 2.698002 1.432107 3.091380
C 0.255307 -2.659705 -1.461648
C 1.139405 2.236928 4.293088
H 1.044958 -3.258153 -0.981940
H 0.856721 3.152981 4.835675
H 0.549719 -2.536463 -2.512709
H 1.279084 1.441079 5.039802
C -1.074488 -4.730388 -1.997387
C 3.578390 2.905300 4.347910
H -0.771710 -4.666038 -3.054551
H 3.730385 2.230077 5.205238
H -0.348102 -5.380382 -1.483870
H 3.382888 3.916106 4.741374
H -2.057245 -5.222773 -1.947301
H 4.517645 2.942216 3.777554
H 0.236352 -1.672610 -0.977048
H 0.294457 1.949408 3.650212
H -0.583215 2.228510 -4.424690
H 5.006243 -5.647990 0.615084
H -1.046814 -0.544658 -3.589560
H 3.048859 -4.270443 2.499977
H -4.865009 -2.673977 4.188190
H 2.981191 5.352905 -2.485669
H -6.105248 -0.837125 2.306158
H 1.985904 2.564493 -3.292763
H -6.784521 3.428197 -0.600271
H 5.192065 0.431304 -2.569506
H -4.154146 3.708732 2.167278
H 2.644603 -0.746730 -5.153673
N 1.529026 -0.884545 3.139267
C 0.293112 -0.914976 2.864220
C 2.115047 -1.863055 4.040801
H 2.283261 -1.390097 5.022700
H 1.472416 -2.748230 4.181395
H 3.100115 -2.178329 3.665408
N 0.014014 0.848225 0.184061
C -0.143067 -0.217737 0.958197
C -0.653485 2.120617 0.447013
H -1.089826 2.528254 -0.477280
H -1.426485 2.077156 1.239048
H 0.081128 2.863848 0.788466
N -1.998198 0.082895 2.648521
C -0.799561 -0.302795 2.220463
C -2.238816 -0.071450 4.053683
H -1.792030 -1.021352 4.424818
H -1.798460 0.742054 4.660507
H -3.319705 -0.084857 4.267967

164
Triimino deltate complex (K)
Ca -2.938284 0.157955 -0.542100
Ca 2.610290 0.574801 0.283811
N -3.750009 -2.000661 -0.884209
N 4.018593 -0.796952 1.556701
N -5.068325 0.269644 0.372276
N 4.714123 1.408156 -0.345272
C -4.991861 -4.104057 -0.767475
C 5.924313 -1.532140 2.907011
H -4.802729 -4.355684 -1.821903
H 5.513925 -1.298920 3.901720
H -5.981222 -4.481326 -0.481848
H 5.681760 -2.586970 2.713629
H -4.227056 -4.640017 -0.184206
H 7.014169 -1.415850 2.946658
C -4.870417 -2.618309 -0.534805
C 5.301612 -0.629967 1.866851
C -5.954559 -1.967066 0.081459
C 6.145410 0.334656 1.285838
H -6.824767 -2.588251 0.291594
H 7.170793 0.335816 1.656687
C -6.036420 -0.631178 0.519383
C 5.903184 1.205881 0.198563
C -7.308071 -0.225668 1.224447
C 7.123323 1.877774 -0.390019
H -7.804787 0.598542 0.690718
H 7.558781 1.236163 -1.173233
H -7.082055 0.157587 2.231256
H 6.876289 2.837830 -0.861903
H -8.008618 -1.064899 1.310265
H 7.895327 2.033344 0.375368
C -2.660048 -2.735110 -1.394518
C 3.309255 -1.918782 2.035657
C -2.541816 -2.953218 -2.789860
C 3.222450 -3.068244 1.208693
C -1.413810 -3.622630 -3.267633

C	2.500412	-4.175938	1.663216
H	-1.307203	-3.805701	-4.338427
H	2.451819	-5.075514	1.043933
C	-0.409603	-4.052710	-2.400302
C	1.853718	-4.158051	2.896912
H	0.469316	-4.567162	-2.794709
H	1.304393	-5.036197	3.243515
C	-0.518992	-3.805965	-1.036881
C	1.903363	-3.009338	3.680606
H	0.278458	-4.120519	-0.360834
H	1.375245	-2.989683	4.637449
C	-1.635634	-3.148902	-0.511775
C	2.617782	-1.879109	3.270772
C	-3.605081	-2.405976	-3.724963
C	3.934065	-3.111078	-0.132040
H	-4.566527	-2.452989	-3.190313
H	4.253568	-2.081030	-0.354427
C	-3.753949	-3.202445	-5.018749
C	5.200496	-3.968315	-0.065133
H	-4.621776	-2.841875	-5.591713
H	5.730277	-3.956858	-1.030957
H	-2.871581	-3.100394	-5.669979
H	4.958736	-5.016241	0.176352
C	-3.339979	-0.927616	-4.031389
C	3.008373	-3.564771	-1.258073
H	-4.105109	-0.514944	-4.706645
H	3.537013	-3.558730	-2.222373
H	-3.369669	-0.310570	-3.119207
H	2.137776	-2.899347	-1.343554
C	-1.721244	-2.869138	0.977458
C	2.624985	-0.634134	4.137952
H	-2.707345	-2.415006	1.167899
H	3.328088	0.076329	3.676760
C	-1.652929	-4.153756	1.804158
C	3.108153	-0.919479	5.561693
H	-1.762759	-3.931483	2.876902
H	3.200476	0.016806	6.134077
H	-2.449242	-4.855854	1.515364
H	4.088085	-1.419798	5.566095
C	-0.644873	-1.871773	1.416645
C	1.244681	0.028812	4.151492
H	0.366847	-2.266124	1.241082
H	0.485812	-0.647314	4.577579
H	-0.701622	-0.917267	0.866426
H	0.926873	0.314354	3.138185
C	-5.270210	1.577770	0.877891
C	4.578551	2.159848	-1.533210
C	-5.921457	2.548358	0.080866
C	4.880533	1.566866	-2.782598
C	-6.065179	3.843152	0.585533
C	4.669953	2.306705	-3.949203
H	-6.567096	4.602617	-0.018201
H	4.899724	1.856857	-4.918290
C	-5.565316	4.187350	1.840015
C	4.160257	3.600764	-3.899069
H	-5.682433	5.206404	2.215875
H	4.003315	4.166257	-4.820793
C	-4.902251	3.232141	2.604196
C	3.836093	4.165257	-2.668733
H	-4.497035	3.510695	3.580254
H	3.422415	5.174839	-2.636835
C	-4.747619	1.920645	2.144634
C	4.028636	3.464255	-1.473877
C	-6.378586	2.199042	-1.323104
C	5.336224	0.122506	-2.869772
H	-6.471695	1.103986	-1.371710
H	5.649631	-0.192490	-1.863968
C	-5.305533	2.606865	-2.339757
C	4.154330	-0.765188	-3.268947
H	-5.204743	3.703063	-2.382433
H	3.769576	-0.484944	-4.262451
H	-4.309457	2.220466	-2.069971
H	3.319704	-0.665023	-2.558670
C	-7.738073	2.793008	-1.686616
C	6.525612	-0.078118	-3.808228
H	-8.066832	2.425865	-2.671257
H	6.885024	-1.117945	-3.758046
H	-8.507163	2.520421	-0.947696
H	7.362681	0.585797	-3.543902
C	-3.995519	0.894072	2.972067
C	3.695621	4.098260	-0.135660
H	-4.165133	-0.085084	2.497291
H	3.429144	3.280967	0.553818
C	-2.489456	1.173737	2.942360
C	2.493148	5.035825	-0.197044
H	-2.255968	2.128622	3.438075
H	2.699654	5.937548	-0.795180
H	-1.921171	0.381620	3.452146
H	2.227291	5.375248	0.814494
C	-4.510890	0.796124	4.407714
C	4.917122	4.802954	0.461461
H	-3.997018	-0.013702	4.949018
H	4.670422	5.250093	1.437705
H	-4.335901	1.727846	4.968357
H	5.269224	5.608159	-0.203656
H	-5.591529	0.589858	4.430858
H	5.749513	4.102748	0.615186
H	-2.101403	1.267847	1.915454
H	1.614781	4.531445	-0.626873
H	-0.686453	-4.660667	1.670720
H	2.403849	-1.569109	6.105470
H	-0.731281	-1.645609	2.488285
H	1.258362	0.945174	4.761803
H	-7.705450	3.892312	-1.745681
H	6.260131	0.123993	-4.857979
H	-5.557160	2.247756	-3.350285
H	4.450500	-1.824493	-3.304160
H	-3.900774	-4.274027	-4.815566

H 5.894708 -3.598200 0.702743
H -2.352330 -0.786136 -4.495566
H 2.639103 -4.590120 -1.097278
N 1.234213 2.152464 1.361784
C 0.329408 1.813910 0.467575
C 0.904088 3.165235 2.331477
H 1.818394 3.503556 2.843301
H 0.427191 4.052402 1.877014
H 0.214151 2.791498 3.112898
N 0.900479 -0.014082 -1.247451
C 0.205855 0.929918 -0.635308
C 0.329488 -0.576721 -2.443827
H 0.978367 -1.368873 -2.842273
H -0.653369 -1.059771 -2.265273
H 0.189440 0.172369 -3.247059
N -2.081696 2.253833 -0.584847
C -0.835966 1.831031 -0.329522
C -2.408909 3.545879 -0.024259
H -1.855668 4.367919 -0.519518
H -3.481786 3.746794 -0.143061
H -2.186415 3.614727 1.059020

6
MeNC
N -0.308373 -0.000002 0.000020
C -1.482074 0.000001 0.000012
C 1.101126 0.000005 -0.000012
H 1.481715 -0.043210 1.031372
H 1.481280 -0.871678 -0.553170
H 1.481301 0.914866 -0.478344

158
LCa-(CN-Me)2-CaL (D) restricted singlet
Ca -2.183386 -0.118878 -0.168724
Ca 1.823085 -0.114599 0.329973
N -4.285693 -1.049425 -0.539579
N 3.406040 -1.736098 0.930398
N -3.305060 1.719747 -1.099497
N 3.637637 1.275601 0.827140
C -6.518375 -1.510390 -1.448119
C 5.115140 -2.739270 2.386278
H -6.598399 -2.262351 -0.651177
H 6.097682 -2.855127 1.902738
H -6.433621 -2.051665 -2.404279
H 5.298888 -2.592162 3.460148
H -7.444174 -0.920557 -1.485485
H 4.557166 -3.673304 2.239051
C -5.312236 -0.621274 -1.250019
C 4.379091 -1.548700 1.810947
C -5.356690 0.651565 -1.861779
C 4.826767 -0.282299 2.250899
H -6.256754 0.850533 -2.445221
H 5.600355 -0.315084 3.020728
C -4.468034 1.741699 -1.747111
C 4.564700 1.008238 1.733649
C -4.931395 3.035064 -2.382102
C 5.417086 2.125284 2.295399
H -4.092650 3.578149 -2.840054
H 6.456002 1.799160 2.441110
H -5.359797 3.705760 -1.620767
H 5.403208 3.012508 1.649016
H -5.702846 2.850207 -3.140527
H 5.027091 2.422861 3.281929
C -4.229445 -2.283613 0.126130
C 3.176714 -2.972466 0.298091
C -3.935833 -3.484953 -0.564388
C 4.078984 -3.455887 -0.682705
C -3.769172 -4.660348 0.174392
C 3.753429 -4.619296 -1.388368
H -3.545091 -5.592286 -0.351183
H 4.445480 -4.993139 -2.147560
C -3.868463 -4.666227 1.562158
C 2.559093 -5.296989 -1.160674
H -3.733975 -5.596265 2.119459
H 2.319858 -6.199314 -1.728325
C -4.120541 -3.475136 2.238787
C 1.668554 -4.809797 -0.206753
H -4.179730 -3.480392 3.328926
H 0.728408 -5.336361 -0.029424
C -4.294419 -2.274842 1.545940
C 1.956716 -3.659876 0.532371
C -3.746195 -3.505000 -2.069406
C 5.342602 -2.695454 -1.043125
H -4.026091 -2.511914 -2.449746
H 5.470324 -1.884412 -0.312872
C -4.643642 -4.539184 -2.751816
C 6.592566 -3.575227 -0.989574
H -4.560347 -4.462343 -3.847315
H 7.498912 -2.972072 -1.156330
H -4.362280 -5.567189 -2.473248
H 6.573031 -4.358479 -1.763972
C -2.272100 -3.720491 -2.425794
C 5.189812 -2.033233 -2.414262
H -2.130898 -3.725179 -3.518594
H 6.082864 -1.440527 -2.666671
H -1.642726 -2.917341 -2.011035
H 4.320972 -1.360620 -2.428818
C -4.588066 -0.976995 2.276666
C 0.998591 -3.182553 1.606121
H -4.202018 -0.156846 1.646667
H 1.198217 -2.113820 1.773628
C -6.097758 -0.741245 2.378762
C 1.303655 -3.866502 2.942588
H -6.312729 0.223552 2.865019
H 0.630735 -3.498669 3.733891
H -6.564350 -0.729422 1.383638
H 2.340194 -3.676644 3.257577
C -3.905338 -0.873033 3.636888

C -0.473034 -3.324166 1.229432
H -4.313396 -1.594799 4.361775
H -0.805988 -4.372351 1.220429
H -2.821468 -1.044903 3.549350
H -0.670974 -2.907525 0.230924
C -2.627101 2.933466 -0.853726
C 3.482266 2.539302 0.221682
C -1.412109 3.216701 -1.523153
C 4.041803 2.740398 -1.067458
C -0.784853 4.446674 -1.296041
C 3.831134 3.960661 -1.712874
H 0.143787 4.680371 -1.818202
H 4.268463 4.129666 -2.698765
C -1.316772 5.370700 -0.403087
C 3.058596 4.962663 -1.128177
H -0.820520 6.331806 -0.247788
H 2.904571 5.911362 -1.648116
C -2.458169 5.044764 0.325849
C 2.464705 4.736732 0.108291
H -2.837843 5.747790 1.070779
H 1.827922 5.506644 0.550200
C -3.115854 3.827942 0.132443
C 2.652894 3.533041 0.796196
C -0.809559 2.215674 -2.489722
C 4.846936 1.631371 -1.717340
H -1.106650 1.207034 -2.153082
H 4.440947 0.685506 -1.320333
C 0.716289 2.245968 -2.496771
C 4.713379 1.604240 -3.237883
H 1.116618 3.138497 -2.999833
H 5.196654 2.474018 -3.710285
H 1.127134 2.258443 -1.474584
H 3.657686 1.596750 -3.548899
C -1.381744 2.373048 -3.899801
C 6.317299 1.684110 -1.293281
H -0.945417 1.628901 -4.585109
H 6.885475 0.856721 -1.747064
H -2.471981 2.233913 -3.900369
H 6.423004 1.603634 -0.203232
C -4.265621 3.417248 1.034615
C 1.948222 3.307259 2.119034
H -4.839404 2.635011 0.517210
H 2.169246 2.271474 2.417943
C -3.697116 2.784436 2.310355
C 0.430794 3.452014 1.981305
H -3.117880 3.523046 2.887373
H 0.142547 4.496244 1.794861
H -4.502714 2.399087 2.955184
H -0.079755 3.126332 2.900499
C -5.229916 4.554414 1.364866
C 2.480302 4.233674 3.215737
H -6.093899 4.172404 1.930519
H 2.007512 4.004888 4.184427
H -4.756024 5.332036 1.984682
H 2.260926 5.287526 2.980782
H -5.608955 5.039971 0.452511
H 3.568797 4.140481 3.334509
H -3.014936 1.950672 2.079990
H 0.030674 2.859952 1.145975
H -6.576930 -1.538555 2.969265
H 1.169384 -4.957061 2.862087
H -4.053222 0.131545 4.060382
H -1.112145 -2.796213 1.953372
H -1.163570 3.376806 -4.298505
H 6.780987 2.632069 -1.610605
H 1.114905 1.365742 -3.021690
H 5.197835 0.706309 -3.648626
H -5.699901 -4.398987 -2.477333
H 6.689569 -4.077013 -0.015332
H -1.899229 -4.679480 -2.031997
H 5.045733 -2.783666 -3.207988
N 0.465648 0.135830 2.240295
C -0.547798 0.122319 1.529216
C 0.574475 0.236402 3.684529
H 1.272311 1.044748 3.945030
H -0.405796 0.422704 4.151729
H 0.997531 -0.702165 4.073126
N 0.959420 -1.100991 -1.617996
C -0.185278 -0.855367 -1.213048
C 1.372281 -1.849733 -2.793421
H 2.047422 -1.230180 -3.401686
H 1.931276 -2.738260 -2.464570
H 0.505340 -2.156466 -3.399203

158
LCa-(CN-Me)2-CaL (D) unrestricted singlet
Ca -2.183456 -0.118463 -0.168599
Ca 1.823131 -0.114411 0.330039
N -4.285543 -1.049525 -0.539644
N 3.406032 -1.735992 0.930389
N -3.305138 1.719711 -1.100003
N 3.637599 1.275624 0.827276
C -6.518155 -1.510764 -1.448160
C 5.115001 -2.739214 2.386401
H -6.598000 -2.262837 -0.651307
H 6.097950 -2.854535 1.903552
H -6.433367 -2.051912 -2.404387
H 5.297904 -2.592483 3.460473
H -7.444047 -0.921067 -1.485400
H 4.557434 -3.673362 2.238389
C -5.312154 -0.621472 -1.250026
C 4.378998 -1.548629 1.811037
C -5.356864 0.651407 -1.861740
C 4.826712 -0.282240 2.251028
H -6.257088 0.850294 -2.444956
H 5.600309 -0.315068 3.020851
C -4.468379 1.741650 -1.747128
C 4.564707 1.008297 1.733755

C -4.932015 3.035095 -2.381770
C 5.417157 2.125345 2.295379
H -4.093467 3.577894 -2.840452
H 6.456102 1.799236 2.440936
H -5.359580 3.706022 -1.620181
H 5.403203 3.012550 1.648976
H -5.704107 2.850394 -3.139577
H 5.027286 2.422928 3.281957
C -4.229168 -2.283751 0.126039
C 3.176726 -2.972297 0.297925
C -3.935097 -3.484955 -0.564519
C 4.078932 -3.455492 -0.683023
C -3.768210 -4.660349 0.174206
C 3.753333 -4.618721 -1.388972
H -3.543738 -5.592173 -0.351406
H 4.445378 -4.992414 -2.148246
C -3.867759 -4.666369 1.561958
C 2.558969 -5.296404 -1.161439
H -3.733099 -5.596413 2.119207
H 2.319682 -6.198556 -1.729343
C -4.120357 -3.475411 2.238625
C 1.668489 -4.809456 -0.207332
H -4.179813 -3.480739 3.328754
H 0.728327 -5.336030 -0.030120
C -4.294433 -2.275125 1.545816
C 1.956733 -3.659778 0.532127
C -3.745334 -3.504888 -2.069523
C 5.342559 -2.695006 -1.043254
H -4.025202 -2.511775 -2.449813
H 5.470218 -1.884040 -0.312909
C -4.642760 -4.539021 -2.752042
C 6.592538 -3.574762 -0.989683
H -4.559526 -4.462012 -3.847535
H 7.498889 -2.971563 -1.156262
H -4.361324 -5.567051 -2.473635
H 6.573109 -4.357923 -1.764183
C -2.271209 -3.720363 -2.425813
C 5.189841 -2.032679 -2.414348
H -2.129943 -3.725124 -3.518605
H 6.082968 -1.440089 -2.666764
H -1.641888 -2.917178 -2.011042
H 4.321097 -1.359940 -2.428850
C -4.588466 -0.977394 2.276594
C 0.998796 -3.182852 1.606212
H -4.202863 -0.157123 1.646485
H 1.198248 -2.114119 1.773894
C -6.098223 -0.742211 2.378979
C 1.304432 -3.867051 2.942427
H -6.313475 0.222508 2.865270
H 0.631730 -3.499514 3.734055
H -6.565016 -0.730585 1.383940
H 2.341050 -3.677084 3.257083
C -3.905525 -0.873166 3.636703
C -0.472914 -3.324643 1.229963
H -4.313113 -1.595156 4.361633
H -0.805658 -4.372897 1.220628
H -2.821590 -1.044528 3.548962
H -0.671341 -2.907602 0.231714
C -2.627251 2.933342 -0.853861
C 3.482153 2.539331 0.221800
C -1.412080 3.216700 -1.522999
C 4.041635 2.740467 -1.067328
C -0.784862 4.446608 -1.295544
C 3.830857 3.960742 -1.712716
H 0.143887 4.680371 -1.817466
H 4.268194 4.129836 -2.698589
C -1.316973 5.370519 -0.402570
C 3.058227 4.962647 -1.128003
H -0.820769 6.331612 -0.247018
H 2.904103 5.911330 -1.647942
C -2.458506 5.044471 0.326083
C 2.464365 4.736654 0.108483
H -2.838324 5.747383 1.071047
H 1.827506 5.506501 0.550391
C -3.116145 3.827665 0.132381
C 2.652694 3.532985 0.796363
C -0.809405 2.215804 -2.489651
C 4.846764 1.631478 -1.717284
H -1.105969 1.207075 -2.152781
H 4.440790 0.685618 -1.320250
C 0.716397 2.246671 -2.497288
C 4.713018 1.604365 -3.237807
H 1.116201 3.139380 -3.000479
H 5.196205 2.474154 -3.710280
H 1.127762 2.259186 -1.475308
H 3.657283 1.596846 -3.548682
C -1.382193 2.372757 -3.899537
C 6.317166 1.684157 -1.293377
H -0.945880 1.628652 -4.584898
H 6.885272 0.856848 -1.747395
H -2.472374 2.233219 -3.899622
H 6.422986 1.603444 -0.203350
C -4.265996 3.416824 1.034378
C 1.948181 3.307137 2.119286
H -4.839734 2.634643 0.516843
H 2.169309 2.271356 2.418128
C -3.697614 2.783852 2.310106
C 0.430728 3.451808 1.981711
H -3.118481 3.522406 2.887299
H 0.142383 4.496043 1.795437
H -4.503273 2.398353 2.954772
H -0.079752 3.125947 2.900877
C -5.230357 4.553911 1.364714
C 2.480352 4.233532 3.215948
H -6.094413 4.171768 1.930162
H 2.007699 4.004688 4.184692
H -4.756566 5.331419 1.984750
H 2.260911 5.287389 2.981082

H -5.609270 5.039643 0.452401
H 3.568867 4.140365 3.334596
H -3.015334 1.950161 2.079760
H 0.030596 2.859891 1.146287
H -6.576996 -1.539694 2.969573
H 1.170326 -4.957615 2.861718
H -4.053792 0.131333 4.060249
H -1.111897 -2.797105 1.954318
H -1.164534 3.376528 -4.298490
H 6.780831 2.632171 -1.610573
H 1.115104 1.366648 -3.022488
H 5.197441 0.706447 -3.648624
H -5.699010 -4.398919 -2.477480
H 6.689433 -4.076651 -0.015484
H -1.898332 -4.679317 -2.031939
H 5.045628 -2.783063 -3.208097
N 0.465769 0.135590 2.240537
C -0.547691 0.121984 1.529490
C 0.574661 0.235884 3.684832
H 1.271383 1.045165 3.945389
H -0.405827 0.420690 4.152156
H 0.999116 -0.702202 4.073035
N 0.959499 -1.100411 -1.617996
C -0.185213 -0.854985 -1.212870
C 1.372294 -1.848773 -2.793758
H 2.047132 -1.228827 -3.401958
H 1.931575 -2.737228 -2.465202
H 0.505273 -2.155548 -3.399388

158
LCa-(CN-Me)2-CaL (D) unrestricted triplet
Ca -1.937595 -0.077659 -0.313463
Ca 1.667324 -0.118900 0.162915
N -4.029560 -1.086794 -0.557601
N 3.214190 -1.726816 0.870593
N -3.165255 1.746806 -1.103841
N 3.419435 1.294894 0.839151
C -6.297539 -1.563956 -1.370913
C 4.697287 -2.746324 2.527673
H -6.393052 -2.249044 -0.517443
H 5.437476 -3.205307 1.854271
H -6.199043 -2.184958 -2.275651
H 5.204720 -2.479913 3.463292
H -7.219937 -0.977181 -1.470374
H 3.941928 -3.519434 2.730267
C -5.090800 -0.664765 -1.226858
C 4.065076 -1.539281 1.873562
C -5.182703 0.603296 -1.841672
C 4.461454 -0.278301 2.363116
H -6.102342 0.774048 -2.402633
H 5.148246 -0.311929 3.210261
C -4.336627 1.725938 -1.737363
C 4.249864 1.016595 1.830796
C -4.862639 3.002901 -2.355072
C 5.052849 2.119974 2.484895
H -4.054161 3.586115 -2.817489
H 6.081094 1.789777 2.687142
H -5.310724 3.647554 -1.582679
H 5.079605 3.028659 1.870175
H -5.634300 2.790170 -3.105827
H 4.598526 2.381158 3.453732
C -4.002450 -2.324514 0.116069
C 3.067588 -2.998446 0.277534
C -3.771966 -3.536935 -0.576580
C 4.037043 -3.457995 -0.649340
C -3.674156 -4.721721 0.160553
C 3.816634 -4.666605 -1.315697
H -3.500519 -5.664052 -0.365348
H 4.556647 -5.022635 -2.036780
C -3.780887 -4.723675 1.547653
C 2.663721 -5.417956 -1.092989
H -3.700932 -5.660535 2.104040
H 2.506041 -6.356386 -1.629875
C -3.973815 -3.521973 2.225143
C 1.715853 -4.961488 -0.182255
H -4.040864 -3.525891 3.314704
H 0.811758 -5.549386 -0.002989
C -4.078687 -2.312774 1.533235
C 1.901211 -3.764183 0.517167
C -3.587436 -3.567448 -2.082695
C 5.258421 -2.618204 -0.977554
H -3.849653 -2.571024 -2.467116
H 5.358919 -1.854082 -0.193846
C -4.504237 -4.588960 -2.758884
C 6.553303 -3.429784 -1.003646
H -4.433995 -4.505869 -3.854729
H 7.421142 -2.764886 -1.135891
H -4.228648 -5.621394 -2.491529
H 6.569553 -4.154826 -1.832802
C -2.120688 -3.813943 -2.447880
C 5.044013 -1.870142 -2.295068
H -1.990768 -3.838590 -3.541574
H 5.905115 -1.223799 -2.525164
H -1.471498 -3.017008 -2.056168
H 4.147122 -1.237057 -2.242481
C -4.307542 -1.003651 2.266710
C 0.865120 -3.311075 1.527648
H -3.858668 -0.205861 1.651137
H 1.186831 -2.324342 1.892000
C -5.801671 -0.678098 2.348454
C 0.813438 -4.238224 2.744224
H -5.965209 0.286949 2.853465
H 0.094445 -3.861210 3.488869
H -6.250304 -0.613491 1.347422
H 1.797975 -4.320041 3.228500
C -3.643055 -0.949089 3.638401
C -0.519764 -3.156068 0.903963
H -4.114466 -1.639815 4.355204

H -0.943414 -4.120116 0.589867
H -2.572852 -1.195265 3.568550
H -0.472630 -2.525316 0.003154
C -2.549264 2.994813 -0.854721
C 3.301182 2.590380 0.291270
C -1.380571 3.363414 -1.559793
C 3.925205 2.852137 -0.954928
C -0.815111 4.621851 -1.324013
C 3.756101 4.106813 -1.544139
H 0.081514 4.917360 -1.871299
H 4.242507 4.323078 -2.497335
C -1.372636 5.496534 -0.398520
C 2.964400 5.084039 -0.942953
H -0.927306 6.481118 -0.236306
H 2.840622 6.059709 -1.419391
C -2.477258 5.094860 0.350611
C 2.317689 4.803275 0.255599
H -2.878979 5.764696 1.114256
H 1.672077 5.558127 0.710469
C -3.067627 3.844867 0.155288
C 2.466856 3.564912 0.889491
C -0.756191 2.428644 -2.576389
C 4.763761 1.772578 -1.610690
H -1.115830 1.409941 -2.357301
H 4.328986 0.810602 -1.292550
C 0.766898 2.402256 -2.478689
C 4.732997 1.823207 -3.135617
H 1.228649 3.319680 -2.871352
H 5.255800 2.708906 -3.530261
H 1.101005 2.324876 -1.431485
H 3.700982 1.842820 -3.516849
C -1.225253 2.743054 -3.998179
C 6.201478 1.792161 -1.083820
H -0.775711 2.045363 -4.722485
H 6.796486 0.983956 -1.537871
H -2.318496 2.659473 -4.081236
H 6.229534 1.656470 0.005775
C -4.179667 3.360513 1.068236
C 1.716987 3.283158 2.176649
H -4.716896 2.554581 0.547871
H 1.959926 2.251335 2.468908
C -3.563987 2.744767 2.329801
C 0.203912 3.364996 1.971811
H -3.016167 3.505980 2.907673
H -0.121790 4.397104 1.781190
H -4.342471 2.315751 2.979962
H -0.330715 2.998455 2.860782
C -5.200027 4.440024 1.423262
C 2.153350 4.213525 3.311623
H -6.035754 4.004288 1.992426
H 1.654307 3.937077 4.254165
H -4.761923 5.233618 2.049080
H 1.885643 5.259290 3.091308
H -5.614846 4.916572 0.521770
H 3.239565 4.177823 3.475115
H -2.846138 1.946505 2.085128
H -0.129440 2.764192 1.113446
H -6.339247 -1.456376 2.913448
H 0.496139 -5.253747 2.458443
H -3.725977 0.063863 4.059428
H -1.229532 -2.710647 1.616845
H -0.939661 3.767631 -4.286052
H 6.690447 2.751086 -1.319800
H 1.177976 1.551812 -3.041505
H 5.234795 0.939399 -3.556815
H -5.555799 -4.442332 -2.470055
H 6.695507 -3.992551 -0.068300
H -1.761133 -4.773037 -2.042232
H 4.911683 -2.572974 -3.133321
N 0.557730 0.006827 2.333133
C -0.489901 0.124369 1.671795
C 0.745273 -0.094657 3.768562
H 1.407811 0.716980 4.104432
H -0.212258 -0.042975 4.313221
H 1.254556 -1.042915 3.997634
N 1.122865 -1.195327 -1.994856
C -0.019228 -0.804736 -1.688479
C 1.492243 -2.171205 -3.011051
H 2.212046 -1.717815 -3.707869
H 1.989961 -3.020481 -2.520760
H 0.611053 -2.525453 -3.569193

148
[(DIPP-BDI)Ca]2(N2)
N 2.172184 -2.607134 -0.000171
N -4.430477 0.822374 -0.000193
N 0.000360 0.000183 -0.617933
C 3.128274 -3.519586 -0.000620
C 2.741791 -4.978958 -0.001247
C 0.802202 -2.942890 -0.000160
C 0.097951 -2.994178 -1.226623
C -1.301714 -3.001044 -1.203449
H -1.851106 -3.027967 -2.147117
C -2.011268 -2.989528 -0.000135
H -3.104998 -3.013548 -0.000126
C -1.301712 -3.000700 1.203173
H -1.851092 -3.027374 2.146854
C 0.097953 -2.993741 1.226328
C 0.832914 -3.011540 -2.553257
H 1.908506 -2.970704 -2.323618
C 0.501818 -1.787860 -3.408095
H -0.554861 -1.789303 -3.720437
H 0.671603 -0.864094 -2.839050
C 0.565739 -4.310150 -3.319924
H -0.495017 -4.393323 -3.606716
H 1.163809 -4.348570 -4.244360

C	0.832964	-3.010758	2.552938
H	1.908498	-2.968511	2.323240
C	0.500329	-1.787947	3.408425
H	0.668776	-0.863694	2.839779
H	-0.556256	-1.791011	3.721070
C	0.567429	-4.310100	3.318955
H	0.817081	-5.195707	2.715167
H	1.165299	-4.348104	4.243540
C	-5.120099	1.953538	-0.000222
C	-6.626990	1.882781	0.000167
C	-5.013516	-0.450849	0.000305
C	-5.214434	-1.129072	1.230482
C	-5.593815	-2.474017	1.205939
H	-5.741954	-3.007809	2.148067
C	-5.781025	-3.149091	0.001220
H	-6.070041	-4.202475	0.001581
C	-5.594367	-2.474688	-1.203958
H	-5.742940	-3.008996	-2.145725
C	-5.215000	-1.129756	-1.229409
C	-4.982019	-0.403394	2.543500
H	-4.924932	0.669891	2.309534
C	-3.641834	-0.798669	3.170511
H	-3.619550	-1.871953	3.416400
H	-2.783911	-0.608734	2.505038
C	-6.128519	-0.603683	3.534991
H	-6.201242	-1.649114	3.874424
H	-5.978875	0.020872	4.429398
C	-4.983307	-0.404747	-2.542925
H	-4.926265	0.668667	-2.309542
C	-3.643373	-0.800115	-3.170412
H	-2.785156	-0.609621	-2.505478
H	-3.620984	-1.873529	-3.415724
C	-6.130261	-0.605718	-3.533752
H	-7.096783	-0.332759	-3.084034
H	-5.981175	0.018425	-4.428539
Ca	2.184214	-0.210696	0.000305
Ca	-2.183818	0.211000	-0.000593
N	4.430640	-0.822674	-0.000298
N	-2.172232	2.607304	-0.000789
N	0.000065	0.000250	0.617979
C	4.511164	-3.220405	-0.000636
H	5.184913	-4.077270	-0.000794
C	5.120164	-1.953875	-0.000297
C	6.627057	-1.883254	0.000265
C	5.013673	0.450567	0.000518
C	5.213722	1.128769	1.230853
C	5.593020	2.473750	1.206611
H	5.740516	3.007513	2.148861
C	5.780967	3.148871	0.002042
H	6.069914	4.202273	0.002623
C	5.595081	2.474506	-1.203285
H	5.744175	3.008877	-2.144930
C	5.215798	1.129560	-1.229042
C	4.980736	0.403022	2.543732

H	4.922868	-0.670179	2.309551
C	3.640899	0.799202	3.170924
H	3.619388	1.872476	3.416922
H	2.782701	0.609958	2.505588
C	6.127469	0.602298	3.535173
H	6.201034	1.647648	3.874674
H	5.977410	-0.022232	4.429529
C	4.984597	0.404671	-2.542697
H	4.928198	-0.668825	-2.309545
C	3.644372	0.799372	-3.169994
H	2.786321	0.608067	-2.505084
H	3.621316	1.872847	-3.414967
C	6.131336	0.606560	-3.533576
H	7.098093	0.334226	-3.083978
H	5.982600	-0.017588	-4.428416
C	-4.511286	3.220160	-0.000567
H	-5.185185	4.076908	-0.000590
C	-3.128458	3.519601	-0.000761
C	-2.742204	4.979031	-0.001034
C	-0.802273	2.943142	-0.000755
C	-0.097938	2.993876	-1.227197
C	1.301724	3.000711	-1.203943
H	1.851173	3.027216	-2.147591
C	2.011188	2.989639	-0.000575
H	3.104923	3.013620	-0.000498
C	1.301562	3.001276	1.202689
H	1.850889	3.028223	2.146397
C	-0.098114	2.994424	1.225764
C	-0.832861	3.010878	-2.553856
H	-1.908438	2.969499	-2.324222
C	-0.501121	1.787459	-3.408803
H	0.555565	1.789411	-3.721116
H	-0.670509	0.863554	-2.839867
C	-0.566329	4.309714	-3.320381
H	0.494430	4.393503	-3.606986
H	-1.164291	4.347887	-4.244899
C	-0.833189	3.011904	2.552332
H	-1.908740	2.970364	2.322612
C	-0.501454	1.788839	3.407797
H	-0.670610	0.864687	2.839193
H	0.555159	1.791081	3.720352
C	-0.566813	4.311025	3.318434
H	-0.815600	5.196838	2.714587
H	-1.164886	4.349479	4.242867
H	-0.493304	-4.394867	3.605371
H	0.813955	-5.196381	-2.716448
H	1.117209	-1.768320	-4.321639
H	6.203281	1.652249	-3.872385
H	3.456123	0.233415	-4.095088
H	7.093914	0.328679	3.085696
H	3.451475	0.232674	4.095425
H	-0.815143	5.195737	-2.716853
H	-1.116464	1.767728	-4.322376
H	0.493907	4.394961	3.605138

H	1.115892	-1.768010	4.321845
H	-1.116992	1.769363	4.321244
H	-3.452690	-0.232089	4.095037
H	-7.095217	-0.330774	3.085622
H	-6.202971	-1.651329	-3.872637
H	-3.454821	-0.233997	-4.095344
H	7.086728	-2.879382	0.000137
H	6.985309	-1.327224	-0.880499
H	6.984604	-1.327783	0.881667
H	3.619203	-5.637376	0.000191
H	2.122419	-5.210920	0.877871
H	2.125545	-5.210861	-0.882600
H	-7.086744	2.878870	-0.000003
H	-6.985064	1.326742	-0.880662
H	-6.984634	1.327255	0.881495
H	-3.619714	5.637320	-0.000650
H	-2.123974	5.211124	0.878861
H	-2.124890	5.210976	-0.881621

2

N2

N	0.000000	0.000000	0.545362
N	0.000000	-0.000000	-0.545362

7. References

S1 B. Rösch, T. X. Gentner, J. Langer, C. Färber, J. Eyselein, L. Zhao, C. Ding, G. Frenking and S. Harder, *Science*, 2021, **371**, 1125–1128.

S2 Rigaku Oxford Diffraction, *CrysAlisPro Softw. Syst. version 1.171.40.84a*, 2020, Rigaku Corporation, Wroclaw, Poland (compounds **2**-**5**).

S3 R. O. Diffraction, *CrysAlisPro Softw. Syst. version 1.171.42.72a*, 2022, Rigaku Corporation, Wroclaw, Poland (compound **1**).

S4 O. V. Dolomanov, L. J. Bourhis, R. J. Gildea, J. A. K. Howard and H. Puschmann, *J. Appl. Cryst.*, 2009, **42**, 339–341.

S5 G. M. Sheldrick, *Acta Crystallogr. Sect. A Found. Adv.*, 2015, **71**, 3–8.

S6 G. M. Sheldrick, *Acta Crystallogr. Sect. C Struct. Chem.*, 2015, **71**, 3–8.

S7 A. Thorn, B. Dittrich and G. M. Sheldrick, *Acta Crystallogr. Sect. A Found. Crystallogr.*, 2012, **68**, 448–451.

S8 P. van der Sluis and A. L. Spek, *Acta Crystallogr. Sect. A Found. Crystallogr.*, 1990, **46**, 194–201.

S9 M. J. Frisch, G. W. Trucks, H. B. Schlegel, G. E. Scuseria, M. A. Robb, J. R. Cheeseman, G. Scalmani, V. Barone, G. A. Petersson, H. Nakatsuji, X. Li, M. Caricato, A. V. Marenich, J. Bloino, B. G. Janesko, R. Gomperts, B. Mennucci, H. P. Hratchian, J. V. Ortiz, A. F. Izmaylov, J. L. Sonnenberg, D. Williams-Young, F. Ding, F. Lipparini, F. Egidi, J. Goings, B. Peng, A. Petrone, T. Henderson, D. Ranasinghe, V. G. Zakrzewski, J. Gao, N. Rega, G. Zheng, W. Liang, M. Hada, M. Ehara, K. Toyota, R. Fukuda, J. Hasegawa, M. Ishida, T. Nakajima, Y. Honda, O. Kitao, H. Nakai, T. Vreven, K. Throssell, J. A. Montgomery, Jr., J. E. Peralta, F. Ogliaro, M. J. Bearpark, J. J. Heyd, E. N. Brothers, K. N. Kudin, V. N. Staroverov, T. A. Keith, R. Kobayashi, J. Normand, K. Raghavachari, A. P. Rendell, J. C. Burant, S. S. Iyengar, J. Tomasi, M. Cossi, J. M. Millam, M. Klene, C. Adamo, R. Cammi, J. W. Ochterski, R. L. Martin, K. Morokuma, O. Farkas, J. B. Foresman, and D. J. Fox, *Gaussian 16, Revis. A.03* Gaussian, Inc., Wallingford CT, 2016.

S10 A. D. Becke, *J. Chem. Phys.*, 1993, **98**, 5648–5652.

S11 J. P. Perdew, J. A. Chevary, S. H. Vosko, K. A. Jackson, M. R. Pederson, D. J. Singh and C. Fiolhais, *Phys. Rev. B*, 1993, **48**, 4978–4978.

S12 F. Weigend, *Phys. Chem. Chem. Phys.*, 2006, **8**, 1057.

S13 F. Weigend and R. Ahlrichs, *Phys. Chem. Chem. Phys.*, 2005, **7**, 3297–3305.

S14 S. Grimme, S. Ehrlich and L. Goerigk, *J. Comput. Chem.*, 2011, **32**, 1456–1465.

S15 E. D. Glendening, J. K. Badenhoop, A. E. Reed, J. E. Carpenter, J. A. Bohmann, C. M. Morales, P. Karafiloglou, C. R. Landis, F. Weinhold, *NBO 7.0*, Theoretical Chemistry Institute, University of Wisconsin, Madison, 2018.

S16 N. J. R. van Eikema Hommes, *Molecule*, Erlangen, 2018.

S17 R. F. W. Bader, *Chem. Rev.*, 1991, **91**, 893–928.

S18 T. A. Keith, *AIMAII Version 17.01.25*, TK Gristmill Software, Overland Park KS, USA, 2017.
